# Are bis(pyridine)iodine(i) complexes applicable for asymmetric halogenation?†

**DOI:** 10.1039/d1ob01532j

**Published:** 2021-09-09

**Authors:** Daniel von der Heiden, Flóra Boróka Németh, Måns Andreasson, Daniel Sethio, Imre Pápai, Mate Erdelyi

**Affiliations:** a Department of Chemistry – BMC, Uppsala University SE-751 23 Uppsala Sweden mate.erdelyi@kemi.uu.se; b Institute of Organic Chemistry, Research Centre for Natural Sciences H-1117 Budapest Hungary; c Department of Chemistry and Molecular Biology, University of Gothenburg SE-412 96 Gothenburg Sweden; d Department of Chemistry, University J. Selyeho 94505 Komárno Slovakia

## Abstract

Enantiopure halogenated molecules are of tremendous importance as synthetic intermediates in the construction of pharmaceuticals, fragrances, flavours, natural products, pesticides, and functional materials. Enantioselective halofunctionalizations remain poorly understood and generally applicable procedures are lacking. The applicability of chiral *trans*-chelating bis(pyridine)iodine(i) complexes in the development of substrate independent, catalytic enantioselective halofunctionalization has been explored herein. Six novel chiral bidentate pyridine donor ligands have been designed, routes for their synthesis developed and their [N–I–N]^+^-type halogen bond complexes studied by ^15^N NMR and DFT. The chiral complexes encompassing a halogen bond stabilized iodenium ion are shown to be capable of efficient iodenium transfer to alkenes; however, without enantioselectivity. The lack of stereoselectivity is shown to originate from the availability of multiple ligand conformations of comparable energies and an insufficient steric influence by the chiral ligand. Substrate preorganization by the chiral catalyst appears a necessity for enantioselective halofunctionalization.

## Abstract 2

The enantioselectivity of the iodine(i) transfer process from chiral bis(pyridine)iodine(i) complexes to alkenes is explored.

## Graphical abstract

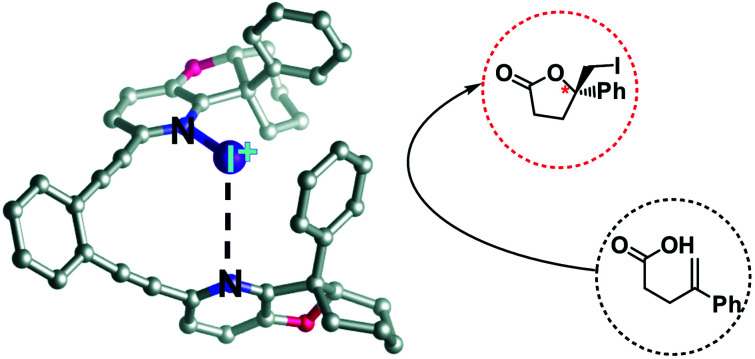

## Introduction

Electrophilic halofunctionalization is a long-established, synthetic transformation that introduces two heteroatoms onto a carbon–carbon double bond.^1,2^ It is frequently applied in the synthesis of bioactive molecules and synthetic building blocks, whose halogen handle can easily be further manipulated. The basis of its understanding has been laid by stereochemical and kinetic studies performed in the early 20^th^ century.^3^ According to the commonly accepted mechanism, electrophilic halofunctionalizations involve the formation of cyclic halonium ions that undergo ring-opening through backside attack by a nucleophile, yielding the *anti*-stereoisomers of the products selectively. An intermediate cyclic halonium ion was first proposed by Roberts and Kimball,^1^ and later NMR spectroscopically proven by Oláh.^4–6^ The formation of acyclic halocarbenium ions, particularly for smaller halogens, was also reported, and even a concerted mechanism has been put forward by Borhan, according to which the nucleophile's electron donation activates the alkene for electrophilic attack by a halenium ion.§§The hypocoordinate (6e) X^+^ is denoted as *halenium*, whereas the halogen of the hypercoordinate (10e) [N−X−N]^+^ complex as *halonium* ion. The former is formally a halogen bond donor (Lewis acid) that may simultaneously interact with two halogen bond acceptors (Lewis bases) and thereby form a 3-center, 4-electron halogen bonded complex. Both halenium and halonium ions are halogen(i) species.^7^ Despite diastereoselectivity, there is generally no facial selectivity in the halenium addition to the olefin, and accordingly the addition results in a racemic product. Due to their high electron affinity, the reactivity of halenium ions is difficult to modulate. The monodentate coordination of halogens makes their positioning into a chiral environment challenging. In the past decade, vast efforts to develop an asymmetric variant of this reaction have been made, however, so far only with limited success whereas vast limitations remain.^7–16^

**Fig. 1:**
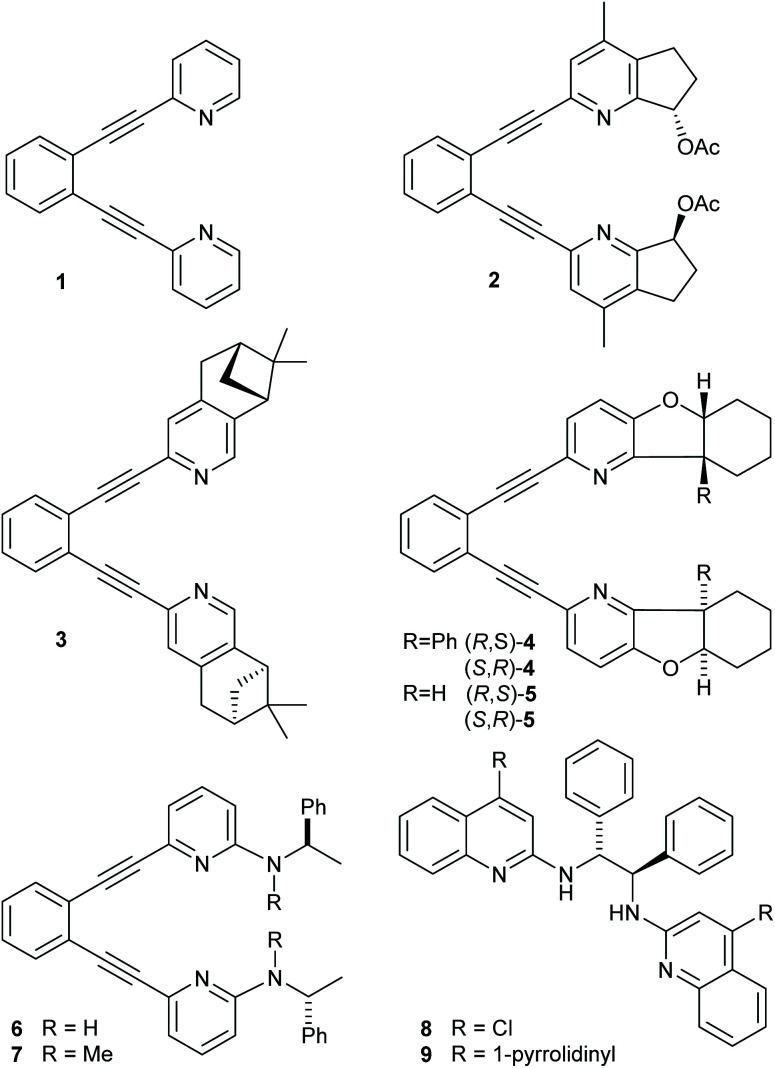
Ligands 1–9 used in iodine(i) transfer reactions. The corresponding iodine(i) complexes are denoted as 1-I, 2-I, … and 8-I.

A reactive halenium ion can be stabilized by a three-center, four-electron halogen bond in a [bis(pyridine)halogen(i)]-type complex,^17^ which allows the rational modulation of halenium ion reactivity.^18^ In such complexes, halonium ions,§ including even chloronium ions,^19^ are stable in solution and hence can be experimentally studied.^17,20–22^ [Bis(pyridine)iodine(i)] tetrafluoroborate was introduced as a mild halogen transfer and oxidation reagent by Barluenga,^23^ and the fundaments for the mechanistic understanding of its halenium transfer reaction were laid by Brown's reaction kinetic studies.^24–26^ [Bis(pyridine)halogen(i)] complexes easily dissociate in solution, leading to rapid ligand scrambling.^27^ To avoid the complications that are caused by such dynamic processes, following some initial studies^22^ the properties of halonium complexes have been primarily investigated using bidentate bis(pyridine)–type ligands,^21^ such as 1 shown in Fig. 1. The (1,2-bis(pyridine-2-ylethynyl)benzene) backbone^28^ promotes the formation of [N–X–N]^+^ complexes with co-planar pyridine rings, providing further helpful geometric control. Following extensive studies of the factors influencing the symmetry and stability of a three-center, four-electron halogen bond of halonium ions,^18–22,29–31^ this backbone also allowed the development of a stable asymmetric halonium complex.^29^ Building on the above studies, halogen bonded halonium ions have been made use of in building complex supramolecular systems,^32–34^ including also the first halonium ion-based halogen bonded molecular framework (XOF).^35^ Their potential applicability in molecular motors has also been recently explored.^36^ The interaction of halogen(i) with silver(i) ions when included into bis(pyridine)-type complexes have lately been reported.^37,38^

The use of a chiral [bis(2-menthylpyridine)bromine(i)] complex for asymmetric halocyclization has been explored by Brown. This yielded only negligible enantioselectivity (2.4–4.8% ee),^25^ likely due to the flexibility and the large distance of the applied 2-menthyl substituent from the reaction center. Herein, we explore whether more rigid (1,2-bis(pyridine-2-ylethynyl)-benzene)-type^18,21^ ligands that encapsulate a halonium ion into a chiral pocket could provide strong enough influence on the stereochemistry of halofunctionalization to make it enantioselective. Accordingly, we synthesized a series of chiral analogues of 1 (Fig. 1). These do not suffer from ligand scrambling,^27^ and may influence the halenium transfer process with both chiral pyridines even upon dissociation of the [N–I–N]^+^ bond. Whereas ligands 2 and 4 hold their chiral centers in a rigid framework and in the proximity of the pyridine nitrogen, 3 has its chiral centers further away, and 5 and 6 allow for geometric adjustments. Ligands 8–9 have originally been introduced by Johnston and coworkers,^39^ and were reported to provide enantioselectivity in halonium transfer when using *N*-iodosuccinimide as iodine-source in the presence of acid, and were here synthesized as positive controls.

## Results and discussion

### Synthesis

**Scheme 1:**
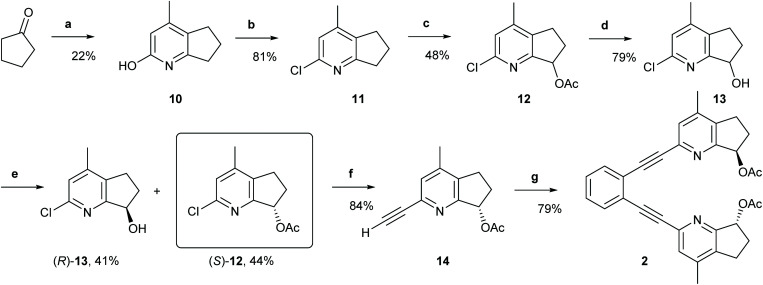
The synthetic route to ligand 2. Reagents and conditions: (a) Ethyl acetoacetate, NH_4_OAc, 135 °C, 20 h.; (b) Phenylphosphonic dichloride, 160 °C, 21 h; (c) (1) Glacial AcOH, H_2_O_2_, 80 °C to r.t., 22 h; (2) Ac_2_O, r.t. to 100 °C, 5 h; (d) LiOH, THF, H_2_O, r.t., 21 h; (e) Novozyme 435, vinyl acetate; r.t., 4 h; (f) (1) Triisopropylacetylene, *trans*-[PdCl_2_(CH_3_CN)_2_], XPhos, Cs_2_CO_3_, CH_3_CN, MW 110 °C, 20 min; (2) TBAF, THF, 0 °C, 2 h; (g) 1,2-diiodobenzene, Pd (PPh_3_)_2_Cl_2_, PPh_3_, CuI, Et_2_NH, MW 110 °C, 20 min. For details, see the ESI.†

**Scheme 2:**
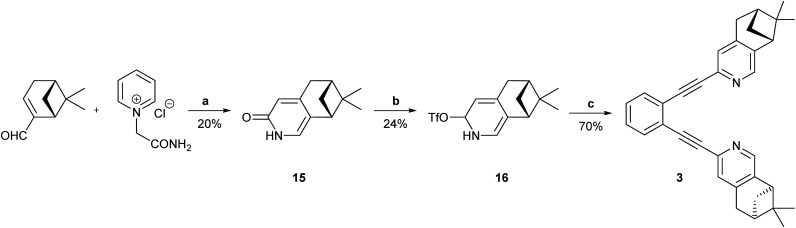
The synthetic route to ligand 3. Reagents and conditions: (a) 1. Piperidine, MeOH, reflux, Ar, 2 h. 2. HCONH_2_, AcOH, 200 °C, 1 h; (b) Tf_2_O, Et_3_N. CH_2_Cl_2_, −50 °C to r.t. 19 h; (c) 1,2-diethynylbenzene, Pd(PPh_3_)_2_Cl_2_, CuI, PPh_3_, DIEA, MW 110 °C, 15 min. For details, see the ESI.†

**Scheme 3:**
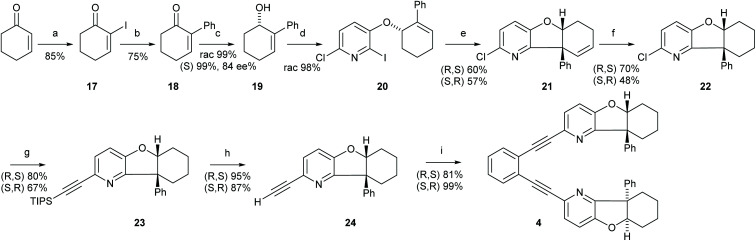
The synthetic route to ligand 4. Reagents and conditions: (a) I_2_, DMAP, K_2_CO_3_, H_2_O/THF (1 : 1), r.t., 2 h.; (b) Pd/C, PhB(OH)_2_, Na_2_CO_3_, DME/H_2_O (1 : 1), r.t., 18 h; (c) CeCl_3_, NaBH_4_, MeOH, 0 °C to r.t. in 2 h; (d) 6-chloro-2-iodopyridine-3-ol, DIAD, PPh_3_, toluene, r.t., 3 days; (e) Pd(PPh_3_)_2_Cl_2_, Ag_2_CO_3_, NEt_3_, toluene, 110 °C, 24 h; (f) Rh/C, H_2_, MeOH; (g) *trans*-[PdCl_2_(CH_3_CN)_2_], XPhos, Cs_2_CO_3_, CH_3_CN, 85 °C, 8 h; (h) (1) NuB4F, THF, 0 °C, 2 h, then (2) 1,2-diiodobenzene, Pd_2_(dba)_3_, PPh_3_, CuI, NEt_3_, 45 °C, 20 h. For details, see the ESI.†

**Scheme 4:**
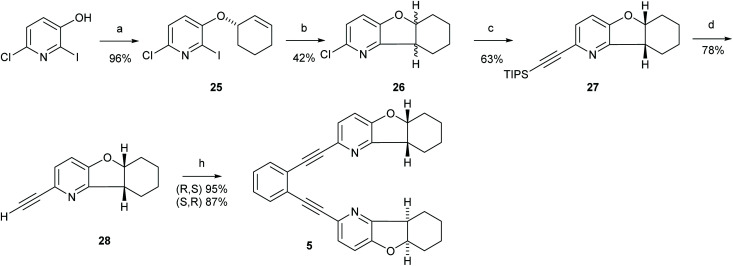
The synthetic route to ligand 5. Reagents and conditions: (a) Bromocylohex-1-ene, K_2_CO_3_, DMF, r.t. 17 h; (b) SmI2, Et_3_N, THF, r.t, 1 h; (c) triisopropylacetylene, *trans*-[PdCl_2_(CH_3_CN)_2_], XPhos, Cs_2_CO_3_, CH_3_CN, 90 °C, 18 h; (d) TBAF, THF, 0 °C, 2 h; (e) 1,2-diiodobenzene, Pd(PPh_3_)_2_Cl_2_, PPh_3_, CuI, Et_2_NH, 60 °C, 19 h. For details, see the ESI.†

(1,2-Bis(pyridine-2-ylethynyl)benzene) (1) was prepared according to a previously established protocol,^21^ whereas ligands 2–7 were synthesized following the reaction routes shown in Schemes 1–4 (for details, see the Experimental section). Ligands 8 and 9 were prepared following Johnston's procedure.^39^

### Stability

**Fig. 2:**
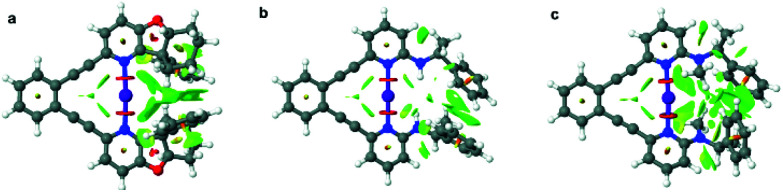
Non-covalent interaction (NCI) plots of 4-I (a), 6-I (b), and 7-I (c), calculated at the ωB97X-D/aug-cc-pVTZ level of theory, with strong repulsions being shown in red, weak repulsion in yellow, weak attraction in green, and strong attraction in blue.

The iodine(i) complexes of 1–6 were achieved by mixing the free ligands with AgBF_4_ in dichloromethane, followed by the addition of I_2_, and removal of the AgI precipitate by centrifugation. The formation of the bis(pyridine)iodine(i) complexes was confirmed by observation of ^15^N NMR chemical shifts (Table 1) characteristic for such complexes.^17,18,21,29^ The iodine(i) complexes of 1–5 are stable at room temperature, whereas those of 6 and 7 can be studied at −5 °C and −35 °C, respectively. Ligands 8–9 do not form stable iodine(i) complexes, but undergo self-iodination.^39^ The stability of the [N−I−N]^+^ complexes remarkably correlates to the steric demand of the pyridine *ortho*-substituent. Hence, 1-I, the iodine(i) complex of 1 that possesses a sterically undemanding alkyne is stable at room temperature in solution, and so is its literature known 2,6-dialkyne analogue.^32^ The iodine(i) complexes 2-I–5-I that have sterically demanding substituents orienting away from the plane of the [N−I−N]^+^ halogen bond are also stable for several hours in solution at room temperature. In contrast, 6-I that has an sp^2^-hybridized *ortho*-pyridine substituent is less stable (Table 1), whereas 7-I was not stable in solution at −35 °C, expectably due to its even more sterically demanding *ortho*-substituent. To confirm our hypothesis on the importance of steric crowding, we performed geometry optimization of complexes 4, 6–7 and visualized the non-covalent interactions (Fig. 2, for details see section 4.3 in the ESI†). It should be noted here that 5-I is stabilized by an intramolecular hydrogen bond, where a filled p-orbital of iodine(i) acts as hydrogen bond acceptor and the amide proton as hydrogen bond donor, analogous to previously reported systems encompassing hydrogen bond-enhanced halogen bonds.^40,41^ In contrast, 7-I is destabilized by the repulsion between the *N*-methyl amine substituent and iodine. No significant steric crowding was predicted for complex 4-I that orients its sterically demanding phenyl group out of the plane of the three-center halogen bond.

**Table 1:** The ^15^N NMR chemical shifts of bis(pyridine)-type ligands, their silver(i) and iodine(i) complexes in CD_2_Cl_2_, and the stability of the bis(pyridine)iodine(i) complexes^a^

Ligand	*δ* ^15^N_lig_	*δ* ^15^N_Ag(I)_	*δ* ^15^N_I(I)_	Stability
1	−65^18^	n.d.	−166^18^	r.t.^18^
**(*S*)-**2	−80	n.d.	n.d.	n.d.
**(*S*,*S*)-**3	−84	−29	−171	r.t.
**(*S*,*R*)-**4	−73	−127	−170	r.t.
**(*S*,*R*)-**5	−74	n.d.	−187.6	n.d.
**(*R*,*R*)-**5	−115 (−285)	−163 (−283)	−199 (−273)	<−5 °C
**(*R*,*R*)-**6	−116 (−296)	−149 (−298)	n.d.	<−35 °C

a The ^15^N NMR chemical shift of the free ligand is denoted as *δ*^15^N_lig_, that of its silver(i) complex as *δ*^15^N_Ag(I)_, of its iodine(i) complex as *δ*^15^N_I(I)_, and the stability of the iodine(i) complex at the conditions under which it is stable for at least 1 hour with < 5% decomposition. n.d. – not determined.

### Halogen(i) transfer reaction

The non-chiral 1-I and its derivatives are mild halonium transfer agents.^17,18^ They react *via* an initial dissociation of one of the pyridines (Scheme 6), followed by coordination of the nucleophilic C=C double bond to the reactive single coordinated [pyridine-iodine(i)]^+^ species, yielding a three membered cyclic iodonium intermediate. The latter undergoes ring opening typically assisted by a nucleophile, which in halocyclizations is present intramolecularly in the substrate. The mechanism of iodine(i) transfer from bis(pyridine) complexes, such as 1, has been recently described based on UV-kinetic and computational data.^31^ Herein we assess whether chiral analogues of 1 presenting chiral functionalities near the coordinating nitrogen may influence the enantioselectivity of iodocyclisation.

**Scheme 5:**
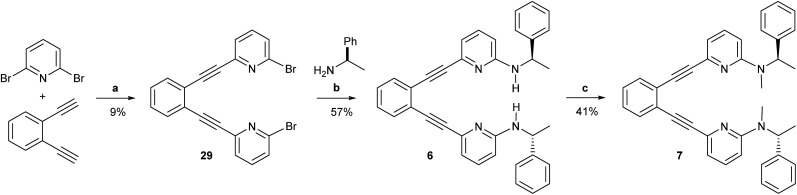
The synthetic route to ligands 6 and 7. Reagents and conditions: (a) Pd(PPh_3_)_2_Cl_2_, CuI NEt_3_, 110 °C, o.n.; (b) Pd_2_(dba)_3_, 1,3-bis(diphenylphosphino)propane, NaOtBu, (*R*)-(+)-1-phenzlethylamine, toluene, 90 °C, 3 h; (c) 1. NaH, DMF, r.t., 1 h, 2. MeI, DMF, r.t., 1 h, 3. NaH, DMF, r.t., 1 h, 4. MeI, DMF, r.t, 1 h. For details, see the ESI.†

**Scheme 6:**
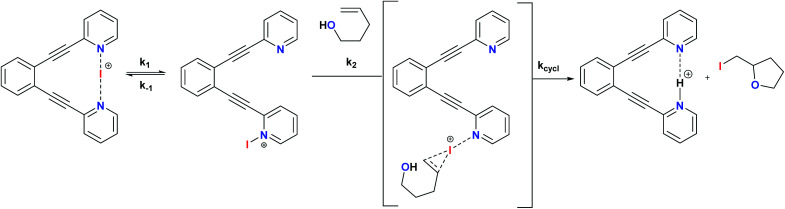
The simplified general mechanism of iodine(i) transfer from [(1,2-bis(pyridine-2-ylethynyl)benzene)iodineI()] (1)-type reagents. A detailed DFT-based description of the mechanism is given in ref. 29. The possible chiral influence of ligands 2–7 on the outcome of the iodocylization of alkenes is assessed herein.†

To explore whether its chiral analogues 2–7 provide stereoinduction in halenium transfer reactions, we used iodocyclization as a model reaction. The iodine(i) complex of **(*R*,*S*)**-4-I was generated *in situ* by addition of stoichiometric I_2_ to a mixture of **(*R*,*S*)**-4-Ag and *trans*-styrylacetic acid at −20 °C, providing halocyclization with the full conversion of the iodine source in toluene, dichloromethane, acetonitrile or tetrahydrofuran as solvents (for details, see the ESI Table S3†). Despite being chiral, 2–5 did not induce significant enantiomeric excess (ee) in the halocyclization of *trans*-styrylacetic acid. As a positive control for iodocyclization, the pyridinium/*N*-iodosuccinimide mediated iodination of 5-phenylhex-5-enoic acid to 6-(iodomethyl)-6-phenyltetrahydro-2*H*-pyran-2-one using 8 and 9 as chiral catalysts, as introduced by Dobisch and Johnston,^39^ was used (Table 2. These provided measurable enantiomeric excesses (ees) confirming our chromatographic method's ability to detect ee (for details see ESI section 1 and 2.1–2.3†). No enantioselectivity was obtained using 2–7 as chiral catalysts.

**Table 2:** Iodocyclization of 5-phenylhex-5-enoic acid induced by 2-I–8-I, the iodine(i) complexes of 2–8 (Fig. 1), as iodine(i) transfer agents^a^

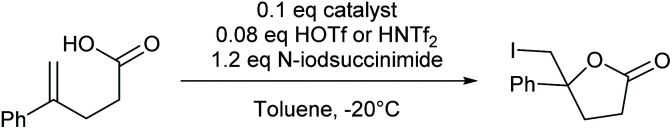		
Catalyst	Acid	ee
**(*S*)-**2	—	0%^b^
**(*S*,*S*)-**3	—	0%^b^
**(*R*,*S*)-**4	HNTf_2_	< 7%
**(*R*,*R*)-**5	—	0%^b^
**(*R*,*R*)-**6	HOTf	3%
8	HNTf_2_	61%
9	HNTf_2_	97%^39^
9	HOTf	93%

a The ees were determined using chiral analytical HPLC using a Lux i-Amylose column (for further details, see Table S12 in section 2.3 in the ESI†).

b The iodine(i) complex of (*S*)-2 was generated *in situ* by addition of I_2_ to its silver(i) complex.

The enantioselectivity of the reactions of 8 and 9 has originally been proposed^39^ to originate from Brønsted acid activation of both *N*-iodosuccinimide and the substrate. The bifunctional catalysts 8 and 9 are supposed to interact with both *N*-iodosuccinimide and the substrate prior to the iodine(i) transfer. Using ^1^H NMR detection, we observed no decomposition when mixing 4 with *N*-iodsuccinimide to generate 4-I, whereas decomposition took place when, 4-H (protonated 4) was used instead (for details, see section 1.10 in the ESI†). Without contradicting the previous findings, this control experiment suggests that protonation is essential for the iodine(i) transfer from *N*-iodosuccinimide to the pyridine–nitrogen. The reactive *N*-iodopyridinium ion is formed upon transferring iodine(i) from *N*-iodosuccinimide to the pyridinium salt *via* I^+^/H^+^ exchange.

**Scheme 7:**
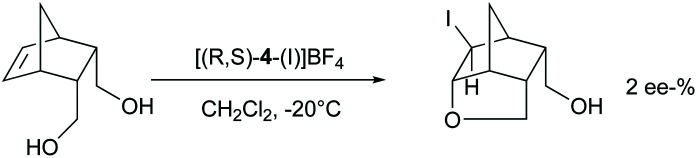
Haloetherification of 5-norbornene-2,3-dimethanol was used to selectively study enantioselectivity of the cyclization step. The enantiomeric excess (ee-%) has been determined following benzylation of the product using benzylanhydride, diisopropylethylamine in CH_2_Cl_2_, at 30 °C for 3 days.

The chiral ligands 2–7 are similar to 8–9 bifunctional molecules, have two pyridine Lewis bases, and allow substrate coordination, and hence could be expected to induce enantioselectivity in halocyclizations. Enantioselectivity may originate from the irreversible addition of iodine(i) to the alkene *via* a halogen-bonded iodine(i) prereactive complex.^42^ In this, the iodine(i) simultaneously coordinates a chiral pyridine and the double bond, providing a chiral bidentate complex of iodine(i). Bidentate iodine(i) complexes are literature known.^17,20^ Alternatively, if the halogen addition step is reversible, the intramolecular nucleophilic attack of the carboxylate oxygen may provide enantioselectivity if the ligand remains coordinated at the time point of the nucleophilic attack. To assess the intermediate that undergoes the nucleophilic attack, we used 5-norbornene-2,3-dimethanol as a substrate (Scheme 7) as for this molecule enantioselectivity in product formation is determined at the time point of the nucleophilic attack, instead of the coordination of iodine(i) to the double bond. In case the chiral pyridine ligand remains coordinated to iodine(i) at the time point of the nucleophilic ring closure, enantioselectivity in the product formation is expected, presuming that the chiral information provided by the bis(pyridine)-ligand is close enough to the reaction centre. If the chiral ligand is dissociated from iodine(i) when the nucleophilic ring closure occurs, a racemic outcome is expected. Using **(*R*,*S*)-**4, no significant ee (2%) in the haloetherification of 5-norbornene-2,3-dimethanol was observed (Scheme 5). This suggests that pyridines do not form stable enough bis-coordinate iodine(i) complexes involving an alkene to induce enantioselectivity, or that the chiral ligand used in this experiment did not provide sufficient enough energy difference of the diaestereotopic transition states.

**Fig. 3:**
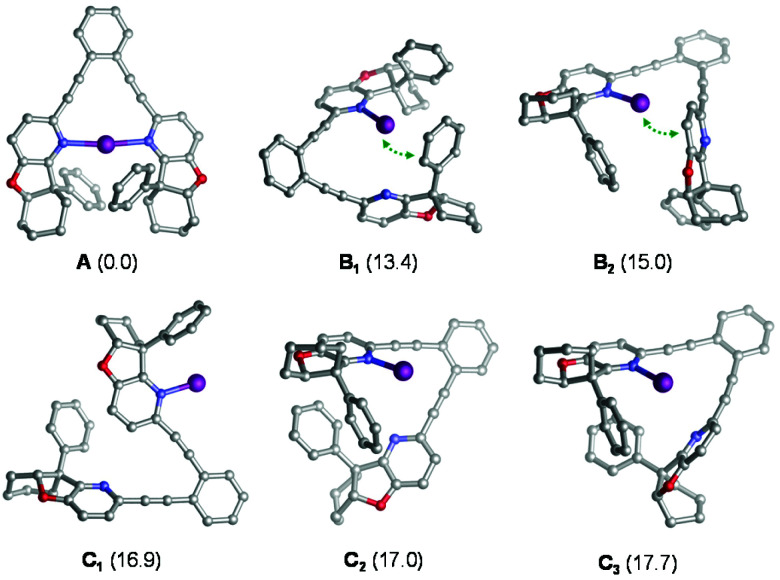
Various forms of complex [2-I]^+^ as obtained from DFT calculations. Relative stabilities are given in kcal mol^−1^ with respect to the symmetric chelating form. Hydrogen atoms are omitted for clarity. Close I^+^−π contacts in B_1_ and B_2_ are highlighted with green dotted arrows.

Iodocyclization reactions induced by 1–7-I are assumed to proceed *via* their singly coordinated open forms, which allow the interaction of the electrophilic iodine(i) with the substrate's olefinic double bond prior to iodocyclization. DFT calculations (see the Experimental section and section 4.1 in the ESI†) carried out for 4-I indicate that the ground state of this complex is the symmetric structure A (Fig. 3), which involves a strong three-center, four-electron [N–I–N]^+^ bond.^20^ The open forms B and C are by at least 13 kcal mol^−1^ less stable and may adopt several conformations, such as B_1_–C_3_ (Fig. 3) In the most favoured open forms B_1_ and B_2_, the pyridine-bound iodine(i) is in close contact either with the phenyl substituent or with the pyridine ring of the second arm of the ligand. Conformers C_1_, C_2_ and C_3_ lack such stabilizing interactions making the iodine(i) readily available for binding the olefinic bond of the ligand. Even if these conformers are considerably less stable than B_1_ and B_2_, they can be regarded as the reactive forms of the 4-I complex towards iodocyclization. Our structural analysis of these energetically close-lying reactive forms suggests that the chiral environment provided by ligand 4 is vaguely defined. The phenyl substituent of the chiral fused ring fragment of the dissociated arm of the ligand is in close vicinity to the iodine(i) providing partial steric shielding, whereas the rest of the fused ring system of the dissociated arm are remotely displaced. Most likely due to the weakness of the steric influence and to the availability of multiple ligand conformations, sufficient stereospecificity in substrate binding is not induced, explaining the inefficiency of 4 to induce enantiomeric excess.

## Conclusions

We report the design and synthesis of six novel, bidentate and chiral bis(pyridine)-type ligands (2–7) and their assessment for enantioselective iodine(i) transfer. The three-center, four-electron [N–I–N]^+^ halogen bond complexes of the ligands were formed through two alternative pathways, either by conversion of the silver(i) complex of the ligands using iodine^21,22^ or by iodine transfer from *N*-iodosuccinimide in the presence of triflic acid.^39^ The stability of bidentate complexes was observed to be limited by the bulkiness of the substituent in the *ortho* position to the pyridine nitrogen, and thus we found that a hydrogen or alkyne (1) was well tolerated, whereas a substituted *ortho*-quarternary carbon (4) or an *ortho*-secondary amide (5) destabilized the iodine(i) complex. The *ortho* tertiary amide of 6 made the complex so unstable that it could not be studied by ^15^N NMR at −35 °C, but only generated *in situ*.

The chiral complexes 2–7-I straightforwardly transferred iodenium ions to a model alkene; however, in our hands without enantioselectivity. We hypothesize that this is due to insufficient substrate preorganization by the chiral catalyst, which appears necessary for the induction of enantioselectivity.^39^ Our control experiments using 5-norbornene-2,3-dimethanol as substrate suggests the irreversible addition of the pyridine bound iodine(i) to the alkene along with rapid ligand dissociation, which prevents stereocontrol by the chiral bis(pyridine) ligands in the nucleophilic ring closure step. Alternatively, the lack of enantioselectivity of the iodine(i) transfer may be explained by the chiral information not being close enough to the reaction centre. Whereas a substrate-independent, catalytic enantioselective halofunctionalization protocol has been a long-sought target of the field, so far all known catalytic enantioselective halogenation protocols are substrate-dependent and require the pre-orientation of the substrate by the catalysts.^7–14,16,39,43^

This study not just provides some guidance for the further development of enantioselective halenium transfer reagents, but also presents a set of chiral bidentate ligands that may find applications in other fields, such as transition metal catalysis. Structurally closely related *trans*-chelating bis(pyridine)-ligands have been applied for complexation of copper,^44–46^ silver,^28,47,48^ iron,^49^ palladium,^28,47,50–56^ mercury,^57^ gold,^58^ and even of carbenes^59^ with several of these complexes having shown synthetically useful catalytic activities.^45,51,54–56,58^

## Experimental section

### General methods and materials

CH_2_Cl_2_ was dried by distillation over CaH_2_, and *n*-hexane by distillation over Na, benzophenone and tetraglyme. For NMR, deuterated solvents were dried by adding 3 Å molecular sieves to freshly opened bottles. All dry solvents were stored over 3 Å molecular sieves in a glovebox. Pyridine was redistilled prior to use. All other chemicals were used without further purification. For all synthesis performed in a glovebox, glassware had been dried at 150 °C in an oven, or *in vacuo*, at least overnight. NMR spectra were recorded on a Bruker Avance Neo 500 MHz spectrometer equipped with a TXO cryogenic probe, or an Agilent MR-400 equipped with an OneNMR probe. Chemical shifts are reported on the *δ* scale (ppm), with the residual solvent signal as an internal reference; CD_2_Cl_2_ (*δ*_H_ 5.32, *δ*_C_ 53.84), CDCl_3_ (*δ*_H_ 7.26, *δ*_C_ 77.16). Nitromethane (*δ*_N_ 0.0 ppm) was used as an external standard for ^15^N. To assign the ^1^H NMR resonances chemical shift (*δ*), multiplicity, coupling constants (*J* Hz) and number of hydrogens were considered. 2D spectra (^1^H,^15^N HMBC, ^1^H,^13^C HSQC, ^1^H,^13^C HMBC, TOCSY, and COSY) also aided correct assignment. Multiplicities are denoted as s (singlet), d (doublet), t (triplet), q (quartet), h (heptet), and m (multiplet). MestReNova 12.0.2. was used to process the NMR spectra.

### (1,2-Bis(pyridine-2-ylethynyl)benzene) (1) was prepared according to a previously established protocol^21^

#### (7*R*,7′*R*)-(1,2-Phenylenebis(ethyne-2,1-diyl))bis(4-methyl-6,7-dihydro-5*H*-cyclopenta[*b*]pyridine-2,7-diyl) diacetate (2)

To an oven-dried microwave vial, Pd(PPh_3_)_2_Cl_2_ (14 mg, 10 mol%), CuI (5 mg, 15 mol%) and PPh_3_ (10 mg, 20 mol%) and compound 13 (76 mg, 0.35 mmol) were added successively under N_2_ gas. The vial was sealed and flushed with N_2_. Deoxygenated, dry Et_2_NH (900 μL) was added and N_2_ was bubbled through the solution. Diiodobenzene (23 μL, 0.18 mmol) was added *via* a syringe. The resulting reaction mixture was bubbled with N_2_ and heated at 120 °C for 20 min under microwave irradiation. Next, the flask was allowed to cool to room temperature, the solvent was removed under reduced pressure, the crude was dissolved in CH_2_Cl_2_, and washed twice with aqueous NH_4_Cl solution. The organic phase was dried with Na_2_SO_4_ and the solvent was removed under reduced pressure to yield a brown oil, which was purified by flash chromatography with hexane : EtOAc (6 : 4) eluent. Compound 2 was obtained as an orange solid (61 mg, 67% yield). ^1^H NMR (500 MHz, CDCl_3_) *δ* 7.63 (AA′ part of AA′BB′, 2H, H-14), 7.39 (s, 1H, H-3), 7.39–7.42 (2 × s, 1H, H-3) 7.34 (BB′ part of AA′BB′, 2H, H-13), 6.06 (dd, *J* = 7.4, 4.1 Hz, 1H, H-7), 3.05–2.98 (m, 1H, H-5), 2.88–2.79 (m, 1H, H-5), 2.70–2.60 (m, *J*, 1H, H-6), 2.26 (s, 3H, Me-4), 2.14–2.07 (m, 4H, Me–9 and H-6). ^13^C NMR (126 MHz, CDCl_3_) *δ* 170.9 (C-8), 160.5 (C-7a), 144.2 (C-4), 142.8 (C-2), 137.2 (C-4a), 132.4 (C-13), 128.7 (C-14), 128.6 (C-3), 125.6 (C-12), 93.3 (C-11), 93.2 (C-10), 87.8 (C-7), 30.6 (C-6), 26.8 (C-5), 21.5 (Me–9), 18.6 (Me–4). ^15^N NMR (CDCl_3_) *δ* –80.2.

#### 1,2-Bis(((6*R*,8*R*)-7,7-dimethyl-5,6,7,8-tetrahydro-6,8-methanoisoquinolin-3-yl)ethynyl)benzene (3)

To a flame dried N_2_ purged microwave vial, CuI (13 mg, 0.07 mmol), Pd(PPh_3_)_2_Cl_2_ (50 mg, 0.07 mmol) and PPh_3_ (37 mg, 0.14 mmol) were added under N_2_. Deoxygenated and dry Et_2_NH (3 mL; purified by redistillation, 99.5%) was then added. N_2_ was bubbled through this solution for 15 min. Compound 15 (470 mg, 1.5 mmol) in dry Et_2_NH (3 mL) was added to the flask. The resulting reaction mixture was bubbled with N_2_ for 5 min. Next, 1,2-diethynylbenzene (105 mg, 0.8 mmol) dissolved in dry Et_2_NH (3 mL) was added. After heating the reaction at 60 °C for 15 h, the reaction mixture was allowed to cool to rt, and was filtered through a Celite plug (solvent CH_2_Cl_2_). The organic phase was concentrated under reduce pressure, and then the crude was dissolved with CH_2_Cl_2_ and washed trice with saturated aqueous NH_4_Cl solution. The organic phase was dried with Na_2_SO_4_ and the solvent was removed under reduce pressure to yield a brown oil, which was purified by flash chromatography with Et_2_O : pentane (7 : 3) as eluent, and further purified using ethyl/buthyl phophonic acid silica (Sigma-Aldrich) with MeOH as eluent. Compound 3 was obtained as an orange foam (265 mg, 76%). ^1^H NMR (500 MHz, CD_2_Cl_2_) *δ* 8.16 (s, 1H, H1), 7.63 (1H, H13), 7.59 (t, *J* = 0.9 Hz, 1H, H4), 7.38 (1H, H14), 2.97 (d, *J* = 2.8 Hz, 2H, H5), 2.85 (t, *J* = 5.5 Hz, 1H, H8), 2.72 (dt, *J* = 9.6, 5.5 Hz, 1H, H9′), 2.31 (tt, *J* = 5.5, 2.8 Hz, 1H, H6), 1.42 (s, 3H, CH_3_7′), 1.21 (d, *J* = 9.6 Hz, 1H, H16′), 0.64 (s, 3H, CH_3_7′′). ^13^C NMR (126 MHz, CD_2_Cl_2_) *δ* 147.1 (CH, C1), 145.3 (C, C8a), 143.3 (C, C4a), 141.6 (C, C3), 132.5 (CH, C13), 129.1 (CH, C14), 127.8 (CH, C4), 126.2 (C, C12), 94.3 (C, C10), 86.8 (C, C11), 45.2 (CH, C8), 40.7 (CH, C6), 39.7 (C, C7), 33.1 (CH_2_, C5), 32.2 (CH_2_, C9), 26.3 (CH_3_, C7′′), 21.7 (CH_3_, C7′). HRMS (MALDI TOF) calcd for C_34_H_32_N_2_ 469.2643 [M + H]^+^, found *m*/*z* 469.2612. [*α*]^20^_D_ −36.5 (c 0.135, CH_2_Cl_2_).

#### 1,2-Bis-[((5a*S*,9a*R*)-9a-phenyl-5a,6,7,8,9,9a-hexahydrobenzofuro[3,2-*b*]pyridin-2-yl)-ethinyl]benzene (4)

An oven dried microwave vial was charged with (5a*S*,9a*R*)-2-ethynyl-9a-phenyl-5a,6,7,8,9,9a-hexahydrobenzofuro[3,2-*b*]pyridine (23) (461 mg, 1.60 mmol, 2.20 eq.), Pd_2_(dba)_3_ (26.7 mg, 0.029 mmol, 0.04 eq.), CuI (10.4 mg, 0.546 mmol, 0.10 eq.), PPh_3_ (57.2 mg, 0.218 mmol, 0.40 eq.), was evacuated and flushed with N_2_. Dry NEt_3_ (9 ml), and 1,2-diiodobenzene (95.1 μL, 240 mg, 0.728 mmol, 1.00 eq.) were added and the mixture was evacuated and flushed with N_2_ trice to degass. The mixture was stirred at 45 °C for 20 h, until complete conversion. The mixture was purified by preparative TLC (silica, ∼45 mg crude material/PTLC (2 mm silica, 20 × 20 cm), Et_2_O/pentane 20 : 80 v/v, 3 runs per PTLC) to give (5a*S*,9a*R*)-4 (370 mg, 0.592 mmol, 81%. Alternatively, (5a*R*,9a*S*)-4 was synthesized (340 mg, 0.544 mmol, 99%. Analytical HPLC was performed on a Lux® 5 μm Amylose-1 column (250 × 4.6 mm) with H_2_O/CH_3_CN 30 : 70 eluent at 1 ml min^−1^ flow rate, giving (5a*S*,9a*R*)-4 at 18.02 min, and (5a*R*,9a*S*)-4 at 17.65 min.

(5a*S*,9a*R*)-4^1^H NMR (500 MHz, CDCl_3_): *δ* 7.61–7.57 (m, 2H, 3-H, 6-H), 7.49 (d, *J* = 8.3 Hz, 2H, 3′′-H), 7.42–7.37 (m, 2H, 2′′′-H, 6′′′-H), 7.33–7.27 (m, 6H, 3′′′-H, 5′′′-H, 4-H, 5-H), 7.23–7.19 (m, 2H, 4′′′-H), 6.82 (d, *J* = 8.3 Hz, 2H, 4′′-H), 5.08 (t, *J* = 5.0 Hz, 2H, 5a′′-H), 2.29–2.19 (m, 4H, 9′′-H), 2.05–1.97 (m, 2H, 6′′-H_a_), 1.94–1.84 (m, 2H, 6′′-H_b_), 1.69–1.50 (m, 2H, 7′′-H_a_, 7′′-H_b_, 8′′-H_a_), 1.48–1.37 (m, 2H, 8′′-H_b_). ^13^C NMR (126 MHz, CDCl_3_): *δ* 157.5 (C-10), 152.9 (C-5′′), 144.4 (C-1′′′), 135.6 (C-2′′), 131.9 (C-3, C-6), 128.6 (C-3f′′′, C-5′′′), 128.2 & 128.1 (C-3′′, C-4, C-5), 127.3 (C-2′′′, C-6′′′), 126.8 (C-4′′′), 125.9 (C-1, C-2), 116.6 (C-4′′), 93.8 (C-1′), 89.5 (C-5a′′), 86.5 (C-2′), 51.8 (C-9a′′), 33.2 (C-9′′), 27.2 (C-6′′), 21.1 (C-8′′), 19.3 (C-7′′). ^15^N NMR (51 MHz, CD_2_Cl_2_): *δ*–72.8 ppm (CH_3_NO_2_ as 0 ppm). LC-MS (ESI), [*m*/*z*]: 313.3 [M + 2H]^2+^, 625.4 [M + H]^+^.

(5a*R*,9a*S*)-4^1^H NMR (500 MHz, CDCl_3_): *δ* 7.61–7.56 (m, 2H, 3-H, 6-H), 7.49 (d, *J* = 8.3 Hz, 2H, 3′′-H), 7.42–7.36 (m, 4H, 2′′′-H, 6′′′-H), 7.34–7.24 (m, 6H, 3′′′-H, 5′′′-H, 4-H, 5-H), 7.24–7.19 (t, *J* = 7.3, 1.6 Hz, 2H, 4′′′-*H*), 6.82 (d, *J* = 8.2 Hz, 2H, 4′′-*H*), 5.08 (t, *J* = 5.0 Hz, 2H, 5a-*H*), 2.29–2.20 (m, 4H, 9′′-*H*), 2.05–1.98 (m, 2H, 6′′-*H*_a_), 1.94–1.85 (m, 2H, 6′′-*H*_b_), 1.68–1.51 (m, 6H, 7′′-*H*_a_, 7′′-*H*_b_, 8′′-*H*_a_), 1.48–1.37 (m, 2H, 8′′-*H*_b_). ^13^C NMR (126 MHz, CDCl_3_): *δ* 157.5 (C-10), 152.8 (C-5′′), 144.4 (C-1′′′), 135.5 (C-2′′), 131.9 (C-3, C-6), 128.6 (C-3′′′, C-5′′′), 128.2 and 128.1 (C-3′′, C-4, C-5), 127.3 (C-2′′′, C-6′′′), 126.8 (C-4′′′), 125.8 (C-1, C-2), 116.6 (C-4′′), 93.8 (C-1′), 89.5 (C-5a′′), 86.5 (C-2′), 51.8 (C-9a′′), 33.2 (C-9′′), 27.2 (C-6′′), 21.1 (C-8′′), 19.3 (C-7′′). ^15^N NMR (51 MHz, CD_2_Cl_2_): *δ* −72.8 ppm. LC-MS (ESI), [*m*/*z*]: 313.3 [M + 2H]^2+^, 625.4 [M + H]^+^.

#### 1,2-Bis-[((5a*S*,9a*R*)-9a-phenyl-5a,6,7,8,9,9a-hexahydro-benzofuro[3,2-*b*]pyridin-2-yl)-ethinyl]benzenesilver(i) tetrafluoroborate (4-Ag)

1,2-Bis-[((5a*S*,9a*R*)-9a-phenyl-5a,6,7,8,9,9a-hexahydrobenzofuro[3,2-*b*]pyridin-2-yl)-ethinyl]benzene (20 mg, 0.032 mmol, 1.00 eq.) were dissolved in CH_2_Cl_2_ (2 ml) and AgBF_4_ (5.9 mg, 0.030 mmol, 0.95 eq.) was added and stirred for 1 h. *n*-Hexane (4 ml) was added and the product was precipitated. The solution was pipetted off and the solid was washed with *n*-hexane and dried in vacuum giving 23 mg (0.028 mmol, 88%)product. ^1^H NMR (500 MHz, CD_2_Cl_2_): *δ* 7.77 (d, *J* = 8.4 Hz, 2H, 3′′-H), 7.69 (dd, *J* = 5.8, 3.3 Hz, 2H, H-3 and H-6), 7.47 (dd, *J* = 5.9, 3.3 Hz, 2H, H-4 and H-5), 7.36 (d, *J* = 8.5 Hz, 2H, H-4′′), 7.32 (t, *J* = 7.4 Hz, 4H, H-3′′ and H-5′′), 7.26 (t, *J* = 7.3 Hz, 2H, H-4′′′), 6.76 (d, *J* = 7.5 Hz, 4H, H-2′′′ and H-6′′′), 4.71 (t, *J* = 5.7 Hz, 1H, H-5a′′), 1.95 (ddt, *J* = 13.8, 8.7, 4.9 Hz, 2H, H-6_a_′′), 1.50–1.44 (m, 2H, H-6_b_′′), 1.41–1.10 (m, 8H, H-7′′′, H-9′′), 1.03–0.93 (m, 2H, H-8_a_′′), 0.72–0.56 (m, 2H, H-8_b_′′). ^13^C NMR (126 MHz, CDCl_3_) *δ* 157.7 (C-10′′), 155.2 (C-5′′), 145.4 (C-1′′′), 136.4 (C-2′′), 132.5 (C-4 and C-5), 131.3 (C-3′′), 130.6 (C-3 and C-6), 130.1 (C-3′′′ and C-5′′′), 128.7 (C-4′′′), 127.0 (C-2′′′ and C-6′′′), 123.5 (C-1, C-2), 121.4 (C-4′′), 92.6 (C-5a′′), 92.5 (C-2′), 91.4 (C-1′), 53.9 (C-9a′′), 31.6 (C-9′′), 27.6 (C-6′′), 20.8 (C-8′′), 17.7 (C-7′′). ^15^N NMR (51 MHz, CD_2_Cl_2_) *δ* −127 ppm.

#### 1,2-Bis-[((((5a*S*,9a*R*)-9a-phenyl-5a,6,7,8,9,9a-hexahydro-benzofuro[3,2-*b*]pyridin-2-yl)-ethinyl)benzene)iodine(i)] tetrafluoroborate (4-I)

To a solution of the corresponding [bis(pyridine)silver(i)]-type complex (1.0 eq.) a solution of I_2_ was added slowly. In most cases the addition was carried out under cooling. The solution of the silver complex was cooled down to −78 °C by an CH_3_CN dry-ice bath and a fresh solution of I_2_ in the corresponding solvent was added *via* a long syringe needle in a way, that the solution is precooled in the needle before getting injected to the solution ideally resulting in a two layer system that is mixed after both layers have been cooled down to −78 °C. Next the sample was put to a cooling system and warmed up to the desired reaction/measurement temperature. The reaction is quantitative. ^1^H NMR (500 MHz, CD_2_Cl_2_, −35 °C) *δ* 7.72 (d, *J* = 8.5 Hz, 2H. 4′′-H), 7.69 (dd, *J* = 5.8, 3.3 Hz, 2H, 3-H and 6-H), 7.52 (dd, *J* = 5.7, 3.3 Hz, 2H, 4-H and 5-H), 7.50 (d, *J* = 8.5 Hz, 2H, 3′′-H), 7.44 (t, *J* = 7.6 Hz, 4H, 3′′′-H, 5′′′-H), 7.36 (t, *J* = 7.3 Hz, 2H, 4′′′-H), 7.17 (d, *J* = 7.7 Hz, 2H, 2′′′-H and 6′′′-H), 4.56 (d, *J* = 3.0 Hz, 2H, 5′′-H), 2.12–2.02 (m, 2H, 6′′-H_a_), 1.84–1.75 (m, 2H, 9′′-H_a_), 1.76–1.67 (m, 2H, 6′′-H_b_ and 7′′-H_a_), 1.67–1.53 (m, 2H, 8′′-H_a_), 1.52–1.43 (m, 2H, 7′′-H_b_), 1.43–1.32 (m, 2H, 8′′-H_b_), 1.08 (ddd, *J* = 14.5, 9.6, 4.6 Hz, 2H, 9′′-H_b_). ^13^C NMR (125 MHz, CD_2_Cl_2_, −35 °C) *δ* 156.5 (C-10′′), 156.4 (C-5′′), 141.4 (C-1′′), 136.2 (C-2′′), 132.7 (C-3, C-6), 131.9 (C-4′′), 130.3 (C-3, C-5), 129.5 (C-3′′′, C-5′′′), 127.9 (C-4′′′), 127.5 (C-2′′′, C-6′′′), 124.4 (C-1, C-2), 122.5 (C-3′′), 96.9 (C-1′), 92.5 (C-5a′′), 92.0 (C-2′), 54.0 (C-9a′′), 53.9 (C-9′′), 29.5 (C-6′), 23.7 (C-8′′), 20.5 (C-8′′), 16.6 (C-7′′). ^15^N NMR (51 MHz, CD_2_Cl_2_) *δ* −170.2.

#### 1,2-Bis(((5a*S*,9a*S*)-5a,6,7,8,9,9a-hexahydrobenzofuro[3,2-*b*]pyridin-2-yl)ethynyl)benzene (5)

To an oven-dried microwave vial, Pd(PPh_3_)_2_Cl_2_ (5 mg, 9 mol%), CuI (2 mg, 14 mol%) and PPh_3_ (20 mg, 20 mol%) were added under N_2_. The vessel was sealed and filled with N_2_. Compound 27 (31 mg, 0.15 mmol) in deoxygenated dry DEA (700 μL) was added, and N_2_ was bubbled through the solution. A solution of diiodobenzene in Et_2_NH (90 μL, 0.76 nM, 0.077 mmol) was added *via* a syringe, and the resulting reaction mixture was bubbled with N_2_ and heated at 60 °C for 19 h. The flask was allowed to cool to r.t., the solvent was removed under reduced pressure, the crude was dissolved with CH_2_Cl_2_ and washed twice with aq. NH_4_Cl solution. The organic phase was dried with Na_2_SO_4_, and the solvent was removed under reduce pressure to yield a brown oil, which was purified by flash chromatography with pentane : Et_2_O (1 : 1) as eluent. Compound 5 was obtained as a yellow solid (17 mg, 47% yield) along with the monocoupled analogue. ^1^H NMR (400 MHz, CD_2_Cl_2_) *δ* 7.60 (m, 1H, H13), 7.51 (dt, *J* = 8.2, 0.7 Hz, 1H, H3), 7.35 (m, 1H, H14), 6.99 (d, *J* = 8.2 Hz, 1H, H4), 4.86 (dt, *J* = 7.2, 5.2 Hz, 1H, H5a), 3.30 (q, *J* = 7.2 Hz, 1H, H9a), 2.04–1.86 (m, 3H, C6 and C9), 1.79–1.66 (m, 1H, C9), 1.58–1.39 (m, 4H, C7 and C8). ^13^C NMR (101 MHz, CD_2_Cl_2_) *δ* 157.3 (C9b), 153.7 (C2), 135.0 (C4a), 132.3 (C13), 128.8 (C14), 127.8 (C3), 126.1 (C12), 116.3 (C4), 94.1 (C10), 86.3 (C11), 83.8 (C5a), 41.6 (C9a), 28.2 (C6), 26.7 (C9), 22.7 (C8), 20.8 (C7). ^15^N NMR (CD_2_Cl_2_) *δ* −71.25 (ppm). Enantiomer 5b [*α*]^20^_D_ +130 (c 2 mg mL^−1^, CH_2_Cl_2_).

#### 1,2-Bis((6-(*N*-((1*R*)-1-phenylethyl)amino)pyridin-2-yl)ethinyl)benzene (6)

A microwave vial was charged with 1,2-bis(2-(6-bromopyridin-2-yl)ethinyl)benzene (29) (155 mg, 0.354 mmol, 1.00 eq.), Pd_2_(dba)_3_ (16.2 mg, 0.0177 mmol, 0.05 eq.), 1,3-bis(diphenylphosphino)propane (14.6 mg, 0.035 mmol, 0.10 eq.), and NaO*t*Bu (81.6 mg, 0.85 mmol, 2.40 eq.) was alternatingly evacuated and refilled with N_2_ trice. Next toluene and (*R*)-(+)-1-phenzlethylamine (99.5 μL, 0.778 mmol, 2.20 eq.) were added. The mixture was heated to 90 °C until complete conversion of 1,2-bis(2-(6-bromopyridin-2-yl)ethinyl)benzene was observed by LC-MS. The reaction mixture was filtered to a mixture of brine and Et_2_O. The aqueous phase was extracted trice with Et_2_O and the combined organic layer was dried with Na_2_SO_4_. Column chromatography (silica, CH_2_Cl_2_/CH_3_OH 100 : 0 to 96 : 4) provided pure 25 (104 mg, 0.201 mmol, 57%). ^1^H NMR (500 MHz, CDCl_3_): *δ* 7.53 (dd, *J* = 5.8, 3.3 Hz, 2H, H-3, H-6), 7.28–7.20 (m, 10H, H-4, H-5, H-5′′′, H-6′′′), 7.16–7.12 (m, 4H, H-4′′, H-7′′), 6.86 (dd, *J* = 7.3, 0.8 Hz, 2H, H-3′′), 6.02 (dd, *J* = 8.5, 0.8 Hz, 2H, 5′′-H), 5.02 (d, *J* = 6.2 Hz, 2H, NH), 4.56 (p, *J* = 6.5 Hz, 2H, H-1′′′), 1.44 (d, *J* = 6.7 Hz, 6H, H-2′′′). ^13^C NMR (126 MHz, CDCl_3_): *δ* 158.0 (C-6′′), 144.5 (C-3′′′), 141.6 (C-2′′), 137.7 (C-4′′), 132.3 (C-3, C-6), 128.9 (C-6′′′), 128.5 (4, 5), 127.3 (C-7′′′), 126.0 (C-5′′), 125.8 (C-1, C-2), 117.7 (C-3′′), 106.4 (C-5′′), 93.5 (C-2′), 86.7 (C-1′), 52.3 (C-1′′′), 24.7 (C-2′′′). ^15^N NMR (51 MHz, CDCl_3_): *δ* −285 (N-1′′′), −115 (N-1′′). HRMS (TurboSpray TOF) calcd for C_36_H_30_N_4_ 519.2549, found *m*/*z* 519.2566.

#### [(1,2-Bis((6-(*N*-((1*R*)-1-phenylethyl)amino)pyridin-2-yl)ethinyl)benzene)silver(i)] tetrafluoroborate (6-Ag)

The desired ligand (1.0 eq.) and the corresponding silver(i) salt (1.0 eq.) were stirred in the desired solvent (CH_2_Cl_2_). In selected cases, the complex was precipitated by addition of *n*-hexane (∼50 v-%) addition. The precipitate was filtered of and dried in vacuum. The reaction was quantitative. ^1^H NMR (500 MHz, CD_2_Cl_2_) *δ* 7.67 (dd, *J* = 5.8, 3.3 Hz, 2H, 3-H and 6-H), 7.46 (dd, *J* = 5.8, 3.3 Hz, 4H, 4−H, 5−H and 4′′-H), 7.44 (d, *J* = 7.1 Hz, 4H, 2′′′′-H and 6′′′′-H), 7.34 (t, *J* = 7.6 Hz, 4H, 3′′′′-H and 5′′′′-H), 7.29–7.19 (m, 2H, 4′′′′-H), 7.03 (d, *J* = 7.2 Hz, 2H, 3′′-H), 6.76 (s, 2H, 4′′′-H), 6.44 (d, *J* = 8.7 Hz, 2H, 5′′-H), 4.61 (p, *J* = 6.8 Hz, 2H, 1′′′-H), 1.65 (d, *J* = 6.9 Hz, 6H, 2′′′-H). ^13^C NMR (125 MHz, CD_2_Cl_2_) *δ* 158.9 (C-6′′), 144.1 (C-2′′), 141.6 (C-1′′′′), 140.2 (C-4′′), 132.7 (C-3, C-6), 130.2 (C-4, C-5), 129.4 (C-3′′′′, C-5′′′′), 127.9 (C-4′′′′), 126.4 (C-2′′′′, C-6′′′′), 124.4 (C-1, C-2), 118.5 (C-3′′), 110.2 (C-5′′), 93.1 (C-2′), 89.9 (C-1′), 54.3 (C-1′′′), 25.2 (C-2′′′). ^15^N NMR (125 MHz, CD_2_Cl_2_) *δ* −283 (NH), −163 (N-Ag).

#### [(1,2-Bis((6-(*N*-((1*R*)-1-phenylethyl)amino)pyridin-2-yl)ethinyl)benzene)iodine(i)]tetrafluoroborate (6-I)

This compound is unstable, and could not be characterized despite several attempts. Instead the protonated from, 6-H, was formed. Therefore the 6-I mediated halogenations were performed with *in situ* generated 6-I using NIS. To a solution of 6, HOTf (1 eq.) was added, followed by NIS and the mixture was stirred at r.t. The iodine(i) transfer was confirmed by ^15^N NMR indicating transfer of iodine(i) to 6 and proton transfer to succinimide. The amount of formed [bis(pyridine)iodine(i)] complex is not stoichiometric, but enough to detect its presence by ^15^N NMR.

#### 1,2-Bis((6-(*N*-methyl-*N*-((1*R*)-1-phenylethyl)amino)-pyridin-2-yl)ethinyl)benzene (7)

A microwave vial was charged with 1,2-bis((6-(*N*-((1*R*)-1-phenylethyl)amino)pyridin-2-yl)ethinyl)benzene (6) (173 mg, 0.334 mmol, 1 eq.) and dimethylformamide (1.65 mL). NaH (60 w/w% in mineral oil, 13.4 mg, 0.334 mmol, 1.00 eq.) was added and the mixture was stirred for 1 h, then MeI (20.8 μL, 0.334 mmol, 1.00 eq.) was added and the mixture was stirred for 1 h. Thbis procedure was repeated a second time. The reaction mixture was mixed with brine and extracted with CH_2_Cl_2_. The combined organic layer was dried with Na_2_SO_4_. Column chromatography (silica, *n*Hex/EtOAc 90 : 10, *R*_f_ = 0.41 product, *R*_f_ = 0.16 mono-methylated product) gave pure 26 (75 mg, 0.137 mmol, 41%). Analytical HPLC was performed using a Lux® 5 μm i-Amylose-1 column (250 × 4.6 mm) using hexane/iPrOH (90 : 10 to 20 : 80 [10 min], 20 : 80 [10 min] 0.75 ml min^−1^) providing (1*R*)-26 with 7.882 min retention time. ^1^H NMR (500 MHz, CD_2_Cl_2_) *δ* 7.67–7.60 (AA′BB′, 2H, H-3, H-6), 7.40–7.34 (AA′BB′, 2H, H-4, H-5), 7.35–7.23 (m, 12H, H-4′′, H-2′′′′, H-3′′′′, H-4′′′′), 6.96 (d, *J* = 7.2 Hz, 2H, H-3′′), 6.50 (d, *J* = 8.6 Hz, 2H, H-5′′), 6.19 (q, *J* = 7.0 Hz, 2H, H-1′′′), 2.73 (s, 6H, H-4′′′), 1.56 (d, *J* = 7.0 Hz, 6H, H-2′′′). ^13^C NMR (126 MHz, CD_2_Cl_2_) *δ* 159.2 (C-6′′), 143.0 (C-1′′′), 141.4 (C-2′′), 137.8 (C-4′′), 132.8 (C-6), 129.0 (C-5), 128.9 (C-3′′′′), 127.5 (C-2′′′′), 127.3 (C-4′′′′), 126.1 (C-1, C-2), 116.8 (C-3′′), 106.4 (C-5′′), 94.7 (C-2′), 86.1 (C-1′), 52.5 (C-1′′), 30.6 (C-4′′′), 16.6 (C-2′′′). ^15^N NMR (51 MHz, CD_2_Cl_2_): *δ* −296 (N3′′′), −116 (N1′′). HRMS (TurboSpray TOF) calcd for C_38_H_34_N_4_ [M + H]^+^ 547.2862, found *m*/*z* 547.2838.

#### [(1,2-Bis((6-(*N*-methyl-*N*-((1*R*)-1-phenylethyl)amino)pyridin-2-yl)ethinyl)benzene)silver(i)] tetrafluoroborate (7-Ag)

The desired ligand (1.0 eq.) and the corresponding silver(i) salt (1.0 eq.) were stirred in the desired solvent (CH_2_Cl_2_). In selected cases, the complex was precipitated by addition of *n*-hexane (∼50 v-%) addition. The precipitated was filtered of and dried in vacuum. The reaction was quantitative. ^1^H NMR (500 MHz, CD_2_Cl_2_) *δ* 7.65–7.60 (m, 4H, H-3, H-6, and H-4′′), 7.42 (dd, *J* = 5.8, 3.3 Hz, 2H, H-4 and H-5), 7.21 (dd, *J* = 8.2, 7.1 Hz, 4H, H–3′′′′, H–5′′′′), 7.14–7.09 (m, 8H, H-3′′, H-2′′′′, H-4′′′′, and H-6′′′′), 6.84 (d, *J* = 8.7 Hz, 2H, H-5′′), 5.08 (q, *J* = 7.1 Hz, 2H, H-1′′′), 2.85 (s, 6H, H-4′′′), 1.46 (d, *J* = 7.0 Hz, 6H, H-2′′′). ^13^C NMR (125 MHz, CD_2_Cl_2_) *δ* 162.1 (C-6′′), 141.8 (C-2′′), 141.2 (C-1′′′′), 140.7 (C-4′′), 133.1 (C-3, C-6), 130.5 (C-4, C-5), 129.6 (C-3′′′′, C-5′′′′), 128.6 (C-4′′′′), 126.7 (C-2′′′′, C-6′′′′), 124.1 (C-1, C-2), 118.9 (C-3′′), 113.4 (C-5′′), 93.5 (C-2′), 89.7 (C-1′), 59.5 (C-1′′′), 33.1 (C-4′′′), 18.4 (C-2′′′). ^15^N NMR (125 MHz, CD_2_Cl_2_) *δ* −298 (NMe), −149 (N-Ag).

(1*R*,2*R*)-*N*^1^,*N*^2^-Bis(4-chloroquinolin-2-yl)-1,2-diphenylethane-1,2-diamine (8) and (1*R*,2*R*)-1,2-diphenyl-N,^1^N^2^-bis(4-(pyrrolidin-1-yl)quinolin-2-yl)ethane-1,2-diamine (9) were prepared following the procedure of Johnston.^39^

### Synthesis of (7*R*,7′*R*)-(1,2-phenylenebis(ethyne-2,1-diyl))bis(4-methyl-6,7-dihydro-5*H*-cyclopenta[*b*]pyridine-2,7-diyl) diacetate (2)

#### 4-Methyl-6,7-dihydro-5*H*-cyclopenta[*b*]pyridin-2-ol (10)^60,61^

A mixture of cyclopentanone (4.75 g, 56 mmol), ethyl acetoacetate (7.35 g, 56 mmol) and ammonium acetate (4.32 g, 56 mmol) was refluxed for 20 h. The reaction mixture was then allowed to cool to room temperature and following dilution with ether (25 mL) was allowed to stand overnight during which time the product precipitated from the reaction mixture. The product was isolated by filtration, washed with Et_2_O and recrystallized from EtOH to afford compound 10 (1.8 gg, 22%) as a yellow solid. The spectroscopic data was in agreement with that reported in the literature.^60,61^^1^H NMR (500 MHz, CDCl_3_): *δ* 12.98 (br s, 1H, OH), 6.20 (d, *J* = 0.9 Hz, 1H, H-3), 2.90 (ddd, *J* = 7.5, 7.1, 1.0 Hz, 2H, H-8), 2.67 (ddd, *J* = 7.5, 7.1, 1.0 Hz, 2H, H-6), 2.13 (d, *J* = 0.9 Hz, 3H, CH_3_), 2.11 (m, 2H, CH_2_-5). LC-MS (ESI), [*m*/*z*]: 150.1 [M + H]^+^.

#### 2-Chloro-4-methyl-6,7-dihydro-5*H*-cyclopenta[*b*]pyridine (11)^61^

A mixture of phenylphosphonic dichloride (1.0 mL, 3.36 mmol) and compound 10 (500 mg, 3.36 mmol) was heated at 160 °C in a microwave vial for 21 h. The reaction mixture was then allowed to cool to room temperature and H_2_O was added dropwise on an ice bath. The reaction mixture was then diluted with H_2_O (50 mL), neutralized by the careful addition of K_2_CO_3_ (1.0 g) and gently extracted with CHCl_3_. The combined organic phases were washed with sodium chloride aq. sol., dried over sodium sulfate and concentrated *in vacuo* to afford the crude product as a black oil. Flash chromatography (CHCl_3_ → CHCl_3 _: MeOH 9 : 1) afforded compound 2 (455 mg, 81%) as a yellow oil that solidified upon standing in the fridge. The spectroscopic data was in agreement with that reported in the literature.^61^^1^H NMR (500 MHz, CDCl_3_): *δ* 6.90 (s, 1H, H-3), 2.98 (dd, *J* = 7.5, 7.5 Hz, 2H, H-8), 2.82 (dd, *J* = 7.5, 7.5 Hz, 2H, H-6), 2.122 (s, 3H, CH_3_), 2.11 (m, 2H, CH_2_-5).

#### 2-Chloro-4-methyl-6,7-dihydro-5*H*-cyclopenta[*b*]pyridin-7-yl acetate (12)^61^

To a solution of compound 11 (460 mg, 2.75 mmol) in glacial acetic acid (3.8 mL) was added an aqueous solution of hydrogen peroxide (35% w/w, 1.4 mL, 11.55 mmol) and the resultant mixture was heated at 80 °C for 22 h. The reaction mixture was then allowed to cool to room temperature, the solvent was removed under reduce pressure and the crude was diluted with H_2_O. The slightly acidic solution was neutralized by addition of potassium carbonate and then extracted with CHCl_3_. The combined organic phases were washed with sodium chloride aq. sol., dried over sodium sulfate and concentrated *in vacuo* to afford the corresponding pyridine *N*-oxide. To this compound was added dry and distilled acetic anhydride (3.9 mL) and the resultant suspension was stirred at room temperature for 1 h and then heated at 100 °C for 4 h. The reaction mixture was then allowed to cool to room temperature and was concentrated *in vacuo*. Flash chromatography using hexane : Et_2_O (1 : 1) as eluent afforded compound 3 (615 mg, 48%) as a yellow oil. The spectroscopic data was in agreement with that reported in the literature.^60,61^^1^H NMR (400 MHz, CDCl_3_): *δ* 9.8 (br s, 1H, OH), 7.24 (s, 1H, H-3), 3.22 (dd, *J* = 7.8, 7.8 Hz, 2H, H-8), 2.92 (dd, *J* = 7.8, 7.8 Hz, 2H, H-6), 2.22 (d, *J* = 0.9 Hz, 3H, CH_3_), 2.20 (m, 2H, CH_2_-5), 2.05 (s, 3H).

#### 2-Chloro-4-methyl-6,7-dihydro-5*H*-cyclopenta[*b*]pyridin-7-ol (13)^62^

To a solution of 12 98.9 (300 mg, 1.3 mmol) in THF : H_2_O (3 : 1, 3 mL) was added lithium hydroxide monohydrate (220 mg, 5.2 mmol) and the resultant black solution was stirred at room temperature for 21 h. The reaction mixture was then diluted with HO and extracted with DCM. The combined organic phases were washed with sodium chloride aq. sol., dried over sodium sulfate and concentrated *in vacuo* to afford the corresponding crude product. Flash chromatography with hexane : EtOAc (1 : 1) as eluent afforded compound 13 (189 mg, 79%) as a white crystalline solid. The spectroscopic data was in agreement with that reported in the literature.^62^^1^H NMR (400 MHz, CDCl_3_): *δ* 7.04 (s, 1H, H-3), 5.15 (m, 1H, H-8), 2.90–3.02 (m, 1H), 2.75–2.66 (m, 1H), 2.60–2.49 (m, 1H) 2.22 (s, 3H, CH_3_), 2.09–1.99 (m, 1H).

#### (*S*)-2-Chloro-4-methyl-6,7-dihydro-5*H*-cyclopenta[*b*]pyridin-7-ol (12)^62^ and (*R*)-2-chloro-4-methyl-6,7-dihydro-5*H*-cyclopenta[*s*]pyridin-7-yl acetate (13)^62^

To a mixture of racemic compound 13 (185 mg, 1.0 mmol) in vinyl acetate (2 mL), Novozyme 435 (185 mg) was added. The resulting suspension was vigorously stirred at r. t. for 4 h. Then, the enzyme was separated *via* filtration and washed with CH_2_Cl_2_. The solvent was then removed under reduced pressure and the resulting mixture was separated *via* flash chromatography (hexane : EtOAC 1 : 1). Compound (*R*)-13 (100 mg, 44%) was obtained as a yellow oil and (*S*)-12 (75 mg, 41%) as a white crystalline solid. See NMR data given above.

#### (*R*)-2-Ethynyl-6,7-dihydro-5*H*-cyclopenta[*b*]pyridin-7-yl acetate (14)^63^

To an oven-dried microwave vial and under nitrogen, CsCO_3_ (358 mg, 1.1 mmol), XPhos (31 mg, 0.065 mmol) and Pd(PPh_3_)_2_Cl_2_ (6 mg, 0.023 mmol) were added. The vial was sealed and filled with N_2_. Compound 12 (100 mg, 0.44 mmol) dissolved in deoxygenated dry CH_3_CN (11 mL) was added. N_2_ was bubbled through this solution for 10 min. Trisopropylsilylacetylene (250 μL, 1.1 mmol) was added dropwise *via* a syringe. The resulting reaction mixture was heated at 120 °C for 20 min using microwave irradiation. The vial was allowed to cool to room temperature, the mixture was dissolved with CH_2_Cl_2_ and washed twice with brine. The organic phase was dried with Na_2_SO_4_ and the solvent was removed under reduce pressure to yield a brown oil. The resulting dark brown crude was filtered through a silica plug, using hexane : EtOAc (1 : 1) as eluent. Compound 14 was obtained as a brown oil, which was used in the next step without further purification. A solution of TBAF in THF (1.0 M, 0.5 mL, 0.5 mmol, 1.1 eq.) was added to a solution of the TIPs-protected alkyne in dry THF (4.4 mL). The reaction mixture was stirred at 0 °C for 2 h under N_2_ atmosphere. After being warmed to room temperature, the reaction mixture was diluted in Et_2_O and washed twice with brine. The organic phase was dried over Na_2_SO_4_ and the solution was subsequently concentrated under reduced pressure. The crude mixture was purified by column chromatography on silica gel with hexane : EtOAc (7 : 3) as eluent to give 14 ^63^ as a brown solid (80 mg, 84% (over two steps)). ^1^H NMR (500 MHz, CDCl_3_) *δ* 7.24 (s, 1H, H3), 6.03 (dd, *J* = 7.5, 4.2 Hz, 1H, H7), 3.10 (s, 1H, H12), 3.0–2.96 (m, 1H, H6), 2.86–2.77 (m, 1H, H6), 2.67–2.57 (m, 1H, H5), 2.28 (s, 3H, -Me4,), 2.10 (s,3H, -Me9), 2.08–2.03 (m, 1H, H5). ^13^C NMR (126 MHz, CDCl_3_) *δ* 170.9 (C8), 160.5 (C7a), 144.5 (C4), 141.6 (C2), 137.7 (C4a), 128.3 (C3), 83.1 (C7), 77.5 (C10), 76.5 (C12), 30.5 (C6), 26.7 (C5), 21.4 (-Me9), 18.6 (Me-4). LC-MS (ESI), [*m*/*z*]: 216.3 [M + H]^+^.

### Synthesis of 1,2-bis(((6*R*,8*R*)-7,7-dimethyl-5,6,7,8-tetrahydro-6,8-methanoisoquinolin-3-yl)ethynyl)benzene (3)

#### (6*R*,8*R*)-(−)-7,7-Dimethyl-5,6,7,8-tetrahydro-6,8-methano-isoquinolin-3(4*H*)-one (15)^64^

A solution of (−)-myrtenal (2.0 g, 13.3 mmol), Kröhnke salt 1-(2-amino-2-oxoethyl)pyridin-1-ium chloride (2.6 g, 15 mmol), and freshly distilled piperidine (1.5 mL, 15 mmol) in dry MeOH (50 mL) was heated at reflux for 3 h under Ar atmosphere. After cooling to rt, the solvent was removed under reduced pressure. The red-brown crude was dissolved in dry formamide (15 mL) and glacial AcOH (3 mL) was added. This mixture was heated at 200 °C for 1 h. The mixture was cooled to rt, quenched with H_2_O (50 mL), and the product was extracted with CH_2_Cl_2_. The aqueous layer was basified with 1 M NaOH. The organic phase was separated, and the aqueous layer was extracted with CH_2_Cl_2_ (4 × 50 mL). The combined organic layers were washed with brine and dried (Na_2_SO_4_), and the solvent was removed under vacuum to give a brown oil, which was purified by flash chromatography on silica gel with a mixture of CH_2_Cl_2_/EtOAc (1 : 1) for the elution of secondary products, and with EtOAc/MeOH (9 : 1) to give 15 (500 mg, 20%) as a brown foam, which was used in the next step without further purification. The analytical data was in agreement with that reported.^64^^1^H NMR (400 MHz, CDCl_3_) *δ* 6.88 (s, 1H), 6.39 (s, 1H), 2.89 (d, *J* = 1.5 Hz, 2H), 2.68–2.54 (m, 2H), 2.17 (tt, *J* = 5.8, 2.9 Hz, 1H), 1.34 (s, 1H), 1.17 (d, *J* = 9.6 Hz, 1H), 0.67 (s, 3H). ^13^C NMR (101 MHz, CDCl_3_) *δ* 165.3, 152.4, 127.6, 126.4, 118.3, 43.9, 40.2, 39.5, 32.9, 32.4, 26.00, 21.6.

#### (6*R*,8*S*)-(−)-5,6,7,8-Tetrahydro-7,7-dimethyl-6,8-methano-isoquinolin-3-yl trifluoromethanesulfonate (16)^65,66^

Freshly destilled Et_3_N (2.5 mL, 18 mmol) was added to a solution of 15 (2.80 g, 15 mmol) in dry CH_2_Cl_2_ (55 mL) and the solution was cooled to −45 °C. Trifluoromethanesulfonic acid anhydride (3.0 mL, 18 mmol) was added dropwise *via* a syringe pump over a period of 1 h. After stirring at −45 °C for 1 h, the solution was allowed to slowly warm to rt. The reaction mixture was stirred overnight and then quenched by the addition of H_2_O and an aqueous NaHCO_3_ solution. The mixture was extracted with CH_2_Cl_2_ and the combined organic phases were washed with brine and dried with Na_2_SO_4_. The solvent was removed under reduced pressure, and the residue was purified by flash chromatography (pentane/EtOAc 9 : 1) to give compound 16 (1160 mg, 24%) as a yellow oil along, with recovered starting material 15 (1040 mg). ^1^H NMR (400 MHz, CDCl_3_) *δ* 7.88 (s, 1H, H-4), 6.95 (s, 1H, H-1), 3.03 (d, *J* = 3.0 Hz, 2H, H-5), 2.88 (t, *J* = 5.5 Hz, 1H, H-8), 2.72 (dt, *J* = 9.9, 5.7 Hz, 1H, H-9), 2.31 (tt, *J* = 5.7, 2.9 Hz, 1H, H-6), 1.41 (s, 3H, CH_3_), 1.19 (d, *J* = 9.9 Hz, 1H, H9), 0.62 (s, 3H, CH_3_). ^13^C NMR (101 MHz, CDCl_3_) *δ* 154.6 (C, C-3), 150.7 (C, C-8a), 144.0 (CH, C-1), 143.7 (C, C-4a), 123.5 (C, OTf), 120.3 (C, OTf), 117.2 (C, OTf), 114.3 (CH, C-4), 114.0 (C, OTf), 44.1 (CH, C-8), 39.6 (CH, C-6), 39.3 (C, C-7), 33.2 (CH_2_, C-5), 31.6 (CH_2_, C-9), 25.9 (CH_3_), 21.4 (CH_3_). ^19^F NMR (376 MHz, CDCl_3_) *δ* −73.36. HRMS (MALDI TOF) calcd for C_13_H_15_F_3_NO_3_S 322.0724 [M + H]^+^, found *m*/*z* 322 0716. [*α*]^20^_D_ −36.5 (c 1.27, CDCl_3_).

#### 1,2-Bis((trimethylsilyl)ethynyl)benzene^67^

To a microwave vial was added CuI (60 mg, 0.31 mmol) and Pd(PPh_3_)_2_Cl_2_ (120 mg, 0.17 mmol). Then, the vial was sealed and deoxygenated. Et_2_NH (12 mL; purified by redistillation, 99.5%) was added to generate a pale red solution. N_2_ was bubbled through this solution for 15 min 1,2-diiodobenzene (400 μL, 3.03 mmol) was added to the flask, and N_2_ was bubbled into the solution for another 5 min. Ethynyltrimethylsilane (1.3 mL, 9 mmol) was added dropwise *via* syringe. The resulting yellow reaction mixture was bubbled with N_2_ for 2 min and heated using microwave irradiation for 15 min (110 °C, 30 s of pre-stirring, high absorption level). After that, the vial was allowed to cool to r.t., the solvent was removed under reduce pressure, the crude was dissolved with hexane and washed trice with saturated aqueous NH_4_Cl solution. The aqueous phase was extracted twice with hexane. The combined organic phases were dried with Na_2_SO_4_ and the solvent was removed under reduce pressure to yield a brown oil which was purified by flash chromatography with hexane as eluent. 1,2-Diethynylbenzene (800 mg, 95%) was obtained as a pale yellow oil which solidified upon standing in the freezer. The spectroscopic data was in agreement with that previously reported.^67^^1^H NMR (400 MHz, CDCl_3_) *δ* (ppm) 7.45 (dd, *J* = 5.8, 3.3 Hz, 2H), 7.23 (dd, *J* = 5.8, 3.3 Hz, 2H), 0.27 (s, 18H, TMS).

#### 1,2-Diethynylbenzene^67^

A solution of 1,2-bis((trimethylsilyl)ethynyl)benzene (760 mg, 2.8 mmol) in THF/MeOH (v/v = 1 : 1, 30 mL) was treated with K_2_CO_3_ (800 mg, 5.8 mmol) at r.t. for 2.5 h. Following the removal of organic solvent, H_2_O was added and the aqueous solution was extracted with CH_2_Cl_2_ trice. The CH_2_Cl_2_ solution was dried over Na_2_SO_4_. After solvent evaporation, the residue was purified by column chromatography on silica gel with hexane as eluent to give 1,2-diethynylbenzene (270 g, 77% yield) as colorless oil, which solidified upon standing in the freezer. The spectroscopic data was in agreement with that previously reported.^67^^1^H NMR (400 MHz, CDCl_3_) *δ* (ppm) *δ* 7.52 (dd, *J* = 5.7, 3.4 Hz, 1H), 7.31 (dd, *J* = 5.7, 3.4 Hz, 1H), 3.34 (s, 1H).

### Synthesis of 1,2-bis-[((5a*S*,9a*R*)-9a-phenyl-5a,6,7,8,9,9a-hexahydrobenzofuro[3,2-*b*]pyridin-2-yl)-ethinyl]benzene (4)

#### 2-Iodocyclohex-2-en-1-one (17)^68^

K_2_CO_3_ (9.25 g, 66.9 mmol, 1.20 eq.), I_2_ (21.2 g, 83.7 mmol, 1.50 eq.) and DMAP (1.36 g, 11.2 mmol, 0.20 eq.) were stirred in a solution of THF/H_2_O 1 : 1 (250 mL) for 5 min. The solution was cooled to 0 °C. The cooling bath was removed and cyclohex-2-enone (5.40 mL, 55.8 mmol, 1.00 eq.) was added, by fast addition of the first 2 mL and the remaining volume added dropwise. The mixture was stirred for 1.5 h, complete conversion was confirmed by NMR of the reactions mixture. The mixture was cooled to 0 °C and was neutralized with aqueous 2 M HCl solution, extracted with Et_2_O (4 times of the THF volume), and the organic layer was 5 times carefully (!) washed with 1 M HCl to remove DMAP (causes deiodination). Washing with brine and finally with Na_2_S_2_O_3_ solution removes Iodine and the organic layer was dried over Na_2_SO_4_. Removal of the solvent gave a pure product; if necessary purification can be performed by column chromatography (silica gel ∼3 g mmol^−1^, *n*-hexane/EtOAc (10 : 90) to (30 : 70) [10CV], then (30 : 70) [5 CV]) to yield a yellow solid (10.6 g, 47.6 mmol, 85%). The product is volatile and should not be dried over vacuum or be exposed to heat. It was stored at −20 °C and under N_2_ atmosphere. On long time storage, C-2 homo-coupling was observed. The analytical data was in agreement with that reported.^68^^1^H NMR (400 MHz, CDCl_3_): *δ* 7.76 (q, ^3^*J*_HH_ = 4.3 Hz, 1H, H-3), 2.72–2.60 (m, 2H, H-6), 2.49–2.38 (m, 2H, H-4), 2.15–1.99 (m, 2H, H-5). ^13^C NMR (100 MHz, CDCl_3_): *δ* 192.3 (C-1), 159.6 (C-3), 104.0 (C-2), 37.4 (C-6), 30.1 (C-4), 23.0 (C-5).

#### Synthesis of 2-phenyl-cyclohexa-2-en-1-one (18)^69^

In a round-bottom-flask 10 w/w% Pd/C Type487 (5.45 g, 24.6 mmol, 0.10 eq. Pd) and Na_2_CO_3_ (5.21 g, 49.1 mmol, 2.00 eq.) were evacuated for 2 h, then a solution of degassed stabilized dimethoxyethane (DME) and degassed H_2_O (80 mL 1 : 1 v/v) was added. PhB(OH)_2_ (4.49 g, 36.8 mmol, 1.50 eq.) and 2-iodocyclohex-2-en-1-one (17) (5.45 g, 24.6 mmol, 1.00 eq.) were added, and the mixture was stirred for 18 h at room temperature. It was filtered twice by vacuum filtration through a P2 frit, and the resulting dark red solution was extracted with Petroleum ether (4 × 100 mL). The combined organic layer was washed with brine (3 × 100 mL), dried over MgSO_4_, and the solvent was removed in vacuum. Flash column chromatography (50 g silica /3 g crude, *n*-hexane/EtOAc 10 : 90 to 20 : 80 [5CV], then 20 : 80 [5CV]) gave the desired product as a white solid (3.17 g, 18.4 mmol, 75%). The analytical data were in agreement with that reported in the literature.^69^^1^H NMR (400 MHz, CDCl_3_): *δ* 7.38–7.27 (m, 5H, 8-H, 9-H, 10-H, 11-H, 12-H), 7.03 (t, ^3^*J*_HH_ = 4.3 Hz, 1H, 3-H), 2.63–2.58 (m, 2H, 5-H), 2.54 (td, ^3^*J*_HH_ = 6.0, 4.3 Hz, 2H, 4-H), 2.12 (p, ^3^*J*_HH_ = 6.2 Hz, 2H, 5-H). ^13^C NMR (101 MHz, CDCl_3_): *δ* 198.1 (C-1), 148.1 (C-3), 140.6 (C-7), 136.7 (C-2), 128.8 and 128.1 (C-8,9,11,12), 127.7 (C-10), 39.2 (C-6), 26.8 (C-4), 23.1 (C-5). LC-MS (ESI), [*m*/*z*]: 173.3 [M + H]^+^

#### *rac*-2-Phenylcyclohex-2-ene-1-ol (19)^70^

Following a literature procedure,^71,72^ a round-bottom flask was charged with 2-phenylcyclohex-2-ene-1-one (18) (4.24 g, 24.6 mmol, 1.00 eq.), CeCl_3_·7 H_2_O (2.75 g, 7.38 mmol, 0.30 eq.) and MeOH (4 mL mmol^−1^). When the solids have been dissolved, the solution was cooled to 0 °C. At the first sign of precipitation NaBH_4_ (1.60 g, 42.3 mmol, 1.72 eq.) was added in small portions. The reaction progress was monitored by LC-MS. After 2 h, the reaction mixture was deactivated with saturated aqueous NH_4_Cl solution (100 mL), the mixture was extracted with CH_2_Cl_2_ (3 × 100 mL), the combined organic layer was washed with brine (3 × 100 mL) and dried over MgSO_4_. Removal of the solvent afforded the desired product as a colourless oil, which crystallized upon cooling (3.992 g, 22.9 mmol, 93%). Analytical HPLC using a Lux® 5 μm Amylose-1 column (250 × 4.6 mm), H_2_O/CH_3_CN (60 : 40 to 70 : 30 in 30 min), 1 ml min^−1^, retention time of (*S*)-19 = 18.6 min, and of (*R*)-19 = 9.3 min. The enantiomers can be separated by preparative HPLC on a Lux® 5 μm Amylose-1 column (50 × 21.2 mm), with H_2_O/CH_3_CN (20 : 80) eluent mixture at 20 ml min^−1^ flow rate (loading: ∼200 mg per run in 2 mL CH_3_CN). The spectroscopic data of the enantiomers was in agreement with that previously reported.^70^^1^H NMR (400 MHz, CD_2_Cl_2_): *δ* 7.46 (d, ^3^*J*_HH_ = 7.4 Hz, 2H, 8-H, 12-H), 7.33 (t, ^3^*J*_HH_ = 7.6 Hz, 2H, 9-H, 11-H), 7.27–7.22 (m, 1H, 10-H), 6.16 (t, ^3^*J*_HH_ = 4.1 Hz, 1H, 3-H), 4.68 (br. q, ^3^*J*_HH_ = 4.6 Hz, 1H, 1-H), 2.32–2.10 (m, 2H, 4-H), 1.96–1.63 (m, 4H, 5-H, 6-H), 1.61 (d, ^3^*J*_HH_ = 5.92 Hz, 1H, O–H). ^13^C NMR (101 MHz, CD_2_Cl_2_): *δ* 141.1 (C-7), 139.7 (C-2), 129.2(C-3), 129.0 (C-8, C-12), 127.5 (C-10), 126.5 (C9, C-11), 65.9 (C-1), 32.4 (C-6), 26.6 (C-4), 17.9 (C-5). LC-MS (ESI), [*m*/*z*]: 157.2 [M + H-H_2_O]^+^.

#### (*S*)/(*R*)-2-Phenylcyclohex-2-ene-1-ol (*S*-20)

Following the literature,^73^ an oven dried round-bottom flask was charged with (*R*)-(–)-2-methyl-CBS-oxazaborolodine (1 m in toluene, 7.5 mL, 7.5 mmol, 0.29 eq.) and BH_3_·SMe_2_ (94 w/w%, 3.18 mL, 0.801 g mL^−1^, 31.5 mmol, 1.24 eq.), and was cooled to 0 °C. 2-Phenylcyclohex-2-ene-1-one (4.39 g, 25.5 mmol, 1.00 eq.) dissolved in 75 mL toluene was added *via* a syringe pump within 2 h, and the mixture was stirred for 1 h at room temperature. The mixture was neutralized with 1 M H_2_SO_4_ (∼90 mmol H^+^). The aqueous phase was extracted with Et_2_O (3 × 50 mL), and the combined toluene and Et_2_O layer was washed with brine (3 × 50 mL), and was dried with MgSO_4_. The solvent was removed *in vacuo* to yield the crude product (4.40 g, 99%, 84 ee-% (based on the separated amounts of enantiomers)). The crude product was purified using preparative HPLC with a Lux® 5 μm Amylose-1 column (250 × 21.2 mm), with H_2_O/CH_3_CN (20 : 80) and 20 ml min^−1^ flow rate, with 200 mg per run dissolved in 2 mL CH_3_CN for loading. Analytical HPLC using a Lux® 5 μm Amylose-1 column (250 × 4.6 mm), H_2_O/CH_3_CN (60 : 40 to 70 : 30 in 30 min), 1 ml min^−1^, retention time (*S*)-20 = 16.8 min, (*R*)-20 = 9.3 min ^1^H NMR (400 MHz, CDCl_3_): *δ* 7.47 (d, ^3^*J*_HH_ = 7.7 Hz, 2H, 8-H, 12-H), 7.34 (t, ^3^*J*_HH_ = 7.5 Hz, 2H, 9-H, 11-H), 7.27 (d, ^3^*J*_HH_ = 7.1 Hz, 1H, 10-H), 6.17 (t, ^3^*J*_HH_ = 4.0 Hz, 1H, 3-H), 4.72 (q, ^3^*J*_HH_ = 4.6 Hz, 1H, 1-H), 2.34–2.11 (m, 2H, 4-H), 2.03–1.65 (m, 4H, 5-H, 6-H), 1.64–1.59 (m, 1H, 13-H). ^13^C NMR (101 MHz, CDCl_3_): *δ* 140.3 (C-7), 139.2 (C-2), 128.8 (C-3), 128.7 (C-9, C-11), 127.2 (C-10), 126.1 (C-8, C-12), 65.6 (C-1), 31.7 (C-6), 26.2 (C-4), 17.5 (C-5). HRMS (MALDI TOF) calcd for C_11_H_11_ClINO 335.9652 [M + H]^+^, found *m*/*z* 335.9648.

#### (*S*)/(*R*)-6-Chloro-2-iodo-3-((2-phenylcyclohex-2-ene-1-oxy)pyridine (21)

A round-bottom flask was charged with *rac*-2-phenyl-cyclohex-2-enol (20) (1.43 g, 8.21 mmol, 1.00 eq.), 6-chloro-2-iodopyridin-3-ol (98 w/w%, 2.18 g, 8.37 mmol, 1.03 eq.) and PPh_3_ (2.26 g, 8.62 mmol, 1.05 eq.) and toluene (15 mL mmol^−1^). The mixture was cooled to 0 °C and diisopropylazodicarboxylate (1.75 mL, 94 w/w%, 1.027 g mL^−1^, 8.37 mmol, 1.03 eq.) was added dropwise. The reaction mixture was stirred in an ice bath 3 d reaching room temperature. Completion of the reaction was confirmed by LC-MS. Then, 1 M aqueous NaOH (20 mL) was added and the mixture was extracted with CHCl_3_ (3 × 50 mL). The combined organic layer was dried over MgSO_4_, and the solvent was removed in vacuum. Flash column chromatography (silica, ∼50 g mmol^−1^ starting material, *n*-hexane/EtOAc 95 : 5 v/v [3CV], then 95 : 5 to 93 : 7 over [5CV], then 93 : 7 [5CV]) gave pure 21. *Rac*-21 was obtained as a colourless oil (3.3 g, 8.02 mmol, 98%). When enantiopure (*R*)-2-phenyl-cyclohex-2-enol (20) was used as starting material, (*S*)-6-chloro-2-iodo-3-((2-phenylcyclohex-2-ene-1-oxy)pyridine was observed in a mixture with its *R*-enantiomer. Thus ee is lost under the reaction conditions. Separation of the enantiomers was performed by preparative HPLC using a Lux® 5 μm Amylose-1 column (250 × 21.2 mm). Optimized conditions for the separation of (*S*)-21: H_2_O/CH_3_CN 30 : 70, 22 ml min^−1^ flow; loading: ∼100 mg per run sample in 0.5 mL CH_3_CN. Optimized conditions for (*R*)-21 (major product from (*R*)-Me-CBS): H_2_O/CH_3_CN 30 : 70, 22 mL min^−1^ flow rate; loading: ∼200 mg per run sample in 1 mL CH_3_CN. Analytical HPLC using a Lux® 5 μm Amylose-1 column (250 × 4.6 mm), with H_2_O/CH_3_CN (60 : 40 to 70 : 30 in 30 min), eluent at 1 ml min^−1^, eluting (*S*)-21 at 18.3 min, and (*R*)-21 at 21.6 min retention time. ^1^H NMR (400 MHz, CDCl_3_): *δ* 7.34–7.20 (m, 5H, 8′-H, 9′-H, 10′-H, 11′-H, 12′-H), 7.13 (d, ^3^*J*_HH_ = 8.4 Hz, 1H, 5-H), 7.00 (d, ^3^*J*_HH_ = 8.5 Hz, 1H, 4-H), 6.34 (dd, ^3^*J*_HH_ = 5.1, 2.9 Hz, 1H, 3′-H), 5.13 (d, ^3^*J*_HH_ = 3.7 Hz, 1H, 1′-H), 2.52–2.36 (m, 1H, 4′-Ha), 2.33–2.17 (m, 1H, 4′-Hb), 2.16–2.07 (m, 1H, 6′-Hb), 2.07–1.92 (m, 1H, 5′-Ha), 1.81 (dt, *J* = 13.4, 3.4 Hz, 1H, 6′-Ha), 1.77–1.69 (m, 1H, 5′-Hb). ^13^C NMR (101 MHz, CDCl_3_): *δ* 153.8 (C′–3), 141.5 (C′–6), 141.0 (C′–7′), 135.5 (C′–2′), 132.3 (C′–3′), 128.6 (C′–9′, C′–11′), 127.3 (C′–10′), 126.2 (C′–8′, C-12′), 123.6 (C′–5), 122.3 (C′–4), 111.4 (C′–2), 75.1 (C′–1′), 27.7 (C′–6′), 25.9 (C′–4′), 17.1 (C′–5′). LC-MS (ESI), [*m*/*z*]: 414.1 [M(^37^Cl) + H]^+^, 412.1 [M(^35^Cl) + H]^+^, 258.1 [M(^37^Cl)-C_12_H_13_]^+^, 256.1 [M(^35^Cl)-C_12_H_13_]^+^.

#### 2-Chloro-9a-phenyl-5a,6,7,8,9,9a-hexahydrobenzofuro [3,2-*b*]pyridine (22)

An oven dried flask was charged with a pure enantiomer of 6-chloro-2-iodo-3-((2-phenylcyclohex-2-ene-1-oxy)pyridine (21) (2.26 g, 5.49 mmol, 1.00 eq.), [Pd(PPh_3_)_2_Cl_2_] (771 mg, 1.10 mmol, 0.20 eq.) and Ag_2_CO_3_ (151 mg, 0.55 mmol, 0.10 eq.), and was evacuated and filled with N_2_ trice. Toluene (110 mL) and NEt_3_ (2.30 mmol, 16.5 mmol, 3.00 eq.) were added, and the mixture was stirred at 110 °C for 48 h the reaction progress being monitored by LC-MS. Toluene was removed in vacuum in the presence of silica gel, and the crude mixture was purified by column chromatography (silica, ∼10 g mmol^−1^ starting material, 75 mL min^−1^, *n*-hexane/EtOAc gradient 97 : 3 to 90 : 10 v/v [10CV]) yielding (5a*S*,9a*R*)-mix of regioisomers of 21 (940 mg, 3.31 mmol, 60%) (5a*R*,9a*S*)-mix of regioisomers of 21 (740 mg, 2.61 mmol, 57%). All fractions that showed absorption at 300 nm were collected and used for the next step. (Note: finish the reaction after 4–8 h to prevent diasteroisomerisation by double bond migration. Desired product fractions can be identified by the phenyl UV absorption at 300 nm.) The mixed fractions containing 21 were dissolved in MeOH (1 mL/10 mg crude material) and Rh on carbon (5 mg/10 mg crude material, 5% metal loading) was added. 5 bar H_2_ was applied and the mixture was stirred until completion of the reaction was indicated by LC-MS. The product was purified by column chromatography (silica, ∼10 g mmol^−1^ starting material, 60 mL min^−1^, *n*-hexane/EtOAc 90 : 10 v/v [1CV] then gradient 90 : 10 to 70 : 30 v/v [10CV]) yielding (5a*S*,9a*R*)-2-chloro-9a-phenyl-5a,6,7,8,9,9a-hexahydrobenzofuro[3,2-*b*]pyridine, 660 mg, 2.31 mmol, 70%) or (5a*R*,9a*S*)-2-chloro-9a-phenyl-5a,6,7,8,9,9a-hexahydrobenzofuro[3,2-*b*]pyridine, 360 mg, 1.26 mmol, (48%). (Note: the starting material and the product are difficult to separate, and so complete conversion is necessary. Longer reaction times cause hydration of the Ph ring to Cy which is easier to separate.) Analytical HPLC (Lux® 5 μm Amylose-1 column (250 × 4.6 mm), H_2_O/CH_3_CN (50 : 50) with 1 ml min^−1^ flow rate eluting (5a*S*,9a*R*)-22 at 10.82 min, and (5a*R*,9a*S*)-22 at 11.46 min retention time. (5a*R*,9a*S*)-22^1^H NMR (500 MHz, CDCl_3_): *δ* 7.42–7.38 (m, 2H, 2′-H, 6′-H), 7.33 (t, *J* = 7.6 Hz, 2H, 3′-H, 5′-H), 7.25–7.21 (tt, *J* = 7.3, 1.3 Hz, 1H, 4′-H), 7.06 (AB-spin system of higher order, 2H, 3-H, 4-H), 5.10 (t, *J* = 4.8 Hz, 1H, 5a-H), 2.26–2.19 (ddd, *J* = 14.0, 8.5, 3.6 Hz, 1H, 9-H_a_), 2.17–2.10 (m, 1H, 9-H_b_), 2.00–1.91 (m, 2H, 6-H), 1.68–1.55 (m, 3H, 7-H_a_, 8-H_a_, 8-H_b_), 1.49–1.40 (m, 1H 7-H_b_). ^13^C NMR (126 MHz, CDCl_3_): *δ* 157.7 (C-10), 152.3 (C-5), 143.6 (C-1′), 142.6 (C-2), 128.6 (C-3′, C-5′), 127.2 (C-2′, C-6′), 126.9 (C-4′), 122.8 and 119.5 (C-3, C-4), 89.5 (C-5a), 51.6 (C-9a), 33.3 (C-9), 27.0 (C-6), 20.9 (C-7), 19.3 (C-8).

#### (5a*S*,9a*R*)/(5a*R*,9a*S*)-2-(2-Tri(isopropyl)silylethyn-1-yl)-9a-phenyl-5a,6,7,8,9,9a-hexahydrobenzofuro[3,2-*b*]pyridine (23)

An oven-dried flask was charged with [PdCl_2_(CH_3_CN)_2_] (22.4 mg, 0.086 mmol, 0.05 eq.), XPhos (122.6 mg, 0.257 mmol, 0.15 eq.) and Cs_2_CO_3_ (1.117 g, 3.439 mmol, 2.00 eq.), and was then evacuated and flushed with N_2_ trice. 2-Chloro-9a-phenyl-5a,6,7,8,9,9a-hexahydrobenzofuro[3,2-*b*]pyridine (22) (490 mg, 1.71 mmol, 1.00 eq.) dissolved in dry acetonitrile was added. The mixture was stirred at 85 °C until completion of the reaction, as indicated by LC/MS (8 h). The mixture was filtered over Celite, and the solvent was removed in vacuum. Column chromatography (silica, ∼50 g mmol^−1^ starting material, 50 mL min^−1^, *n*-hexane/EtOAc 98 : 2 v/v [10CV]) gave the pure product ((5a*R*,9a*S*)-23 (590 mg, 1.37 mmol, 80%) Alternatively, (5a*S*,9a*R*)-23 was synthesized in (494 mg, 1.14 mmol, 67%). Analytical HPLC was performed on a Lux® 5 μm Amylose-1 column (250 × 4.6 mm), using H_2_O/CH_3_CN (30 : 70) as eluent with 1 ml min^−1^ flow rate, eluting the 5a*S*,9a*R*-23 at 11.29 min, and (5a*R*,9a*S*)-22 at 10.94 min retention times.

#### (5a*S*,9a*R*)-23

^1^H NMR (400 MHz, CDCl_3_): *δ* 7.42–7.39 (m, 2H, 2′-H, 6′-H), 7.34–7.24 (m, 3H, 3-H, 3′-H, 5′-H, 4′-H), 7.22 (t, *J* = 7.5 Hz, 1H, 4′-H), 6.99 (d, *J* = 8.3 Hz, 1H, 4-H), 5.06 (t, *J* = 4.9 Hz, 1H, 5a-H), 2.25–2.14 (m, 2H, 9-H), 2.03–1.86 (m, 1H, 6-H), 1.67–1.50 (m, 3H, 7-H_a_, 7-H_b_, 8-H_a_), 1.47–1.38 (m, 1H, 8-H_b_), 1.13 (d, *J* = 1.9 Hz, 21H, 4′′-12′′-H). ^13^C NMR (126 MHz, CDCl_3_): *δ* 157.5 (C-10), 152.7 (C-5), 144.2 (C-1′), 135.7 (C-2), 128.5 (C-3′, C-5′), 128.3 (C-3), 127.4 (C-2′, C-6′), 126.7 (C-4′), 116.3 (C-4), 107.1 (C-1′′), 89.2 (C-5a), 89.2 (C-2′′) 51.6 (C-9a), 33.4 (C-9), 27.2 (C-6), 21.0 (C-8), 19.5 (C-7), 18.9 (C-5′′, C-6′′, C-8′′, C-9′′, C-11′′, C-12′′), 11.5 (C-4′′, C-7′′, C-10′′). LC-MS (ESI), [*m*/*z*]: 432.4 [M + H]^+^.

#### (5a*R*,9a*S*)-23

^1^H NMR (400 MHz, CDCl_3_): *δ* 7.33–7.29 (m, 2H, 2′-H, 6′-H), 7.23 (tt, *J* = 7.7, 1.7 Hz, 2H, 3′-H, 5′-H), 7.20 (d, *J* = 8.2 Hz, 1H, 3-H), 7.13 (tt, *J* = 7.5, 1.2 Hz, 1H, 4′-H), 6.91 (d, *J* = 8.3 Hz, 1H, 4-H), 4.98 (t, *J* = 4.9 Hz, 1H, 5a-H), 2.18–2.05 (m, 2H, 9-H), 1.94–1.86 (m, 1H, 6-H_a_), 1.87–1.77 (m, 1H, 6-H_b_), 1.55–1.42 (m, 3H, 7-H_a_, 7-H_b_, 8-H_a_), 1.39–1.29 (m, 1H, 8-H_b_), 1.11–0.97 (m, 21H, 4′′to12′′-H). ^13^C NMR (126 MHz, CDCl_3_): *δ* 157.6 (C-10), 152.7 (C-5), 144.1 (C-1′), 135.6 (C-2), 128.5 (C-3′, C-5′), 128.3 (C-3), 127.4 (C-2′, C-6′), 126.8 (C-4′), 116.3 (C-4), 107.0 (C-1′′), 89.2 (5a), 89.2 (2′′), 51.6 (C-9a), 33.4 (C-9), 27.1 (C-6), 21.0 (C-8), 19.4 (C-7), 18.8 (C-5′′, C-6′′, C-8′′, C-9′′, C-11′′, C-12′′), 11.5 (C-4′′, C-7′′, C-10′′). ^15^N NMR (51 MHz, CDCl_3_): *δ* [-72 ppm (CH_3_NO_2_ at 0 ppm). LC-MS (ESI), [*m*/*z*]: 432.4 [M + H]^+^.

#### (5a*S*,9a*R*)-2-Ethynyl-9a-phenyl-5a,6,7,8,9,9a-hexahydrobenzofuro[3,2-*b*]pyridine (24)

An oven dried microwave vial was charged with 2-(2′′-triisopropylsilylethyn-1′′-yl)-9a-phenyl-5a,6,7,8,9,9a-hexahydrobenzofuro[3,2-*b*]pyridine (23) (765 mg, 1.77 mmol, 1.00 eq.), evacuated and flushed with N_2_. Dry THF (4 mL) was added and the mixture was cooled to 0 °C. Then NBu_4_F in THF (1 m, 1.95 mL, 1.95 mmol, 1.10 eq.) was added dropwise, and the mixture was stirred for 2 h. Et_2_O (50 mL) was added and the mixture was extracted with brine (3 × 30 mL). Column chromatography (silica, ∼45 g mmol^−1^ starting material, *n*-hexane/EtOAc 95 : 5 v/v [10CV]) gave pure (5a*S*,9a*R*)-24 (488 mg, 1.69 mmol, 95%), or the corresponding procedure (5a*R*,9a*S*)-24 (330 mg, 1.20 mmol, 88%). Analytical HPLC was performed on a Lux® 5 μm Amylose-1 column (250 × 4.6 mm) using H_2_O/CH_3_CN (60 : 40) eluent at 1 ml min^−1^ flow rate, giving (5a*S*,9a*R*)-24 at 20.40 min, and (5a*R*,9a*S*)-24 at 21.19 min retention time. (5a*S*,9a*R*)-24. ^1^H NMR (500 MHz, CDCl_3_): *δ* 7.42–7.37 (m, 2H, 2′-H, 6′-H), 7.33–7.29 (m, 2H, 3′-H, 5′-H), 7.27 (d, *J* = 12.4 Hz, 1H, 3-H), 7.21 (tt, *J* = 7.3, 1.1 Hz, 1H, 4′-H), 7.02 (d, *J* = 8.3 Hz, 1H, 4-H), 5.11 (t, *J* = 5.0 Hz, 1H, 5a-H), 3.06 (s, 1H, 2′′-H), 2.21 (t, J = 6.0 Hz, 2H, 9-H), 2.04–1.94 (m, 1H, 6-H_a_), 1.94–1.85 (m, 1H, 6-H_b_), 1.66–1.49 (m, 3H, 7-H_a_, 7-H_b_, 8-H_a_), 1.47–1.37 (m, 1H, 8-H_b_). ^13^C NMR (126 MHz, CDCl_3_): *δ* 158.0 (C-10), 153.1 (C-5), 143.8 (C-1′), 134.1 (C-2), 128.4 (C-3′, C-5′), 127.5 (C-3), 127.2 (C-2′, C-6′), 126.7 (C-4′), 116.4 (C-4), 89.3 (C-5a), 83.4 (C-1′′), 75.5 (C-2′′), 51.5 (C-9a), 33.1 (C-9), 27.0 (C-6), 20.9 (C-8), 19.3 (C-7). LC-MS (ESI), [*m*/*z*]: 276.3 [M + H]^+^.

### Synthesis of 1,2-bis(((5a*S*,9a*S*)-5a,6,7,8,9,9a-hexahydrobenzofuro[3,2-*b*]pyridin-2-yl)ethynyl)benzene (5)

#### 6-Chloro-3-(cyclohex-2-en-1-yloxy)-2-iodopyridine (25)

In a two necked round bottom flask, 6-chloro-2-iodopyridin-3-ol (500 mg, 1.95 mmol) was added to a suspension of K_2_CO_3_ (810 mg, 5.9 mmol) in dry and deoxygenated DMF (6.5 mL). 3-Bromocyclohex-1-ene (270 μL, 2.35 mmol) was added dropwise *via* a syringe and the mixture was stirred under N_2_ atmosphere at r.t.; after 17 h, distilled H_2_O (9 mL) was slowly added and the solution was extracted four times with *n*-hexane. The combined organic phase was washed with distilled H_2_O trice, KOH (10% aq. solution, 10 mL) twice, Na_2_SO_3_ aq. Solution, and brine, dried with Na_2_SO_4_, filtered and concentrated. Purification by flash chromatography (SiO_2_, hexane/EtOAC 1 : 1) yielded compound 25 (600 mg, 92%) as a yellow oil. ^1^H NMR (800 MHz, CDCl_3_) *δ* 7.17 (d, *J* = 8.4 Hz, 1H, H5), 7.02 (d, *J* = 8.4 Hz, 1H, H-4), 6.07–6.00 (m, 1H, H-9), 5.83 (m, 1H, H-8), 4.74 (m, 1H, H-7), 2.18 (m, 1H, H-10), 2.10–2.01 (m, 1H, H-10), 1.98–1.88 (m, 3H, H-12 and H-11), 1.72–1.63 (m, 1H, H-11). ^13^C NMR (201 MHz, CDCl_3_) *δ* 153.8 (C-3), 141.5 (C-6), 134.0 (C-9), 124.5 (C-8), 123.6 (C-5), 122.5 (C-4), 111.3 (C-2), 73.9 (C-7), 28.3 (C-12), 25.1 (C-10), 18.8 (C-11). HRMS (MALDI TOF) calcd for C_11_H_11_ClINO 335.9652 [M + H]^+^, found *m*/*z* 335.9652.

#### 2-Chloro-5a,6,7,8,9,9a-hexahydrobenzofuro[3,2-*b*]pyridine (26)

To an oven-dried sealed flask a solution of SmI_2_ in THF (15 mL) was added under N_2_ atmosphere. Compound 25 (200 mg, 0.6 mmol) dissolved in dry Et_3_N (630 μL) was added dropwise. Then, Milli-Q and deoxygenated H_2_O (80 μL) was added dropwise, the initially dark blue solution turned white and a large amount of precipitate is formed. To the quenched solution CH_2_Cl_2_ was added. The combined organic phase was washed twice with HCl (0.1 M) and aq. Na_2_S_2_O_3_ solutions. The clear organic layer was dried with Na_2_SO_4_, filtered and concentrated. Purification by flash chromatography (SiO_2_) using pentane/Et_2_O (9 : 1) yielded 26 (50 mg, 40% yield) along with the 2-chloro-5-(cyclohex-2-en-1-yloxy)pyridine, that is deiodinated 25 (30 mg, 24% yield) and 25 (10 mg). The two enantiomers of 26 were separated on HPLC. Analytical HPLC was performed using a Lux 5μm Amylose-1 column (50 × 4.6 mm) using i-PrOH : CH_3_CN 4 : 1 with 1 mg mL^−1^ flow rate. Preparative HPLC was performed on a Lux 5μm Amylose-1 column (250 × 21.6 mm) using i-PrOH : CH_3_CN (40 : 30) as eluent with 15 ml min^−1^ (4 min) and subsequently 21 ml min^−1^ flow rate. Enantiomer 26a [*α*]^20^_D_ −110 (c 9.9 mg mL^−1^, CH_2_Cl_2_), enantiomer 26b [*α*]^20^_D_ +94 (c 9.8 mg mL^−1^, CH_2_Cl_2_). ^1^H NMR (400 MHz, CDCl_3_) *δ* 7.05–6.97 (m, 2H, H-3 and H-4), 4.81 (dt, *J* = 7.2, 4.9 Hz, 1H, H-5a), 3.23 (q, *J* = 7.2 Hz, 1H, H-9a), 2.01–1.92 (m, 2H, H9 and H-6), 1.91–1.82 (m, 1H, H-6), 1.69–1.58 (m, 1H, H-9), 1.56–1.46 (m, 3H, H-3, H-7 and H-8), 1.38 (m, 1H, H-8). ^13^C NMR (101 MHz, CDCl_3_) *δ* 156.5 (C-9b), 152.6 (C-9a), 142.2 (C-2), 122.4 (C-py), 119.0 (C-py), 83.5 (C-5a), 41.2 (C-9a), 27.6 (C-6), 26.5 (C-9), 21.9 (C-8), 20.2 (C-7). HRMS (MALDI TOF) calcd for C_11_H_12_ClNO 210.0686 [M + H]^+^, found *m*/*z* 210.0675.

#### 2-((Triisopropylsilyl)ethynyl)-5a,6,7,8,9,9a-hexahydrobenzofuro[3,2-*b*]pyridine (27)

To an oven-dried microwave vial CsCO_3_ (330 mg, 1.0 mmol), XPhos (31 mg, 0.065 mmol) and Pd(CH_3_CN)_2_Cl_2_ catalyst (5.5 mg, 0.02 mmol) were added under N_2_ atmosphere. The vial was sealed and filled with N_2_. A pure enantiomer of compound 26 (65 mg, 0.31 mmol) dissolved in deoxygenated dry CH_3_CN (10 mL) was added. N_2_ was bubbled through this solution for 10 min. Triisopropylsilylacetylene (230 μL, 1.0 mmol) was added dropwise *via* a syringe. The resulting reaction mixture was heated at 90 °C for 18 h. The flask was allowed to cool to r.t., the mixture was dissolved with Et_2_O and washed twice with brine. The organic phase was dried with Na_2_SO_4_, and the solvent was removed under reduce pressure to yield a brown oil, which was purified by flash chromatography with pentane : Et_2_O (90 : 10) as eluent. Compound 27 was obtained as a pale brown oil (75 mg, 68% yield). ^1^H NMR (400 MHz, CDCl_3_) *δ* 7.25 (d, *J* = 8.2 Hz, 1H, H-3), 6.94 (d, *J* = 8.2 Hz, 1H, H-4), 4.77 (dt, *J* = 7.0, 4.7 Hz, 1H, H-5a), 3.20 (q, *J* = 7.0 Hz, 1H, H-9a), 2.21–1.92 (m, 2H, H-9 and H-6), 1.93–1.65 (m, 1H, H6), 1.65–1.46 (m, 4H, H9 and H8 and H7), 1.45–1.29 (m, 1H, H-8), 1.12 (s, 21H, TIPS). ^13^C NMR (101 MHz, CDCl_3_) *δ* 156.9 (C-9b), 153.0 (C-4a), 135.1 (C-2), 127.8 (C-3), 115.1 (C-4), 106.6 (C10), 89.0 (C-11), 83.2 (C-5a), 41.2 (C-9a), 27.6 (C-6), 26.8 (C-9), 22.0 (C-8), 20.2 (C-7), 18.8 (CH_3_, TIPS), 11.5 (C, TIPS).

#### 2-Ethynyl-5a,6,7,8,9,9a-hexahydrobenzofuro[3,2-*b*]pyridine (28)

A solution of TBAF in THF (1.0 M, 0.230 mL, 0.23 mmol, 1.1 equiv.) was added to a solution of TIPs-protected alkyne 7 (75 mg, 0.21 mmol) in dry THF (2.0 mL). The reaction mixture was stirred at 0 °C for 2 h under N_2_ atmosphere. After being warmed to room temperature, the reaction mixture was diluted in diethyl ether and washed two times with sodium chloride saturated aqueous solution. The organic phase was dried over Na_2_SO_4_ and the solution was subsequently concentrated under reduced pressure. The crude mixture was purified by column chromatography on silica gel with pentane/diethyl ether (8 : 2) to give 28 as crystalline brown solid (33 mg, 78% yield). Enantiomer 28a [*α*]^20^_D_ −137 (c 2.7 mg mL^−1^, CH_2_Cl_2_). Enantiomer 28b [*α*]^20^_D_ +125.5 (c 4.5 mg mL^−1^, CH_2_Cl_2_). ^1^H NMR (400 MHz, CDCl_3_) *δ* 7.25 (d, *J* = 8.2 Hz, 1H, H-3), 6.96 (d, *J* = 8.2 Hz, 1H, H-4), 4.82 (dt, *J* = 7.2, 5.0 Hz, 1H, H-5a), 3.24 (q, *J* = 7.3 Hz, 1H, H-9a), 3.05 (s, 1H, -C≡CH, H-11), 2.06–1.82 (m, 3H), 1.76–1.58 (m, 1H), 1.57–1.45 (m, 3H), 1.45–1.32 (m, 1H). ^13^C NMR (101 MHz, CDCl_3_) *δ* 157.0 (C-9b), 153.5 (C-4a), 133.7 (C-2), 127.1 (C-3), 116.0 (C-4), 83.3 (C-10), 83.3 (C-5a), 75.4 (-C11), 41.1 (C-9a), 27.6 (C-6), 26.4 (C-9), 21.9 (C-8), 20.2 (C-7). HRMS (MALDI TOF) calcd for C_13_H_13_NO 200.1075 [M + H]^+^, found *m*/*z* 200.1063.

### Syntheses of 1,2-bis((6-(*N*-((1*R*)-1-phenylethyl)amino)pyridin-2-yl)ethinyl)benzene (5) and 1,2-bis((6-(*N*-methyl-N-((1*R*)-1-phenylethyl)amino)pyridin-2-yl)ethinyl)benzene (7)

#### 1,2-Bis((6-brompyridin-2-yl)ethynyl)benzene (29)

A microwave vial was charged with 2,6-dibrompyridine (1.878 g, 7.927 mmol, 2.00 eq.), Pd(PPh_3_)_4_ (229 mg, 0.198 mmol, 0.05 eq.) and CuI (75.5 mg, 0.396 mmol, 0.10 eq.), and was alternatingly evacuated and refilled with N_2_ trice. Next, Et_3_N (20 mL) and 1,2-diethynylbenzene (500 mg, 3.963 mmol, 1.00 eq.) were added. The mixture was stirred first at room temperature for 1 h and then heated to 110 °C overnight. The reaction mixture was deactivated with brine, mixed with Et_2_O, and the organic phase was washed trice with brine to remove Et_3_N. The combined organic layer was dried with Na_2_SO_4_. Column chromatography (silica, hexane/Et_2_O 70 : 30, *R*_F_(product) = 0.21) gave pure 29 (155 mg, 0.354 mmol, 9%). ^1^H NMR (500 MHz, CDCl_3_): *δ* 7.88–7.82 (d, *J* = 8.0 Hz, 2H, H-3′′), 7.69–7.59 (m, 4H, H-4′′, H-3, H-6), 7.49–7.44 (d, *J* = 7.7 Hz, 2H, H-5′), 7.40 (dd, *J* = 5.8, 3.4 Hz, 2H, H-4, H-5). ^13^C NMR (126 MHz, CDCl_3_): *δ* [143.9 (C-2′′), 141.8 (C-6′′), 138.8 (C-4′′), 132.2 (C-5′′), 129.3 (C-4, C-5), 127.7 (C-1, C-2), 127.0 (C-3, C-6), 125.5 (C-3′′), 92.3 (C-2′), 89.2 (C-1′). LC-MS (ESI) [*m*/*z*]: [M + H]^+^ 437.0.

### Computations

We applied density functional theory (DFT) in our present work to characterize the electronic structure of the investigated complexes. Truhlar's M06-2X hybrid-type exchange–correlation functional^74^ was used along with the Def2-SVP basis set^75^ for geometry optimizations and vibrational analysis. This choice for the approximated functional is justified by previous benchmark studies,^76,77^ which reported good performance of M06-2X for non-covalent and halogen bonding interactions, both important in our computational analysis. For each optimized structure, additional single-point energy calculations were performed with the larger Def2-TZVPP basis set.^75^ All these calculations (geometry optimizations and vibrational analysis as well) were carried out with the inclusion of solvent effects *via* the integral equation formalism of the polarizable continuum model (IEFPCM).^78^ The atomic radii and non-electrostatic terms in the IEFPCM calculations were those introduced by Truhlar and coworkers in terms of the SMD solvation model.^79^ For iodine, the atomic radius was set to 2.74 Å as suggested in the SMD18 refinement of the model.^80^ We used dichloromethane as a typical solvent utilized in our experiments.

The thermal and entropic contributions to the Gibbs free energies were computed for 298.15 K and *c* = 1 mol dm^−3^ conditions and employing Grimme's quasi-RRHO approximation.^81^ This approach is expected to be more appropriate than the standard ideal gas RRHO (rigid rotor – harmonic oscillator) model, because the optimized structures of the present iodonium complexes have several low harmonic frequency modes (skeletal vibrations). The relative stabilities reported in our paper correspond to solution phase Gibbs free energies computed as *G* = *E*′_*0,sol*_ + (*G*_*0,sol*_−*E*_*0,sol*_), where *E*′_*0,sol*_ and *E*_*0,sol*_ are solution phase electronic energies obtained at the M06-2X/Def2TZVPP and M06-2X/Def2SVP levels, respectively, and *G*_*0,sol*_ is solution phase Gibbs free energy computed at M06-2X/Def2SVP level. All DFT calculations were carried out with the Gaussian16 software.^82^ The molecular structures were visualized with the *CYLview* program.^83^

The structure of the iodonium complex [4-I]^+^ examined computationally, particularly the singly coordinated open form, is conformationally complex, therefore, we used initial conformational screening to map possible structures. Our conformational analysis involved a Monte Carlo sampling using the OPLS_2005 force field as implemented in MacroModel,^84^ as well as the metadynamics sampling procedure provided by the *CREST* (Conformer–Rotamer Ensemble Sampling Tool)^85^ utility of the xtb program package.^86^ A large number of conformers were identified *via* this initial screening, and many of them (about 70 different structures) were subject to subsequent DFT calculations. We report the most stable and structurally distinct structures (ESI†).

## Conflicts of interest

There are no conflicts to declare.

## Supplementary Material

OB-019-D1OB01532J-s001
