# Supplementary material for: Are bis(pyridine)iodine(i) complexes applicable for asymmetric halogenation?
Source: Org Biomol Chem. 2021 Sep 9;19(38):8307–23. doi: 10.1039/d1ob01532j (PMC8494190; doi:10.1039/d1ob01532j)
Supplement: OB-019-D1OB01532J-s001 [file OB-019-D1OB01532J-s001.pdf]

This journal is © The Royal Society of Chemistry 2021

Electronic Supplementary Information

Are bis(pyridine)iodine(I) complexes applicable for asymmetric halogenation?

Daniel von der Heiden, Flóra Boróka Németh, Måns Andreasson, Daniel Sethio, Imre Pápai, and Mate Erdelyi

Table of Contents

1. Synthesis ... S2

1.1. *rac*-4-Iodo-5-phenyldihydrofuran-2(3*H*)-one (**30**) ... S2

1.2. 5-Phenyl-hex-5-enoic acid (**31**) ... S2

1.3. 6-(Iodomethyl)-6-phenyltetrahydro-2*H*-pyran-2-one (**32**) ... S3

1.4. 4-Phenyl-penten-5-enoic acid (**33**) ... S4

1.5. 5-(Iodomethyl)-5-phenyldihydrofuran-2(3*H*)-one (**34**) ... S4

1.6. *rac*-(6-Iodohexahydro-2H-3,5-methanocyclopenta[*b*]furan-7-yl)methanol (**35**) ... S5

1.7. *rac*-6-Iodohexahydro-*2H*-3,5-methanocyclopenta[*b*]furan-7-yl)methyl benzoate (**36**) ... S5

1.8. 4-Phenyl-but-3-enol (**37**) ... S6

1.9. *rel-(1S,2R)*-2-Iodo-1-phenyl-tetrahydrofuran (**38**) ... S6

1.10. *In situ* generation of iodine(I) complexes of bis(pyridine)-type ligands using *N*-iodsuccinimide S8

2. Enantioselective iodolactonisation ... S12

2.1. Error analysis ... S12

2.2. Formation of iodine(I) complexes from silver(I) complex and I_2 ... S14

2.3. Formation of iodine(I) complexes from pyridinium ligand and NIS ... S21

2.4. Enantioselective iodoetherification – norbornene-2.3-dimethanol desymmetrisation ... S26

3. Spectra ... S30

4. COMPUTATIONS ... S95

4.1. Optimized structures ... S95

4.2. Total energy data ... S96

4.3. Noncovalent interaction analysis ... S96

4.4. Cartesian coordinates ... S98

5. References ... S120

1. Synthesis

1.1. *rac*-4-Iodo-5-phenyldihydrofuran-2(3H)-one (30)

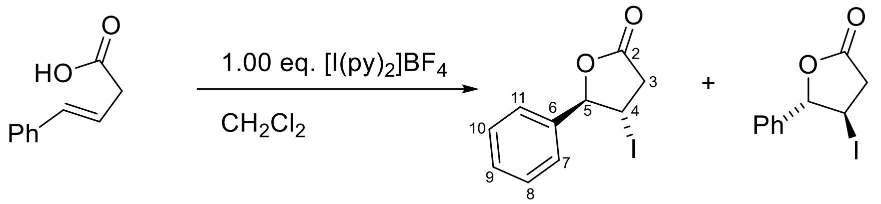

An oven dried microwave vial was charged with Barluenga's reagent (187.0 mg, 0.50 mmol, 1.00 eq.) and CH_2Cl_2 (9 mL). The mixture was cooled to –78 °C (aceton/dry ice) and *trans*-4-phenylbut-3-enoic acid (82.7 mg, 0.51 mmol, 1.02 eq.) in CH_2Cl_2 (1 mL) was added dropwise. The mixture was stirred for 1 h then let to reach r.t. and stirred for another hour. The mixture was diluted with 40 mL CH_2Cl_2, mixed with silica and the solvent was removed in vacuum to apply the mixture to flash column chromatography (Silica 'Sfar HC 10g' column, ~20 g/mmol starting material, *n*-hexane/EtOAc 95:5 *v*/*v* [1CV], then 95:5 to 80:20 over [10CV], then 80:20 [5CV]. to give *rac*-product (110 mg, 76 %). The product was weakly UV/VIS active at λ = 250 nm.) Analytical data are in agreement with data reported previously in the literature.[1]
Analytical HPLC (Lux® 5 µm Amylose-1, LC Column 250 x 4.6 mm, H_2O/CH_3CN 20:80, 1 ml/min. R_t[enantiomer1] = 2.06 min, R_t [enantiomer2] = 4.66 min. Analytical HPLC (Lux® 5 µm Amylose-1, LC Column 250 x 4.6 mm, H_2O/CH_3CN 40:60, 1 ml/min. R_t[enantiomer1] = 3.72 min, R_t [enantiomer2] = 9.872 min.

^{1}H NMR (400 MHz, $CDCl_3$) δ 7.41 (m, 5H, 7–H, 8–H, 9–H, 10–H, 11–H), 5.68 (d, $^3J_{HH}$ = 7.2 Hz, 1H, 2–H), 4.25 (q, $^3J_{HH}$ = 8.3 Hz, 1H), 3.27 (dd, $^3J_{HH}$ = 18.0, 7.8 Hz, 1H), 3.03 (dd, $^3J_{HH}$ = 18.0, 9.1 Hz, 1H, 4). ^{13}C NMR (101 MHz, $CDCl_3$) δ 173.7 (C-5), 135.8 (C-6), 129.6 (C-9), 129.1 (C-8, C-10), 126.1 (C-7, C-11), 89.6 (C-2), 41.1 (C-4), 17.9 (C-3).

1.2. 5-phenyl-hex-5-enoic acid (31)

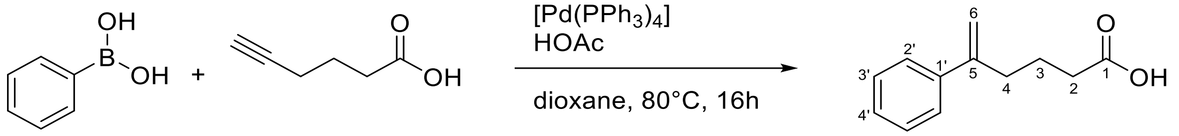

An oven dried round-bottom-flask was charged with phenylboronic acid (3.00 g, 24.6 mmol, 1.2 eq.), $Pd(PPh_3)_4$ (1.178 g, 1.02 mmol, 0.05 eq.) and dry dioxane (100 mL). Next 5-Hexynoic acid (2.31 ml, ρ = 0.99 g/mL, 20.4 mmol, 1.00 eq.) and acetic acid (117 µL, 2.04 mmol, 0.10 eq.) were added and the mixture was heated to 80°C overnight (ca. 16 h). The solvent was removed in vacuum and the crude product has been purified by (Silica column, CH_2Cl_2) to remove the catalyst and the boronic acid residues. The resulting product mixture was the separated by preparative HPLC (Lux Amylose, 20 mL/min, H_2O/CH_3CN, 75:25, 45 min, R_t = 33 min product) and afford the pure product.

^{1}H NMR (400 MHz, $CDCl_3$) δ 9.21 (s, 1H, O–H), 7.39 (d, *J* = 7.1 Hz, 2H, H–2'), 7.32 (t, *J* = 7.4 Hz, 2H, H–3'), 7.26 (overlap with $CHCl_3$, s, 5H, expected 1H, H–4'), 5.31 (s, 1H, H–6a or 6b), 5.08 (s, 1H, H–6a or 6b), 2.58 (t, *J* = 7.5 Hz, 2H, H–4), 2.38 (t, *J* = 7.4 Hz, 2H, H–2), 1.96 – 1.66 (m, 2H, H–3). ^{13}C NMR (101 MHz, CDCl3) δ 179.3 (C-1), 147.5 (C-5), 140.9 (C-1'), 128.5 (C-3', C-5'), 127.6 (C-4'), 126.3 (C-2', C-6'), 113.2 (C-6), 34.6 (C-4), 33.3 (C-2), 23.2 (C-3).

1.3. 6-(Iodomethyl)-6-phenyltetrahydro-2H-pyran-2-one (32)

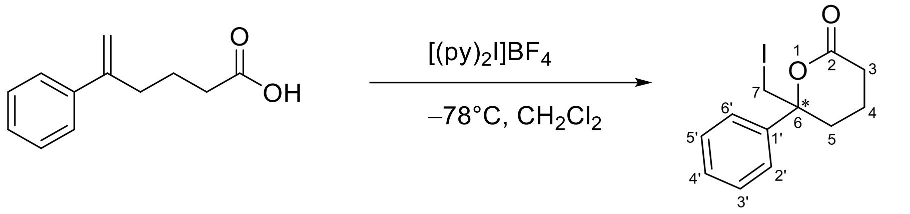

In two oven dried microwave vials two solutions of 5-phenyl-hex-5-enoic acid (40.0 mg, 0.210 mmol, 1.00 eq.) dissolved in CH_2Cl_2 (2mL) and $[(py)_2I]BF_4$ (80.2 mg, 0.215 mmol, 1.00 eq.) dissolved in CH_2Cl_2 (2 mL) were prepared. The solutions were mixed at –78 °C, stirred for 30 min, then the cooling bath was removed and the solution was stirred for another 30 min. The reaction was deactivated by brine (2mL), extracted with CH_2Cl_2 and the combined organic layer was dried over Na_2SO_4. The solvent was removed in vacuum, and the mixture was purified by column chromatography (silica, *n*-hexane/EtOAc 80:20, R_F = 0.33 product). The crude product was further purified by preparative HPLC (Kinetex C8, LC Column 250 x 4.6 mm, H_2O/CH_3CN 50:50, 20 min^{-1}, R_t [product] = 10.6 min) to afford the racemic product (61 mg, 0.193 mmol, 92%). ^{1}H NMR (400 MHz, $CDCl_3$) δ 7.47 – 7.30 (m, 5H, H-2', H-3', H-4', H-5', H-6'), 3.57 (s, 2H, H-7), 2.56 – 2.29 (m, 4H, H-3, H-5), 1.88 – 1.76 (m, 1H, H-4a), 1.66 – 1.57 (m, 1H, H–4b). ^{13}C NMR (101 MHz, $CDCl_3$) δ 170.6 (C-2), 140.4 (C-1'), 129.2 (C-3', C-5'), 128.6 (C-4'), 125.4 (C-2', C-6'), 84.6 (C-6), 32.2 (C-5), 29.1 (C-3), 17.8 (C-7), 16.7 (C-4).

Analytical HPLC (Lux® 5 µm i-Amylose-1, LC Column 250 x 4.6 mm, *n*-hexane/*i*PrOH 95:05, recommend to inject as toluene solution, 1 ml/min. R_t[enantiomer1] = 18.0 min, R_t [enantiomer2] = 19.5 min, 254 nm).

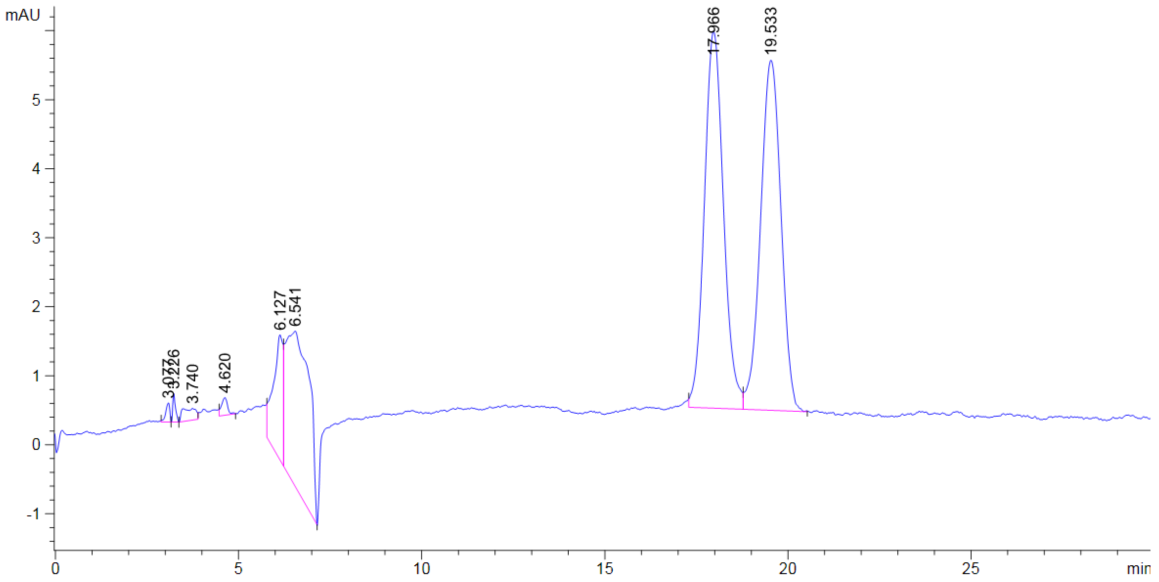

Figure S1. HPLC chromatograms of the enantiomers of 6-(iodomethyl)-6-phenyltetrahydro-2*H*-pyran-2-one.

1.4. 4-Phenyl-penten-5-enoic acid (33)

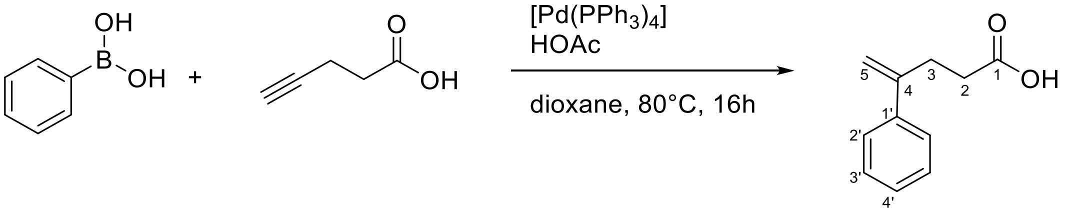

An oven dried round-bottom-flask was charged with phenylboronic acid (2.49 g, 20.4 mmol, 1.0 eq.), [Pd(PPh3)] (1.178 g, 1.02 mmol, 0.05 eq.) and dry dioxane (100 mL). Next 4-pentynoic acid (2.31 ml, ρ = 0.99 g/mL, 20.4 mmol, 1.00 eq.) and acetic acid (150 μL, ρ = 1.05 g mL^{-1}, 2.04 mmol, 0.10 eq.) were added and the mixture was heated to 90°C overnight (ca. 16 h). The solvent was removed in vacuum and the crude product has been purified by (Silica column, CH_2Cl_2) to remove the catalyst and the boronic acid residues. The resulting product mixture was the separated by preparative HPLC (Lux Amylose, 20 mL/min, H_2O/CH_3CN, 75:25, 45 min, R_t = 33 min product) and afford the pure product. ^{1}H NMR (400 MHz, $CDCl_3$) *δ* 7.40 (d, *J* = 7.5 Hz, 2H, H–2'), 7.34 (t, *J* = 7.4 Hz, 2H, H–3'), 7.29 (d, *J* = 7.3 Hz, 1H, H–4'), 5.33 (s, 1H, H–5a or 5b), 5.12 (s, 1H, H–5a or 5b), 2.86 (t, *J* = 7.8 Hz, 2H, H–3), 2.54 (dd, *J* = 8.9, 6.6 Hz, 2H, H–2).

1.5. 5-(Iodomethyl)-5-phenyldihydrofuran-2(3H)-one (34)

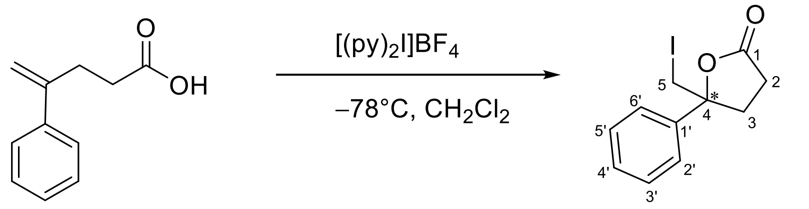

In two oven dried microwave vials were prepared two solutions of 4-phenyl-penten-5-enoic acid (40.0 mg, 0.227 mmol, 1.00 eq.) dissolved in CH_2Cl_2 (2mL) and $[(py)_2I]BF_4$ (84.9 mg, 0.227 mmol, 1.00 eq.) dissolved in CH_2Cl_2 (2 mL). The solutions were mixed at –78 °C, stirred for 30 min, then the cooling bath has been removed and the solution was stirred for another 30 min. The solvent was removed in vacuum, and the mixture was purified by column chromatography (silica, *n*-hexane/EtOAc 80:20, R_F = 0.33 product) afford the racemic product (37 mg, 0.123 mmol, 54%).

Analytical HPLC (Lux® 5 μm Amylose-1, LC Column 250 x 4.6 mm, H_2O/CH_3CN 60:40, 1 ml/min. R_t[enantiomer1] = 8.5 min, R_t [enantiomer2] = 9.5 min).

^{1}H NMR (400 MHz, $CDCl_3$) *δ* 7.44 – 7.30 (m, 5H, H–2', H–3', H–4', H–5', H–6'), 3.62 (AA', 2H, H–5'), 2.82 – 2.45 (m, 4H, H–2, H–3). ^{13}CNMR (101 MHz, $CDCl_3$) δ 175.4 (C-1), 140.7 (C-1'), 128.9 (C-3', C-5'), 128.7 (C-4'), 125.0 (C-2', C-6'), 86.1 (C-4), 34.1 (C-3), 29.3 (C-2), 16.4 (C-5).

1.6. *rac*-(6-iodohexahydro-2H-3,5-methanocyclopenta[b]furan-7-yl)methanol (35)

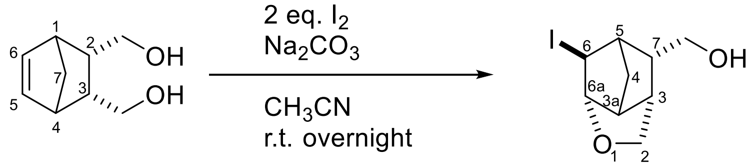

5-Norbornene-2-endo,3-endo-dimethanol (200 mg, 1.30 mmol, 1.00 eq.) was dissolved in 11 mL CH_3CN, Na_2CO_3 (137.5 mg, 1.3mmol, 1.00 eq.) and finally iodine (329.2 mg, 1.30 mmol, 1.00 eq.) was added. The mixture was stirred overnight, and the progress was monitored via TLC (100%EtOAc, MnO_4^-, R_f=0.77) a second equivalent Iodine was added to drive the reaction to completion. The reaction was deactivated with aqueous Na_2SO_3 extracted with Ethyl acetate and the crude product was purified by flash column chromatography (*n*-Hexane/EtOAc 70:30 [1CV] to 0:100 [8CV] then 0:100 [5CV]). 320 mg (88 %) product was isolated as a colourless solid. ^{1}H NMR (400 MHz, $CDCl_3$) *δ* 4.66 (d, *J* = 5.3 Hz, 1H, 6a–H), 3.75 (d, *J* = 2.7 Hz, 1H, 6–H), 3.65 (d, *J* = 9.0 Hz, 1H, 2'–H), 3.57 (dd, *J* = 7.9, 3.0 Hz, 2H, 8'–H), 3.52 (dd, *J* = 8.9, 4.3 Hz, 1H, 2'–H, 2''–H), 2.97 (s, 1H, 12–H), 2.73 – 2.58 (m, 1H, 3a–H), 2.44 – 2.41 (m, 1H, 5–H), 2.41 – 2.38 (m, 1H, 3–H), 2.19 (qd, *J* = 7.9, 4.0 Hz, 1H, 7–H), 2.12 (d, *J* = 11.1 Hz, 1H, 4''–H), 1.77 – 1.70 (m, 1H, 4'–H). ^{13}C NMR (101 MHz, $CDCl_3$) *δ* 89.5 (C-6a), 68.2 (C-2), 59.8 (C-8), 48.0 (C-3a), 45.8 (C-3), 44.3 (C-7), 38.2 (C-5), 37.2 (C-4), 33.6 (C-6).

1.7. *rac-6-iodohexahydro-2H-3,5-methanocyclopenta[b]furan-7-yl)methyl benzoate* (36)

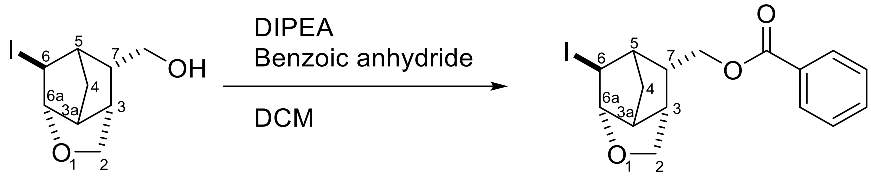

rac-(6-iodohexahydro-2H-3,5-methanocyclopenta[b]furan-7-yl)methanol (9.8 mg, 0.035 mmol, 1.00 eq.) was dissolved in CH_2Cl_2 (0.700 mL) and DIPEA (61 µL, 0.35 mmol, 10.0 eq.) and benzoic anhydride (79 mg, 0.35 mmol, 10 eq.) were added. The mixture was stirred for 4d at 50°C until full conversion has been monitored by NMR. CH_2Cl_2 has been evaporated and CH_3CN/H_2O was added to the reaction mixture and the crude mixture was separated on preparative HPLC to give the pure racemic product (11.4 mg, 0.0297 mmol, 85 %). Preparative HPLC (Lux® 5 µm Amylose-1, H_2O/CH_3CN 80:20, 80:20 [1.5min] to 10:90 [17.5min], 10:90 [20min], 15 ml/min. The isolated enantiomers have been freeze-dried and analysed by HPLC. Analytical HPLC (Lux® 5 µm Amylose-1, LC Column 250 x 4.6 mm, H_2O/CH_3CN 80:20[1min], 80:20 to 10:90 [10min], 10:90 [10min], 1 ml/min. R_t[enantiomer-1] = 12.4 min, R_t [enantiomer-2] = 13.5 min). The Integration Error is estimated to ~2 ee %. ^{1}H NMR (400 MHz, $CDCl_3$) *δ* 8.02 (d, *J* = 7.9 Hz, 2H, 3'–H, 7'–H), 7.57 (t, *J* = 7.4 Hz, 1H, 5'–H), 7.45 (t, *J* = 7.8 Hz, 2H, 4'–H, 6'–H), 4.77 (d, *J* = 5.2 Hz, 1H, 6a–H), 4.38 (d, *J* = 3.8 Hz, 2H, 8'–H), 3.94 (d, *J* = 2.7 Hz, 1H, 6–H), 3.78 (d, *J* = 9.3 Hz, 1H, 2''–H), 3.64 (dd, *J* = 9.4, 3.1 Hz, 1H, 2'–H), 2.76 (s, 1H, 3a–H), 2.54 (s, 3H, 3–H, 5–H, 7–H), 2.26 (d, *J* = 11.1 Hz, 1H, 4'–H), 1.84 (d, *J* = 11.8 Hz, 1H, 4''–H). ^{13}C NMR (126 MHz, $CDCl_3$) *δ* 166.5 (C-1'), 133.3 (C-5'), 130.1 (2'), 129.7 (C-3', C-7'), 128.6 (C-4', C-6'), 89.7 (C-6a), 68.3 (C-2), 62.7 (C-8), 48.2 (C-3a), 46.3 & 41.4 & 38.7 (C-3, C-5, C-7), 37.6 (C-4), 33.1 (C-6).

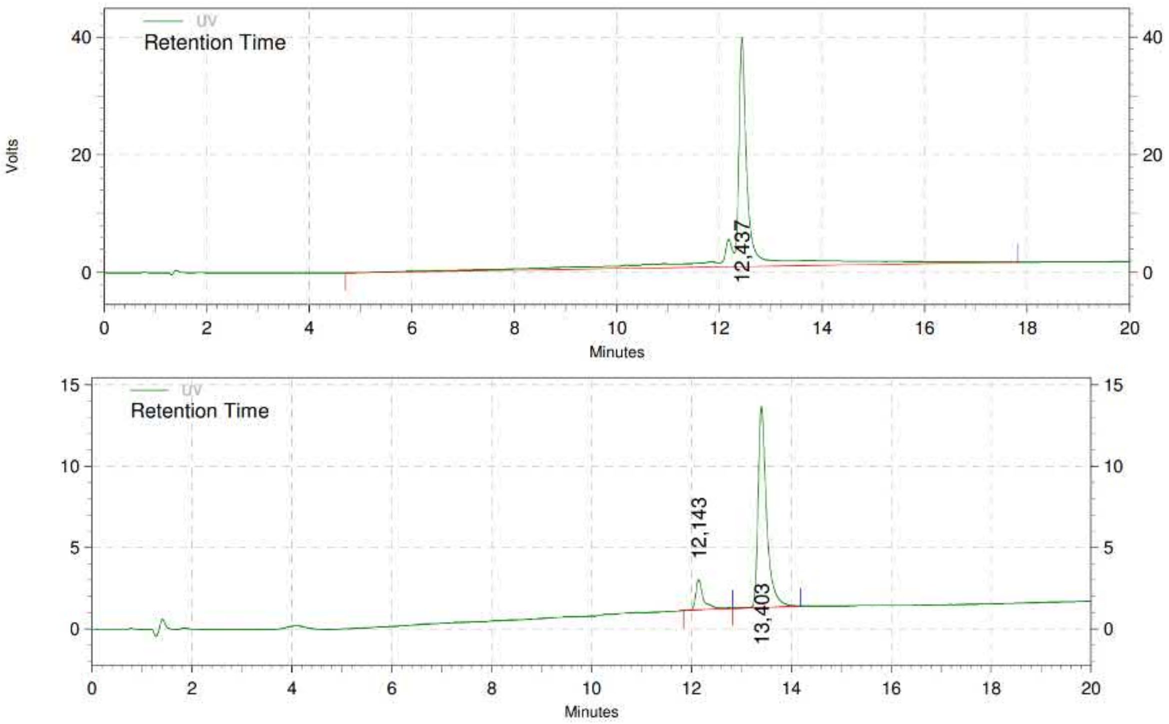

Figure S2. HPLC chromatograms of the isolated enantiomers of rac-6-iodohexahydro-2H-3,5-methanocyclopenta[b]furan-7-yl)methyl benzoate.

1.8. *4-phenyl-but-3-enol*[2] (37)

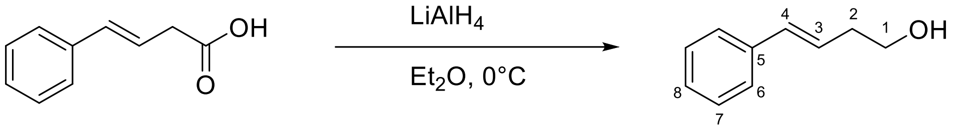

$LiAlH_4$ (936 mg, 24.7 mmol, 0.80 eq.) was suspended in Et_2O (100 mL) and cooled to 0°C. 4-phenyl-pent-3-enoic acid (5.00 g, 30.8 mmol, 1.00 eq.) was dissolved in Et_2O (50mL) and added dropwise to the cooled solution. After the addition has been completed and stirred for another 30 min, the cooling bath was removed and the mixture was stirred for 3 h until the reaction has been completed (LCMS). At 0°C 20 ml of water and 40 ml 2 M NaOH was added. The organic layer was separated and then dried over Na_2SO_4 to give the pure product (2.45 g, 16.5 mmol, 54 %). ^{1}H NMR (400 MHz, $CDCl_3$) δ 7.37 (d, J = 7.2 Hz, 2H, 6–H, 10–H), 7.31 (t, J = 7.5 Hz, 2H, 7–H, 9–H), 7.22 (t, J = 7.3 Hz, 1H, 8–H), 6.51 (d, J = 15.8 Hz, 1H, 4–H), 6.21 (dt, J = 15.2, 7.1 Hz, 1H, 3–H), 3.77 (t, J = 6.2 Hz, 2H, 1–H), 2.50 (q, J = 6.5 Hz, 2H, 2–H). ^{13}C NMR (100 MHz, $CDCl_3$) δ 137.4 (C-5), 133.0 (C-4), 128.7 (C-7, C-9), 127.4 (C-8), 126.5 (C-3), 126.2 (C-6, C-10), 62.2 (C-1), 36.6 (C-2).

1.9. *rel-(1S,2R)*-2-Iodo-1-phenyl-tetrahydrofuran (38)

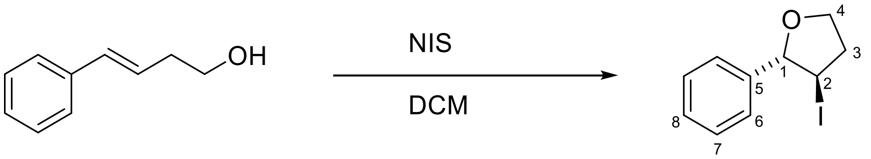

4-Phenyl-but-3-enol (31.0 mg, 0.209 mmol, 1.00 eq.) was dissolved in CH_2Cl_2 and *N*-iodosuccinimid (47.1 mg, 0.209 mmol, 1.00 eq.) was added. The mixture was stirred at r.t. until full conversion is indicated by LCMS. The solvent has been removed and the residue was purified by column

chromatography (Silica, ~15mg/g silica, n-Hex/EtOAc 2% [1CV], 2%-20% [10CV], retention of the product is ~2 CV). The solvent was removed in vacuum to obtain the pure product (16.5 mg, 0.060 mmol, 29 %). Analytical HPLC (Lux® 5 µm Amylose-1, LC Column 250 x 4.6 mm, H_2O/CH_3CN 80:20[1min], 80:20 to 10:90 [10min], 10:90 [10min], 1 ml/min. R_t[enantiomer1] = 10.16 min, R_t [enantiomer2] = 11.2 min). The Integration Error is estimated to ~0.5 ee %. Solvent for injection nHex/EtOAc 98:2. ^{1}H NMR (400 MHz, CD_2Cl_2) δ 7.44–7.28 (m, 5H, 6–H, 7–H, 8–H, 9–H, 10–H), 5.09 (d, J = 6.6 Hz, 1H, 1–H), 4.21 – 4.06 (m, 2H, 4–H), 4.03 (q, J = 7.0 Hz, 1H, 2–H), 2.58 (dq, J = 13.9, 7.2 Hz, 1H, 3–H), 2.37 (dddd, J = 13.3, 7.7, 6.6, 5.5 Hz, 1H, 3'–H). ^{13}C NMR (101 MHz, CD_2Cl_2) δ 140.5 (5–H), 129.0 (7–H, 9–H), 128.7 (8–H), 126.7 (10–H), 90.3 (1–H), 68.5 (4–H), 38.9 (3–H), 27.9 (2–H).

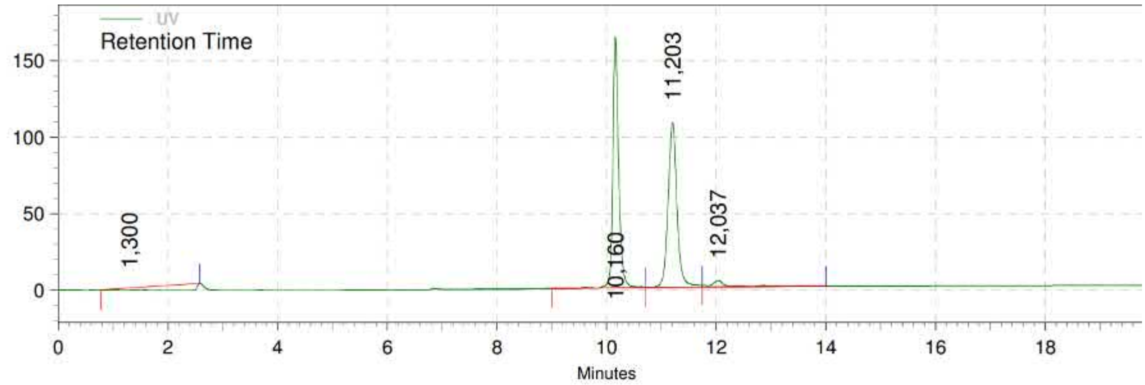

Figure S3. HPLC chromatograms of the isolated enantiomers of *rel*-(1*S*,2*R*)-2-iodo-1-phenyl-tetrahydrofuran.

1.10. *In situ* generation of iodine(I) complexes of bis(pyridine)-type ligands using *N*-iodsuccinimide

To a solution of the bidentate bis(pyridine)-type ligand, acid (HOTf or HNTf2, 1.eq.) was added. Next NIS was added and mixed carefully at r.t.. Iodine(I) transfer was confirmed by ^{15}N NMR following the iodine(I) transfer from NIS, and the protonation of succinimide. The amount of bis(pyridine)iodine(I)-type complex formed is not stoichiometric, but enough to detect its presence by ^{15}N NMR.

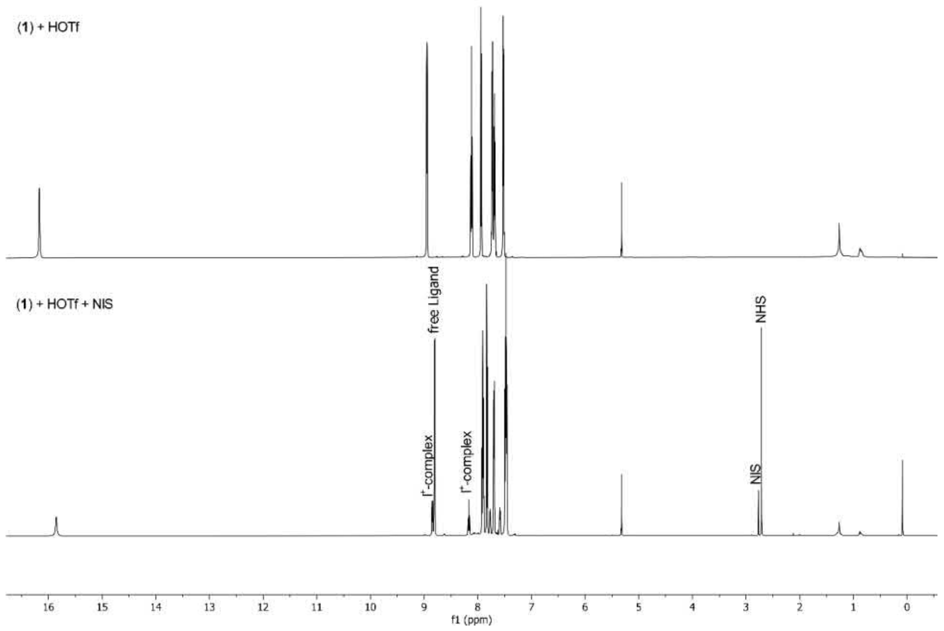

Figure S4. ^{1}H NMR spectrum (500 MHz, CD_2Cl_2, r.t.) before and after addition of *N*-iodsuccinimide to a solution of the [(**1**)-H]OTf complex. Relevant signals and integrals are indicated.

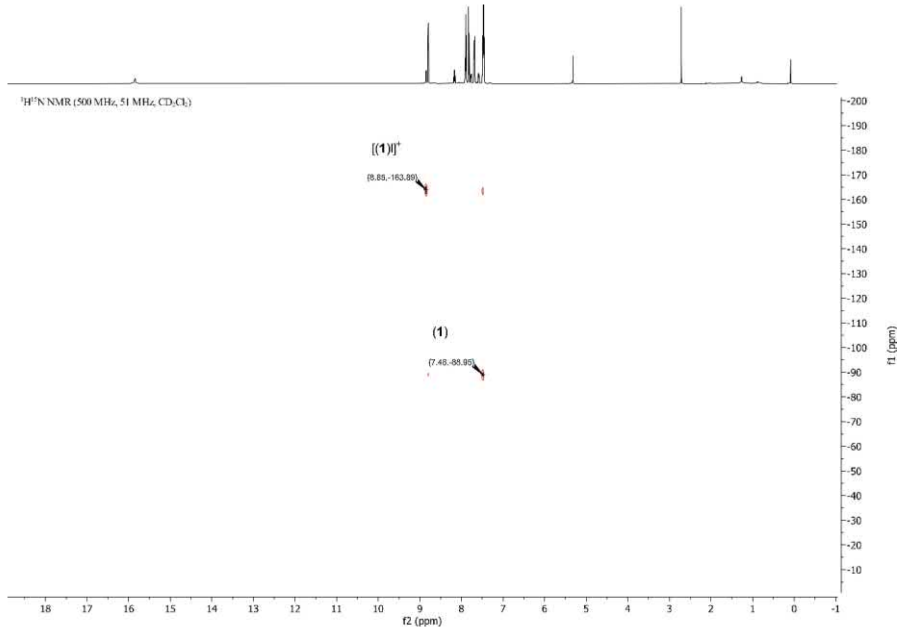

Figure S5. $^{1}H,^{15}N$ HMBC spectrum (500 MHz, 51 MHz, CD_2Cl_2) after addition of *N*-iodsuccinimide to a solution of the [(**1**)-H]OTf complex.

(R,S)-**4** (15 mg, 0.024 mmol, 1.00 eq.) was dissolved in CD_2Cl_2 and *N*-iodsuccinimide (16.2 mg, 0.72 mmol, 3 eq.) was added, chemical shift changes in ^{1}H, ^{13}C and ^{15}N NMR are shown in Figure S7-9. No ligand decomposition was observed within 1h at r.t..

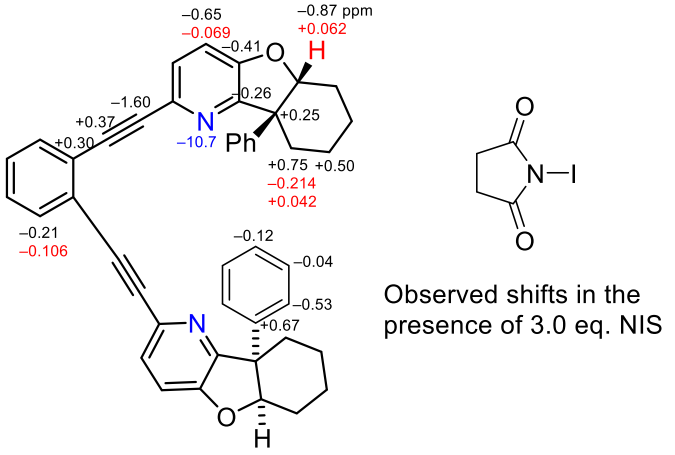

Figure S6. The NMR chemical shifts induced on (R,S)-**4** upon addition of NIS.

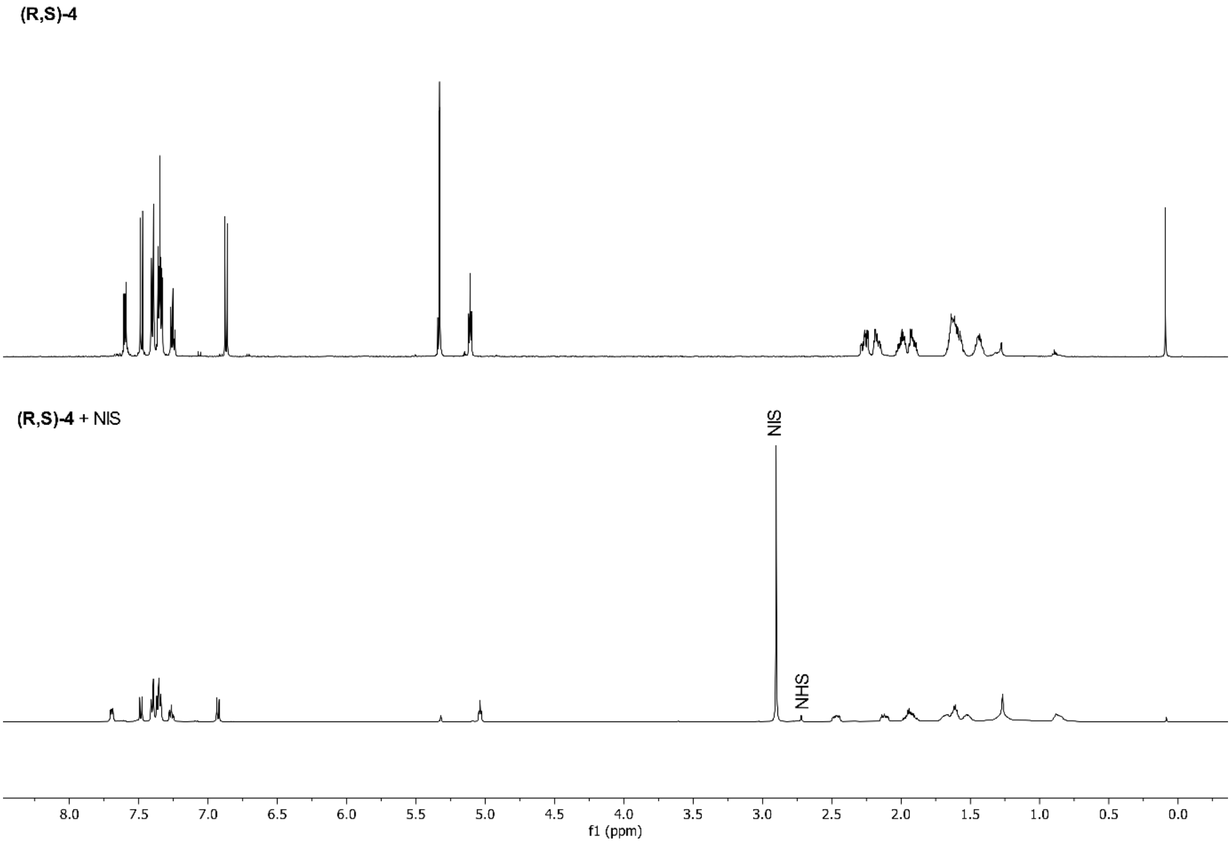

Figure S7. ^{1}H NMR spectrum of (R,S)-**4** upon addition of NIS in CD_2Cl_2 at 25°C (500 MHz).

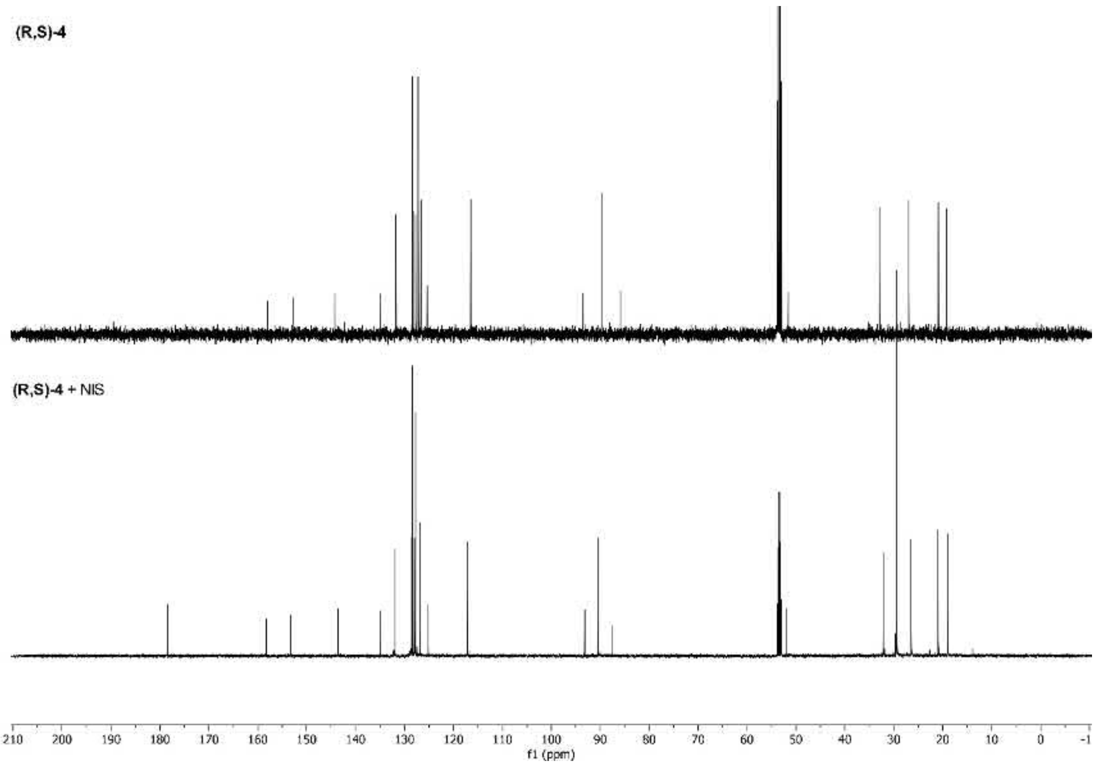

Figure S8. ^{13}C NMR spectrum of (*R*,*S*)-**4** upon addition of NIS in CD_2Cl_2 at 25°C (126 MHz).

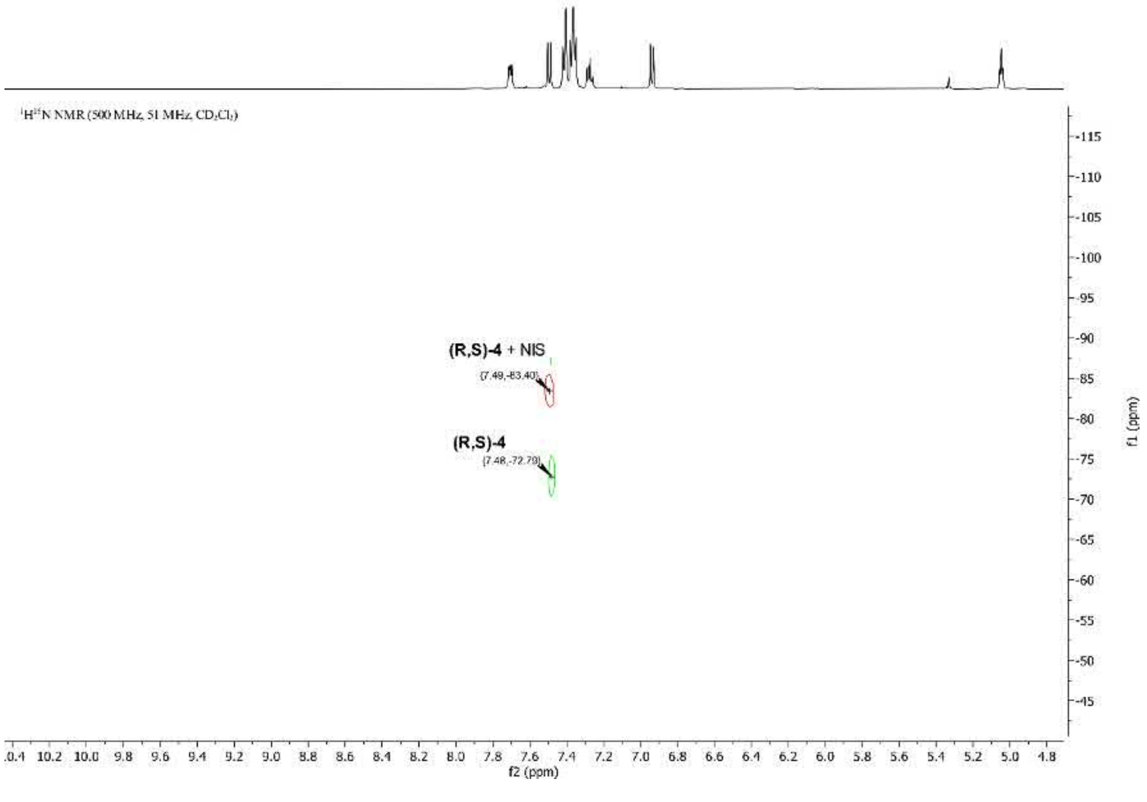

Figure S9. $^1H^{15}N$ HMBC spectrum of (*R*,*S*)-**4** upon addition of NIS in CD_2Cl_2 at 25°C (500/51 MHz).

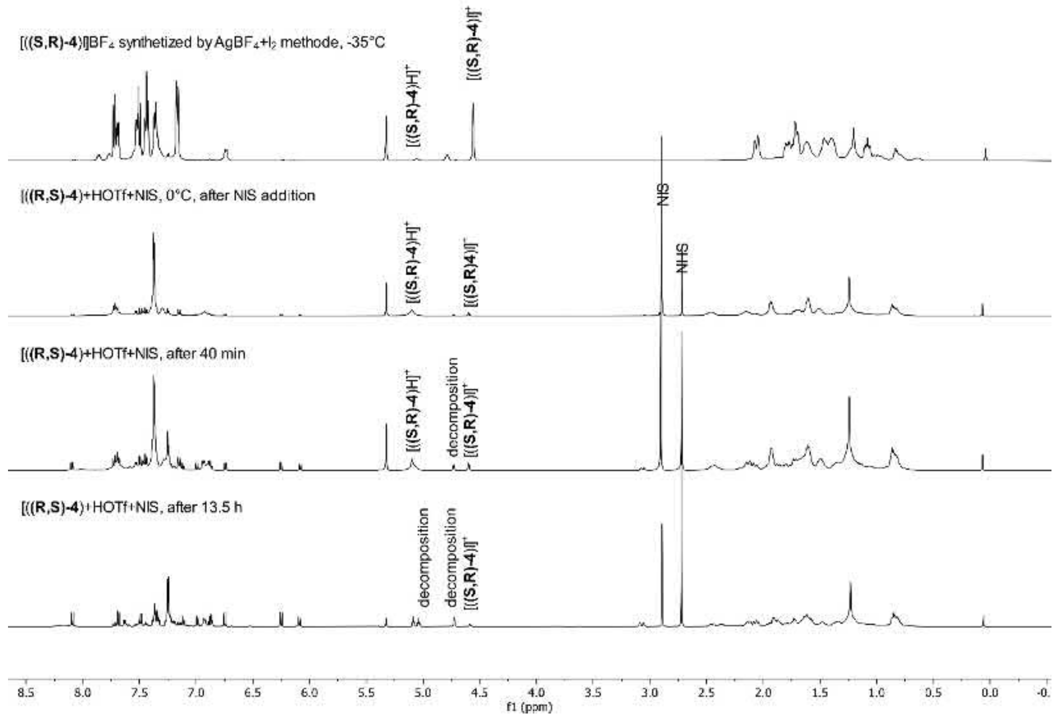

Figure S10. Addition of *N*-iodsuccinimide to a solution of the [((*R*,*S*)-**4**)-H]OTf complex, monitored by ^{1}H NMR (500 MHz, CD_2Cl_2). The NMR spectrum of the iodine(I)complex is shown on the top, the Iodine(I) transfer progresses over time leading to full decomposition.

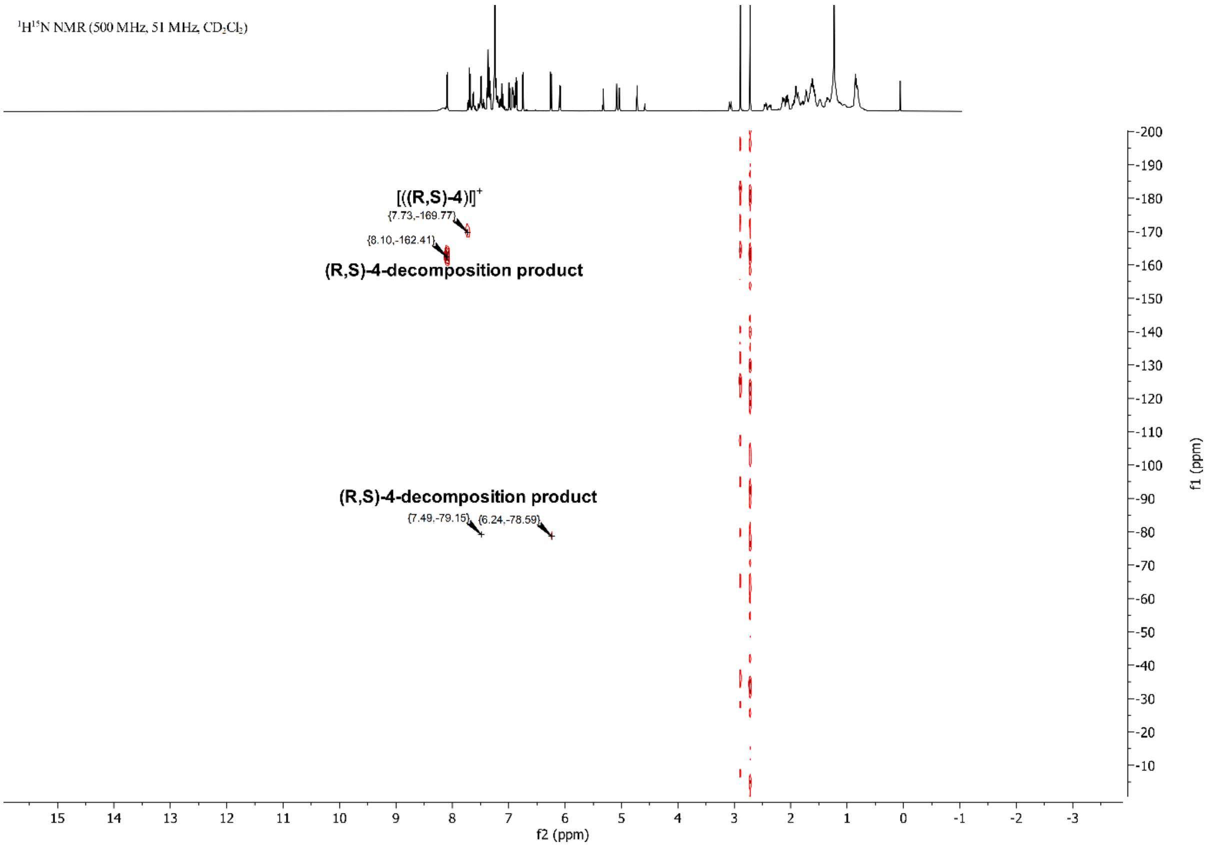

Figure S11. ^{1}H NMR spectrum (500 MHz, CD_2Cl_2) 1.5 h following addition of *N*-iodsuccinimide to a solution of the [((*R*,*S*)-**4**)-H]OTf complex.

2. Enantioselective iodolactonisation

2.1. Error analysis

To estimate the experimental error of a typical ee-determination via HPLC, a calibration for rel-(1*S*,2*R*)-2-iodo-1-phenyl-tetrahydrofuran towards biphenyl as a standard was performed. HPLC conditions: Detection wavelength (240 nm), Analytical HPLC (Lux® 5 µm Amylose-1, LC Column 250 x 4.6 mm, H_2O/CH_3CN 40:60, 1 ml/min. R_t[enantiomer1] = 3.72 min, R_t[biphenyl] = 4.98 min, R_t [enantiomer2] = 9.87 min.

Table S1. Concentration setup for the calibration experiment.

$c_{biphenyl}$ mol L^{-1}	$c_{enant1+enant2}$ mol L^{-1}	$c_{enant1}/c_{biphenyl}$	$c_{enant2}/c_{biphenyl}$	$I_{enant1}/I_{biphenyl}$	$I_{enant1}/I_{biphenyl}$
0.00099	0.00801	4.056	4.056	0.1303	0.1282
0.00101	0.00735	3.650	3.650	0.1170	0.1149
0.00101	0.00653	3.245	3.245	0.1020	0.1004
0.00101	0.00572	2.839	2.839	0.0916	0.0904
0.00101	0.00490	2.433	2.433	0.0771	0.0766
0.00101	0.00408	2.028	2.028	0.0653	0.0648
0.00101	0.00327	1.622	1.622	0.0512	0.0508
0.00101	0.00245	1.217	1.217	0.0394	0.0395
0.00101	0.00163	0.811	0.811	0.0248	0.0254
0.00101	0.00082	0.406	0.406	0.0128	0.0138
0.00101	0.00041	0.203	0.203	0.0070	0.0080
0.00099	0.00016	0.081	0.081	0.0024	0.0035

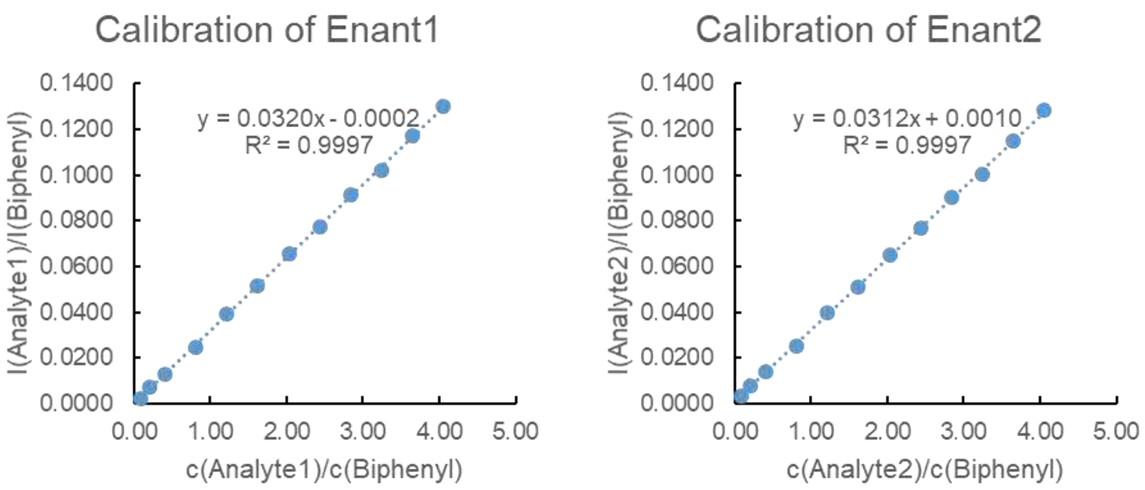

Figure S12. Correlation lines between concentrations of rel-(1S,2R)-2-iodo-1-phenyl-tetrahydrofuran and biphenyl standard. The linear relationship indicates a suitable concentration range for quantification.

Table S2. HPLC Chromatogram for the standard-substrate mixture. Detection wavelength (240 nm), Analytical HPLC (Lux® 5 µm Amylose-1, LC Column 250 x 4.6 mm, H_2O/CH_3CN 40:60, 1 ml/min. R_t[enantiomer1] = 3.72 min, R_t[biphenyl] = 4.98 min, R_t [enantiomer2] = 9.87 min. R_t[trans-styrylacetic acid] = 1.813 min is not considered for calibration because in the reaction mixture it overlaps with the solvent front

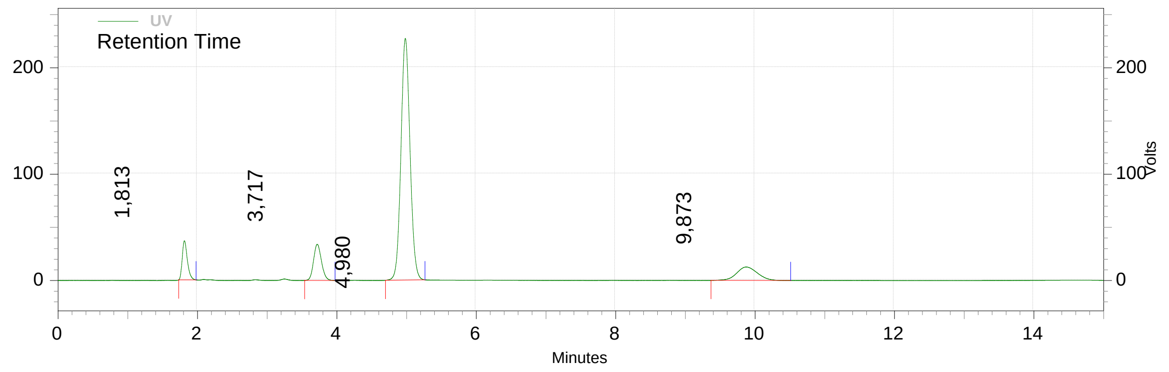

UV Results

Retention Time	Area	Area %	Height	Height %
1,813	750710	6,77	146901	11,83
3,717	1070036	9,65	136069	10,96
4,980	8213670	74,08	908291	73,14
9,873	1052977	9,50	50639	4,08
Totals	11087393	100,00	1241900	100,00

2.2. Formation of iodine(I) complexes from silver(I) complex and I_2

A bis(pyridine)chelate ligand was dissolved in CH_2Cl_2 and AgBF4 (1.0 eq.) was added. When $AgBF_4$ was dissolved, the mixture was cooled to –20°C and I_2 (1.0 eq.) was added as a CH_2Cl_2 solution. The mixture was warmed to -5°C and stirred for 1 h to complete the Ag/I exchange and AgI precipitation. Then the substrate has been added as a temperate stock solution at the desired temperature resulting in a 0.05 M substrate concentration.

The reaction mixture was analysed by HPLC to determine the *ee* of the iodolactionisation product. If the reaction mixture has been to complex, the product has been isolated by flash chromatography.

Table S3. The enantioselective Iodine(I) transfers attempted using iodine(I) complexes generated from their corresponding silver(I) complexes.

Entry	Substrate	Chiral source	solvent	T	t	Product	Yield [%] (ee [%])
1	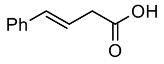		CH_2Cl_2	r.t.		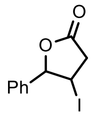	0 ee-%
2	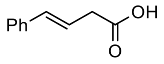		CH_2Cl_2	-20		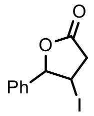	0 ee-%
3	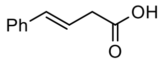		CH_2Cl_2	-20		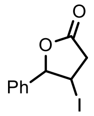	0 ee-%
4 (212/3)	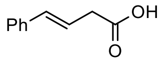	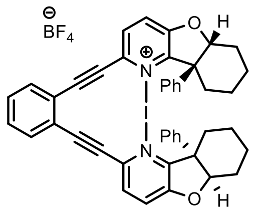	CH_2Cl_2	-20	13 h<br>60 h	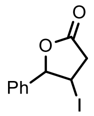	49 (1)<br>52 (1)
5 (212/3)	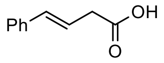		CH_3CN	-20	13 h<br>60 h	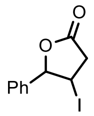	10 (0)<br>11 (2)
6 (212/3)	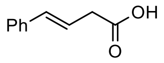		THF	-20	60 h	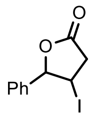	43 (-3)<br>36 (1)
7 (212/3)	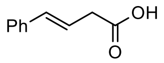		Toluene	-20	60 h	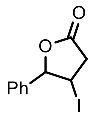	32 (1)<br>38 (3)

Table S4. Chromatogram corresponding to the reaction mixture of Table S3 entry 5, 13h.

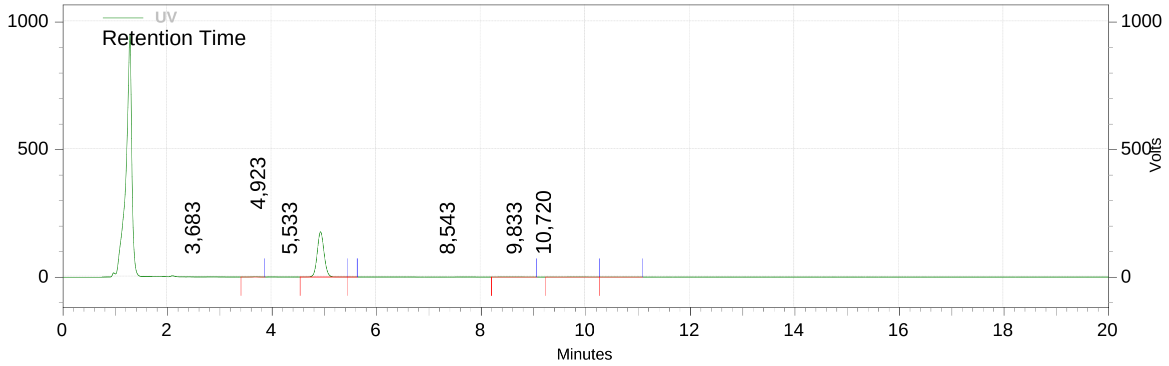

UV Results

Retention Time	Area	Area %	Height	Height %
3,683	34442	0,52	4442	0,62
4,923	6450691	98,29	709945	98,84
5,533	1606	0,02	211	0,03
8,543	30646	0,47	1472	0,20
9,833	34560	0,53	1702	0,24
10,720	11100	0,17	507	0,07
Totals	6563045	100,00	718279	100,00

Table S5. Chromatogram corresponding to the reaction mixture of Table S3 entry 5, 60h.

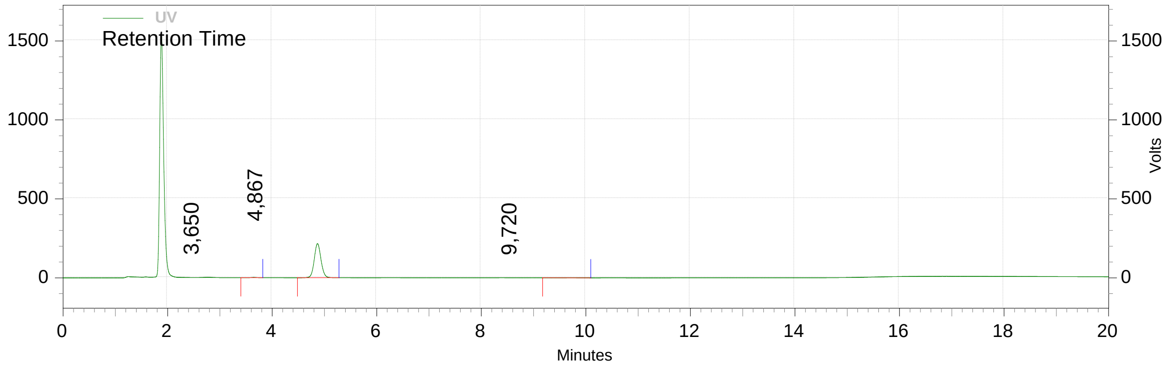

UV Results

Retention Time	Area	Area %	Height	Height %
3,650	45991	0,59	5909	0,68
4,867	7733594	98,80	863892	99,05
9,720	48062	0,61	2365	0,27
Totals	7827647	100,00	872166	100,00

Table S6. Chromatogram corresponding to the reaction mixture of Table S3 entry 4, 13h.

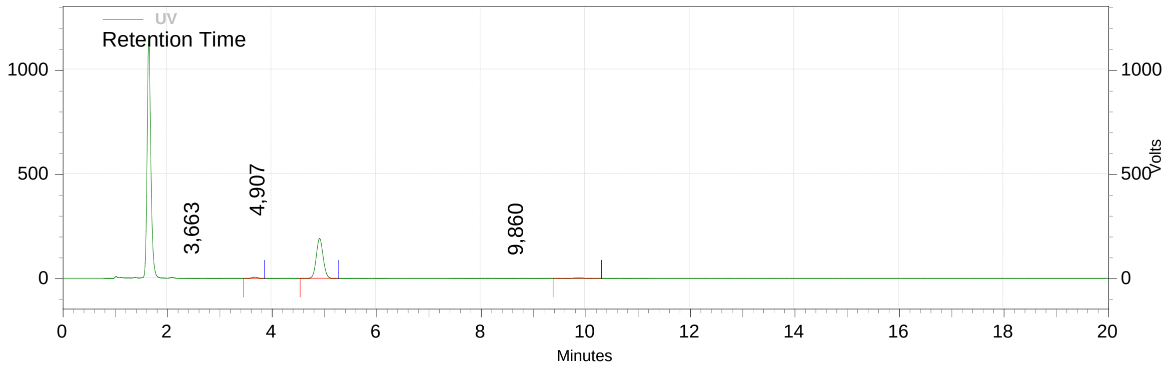

UV Results

Retention Time	Area	Area %	Height	Height %
3,663	215094	2,84	27264	3,37
4,907	7126063	94,24	771304	95,28
9,860	220766	2,92	10937	1,35
Totals	7561923	100,00	809505	100,00

Table S7. Chromatogram corresponding to the reaction mixture of Table S3 entry 4, 60h.

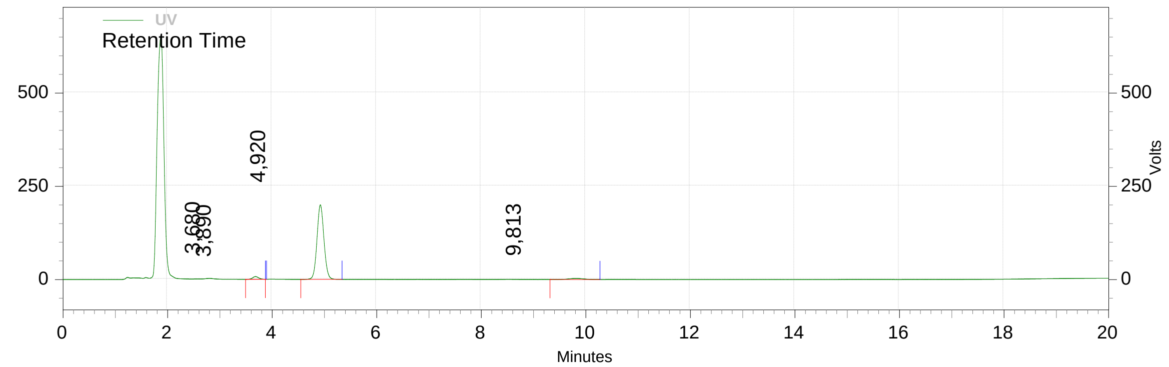

UV Results

Retention Time	Area	Area %	Height	Height %
3,680	234661	2,98	29685	3,53
3,890	81	0,00	29	0,00
4,920	7401131	93,96	800253	95,04
9,813	240805	3,06	12006	1,43
Totals	7876678	100,00	841973	100,00

Table S8. Chromatogram corresponding to the reaction mixture of Table S3 entry 6, 13h.

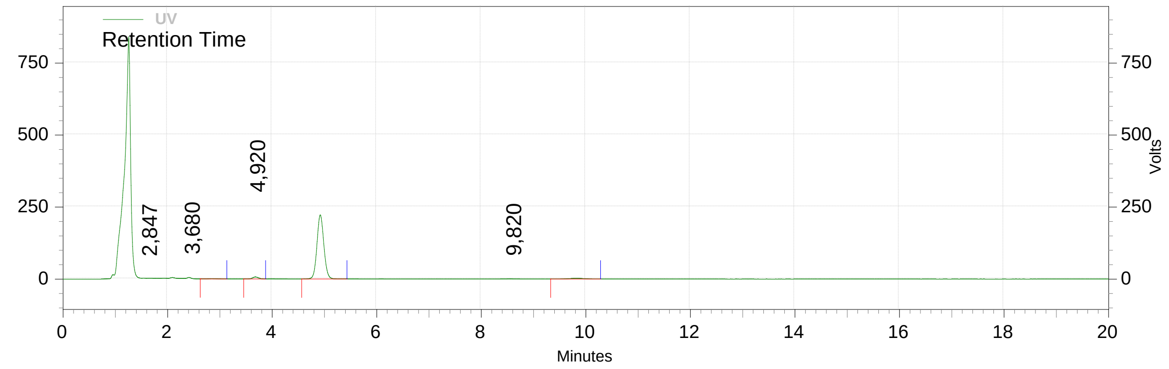

UV Results

Retention Time	Area	Area %	Height	Height %
2,847	35421	0,41	3323	0,36
3,680	221046	2,57	28253	3,03
4,920	8138404	94,63	890924	95,53
9,820	205532	2,39	10143	1,09
Totals	8600403	100,00	932643	100,00

Table S9. Chromatogram corresponding to the reaction mixture of Table S3 entry 6, 60h.

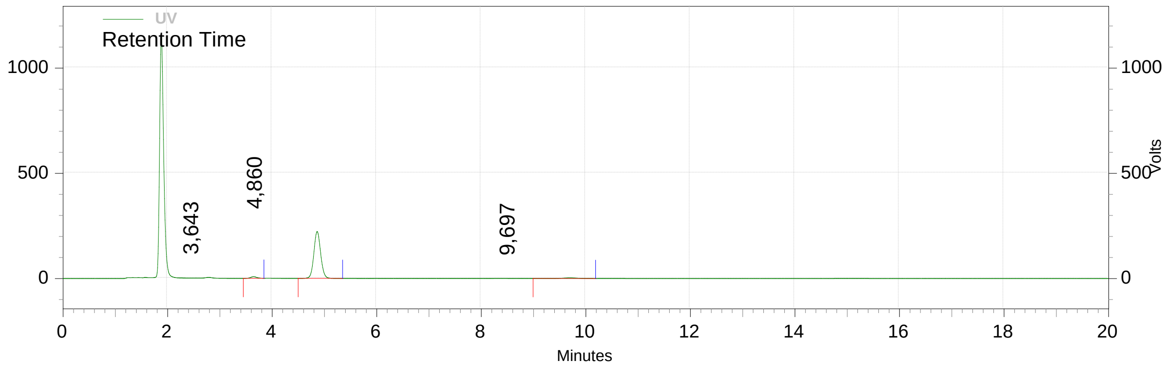

UV Results

Retention Time	Area	Area %	Height	Height %
3,643	251324	2,95	32104	3,43
4,860	8049320	94,50	892779	95,41
9,697	217073	2,55	10812	1,16
Totals	8517717	100,00	935695	100,00

Table S10. Chromatogram of the reaction mixture of Table S3 entry 7, 13 h.

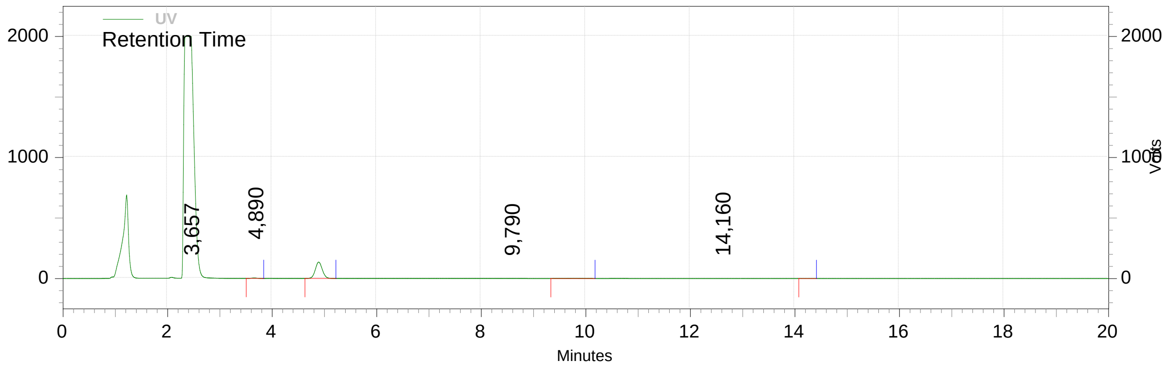

UV Results

Retention Time	Area	Area %	Height	Height %
3,657	110805	2,09	13969	2,49
4,890	5070043	95,76	541588	96,48
9,790	112864	2,13	5731	1,02
14,160	589	0,01	55	0,01
Totals	5294301	100,00	561343	100,00

Table S11. Chromatogram corresponding to the reaction mixture of Table S3 entry 7, 60h.

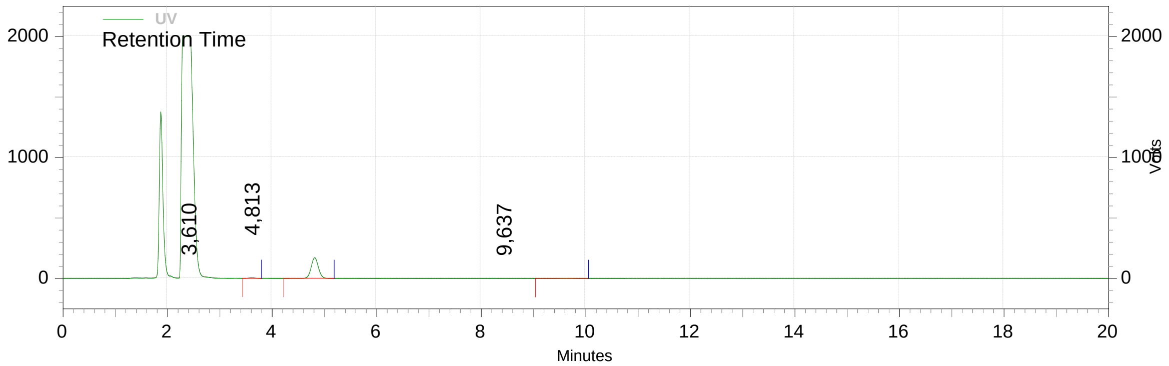

UV Results

Retention Time	Area	Area %	Height	Height %
3,610	135147	1,96	16605	2,34
4,813	6615670	95,95	686461	96,64
9,637	144284	2,09	7255	1,02
Totals	6895101	100,00	710321	100,00

Iodolactonization of 5-phenylhex-5-enoic acid using catalyst (7R,7'R)-(1,2-phenylenebis(ethyne-2,1-diyl))bis(4-methyl-6,7-dihydro-5H-cyclopenta[b]pyridine-2,7-diyl) diacetate (2)

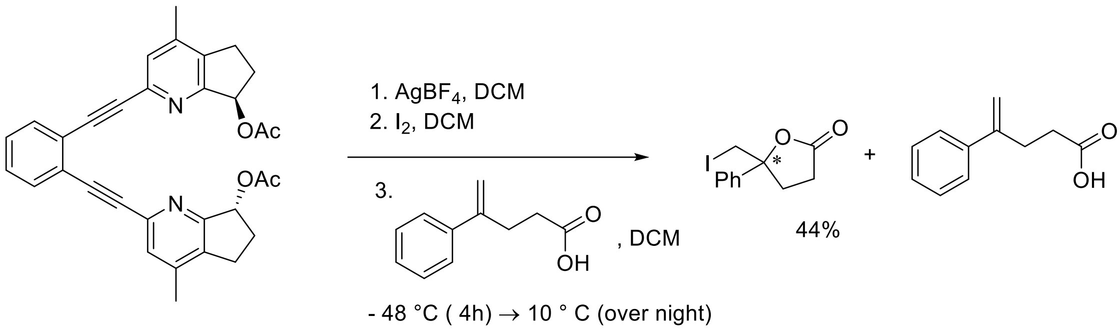

2

To an oven-dried microwave vial, $AgBF_4$ (22 mg, 0.11 mmol) was added in the dark (by wrapping the vial with aluminum foil) under N_2 atmosphere. The vessel was sealed and filled with N_2. A solution of compound (*S*)-**2** (53 mg, 0.10 mmol) in dry,freshly distilled CH_2Cl_2 (2.0 mL) was added and the solution was stirred for 2 h at rt. A color change from pale orange to dark brown was observed. Thereafter, a solution of I_2 (27 mg, 0.105 mmol) in dry and freshly distilled CH_2Cl_2 (5.0 mL) was added by cannula. Immediately, a light yellow precipitate was formed (AgI). The reaction mixture was stirred for 1 h at r.t. under N_2 atmosphere. Then, the mixture was centrifuged at 2000 ppm for 10 min. The supernatant was removed and transferred by cannula to an oven-dry microwave vial, under N_2. Precipitation of the [bis(pyridine)iodine(I)] tetrafluoroborate salt was carried out by addition of dry and freshly distilled hexane. After centrifugation at 2000 ppm for 10 min, the supernatant was removed by cannula and the yellow precipitate dried under high vacuum for 30 min. The vial was filled with N_2, and the solid was re-dissolved in dry and freshly distilled CH_2Cl_2 (1 mL). The vial was cooled to -48˚C, and a solution of 5-phenylhex-5-enoic acid (18 mg, 0.10 mmol) in dry and freshly distilled CH_2Cl_2 (1 mL) was added dropwise via a syringe. The solution was stirred for 4 h at - 48 ˚C and then allowed to warm to 10 ˚C overnight. The reaction was then quenched with $Na_2S_2O_3$ and extracted with CH_2Cl_2. The combined organic extracts were dried over Na_2SO_4, filtered, concentrated in vacuo, and purified by column chromatography (SiO_2, EtOAc:hexane 4:6) to provide 14 mg of 5-(iodomethyl)-5-phenyldihydrofuran-2(*3H*)-one (44 %) as a yellow oil along with starting material.

The enantiomeric purity of this compound was analyzed by analytical chiral HPLC using a Daicel Chiralpack AD column (250 x 4.6mm x 10um) [hexane:isopropanol (95:5), flow rate at 1 mL/min, detection at λ = 240 nm; injection= 10 µL (3 mg of **8** in 1mL (hexane:IPA 7:3); t_1 = 11.5 min, t_2 = 13,05 min, 0 % *ee*].

Area % Report

Data File: C:\Documents and Settings\standard user\Mina dokument\Data-EZchrom-reports\Inma\irl198-6.dat
Method: C:\EZChrom Elite\Enterprise\Projects\Standard\Method\User methods\Inma\chiralpack-AD.met
Acquired: 2017-04-17 16:00:17
Printed: 2017-04-17 16:31:46

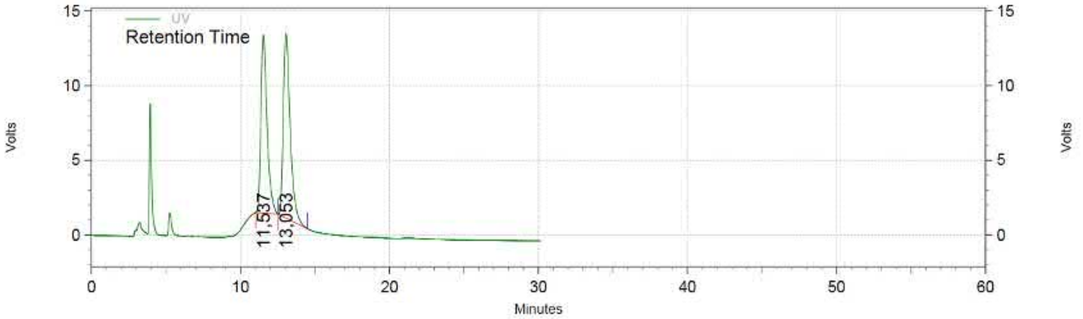

UV Results

Retention Time	Area	Area %	Height	Height %
11,537	1373062	46,31	47754	49,16
13,053	1591962	53,69	49394	50,84
Totals	2965024	100,00	97148	100,00

Figure S12. Chiral HPOLC chromatogram of the 2-I mediated halocyclisation, showning negligible ee.

2.3. Formation of iodine(I) complexes from pyridinium ligand and NIS

The bis(pyridine) ligand (0.10 eq.) was dissolved in the desired solvent and HOTf or $HNTf_2$ (0.08 eq.) was added. The mixture was warmed/cooled to the desired temperature. Next *N*-iodsuccinimide (1.2 eq.) as well as the temperate substrate stock solution (1.0 eq., resulting in a 0.05 M solution) was added. The reaction mixture was analysed by HPLC to determine the *ee* of the iodolactionisation product. If the reaction reached completion, the product was isolated by flash chromatography. The observed *ee* is independent of the reaction time when the sample is taken (shown in Entry 5). The time independence of ee-% was reported by Johnston and co-workers.[3]

Table S12. Enantioselective iodine(I) transfer using NIS and acid, and a chiral catalyst.

Entry	*Substrate*	*Chiral source*	*solvent*	*T*	*t*	*Product*	*ee*
1 (229)	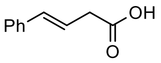	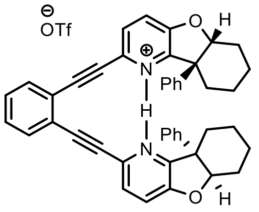	*toluene*	*-20*	*24 h*	*Traces,* 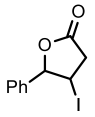	*9 %* *(yield <1%)*

2 (229)	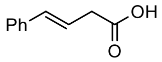	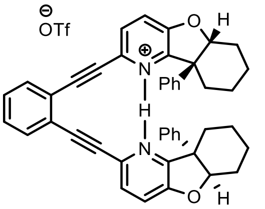	*toluene*	*r.t.*	*24 h*	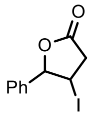	*2 %*<br>*(Yield 98%)*
3 (302)	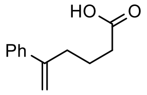	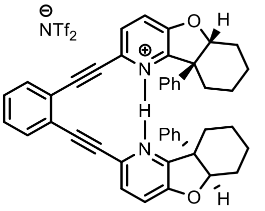	*toluene*	*-20*	*72*	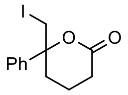	*<7 %*
4 (300)	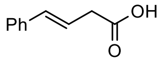	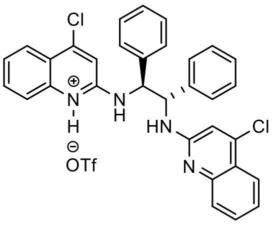	*toluene*	*-20*	*22 h*	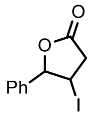	*31 %*
5 (301)	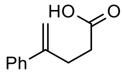	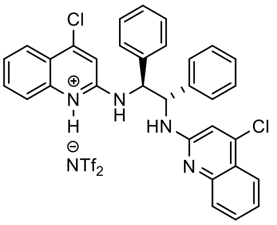	*toluene*	*-20*	*22 h*<br>*72 h*	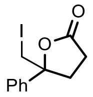	*53 %*<br>*53 %*
6 (296)	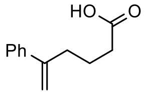	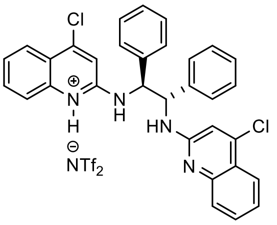	*toluene*	*-20*	*72 h*	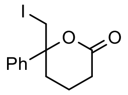	*61 %*
7	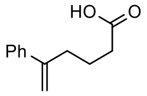	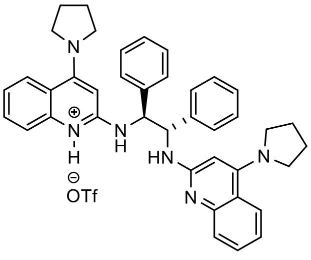	*toluene*	*-20*	*22h*	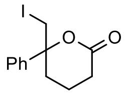	*93 %*
8 (286)	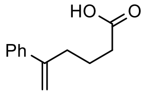	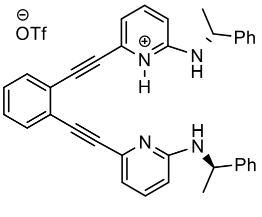	*toluene*	*-20*	*22h*	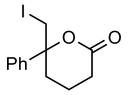	*3%*

Table S13. Chromatogram corresponding to the reaction mixture of Table S12 entry 1.

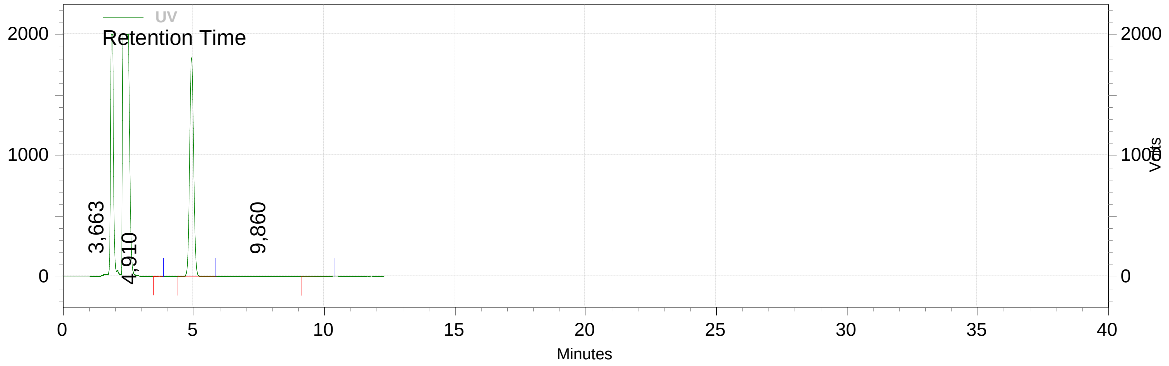

UV Results

Retention Time	*Area*	*Area %*	*Height*	*Height %*
3,663	*195720*	*0,25*	*22406*	*0,31*
4,910	*77272818*	*99,46*	*7237313*	*99,55*
9,860	*224061*	*0,29*	*10297*	*0,14*
Totals	*77692599*	*100,00*	*7270016*	*100,00*

Table S14. Chromatogram corresponding to the reaction mixture of Table S12 entry 2.

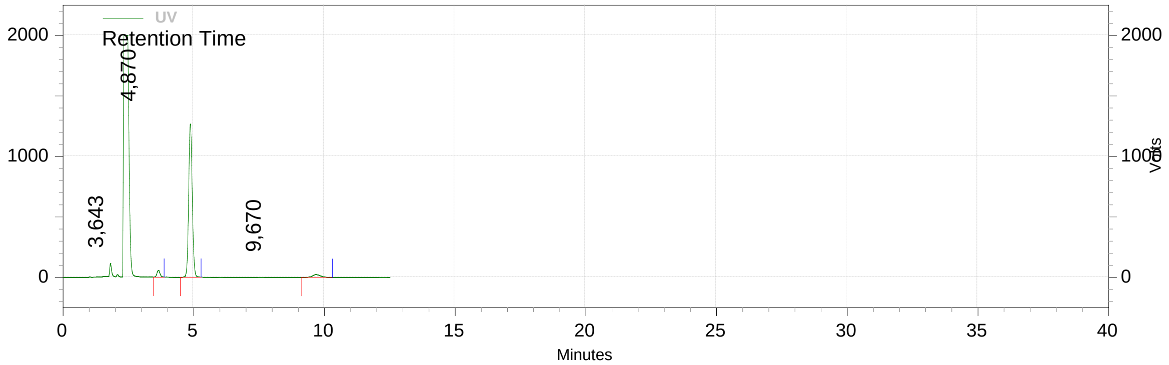

UV Results

Retention Time	*Area*	*Area %*	*Height*	*Height %*
3,643	*1848193*	*3,57*	*226762*	*4,21*
4,870	*48047169*	*92,73*	*5065986*	*94,08*
9,670	*1919805*	*3,71*	*91973*	*1,71*
Totals				

	51815167	*100,00*	*5384721*	*100,00*

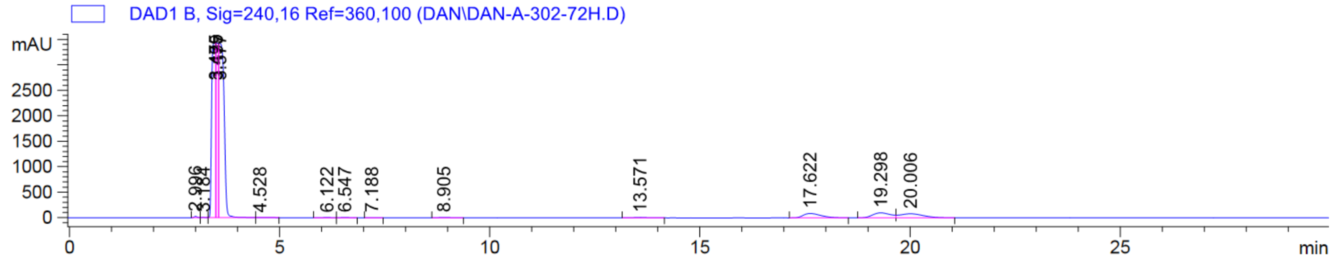

Figure S13. Chromatogram corresponding to the reaction mixture of Table S12 entry 3, different HPLC instrument.

Table S15. Chromatogram corresponding to the reaction mixture of Table S12 entry 4, 22h.

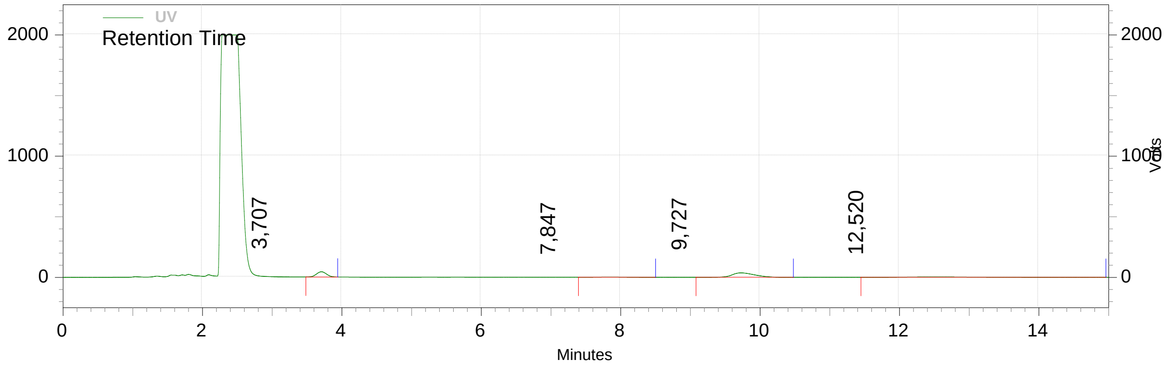

UV Results

Retention Time	*Area*	*Area %*	*Height*	*Height %*
3,707	*1771603*	*28,88*	*174993*	*51,40*
7,847	*160456*	*2,62*	*6154*	*1,81*
9,727	*3341503*	*54,47*	*147581*	*43,35*
12,520	*860969*	*14,03*	*11723*	*3,44*
Totals				

Table S16. Chromatogram corresponding to the reaction mixture of Table S12 entry 5, 22h.

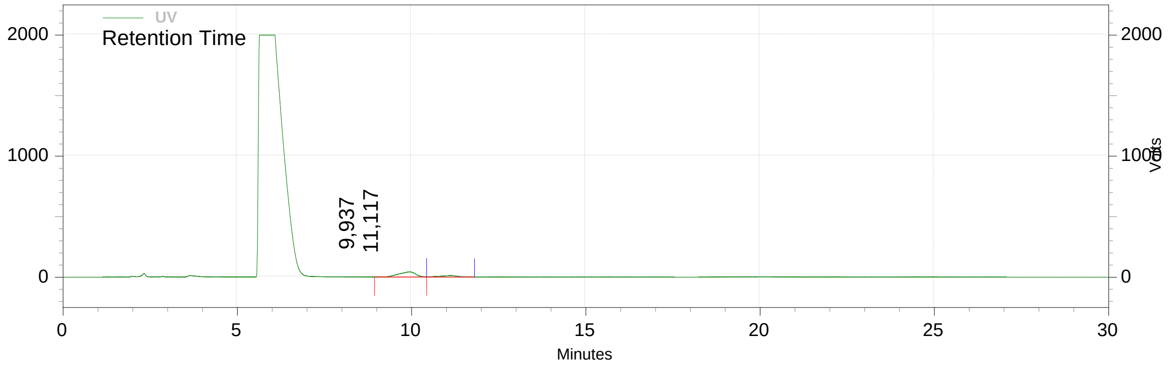

UV Results

Retention Time	*Area*	*Area %*	*Height*	*Height %*
9,937	*5834044*	*76,73*	*172618*	*77,52*
11,117	*1768951*	*23,27*	*50067*	*22,48*
Totals	*7602995*	*100,00*	*222685*	*100,00*

Table S17. Chromatogram corresponding to the reaction mixture of Table S S12 entry 5, 72h.

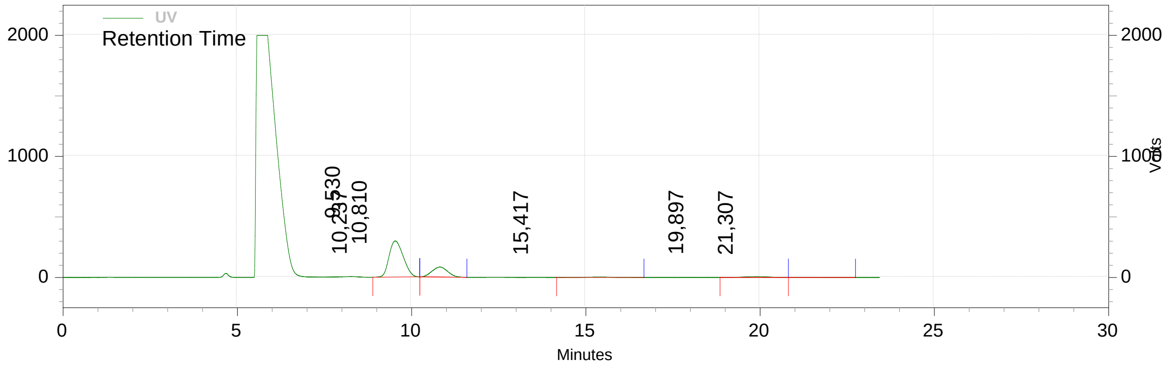

UV Results

Retention Time	*Area*	*Area %*	*Height*	*Height %*
9,530	*33686441*	*73,81*	*1192309*	*76,55*
10,237	*0*	*0,00*	*0*	*0,00*
10,810	*10400194*	*22,79*	*331139*	*21,26*
15,417	*462654*	*1,01*	*9738*	*0,63*

19,897	*1044235*	*2,29*	*23209*	*1,49*
21,307	*48892*	*0,11*	*1139*	*0,07*
Totals	*45642416*	*100,00*	*1557534*	*100,00*

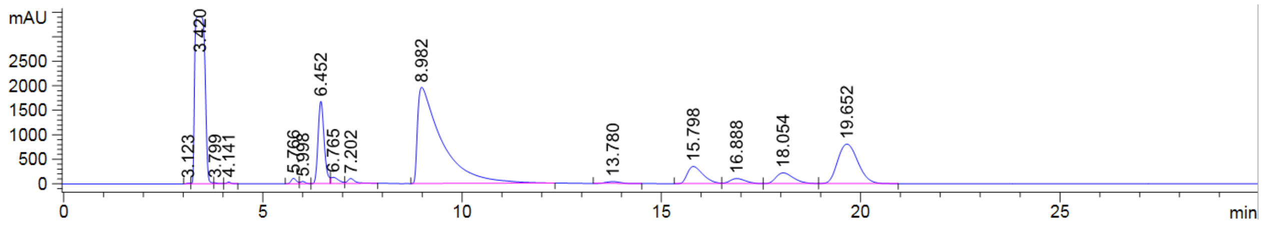

Figure S13. Chromatogram corresponding to the reaction mixture of Table SX entry 6 72h, different HPLC instrument.

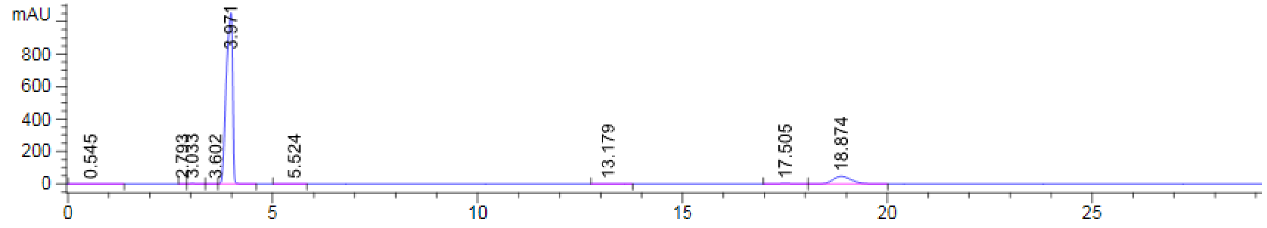

Figure S14. Chromatogram corresponding to the reaction mixture of Table SX entry 7 22h, different HPLC instrument.

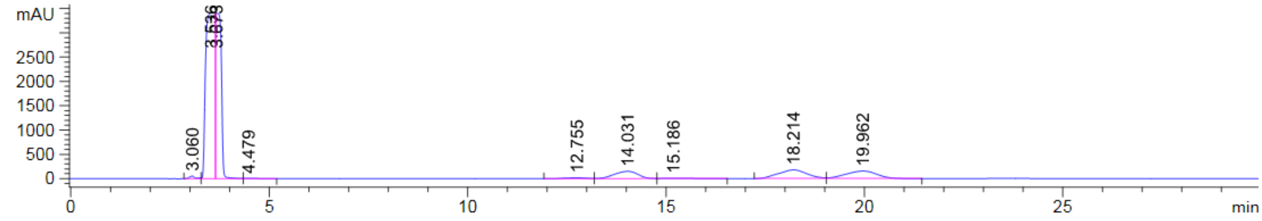

Figure S15. Chromatogram corresponding to the reaction mixture of Table SX entry 8 22h, different HPLC instrument.

2.4. Enantioselective iodoetherification – norbornene-2.3-dimethanol desymmetrisation

Iodoetherification was performed in 4h at -20°C using iodine(I) complex generated from silver(I) complex, and the reaction progress was monitored by NMR. When no reaction progress was detected anymore, the product was isolated by flash column chromatography (2.6 mg, 93%).

The product was dissolved in CH_2Cl_2 (0.700 mL), and diisopropylamine (61 µL, 0.35 mmol) and benzoic anhydride (79 mg, 0.35 mmol) were added. The mixture was stirred for 3d at 50°C and was stopped after observing full conversion of the starting material to the product by NMR. The mixture was then analysed by HPLC indicating 2 ee-%.

The post-modification conditions using benzoic acid and diisopropylethylamine were optimized in an independent reaction, without detecting any side-reactions or deiodination products in NMR.

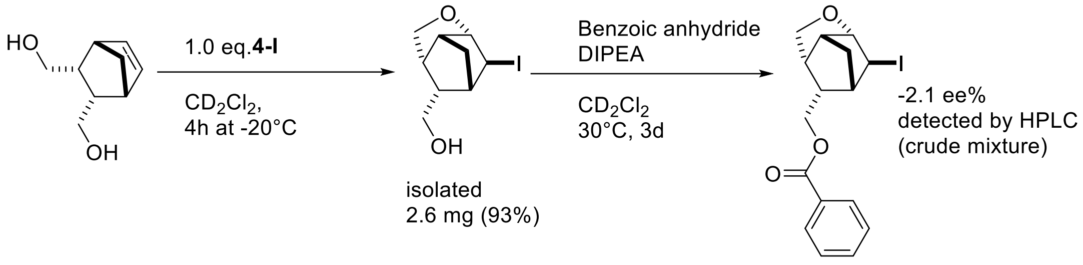

Figure S16. Desymmetric iodoetherification and post-modification of norbornene-2.3-dimethanol using **4**-I.

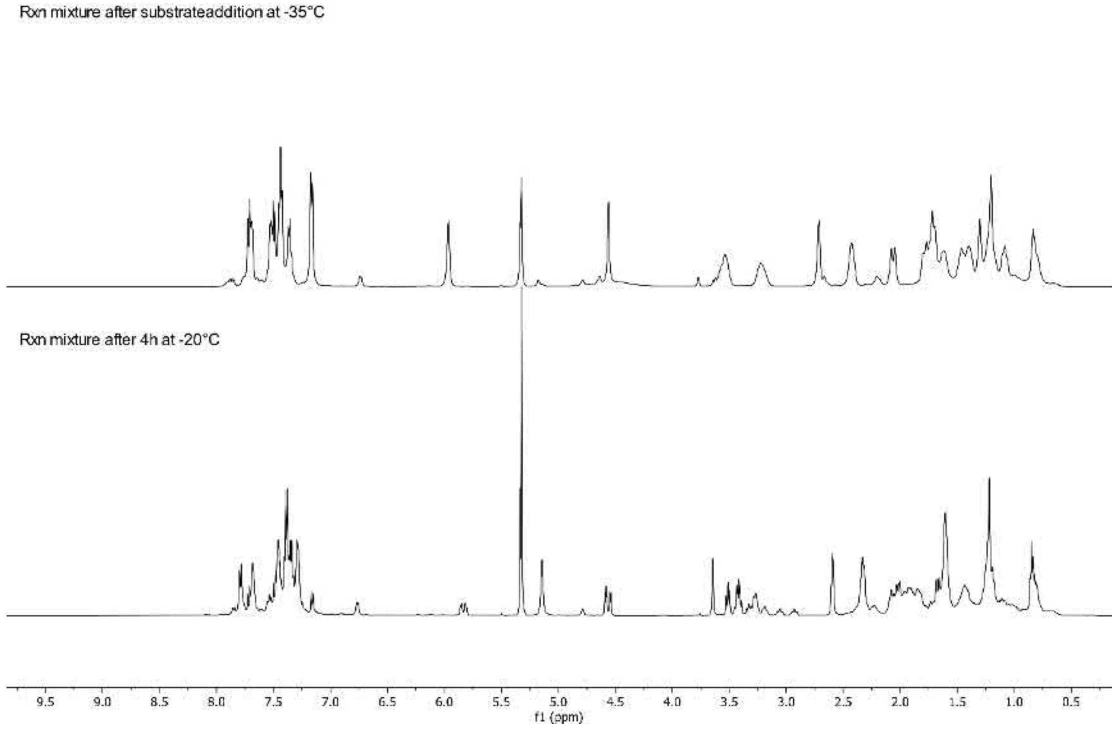

Figure S17. ^{1}H NMR spectra of the iodetherfication reaction mixture on substrate addition (top) and after 4h at -20°C (bottom).

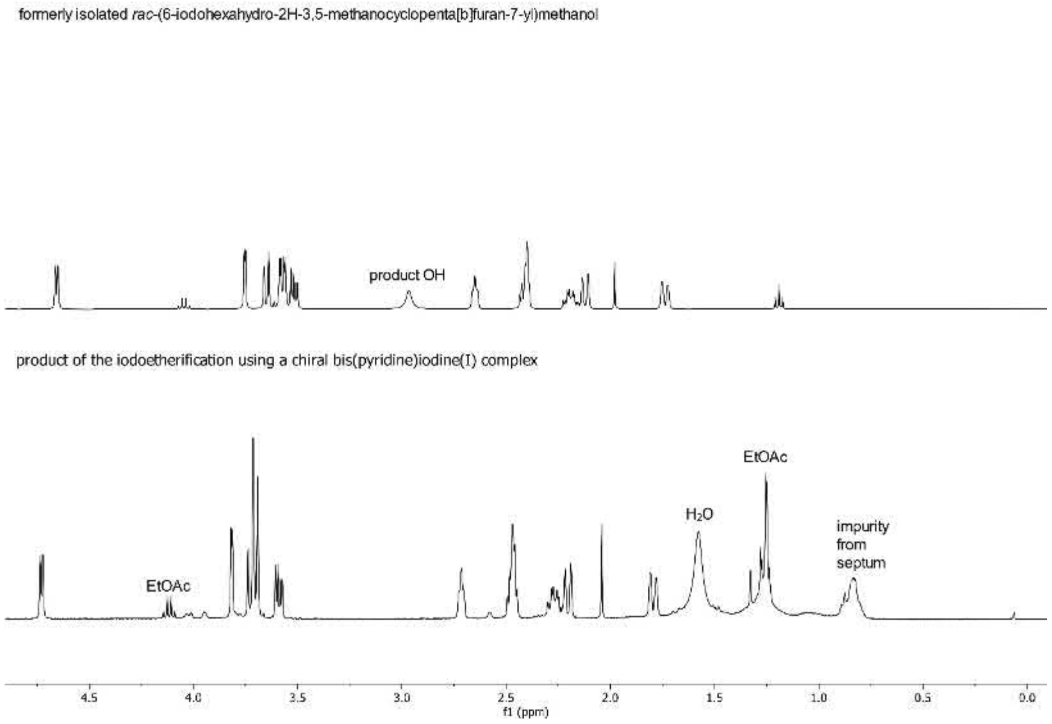

Figure S18. Comparison of the *rac*-(6-iodohexahydro-2*H*-3,5-methanocyclopenta[*b*]furan-7-yl)methanol reference sample (top) and the product isolated after iodoetherification in the presence of **4-I**. Mismatches in the ^{1}H-NMR spectra are observed for OH protons, due to slightly different water content.

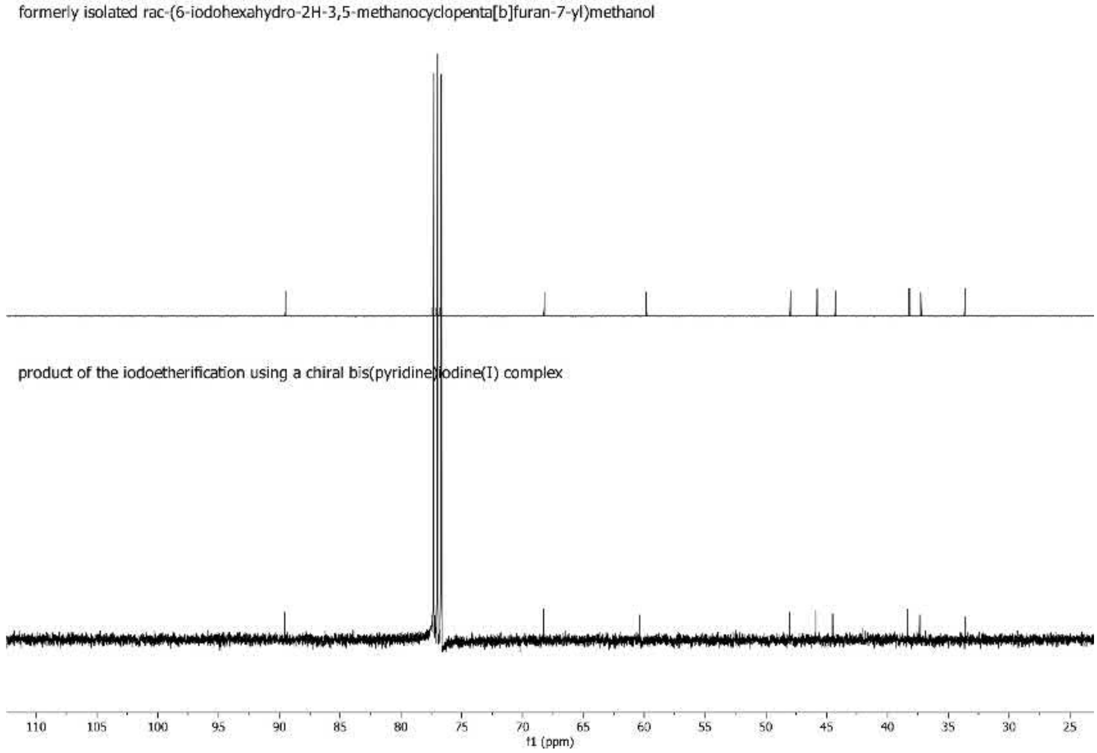

Figure S19. The *rac*-(6-iodohexahydro-2*H*-3,5-methanocyclopenta[*b*]furan-7-yl)methanol ^{13}C NMR spectrum (top), and the product (bottom) isolated after iodetherification in the presence of **4-I**. The ^{13}C NMR spectra shows perfect alignment, and the presence of the C–I carbon atom at 33.6 ppm.

Formation of the benzylic ester is difficult to follow by NMR because of chemical shift overlaps. It is best indicated by the simultaneous observation of two doublets in the ^{1}H NMR spectrum at 4.7-4.8 ppm, where the doublet with lower chemical shift belongs to the 6a–H proton of the *rac*-(6-iodohexahydro-2*H*-3,5-methanocyclopenta[*b*]furan-7-yl)methanol starting material, and the one with higher chemical shift belongs to the benzylic acid esterification product.

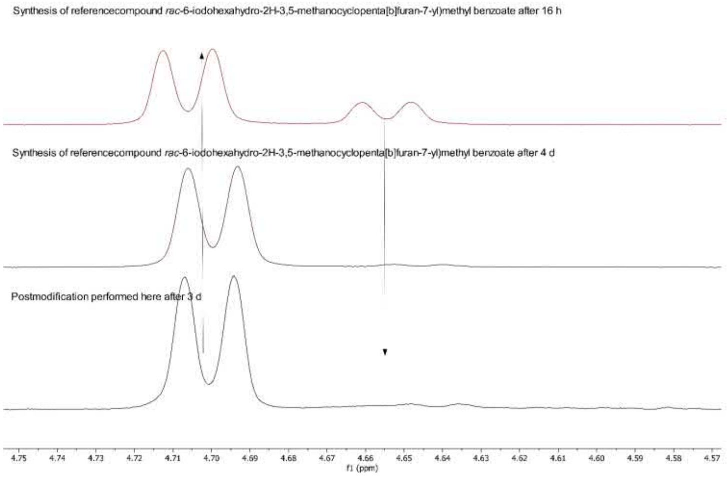

Figure S20. Indicative ^{1}H NMR signals to monitor the benzoic acid ester formation.

Table S18. HPLC chromatogram of the post-modification reaction mixture resulting in 6-iodohexa-hydro-2H-3,5-methanocyclopenta[b]furan-7-yl)methyl benzoate. The enantiomeric access observed was 2.1 ee-%.

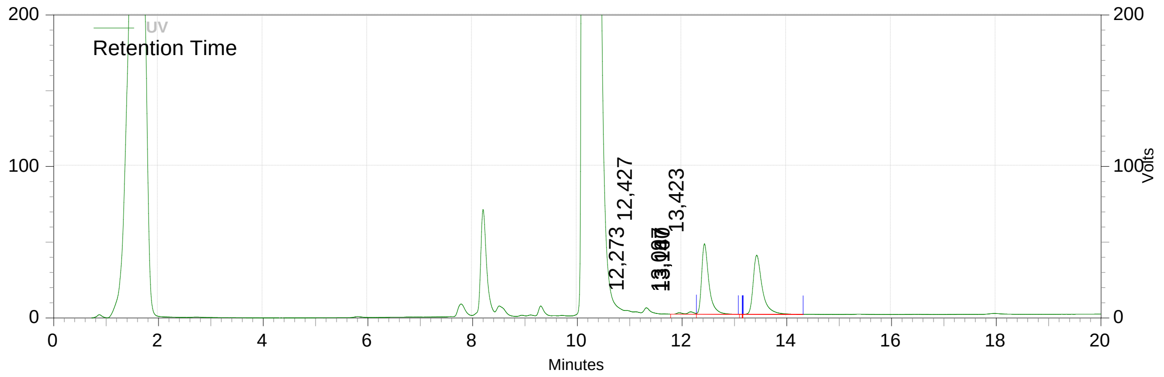

UV Results

Retention Time	Area	Area %	Height	Height %
12,273	73417	1,99	2016	0,59
**12,427**	1773181	47,98	185915	54,03
13,097	263	0,01	125	0,04
13,147	7	0,00	11	0,00
13,147	7	0,00	11	0,00
13,160	15	0,00	28	0,01
**13,423**	1849036	50,03	155969	45,33
Totals	3695926	100,00	344075	100,00

3. Spectra

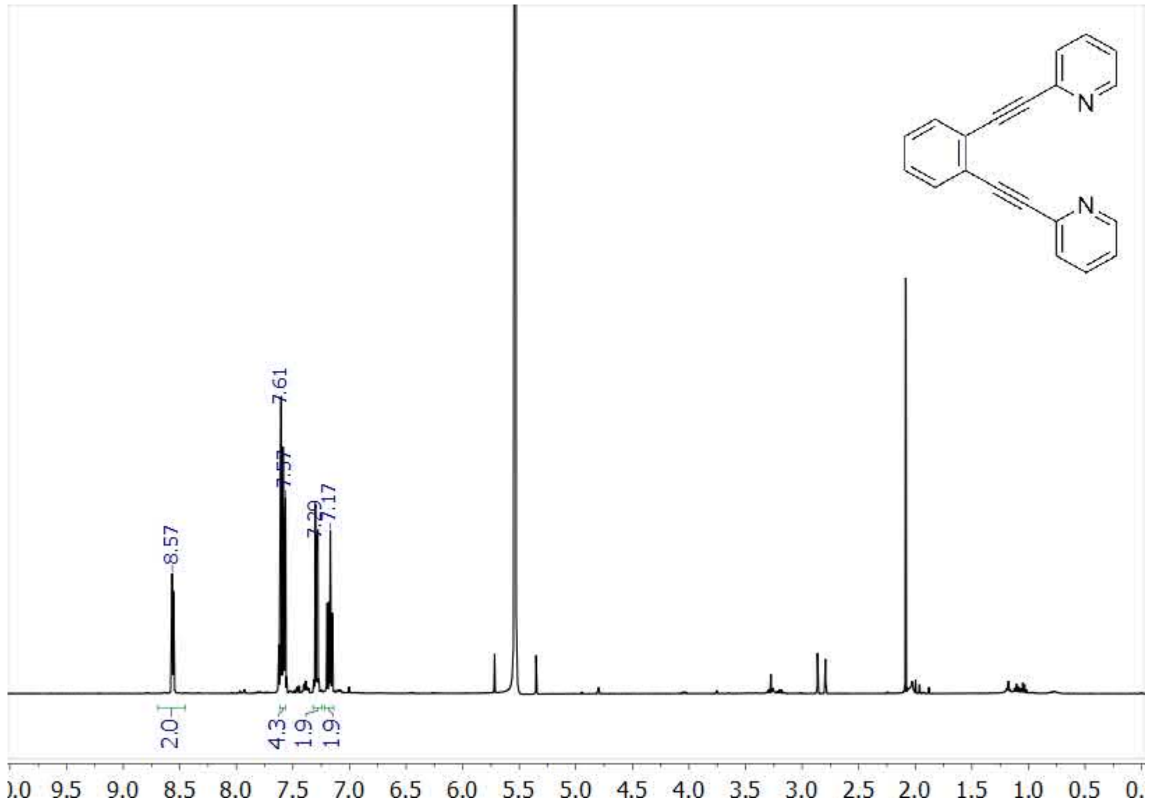

Figure S21. ^{1}H NMR spectrum of (1,2-bis(pyridin-2-ylethynyl)benzene) (**1**) in CD_2Cl_2 at 25℃ (399.9 MHz).

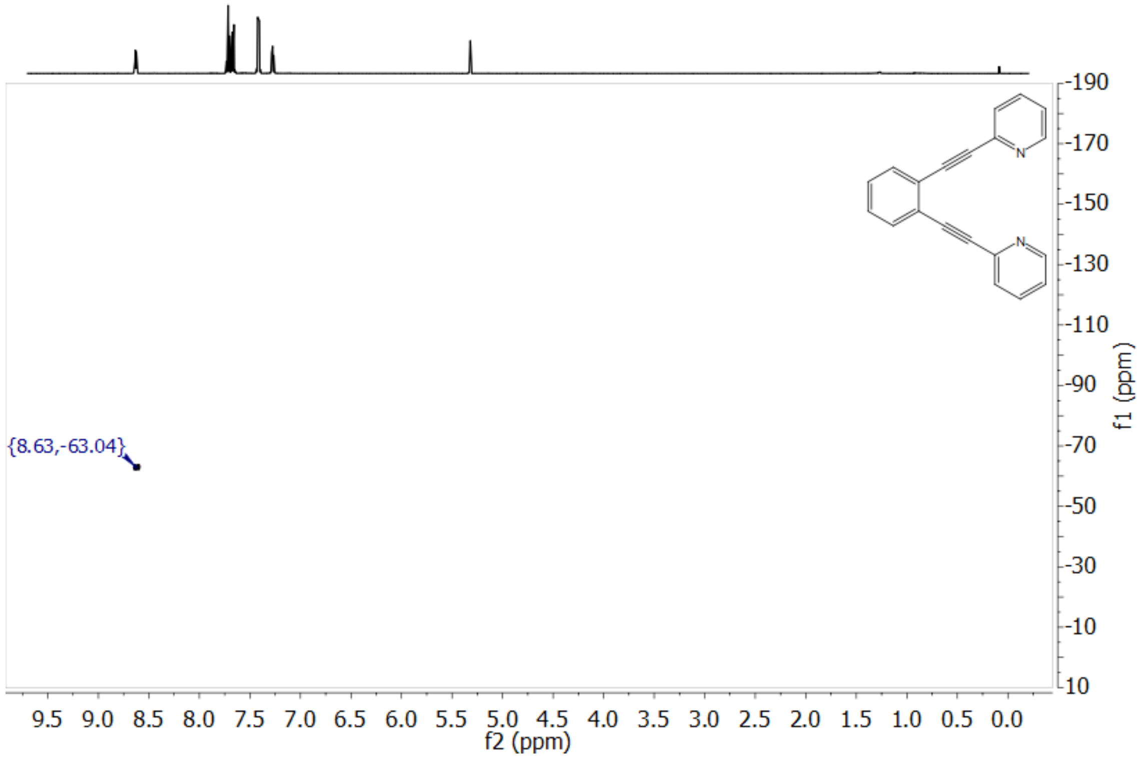

Figure S22. $^{1}H,^{15}N$ HMBC spectrum of (1,2-bis(pyridin-2-ylethynyl)benzene) (**1**) in CD_2Cl_2 at 25°C (499.9/50.66 MHz).

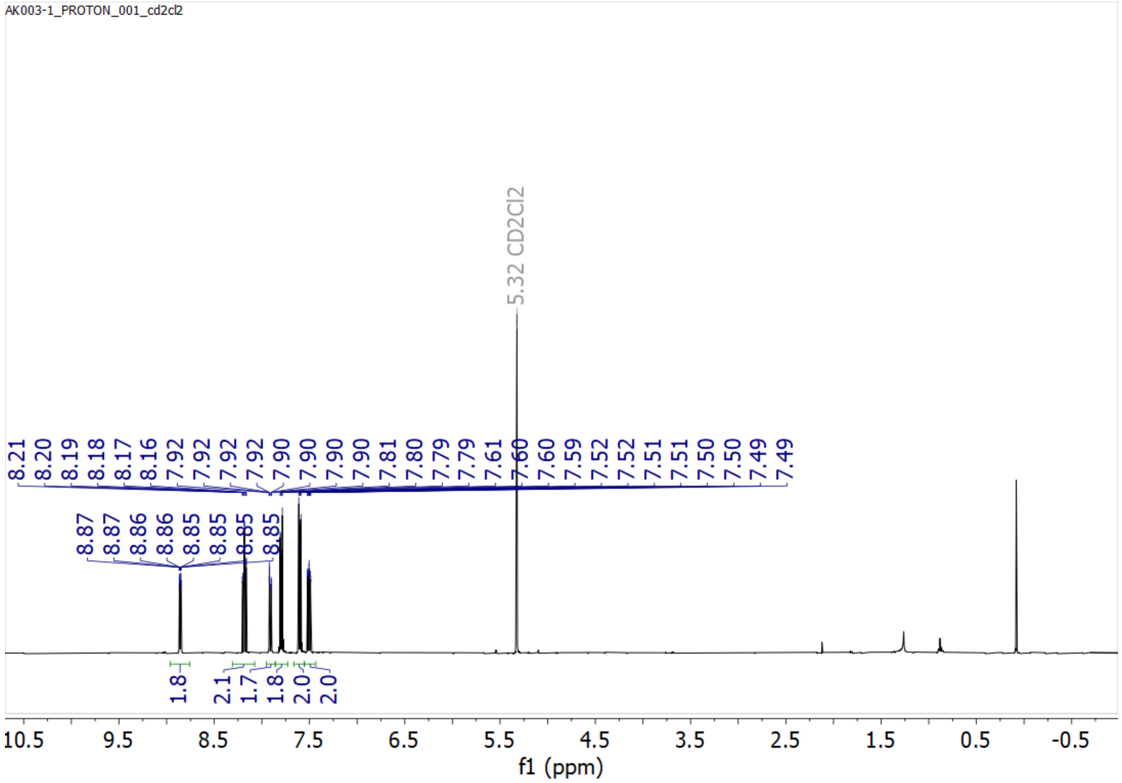

Figure S23. ^{1}H NMR spectrum of [(1,2-bis(pyridin-2-ylethynyl)benzene)iodine(I)] tetrafluoroborate (**1-I**) in CD_2Cl_2 at 25°C (399.9 MHz).

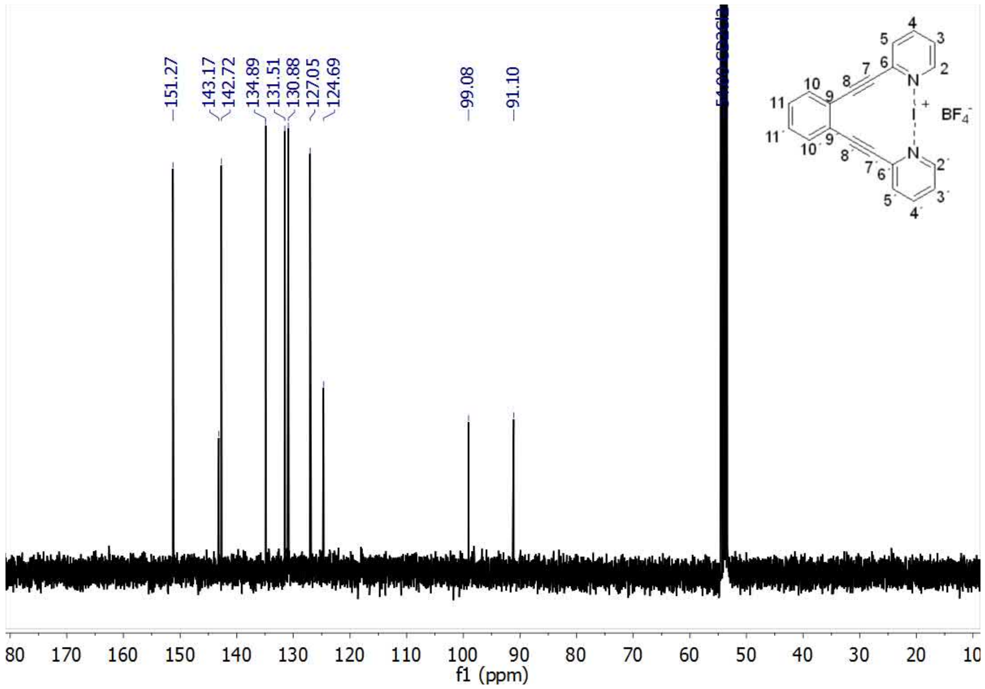

Figure S24. ^{13}C NMR spectrum of [(1,2-bis(pyridin-2-ylethynyl)benzene)iodine(I)] tetrafluoroborate (**1-I**) CD_2Cl_2 at 25°C (125.7 MHz).

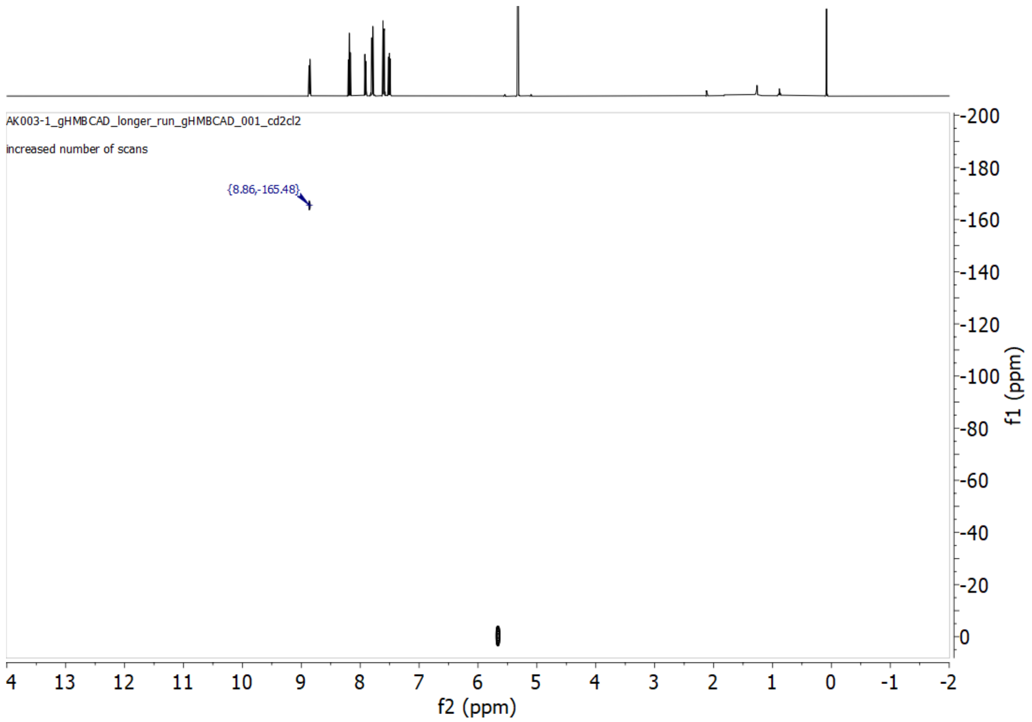

Figure S25. $^{1}H,^{15}N$ HMBC spectrum of [(1,2-bis(pyridin-2-ylethynyl)benzene)iodine(I)] tetrafluoroborate (**1-I**) in CD_2Cl_2 at 25°C (399.9/40.54 MHz).

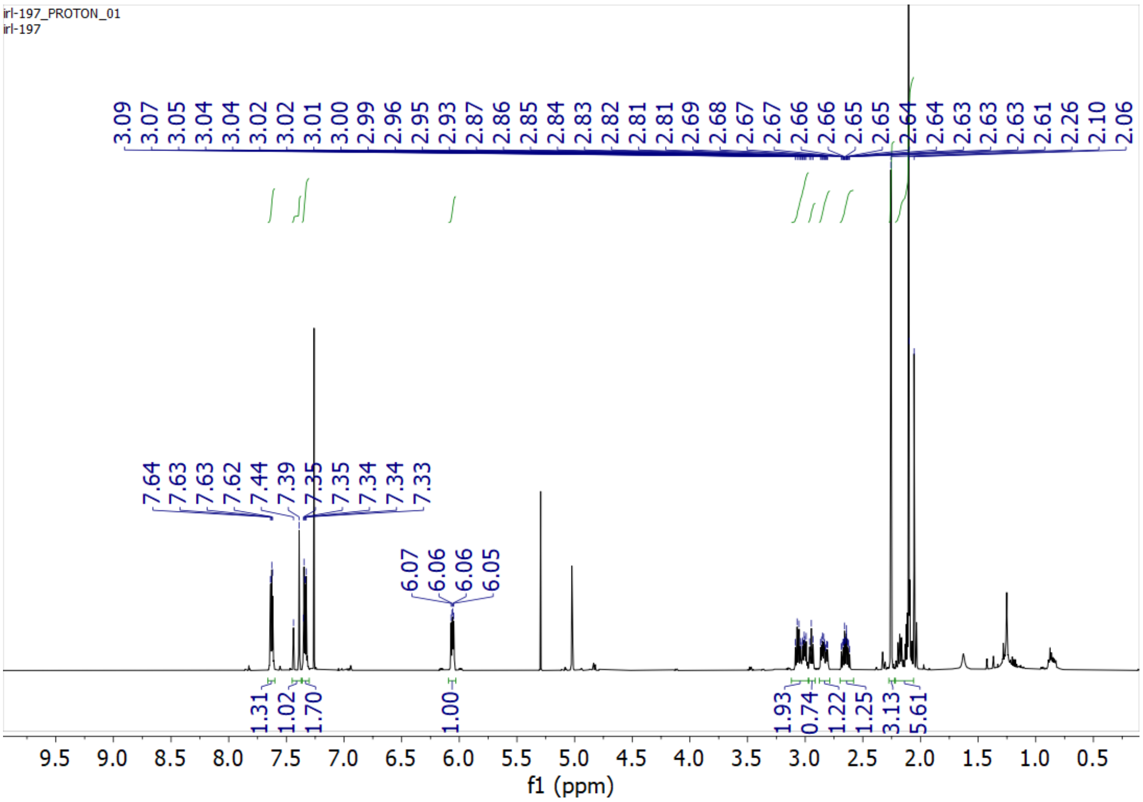

Figure S26. ^{1}H NMR spectrum of (7*R*,7'*R*)-(1,2-phenylenebis(ethyne-2,1-diyl))bis(4-methyl-6,7-dihydro-5H-cyclopenta[b]pyridine-2,7-diyl) diacetate (**2**) in $CDCl_3$ at 25°C (499.9 MHz).

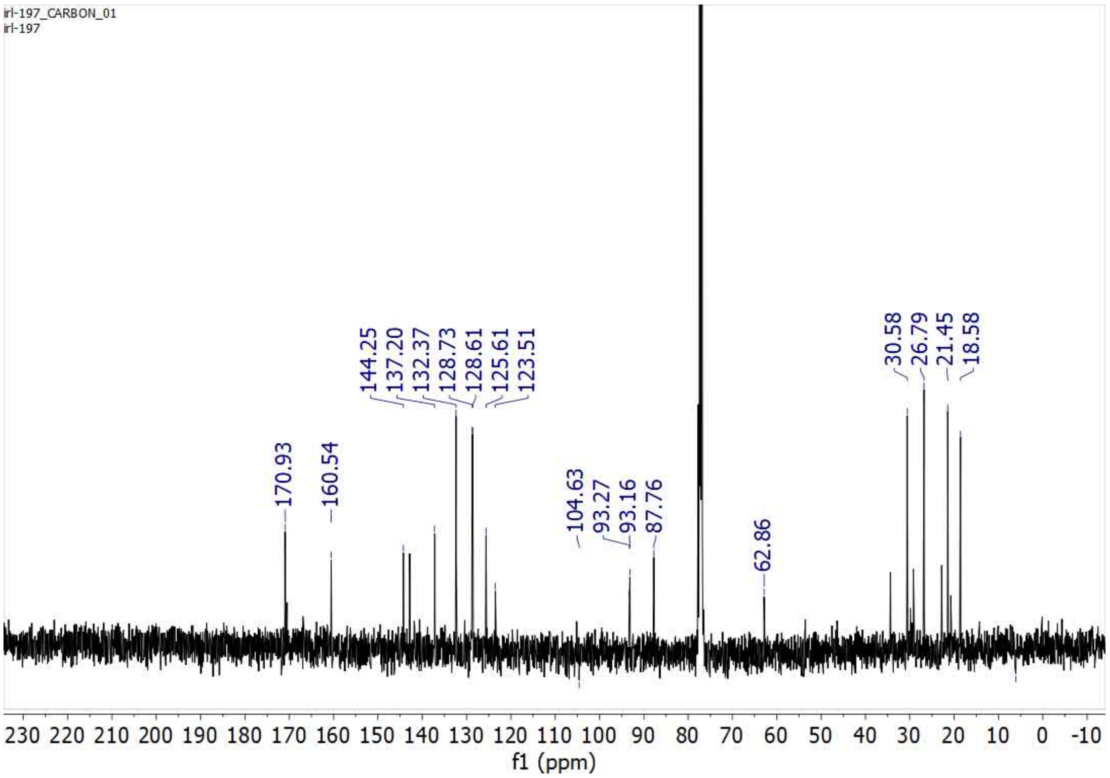

Figure S27. ^{13}C NMR spectrum of (7*R*,7'*R*)-(1,2-phenylenebis(ethyne-2,1-diyl))bis(4-methyl-6,7-dihydro-5H-cyclopenta[b]pyridine-2,7-diyl) diacetate (**2**) in $CDCl_3$ at 25°C (125.7 MHz).

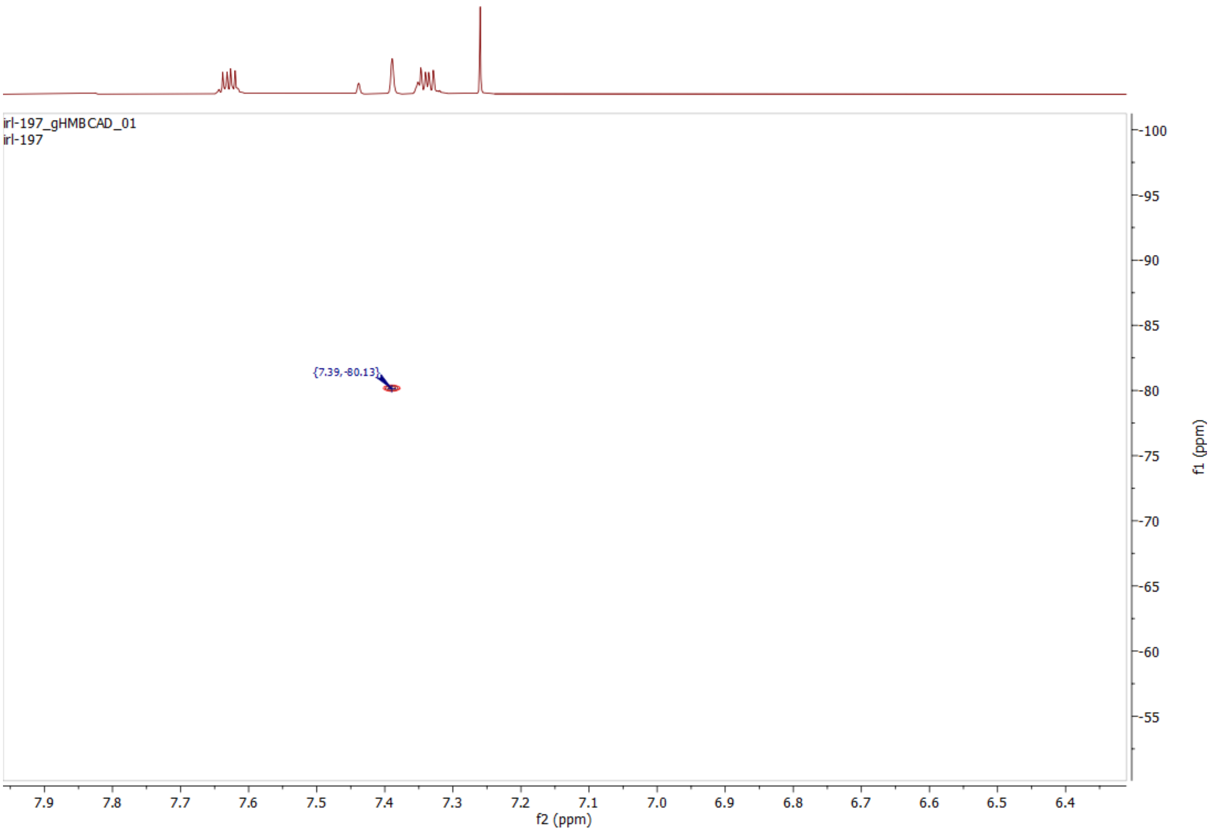

Figure S28. ^{1}H,^{15}N HMBC spectrum of (7*R*,7'*R*)-(1,2-phenylenebis(ethyne-2,1-diyl))bis(4-methyl-6,7-dihydro-5H-cyclopenta[b]pyridine-2,7-diyl) diacetate (**2**) in $CDCl_3$ at 25°C (499.9/50.66 MHz).

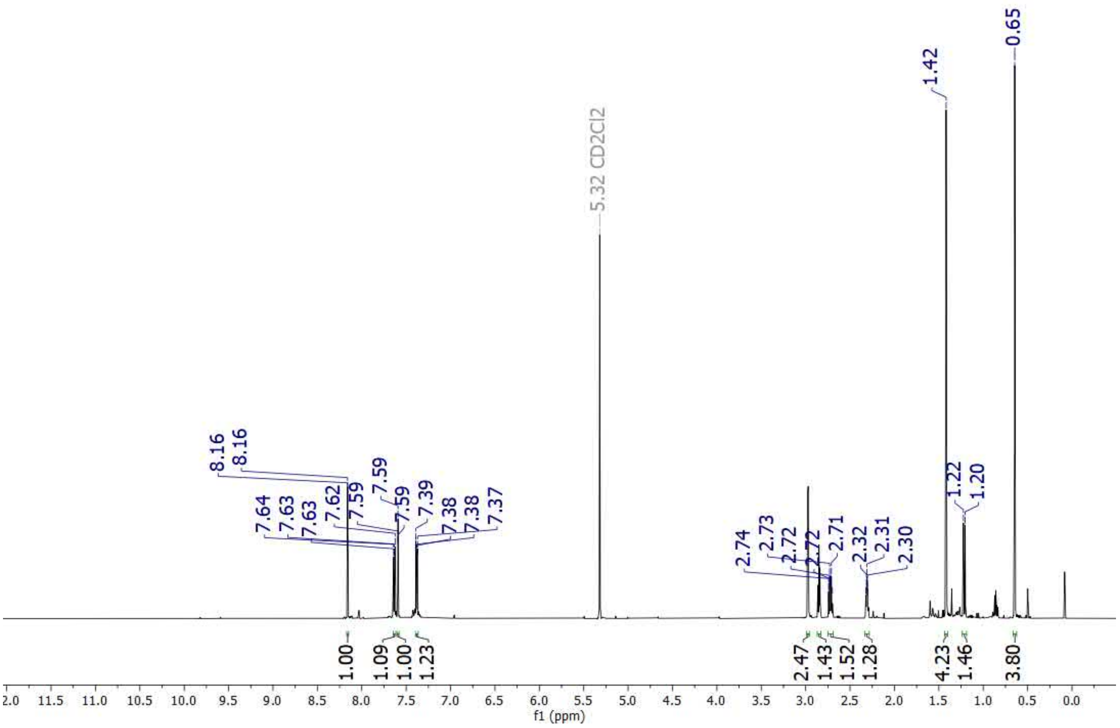

Figure S29. ^{1}H NMR spectrum of 1,2-bis(((6*R*,8*R*)-7,7-dimethyl-5,6,7,8-tetrahydro-6,8-methanoisoquinolin-3-yl)ethynyl)benzene (**3**) in CD_2Cl_2 at 25°C (499.9 MHz).

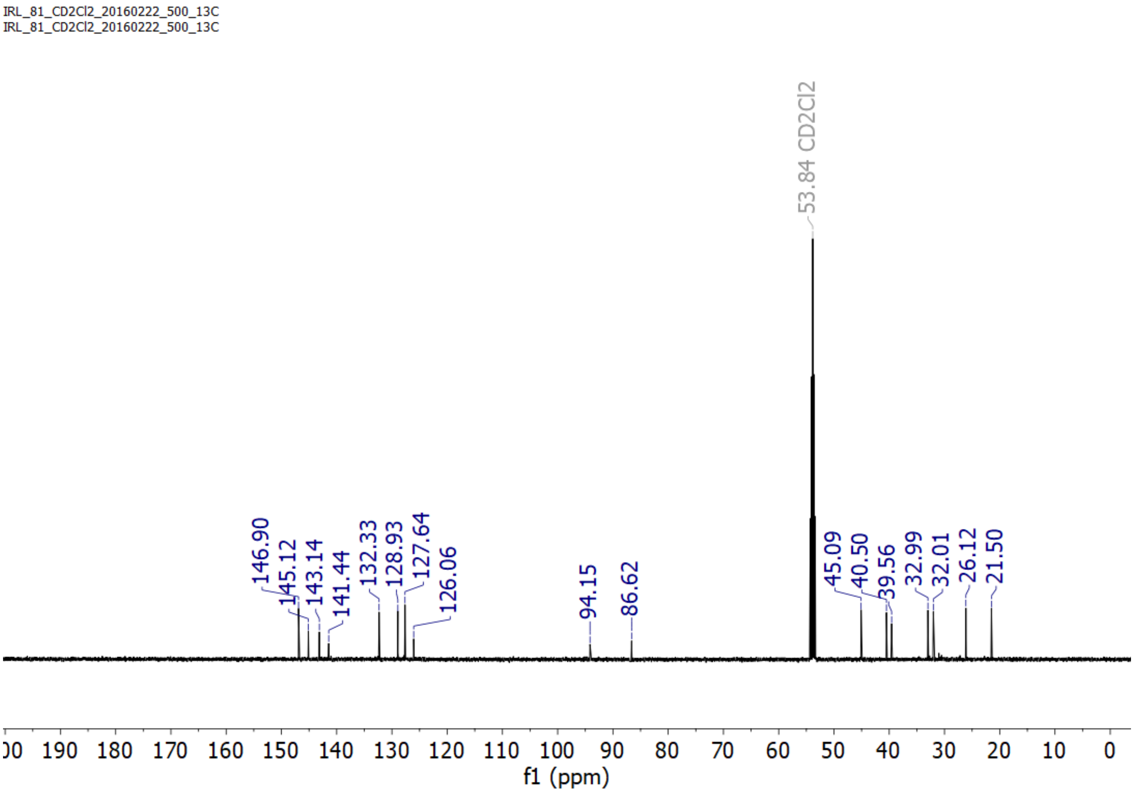

Figure S30. ^{13}C NMR spectrum of 1,2-bis(((6*R*,8*R*)-7,7-dimethyl-5,6,7,8-tetrahydro-6,8-methanoiso-quinolin-3-yl)ethynyl)benzene (**3**) in CD_2Cl_2 at 25°C (125.7 MHz).

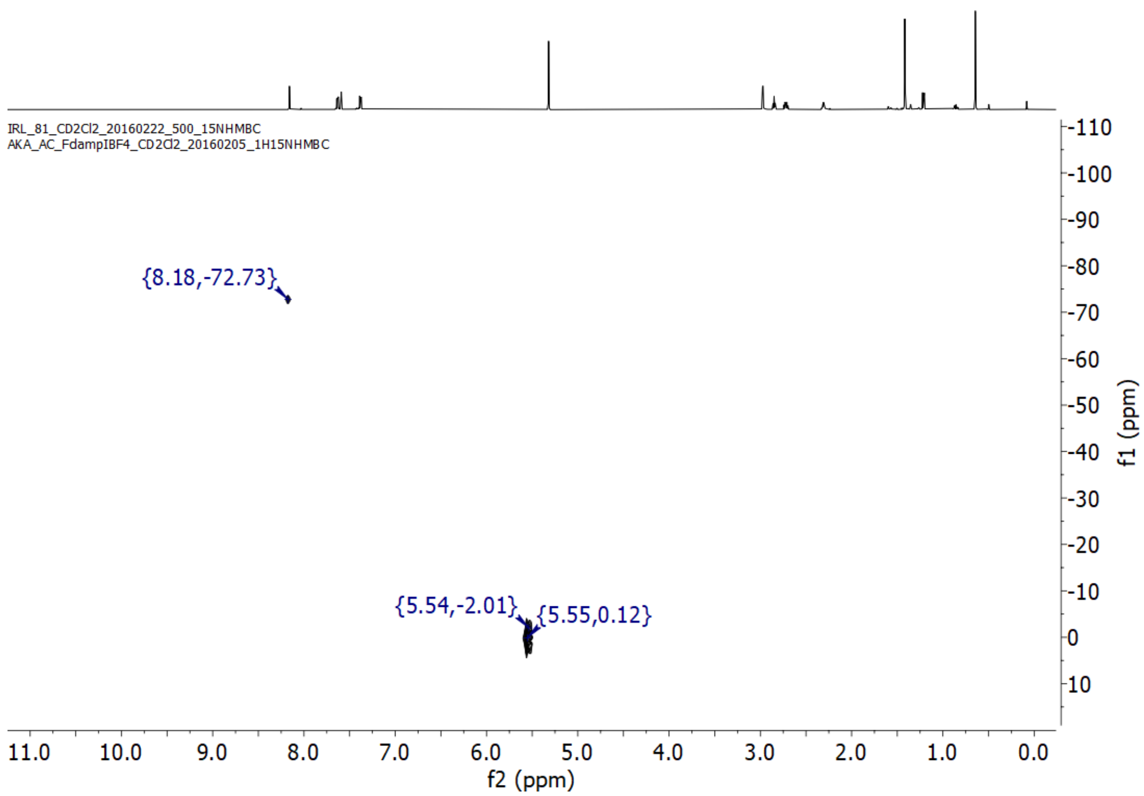

Figure S31. $^{1}H,^{15}N$ HMBC spectrum of 1,2-bis(((6*R*,8*R*)-7,7-dimethyl-5,6,7,8-tetrahydro-6,8-methanoiso-quinolin-3-yl)ethynyl)benzene (**3**) in CD_2Cl_2 at 25℃ (499.9/50.66 MHz).

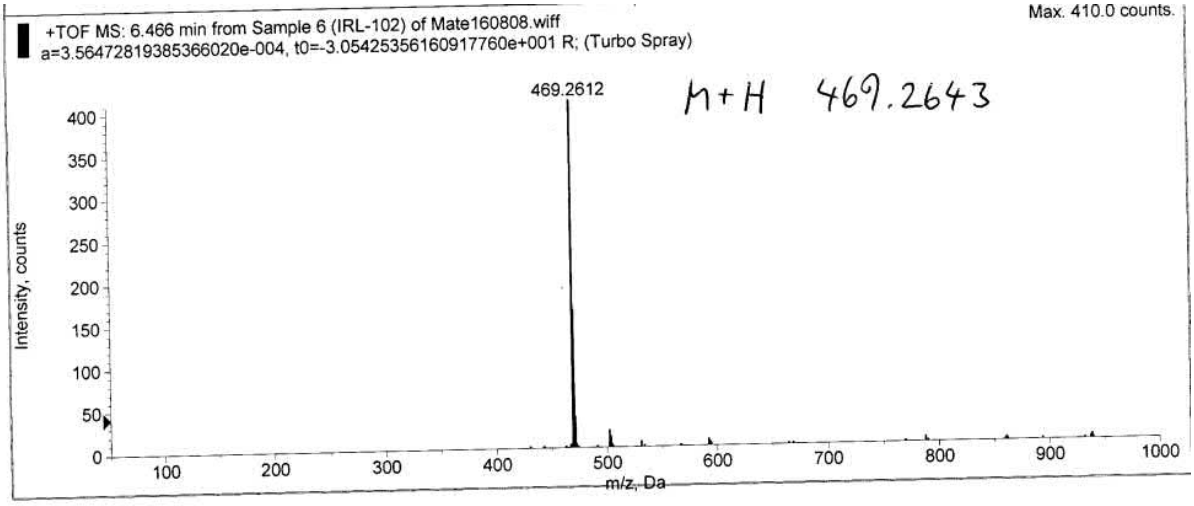

Figure S32. HRMS spectrum of 1,2-bis(((6*R*,8*R*)-7,7-dimethyl-5,6,7,8-tetrahydro-6,8-methanoiso-quinolin-3-yl)ethynyl)benzene (**3**).

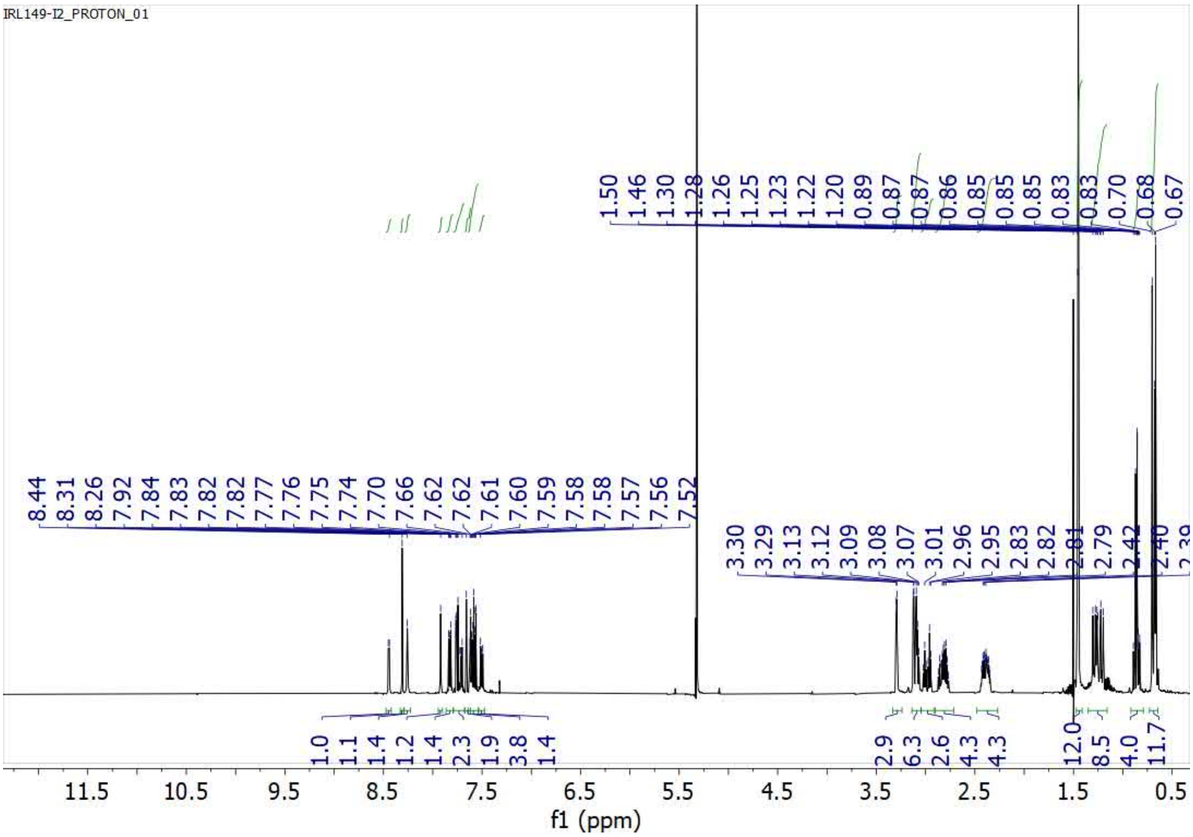

Figure S33. ^{1}H NMR spectrum of [(1,2-bis(((6*R*,8*R*)-7,7-dimethyl-5,6,7,8-tetrahydro-6,8-methanoiso-quinolin-3-yl)ethynyl)benzene)iodine(I)]tetrafluoroborate (**3-I**) in CD_2Cl_2 at 25°C (499.9 MHz).

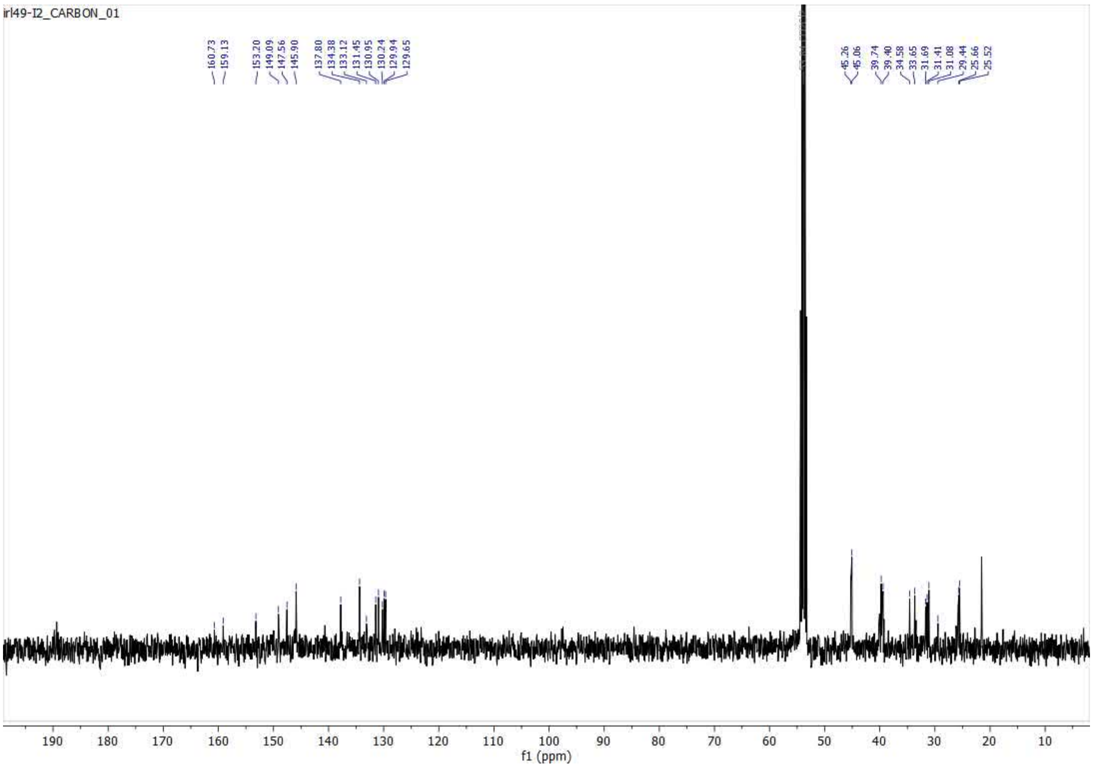

Figure S34. ^{13}C NMR spectrum of [(1,2-bis(((6*R*,8*R*)-7,7-dimethyl-5,6,7,8-tetrahydro-6,8-methanoisoquinolin-3-yl)ethynyl)benzene)iodine(I)] tetrafluoroborate (**3-I**) in CD_2Cl_2 at 25°C (125.7 MHz).

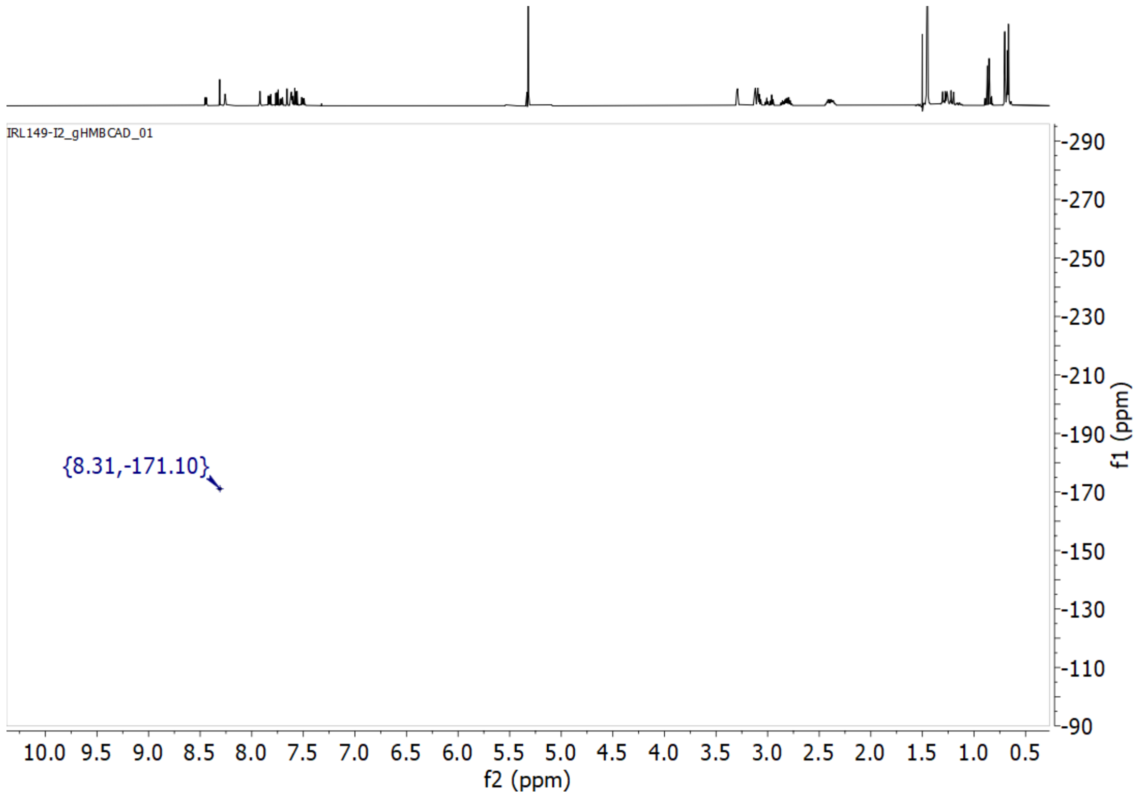

Figure S35. ^{1}H,^{15}N HMBC spectrum of [(1,2-bis(((6*R*,8*R*)-7,7-dimethyl-5,6,7,8-tetrahydro-6,8-methanoisoquinolin-3-yl)ethynyl)benzene)iodine(I)] tetrafluoroborate (**3-I**) in CD_2Cl_2 at 25°C (499.9/50.66 MHz).

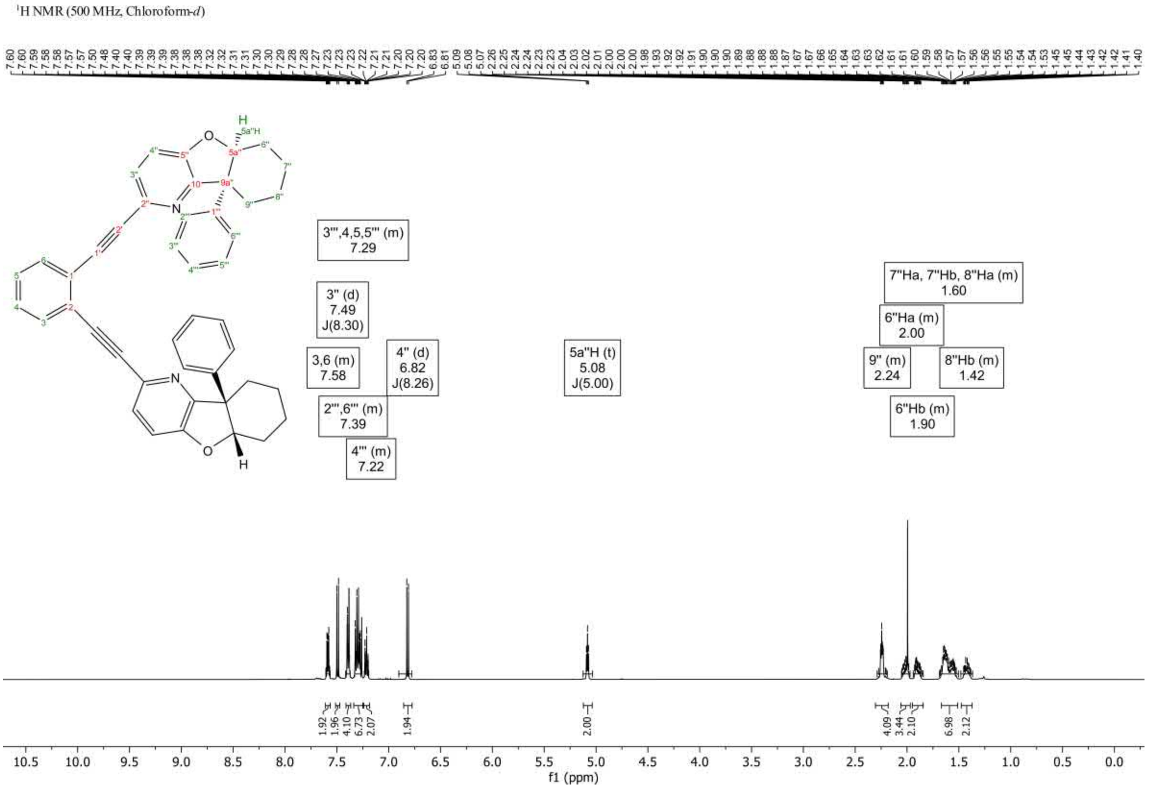

Figure S36. ^{1}H NMR spectrum of 1,2-bis-[((5a*S*,9a*R*)-9a-phenyl-5a,6,7,8,9,9a-hexahydrobenzofuro[3,2-b]pyridin-2-yl)-ethinyl]benzene ((5a*S*,9a*R*)-**4**) in $CDCl_3$ at 25°C and 500 MHz.

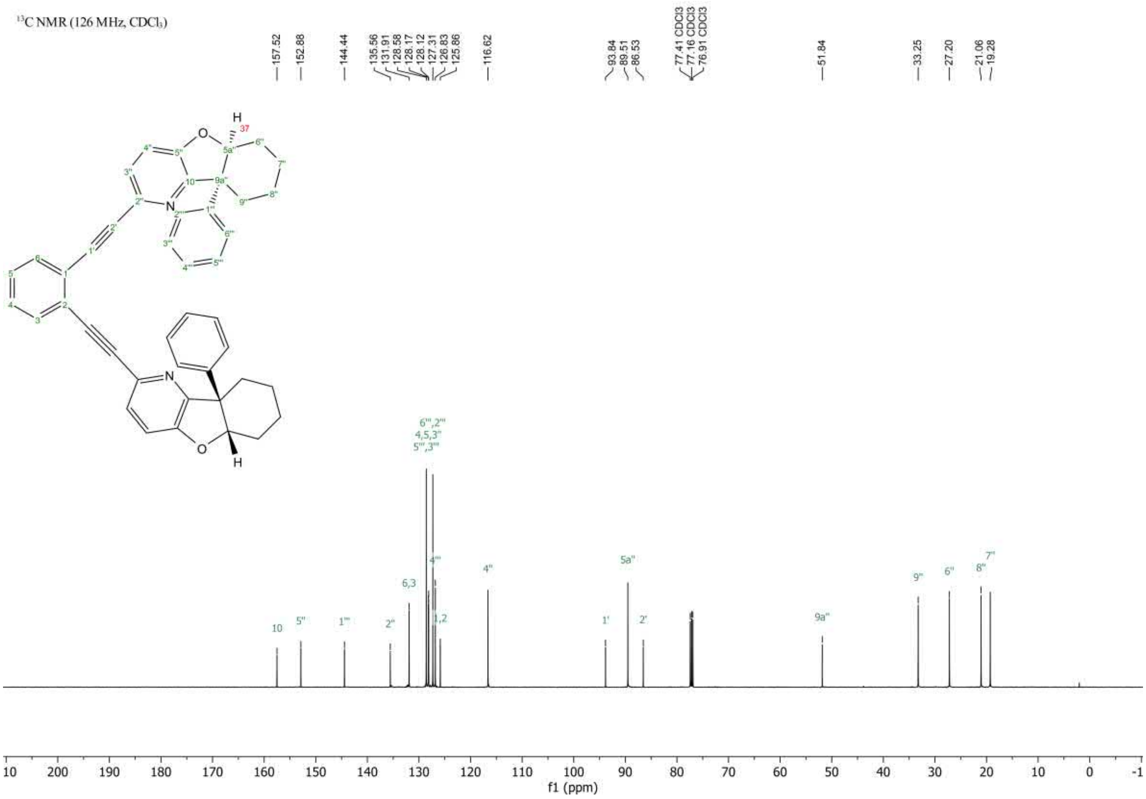

Figure S37. ^{13}C NMR spectrum of 1,2-bis-[((5a*S*,9a*R*)-9a-phenyl-5a,6,7,8,9,9a-hexahydrobenzo-furo[3,2-b]pyridin-2-yl)-ethinyl]benzene ((5a*S*,9a*R*)-**4**) in $CDCl_3$ at 25℃, and 126 MHz.

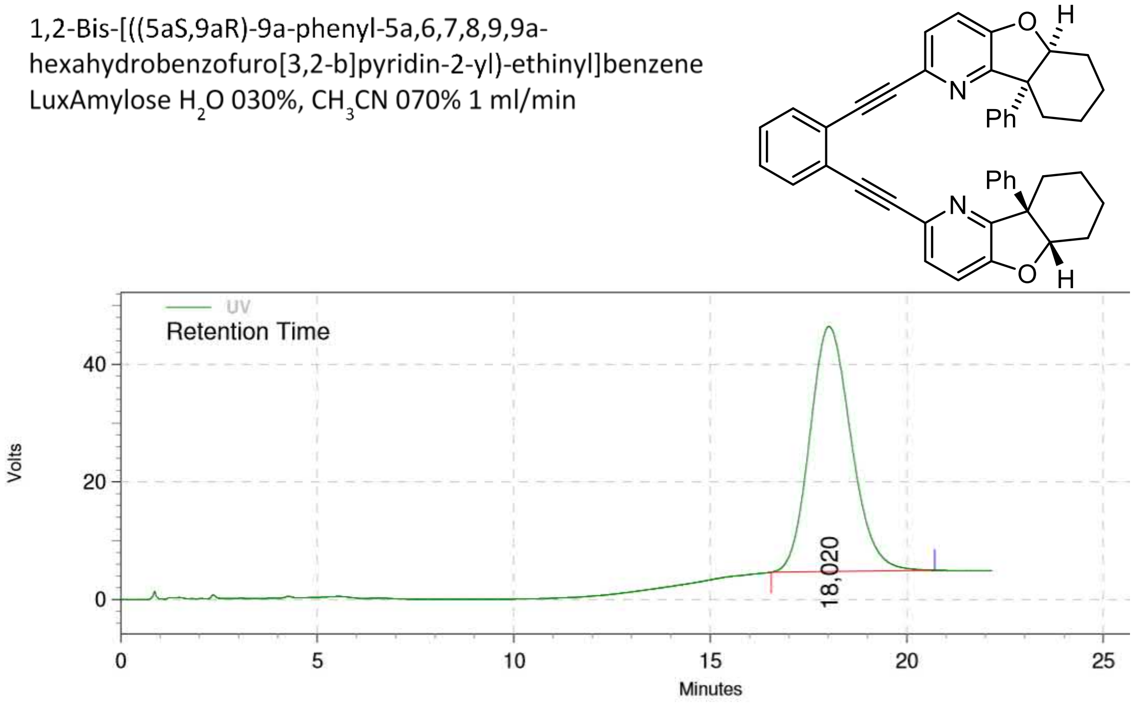

Figure S38. HPLC chromatogram of 1,2-bis-[((5a*S*,9a*R*)-9a-phenyl-5a,6,7,8,9,9a-hexahydrobenzo-furo[3,2-b]pyridin-2-yl)-ethinyl]benzene ((5a*S*,9a*R*)-**4**), LuxAmylose H_2O 30%, CH_3CN 70% 1ml/min.

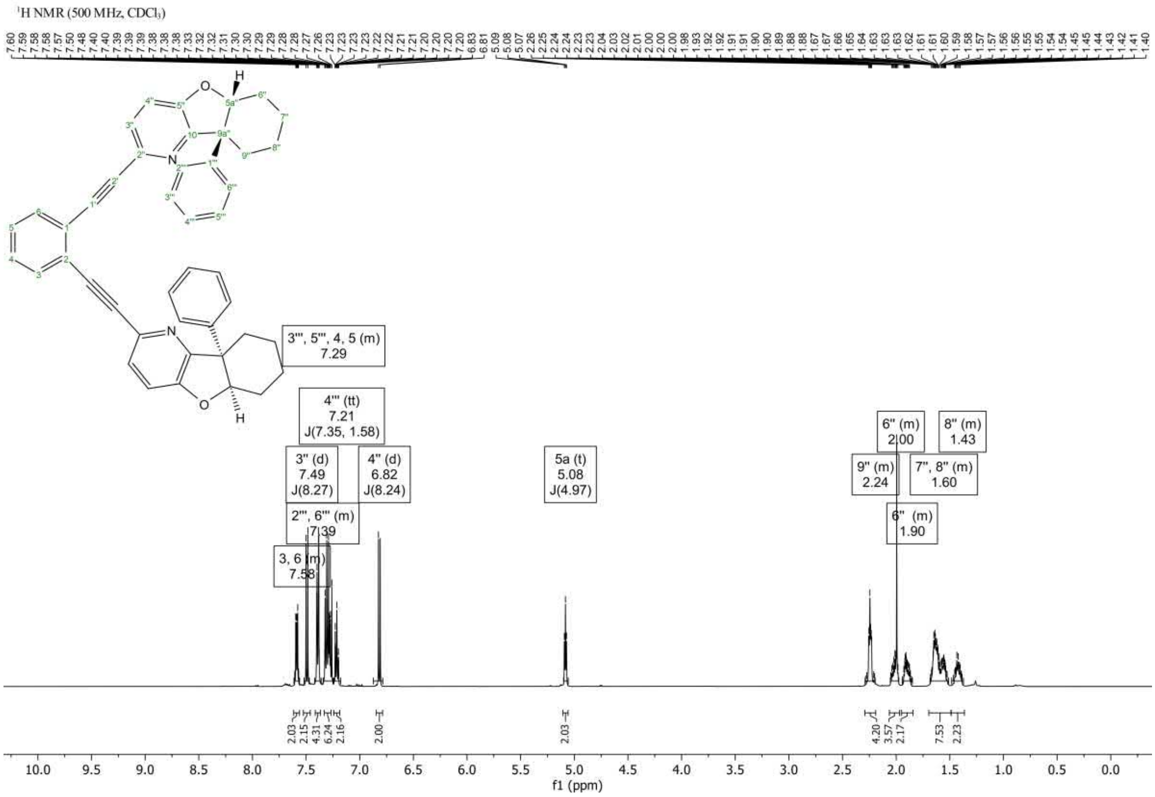

Figure S39. ^{1}H NMR spectrum of 1,2-bis-[((5a*R*,9a*S*)-9a-phenyl-5a,6,7,8,9,9a-hexahydrobenzofuro[3,2-b]pyridin-2-yl)-ethinyl]benzene ((5a*R*,9a*S*)-**4**) in $CDCl_3$ at 25°C and 500 MHz.

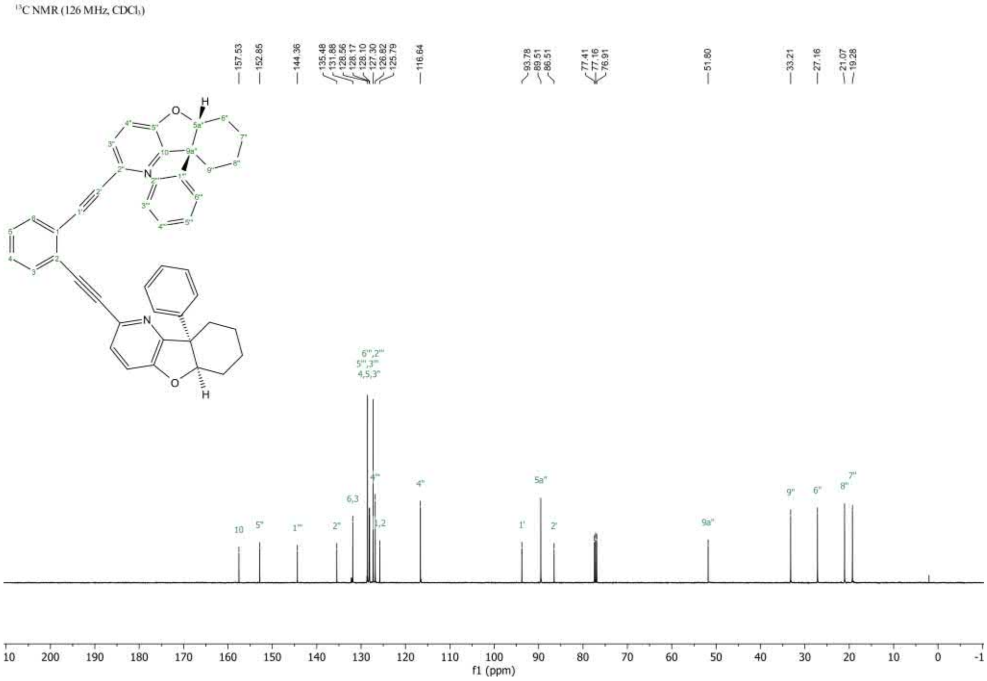

Figure S40. ^{13}C NMR spectrum of 1,2-bis-[((5a*R*,9a*S*)-9a-phenyl-5a,6,7,8,9,9a-hexahydrobenzofuro[3,2-b]pyridin-2-yl)-ethinyl]benzene ((5a*R*,9a*S*)-**4**) in $CDCl_3$ at 25°C, and 126 MHz.

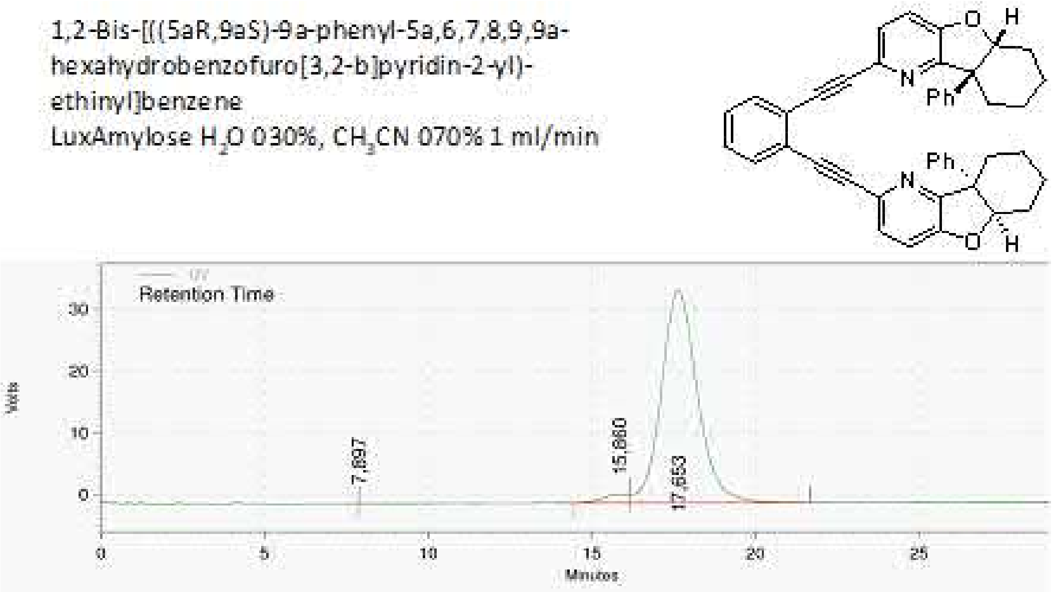

UV Results

Retention Time	Area	Area %	Height	Height %
7,897	7	0,00	4	0,00
15,860	290906	2,70	5421	3,79
17,653	10494262	97,30	137518	96,20

Figure S41. HPLC chromatogram of 1,2-bis-[((5aR,9aS)-9a-phenyl-5a,6,7,8,9,9a-hexahydrobenzo-furo[3,2-*b*]pyridin-2-yl)-ethinyl]benzene ((5aR,9aS)-**4**), LuxAmylose H_2O 30%, CH_3CN 70% 1ml/min.

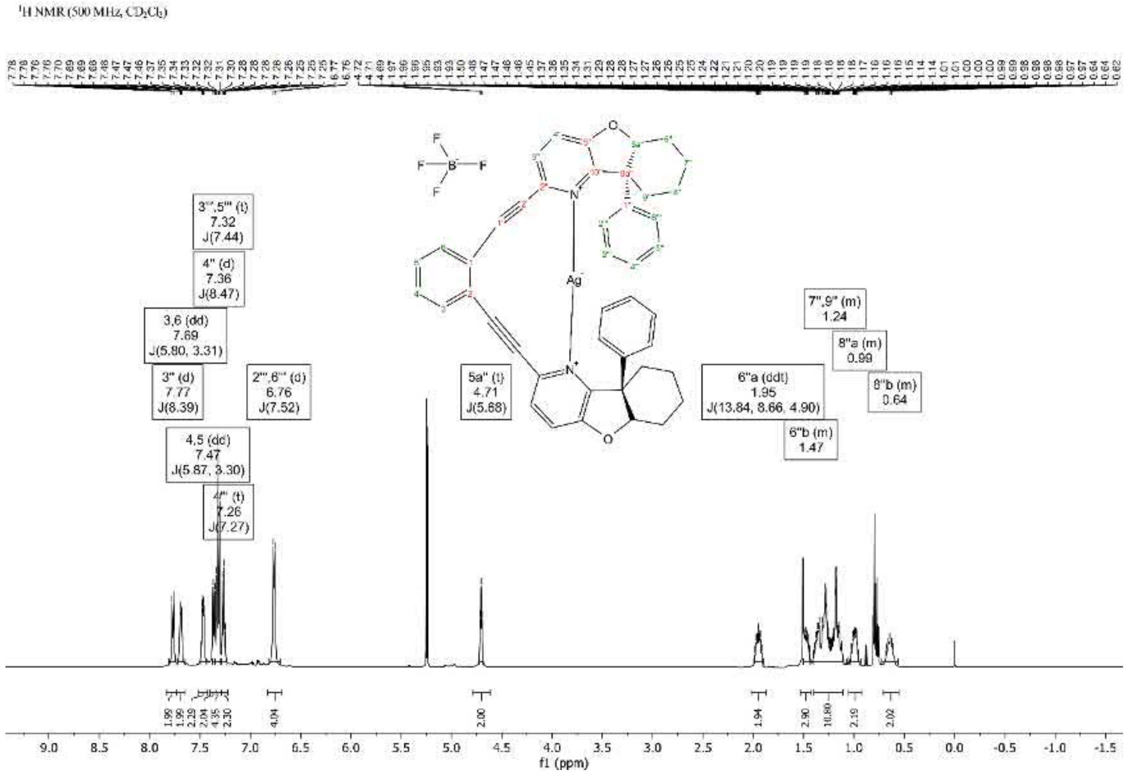

Figure S42. ^{1}H NMR spectrum of [(1,2-bis-[((5a*R*,9a*S*)-9a-phenyl-5a,6,7,8,9,9a-hexahydrobenzofuro[3,2-b]pyridin-2-yl)-ethinyl]benzene)silver(I)]tetrafluoroborate ((5a*R*,9a*S*)-**4-Ag**) in CD_2Cl_2 at 25°C and 500 MHz.

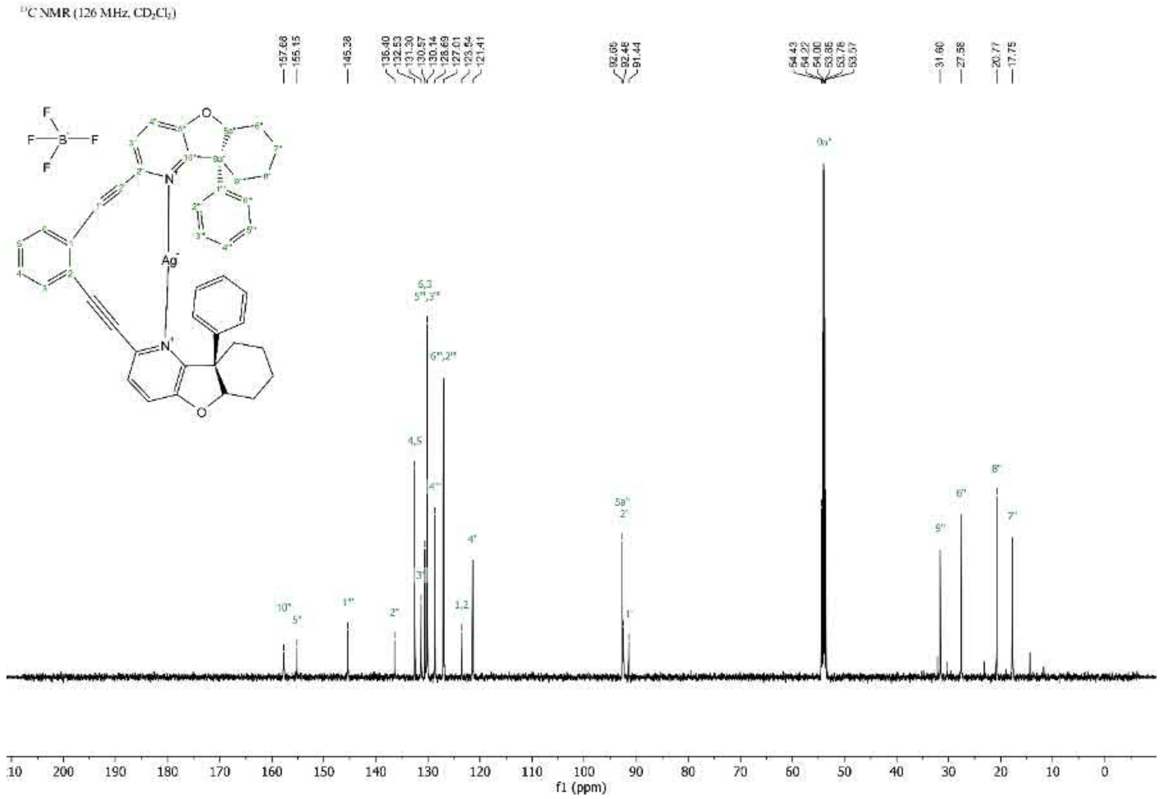

Figure S43. ^{13}C NMR spectrum of of [(1,2-bis-[((5a*R*,9a*S*)-9a-phenyl-5a,6,7,8,9,9a-hexahydrobenzo-furo[3,2-b]pyridin-2-yl)-ethinyl]benzene)silver(I)]tetrafluoroborate ((5a*R*,9a*S*)-**4-Ag**) in CD_2Cl_2 at 25℃, and 126 MHz.

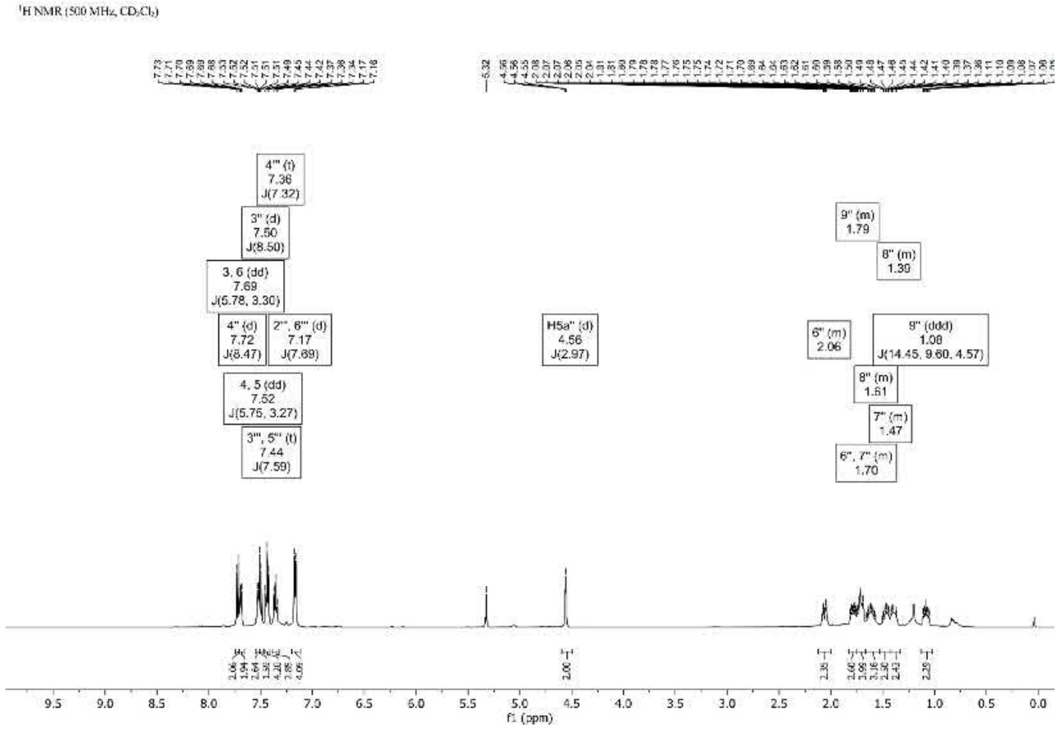

Figure S44. ^{1}H NMR spectrum of [(1,2-bis-[((5a*R*,9a*S*)-9a-phenyl-5a,6,7,8,9,9a-hexahydrobenzofuro[3,2-b]pyridin-2-yl)-ethinyl]benzene)iodine(I)]tetrafluoroborate ((5a*R*,9a*S*)-**4-I**) in CD_2Cl_2 at 25℃ and 500 MHz.

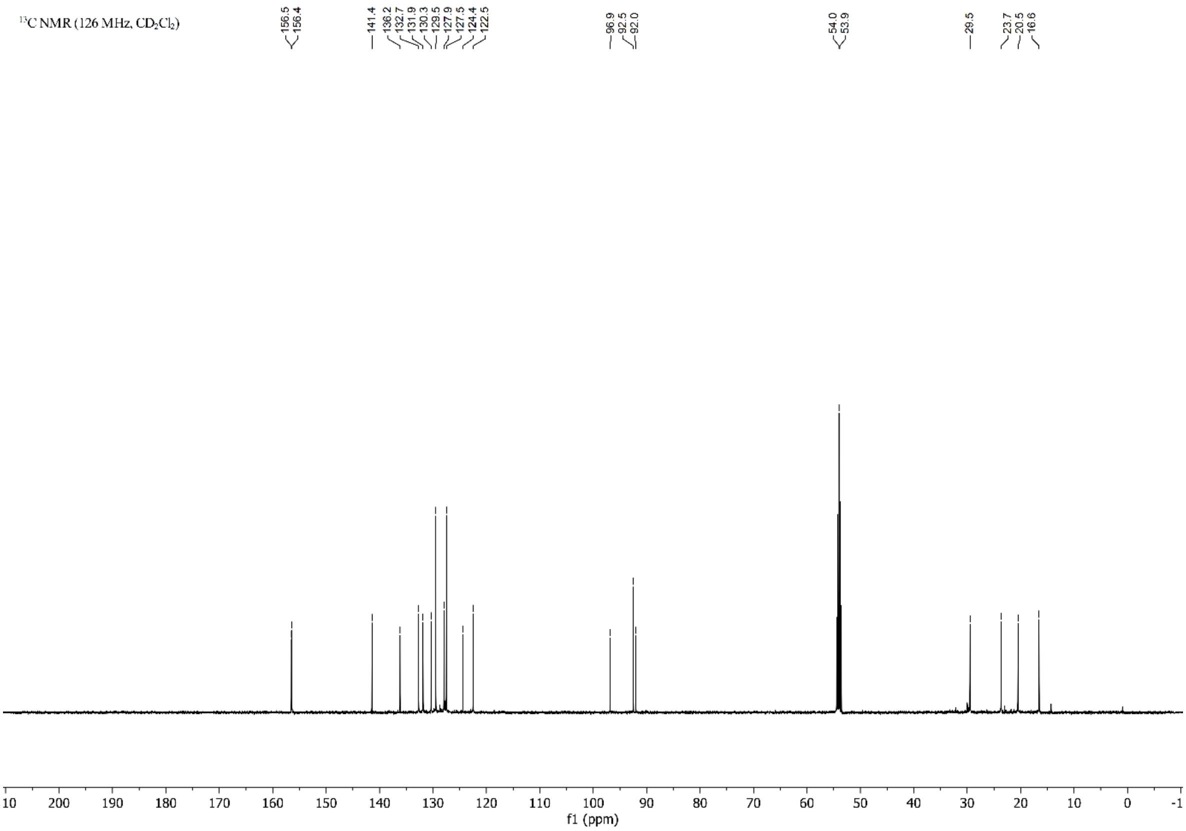

Figure S45. ^{13}C NMR spectrum of of [(1,2-bis-[((5a*R*,9a*S*)-9a-phenyl-5a,6,7,8,9,9a-hexahydrobenzofuro[3,2-b]pyridin-2-yl)-ethinyl]benzene)iodine(I)]tetrafluoroborate ((5a*R*,9a*S*)-**4-I**) in CD_2Cl_2 at 25°C, and 126 MHz.

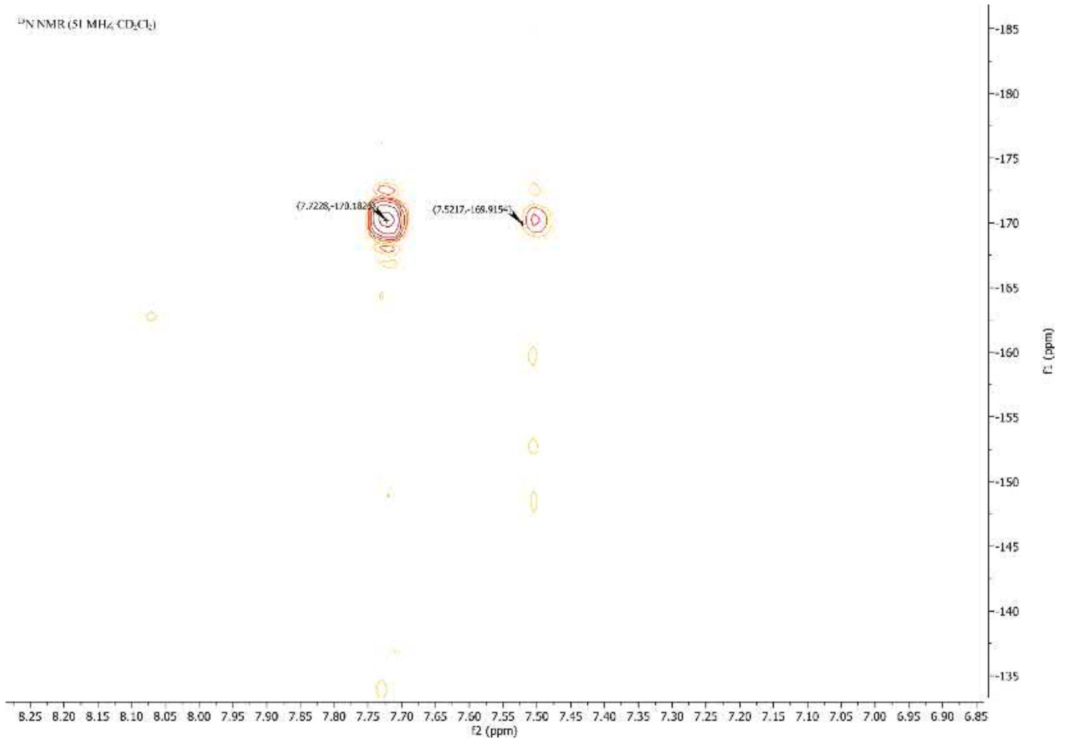

Figure S46. ^{1}H,^{15}N HMBC spectrum of of [(1,2-bis-[((5a*R*,9a*S*)-9a-phenyl-5a,6,7,8,9,9a-hexahydrobenzofuro[3,2-b]pyridin-2-yl)-ethinyl]benzene)iodine(I)]tetrafluoroborate ((5a*R*,9a*S*)-**4-I**) in CD_2Cl_2 at 25°C (499.9/50.66 MHz).

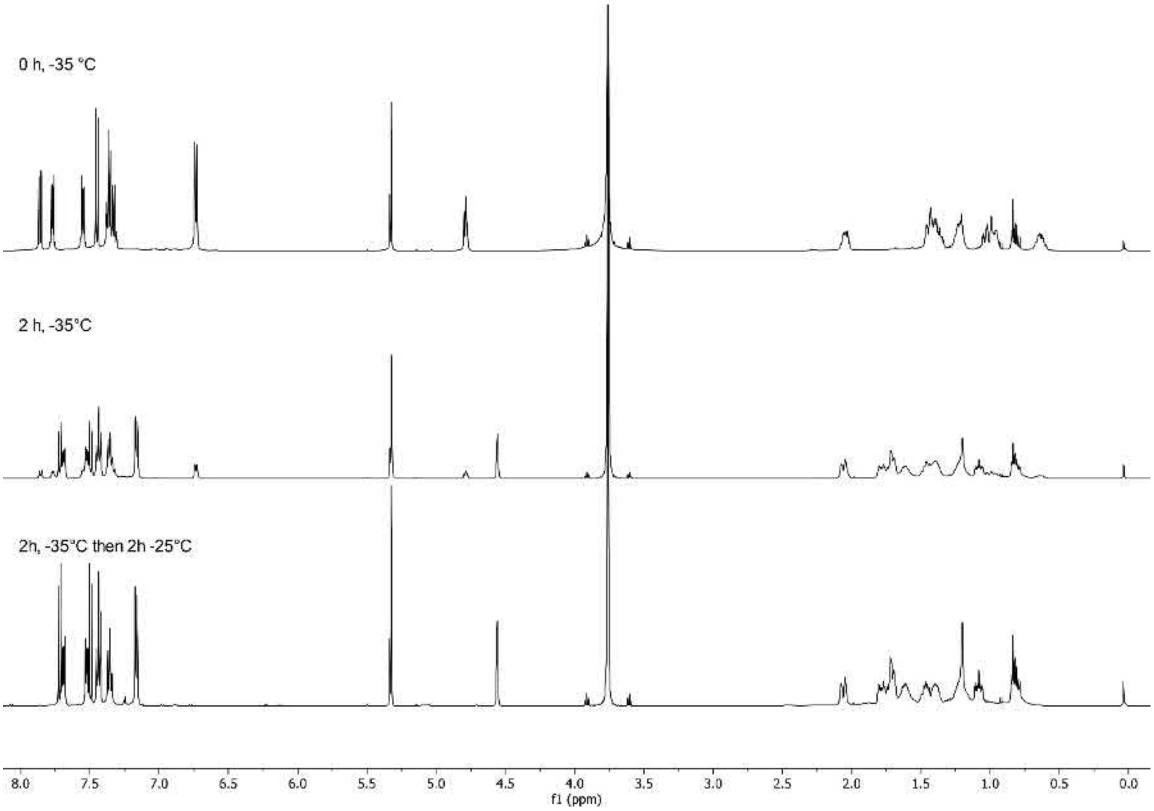

Figure S47. The conversion of (5a*R*,9a*S*)-4-Ag to (5a*R*,9a*S*)-**4**-I followed by ^{1}H NMR at -5°C in CD_2Cl_2.

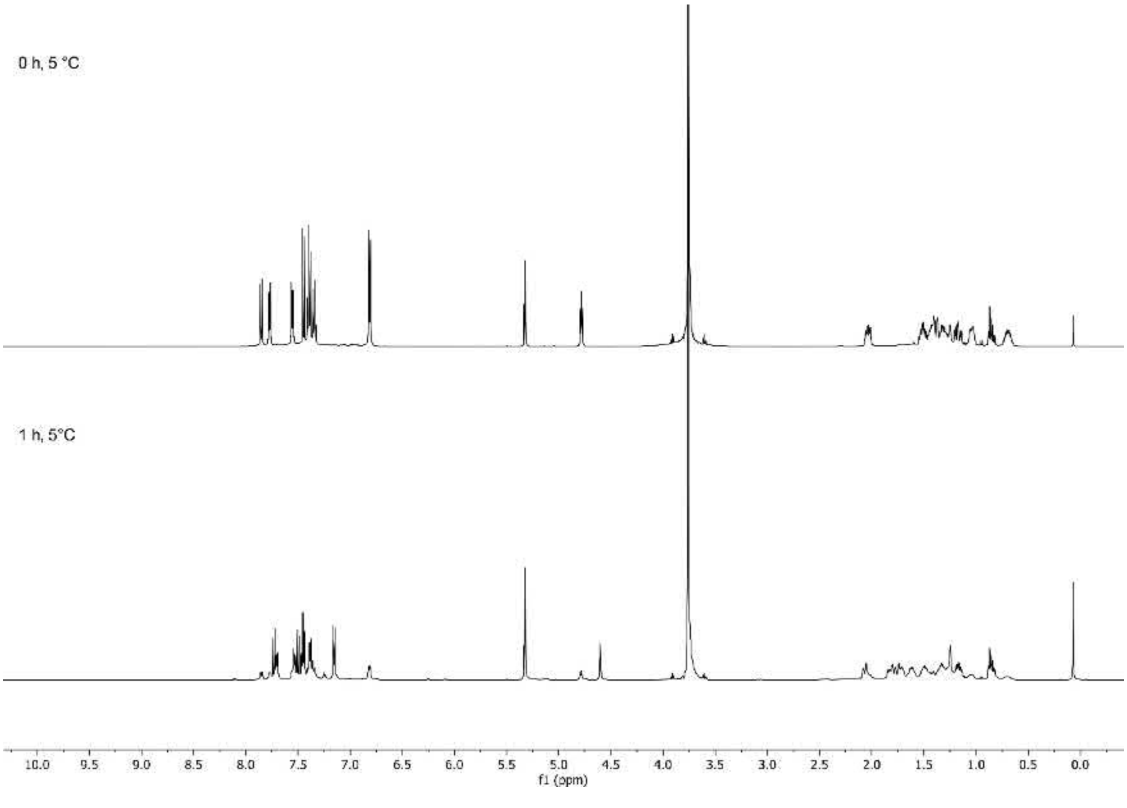

Figure S48. The conversion of (5a*R*,9a*S*)-4-Ag to (5a*R*,9a*S*)-**4**-I followed by ^{1}H NMR at 5°C in CD_2Cl_2.

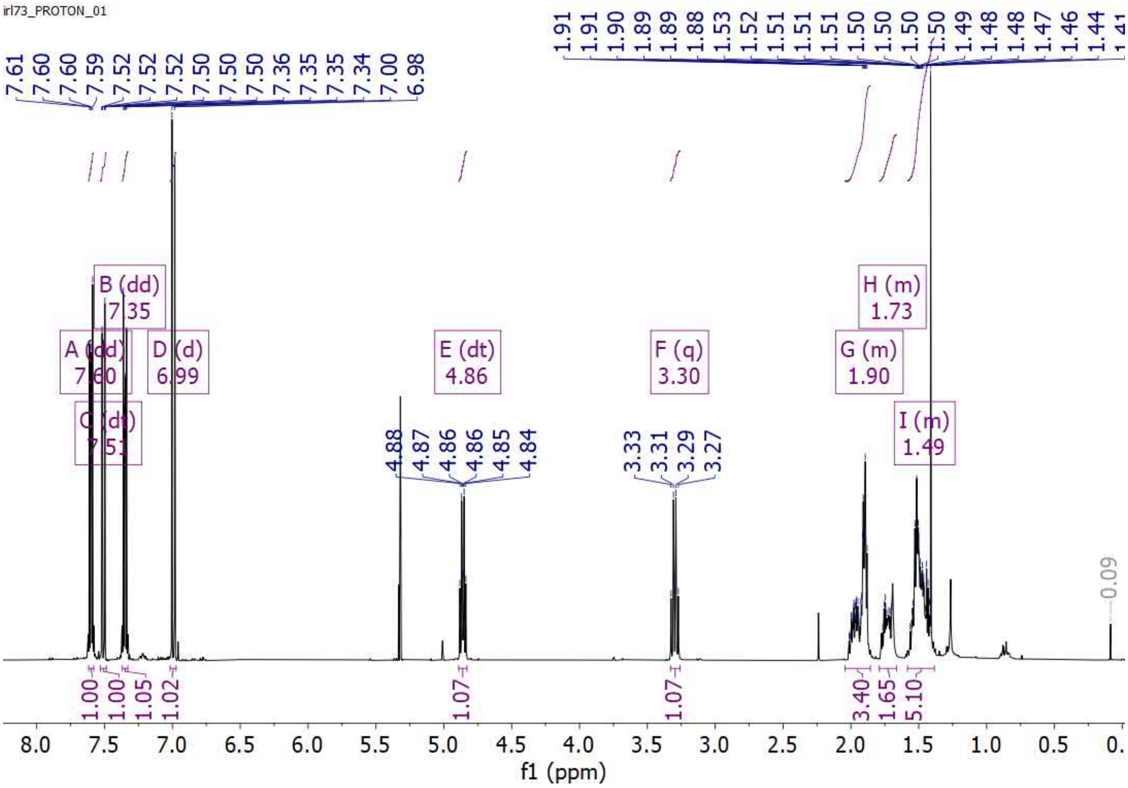

Figure S49. ^{1}H NMR spectrum of 1,2-bis(((5a*S*,9a*S*)-5a,6,7,8,9,9a-hexahydrobenzofuro[3,2-*b*]pyridin-2-yl)ethynyl)benzene (**5**) in CD_2Cl_2 at 25°C and 399.9 MHz.

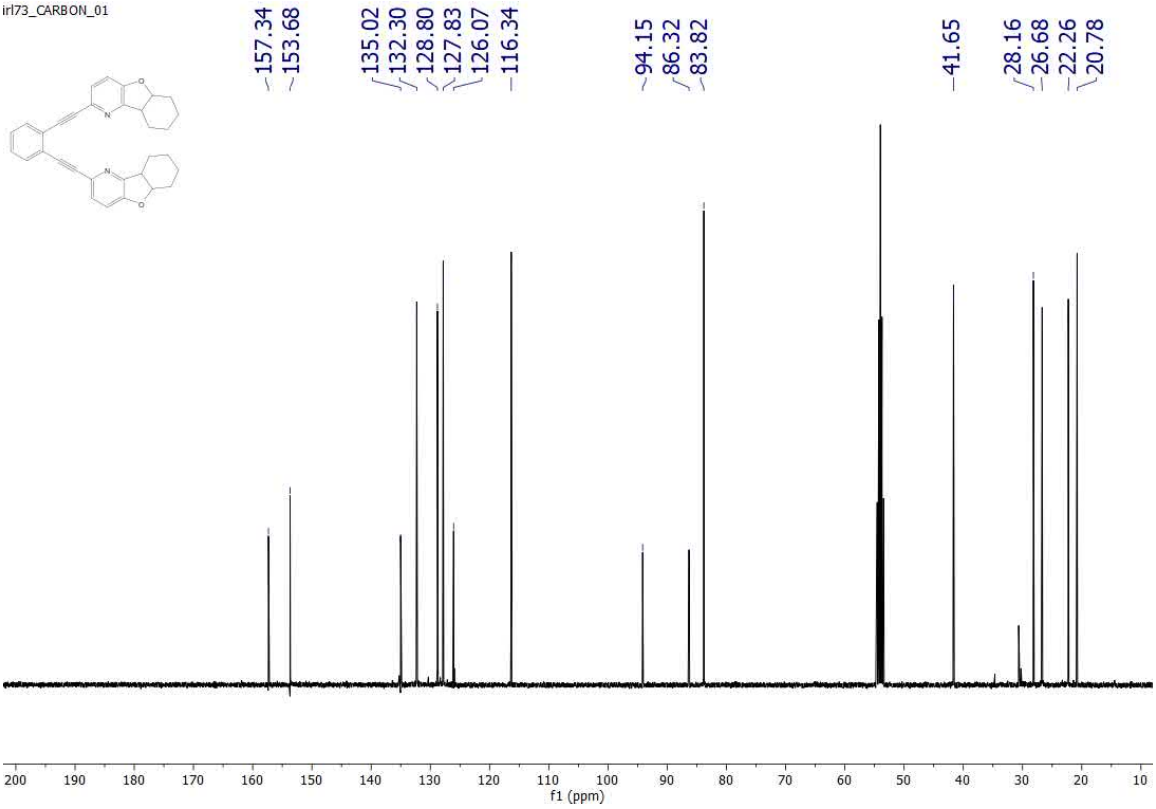

Figure S50. ^{13}C NMR spectrum of of 1,2-bis(((5a*S*,9a*S*)-5a,6,7,8,9,9a-hexahydrobenzofuro[3,2-*b*]pyridin-2-yl)ethynyl)benzene (**5**) in CD_2Cl_2 at 25°C, and 100.58 MHz.

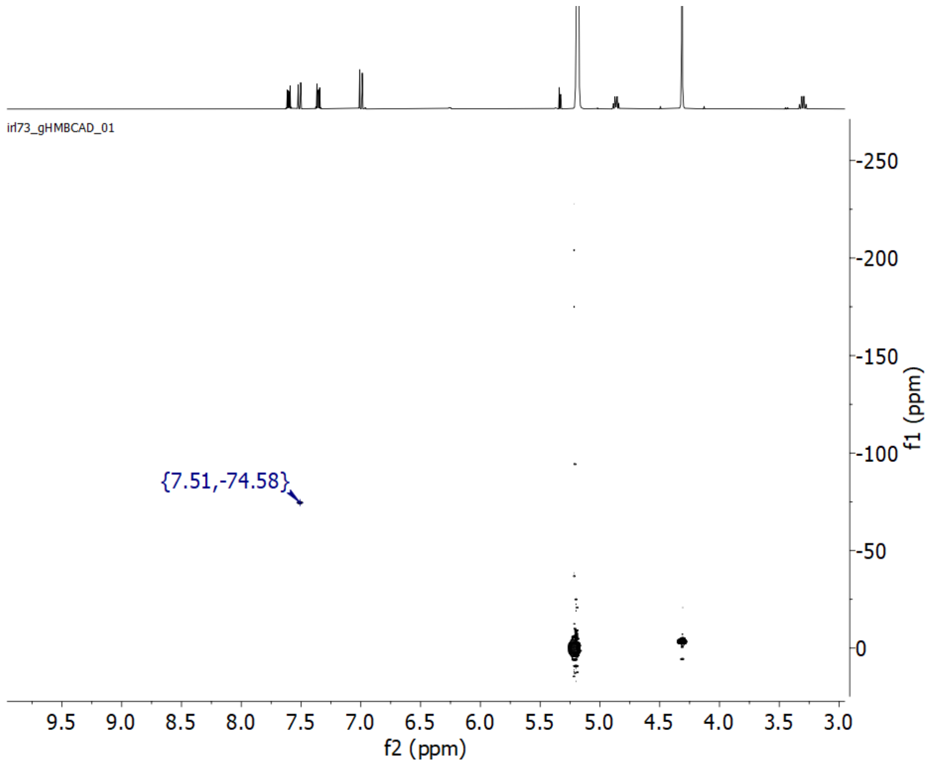

Figure S51. $^{1}H,^{15}N$ HMBC spectrum of 1,2-bis(((5a*S*,9a*S*)-5a,6,7,8,9,9a-hexahydrobenzofuro[3,2-*b*]pyridin-2-yl)ethynyl)benzene (**5**) in CD_2Cl_2 at 25°C (499.9/50.66 MHz).

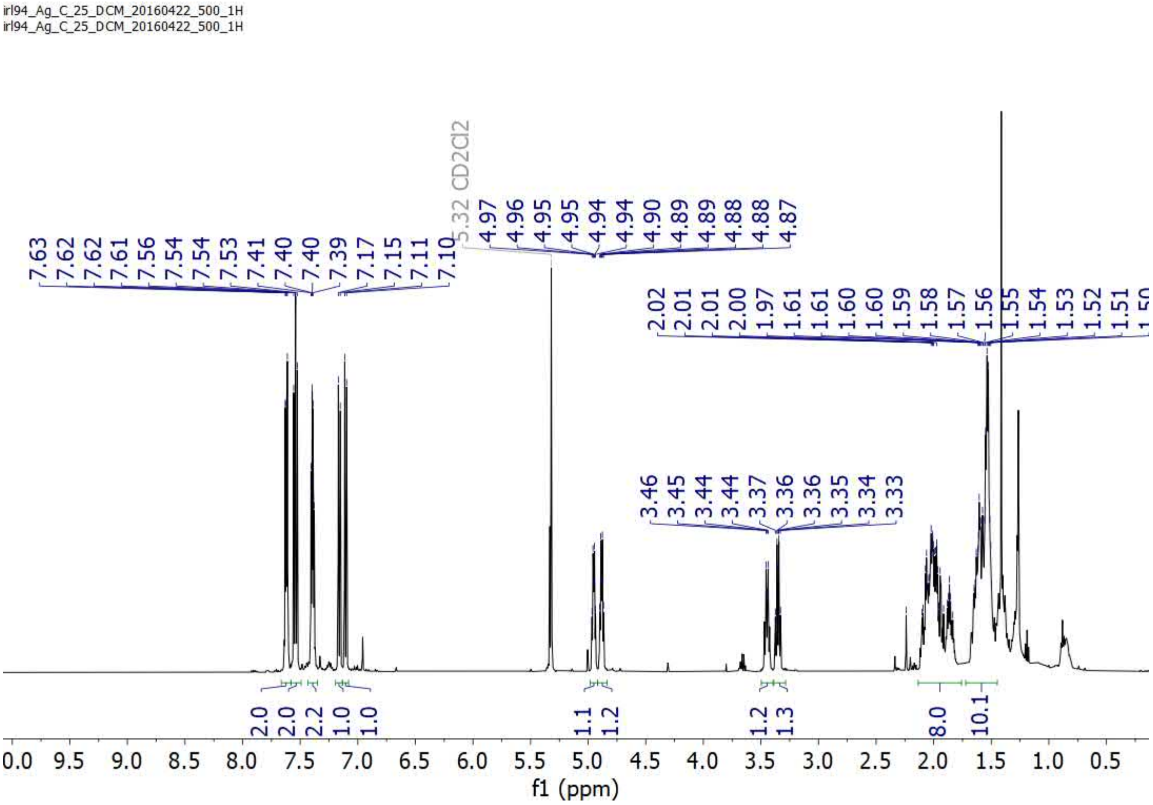

Figure S52. ^{1}H NMR spectrum of [(1,2-bis(((5a*S*,9a*S*)-5a,6,7,8,9,9a-hexahydrobenzofuro[3,2-*b*]pyridin-2-yl)ethynyl)benzene)silver(I)]tetrafluoroborate (**5-Ag**) in CD_2Cl_2 at 25°C, and 400 MHz.

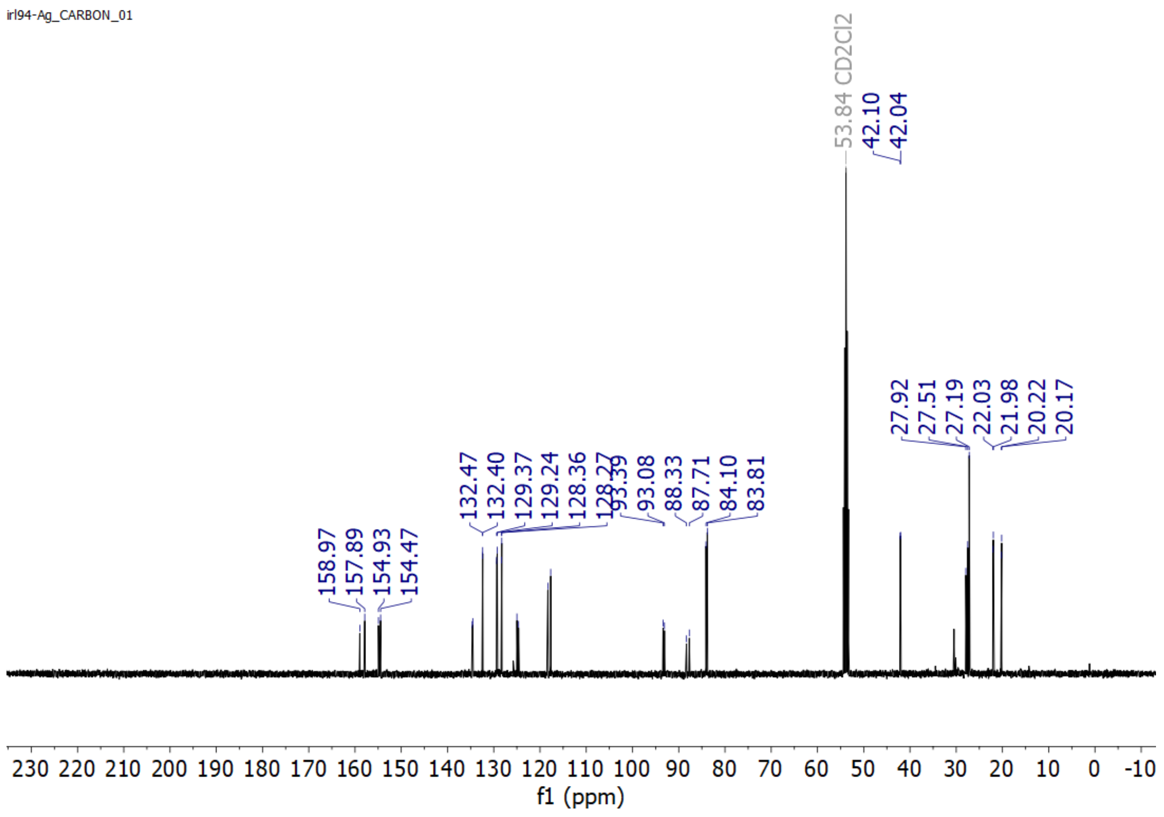

Figure S53. ^{13}C NMR spectrum of [(1,2-bis(((5a*S*,9a*S*)-5a,6,7,8,9,9a-hexahydrobenzofuro[3,2-*b*]pyridin-2-yl)ethynyl)benzene)silver(I)]tetrafluoroborate (**5-Ag)** in CD_2Cl_2 at 25°C, and 100.58 MHz.

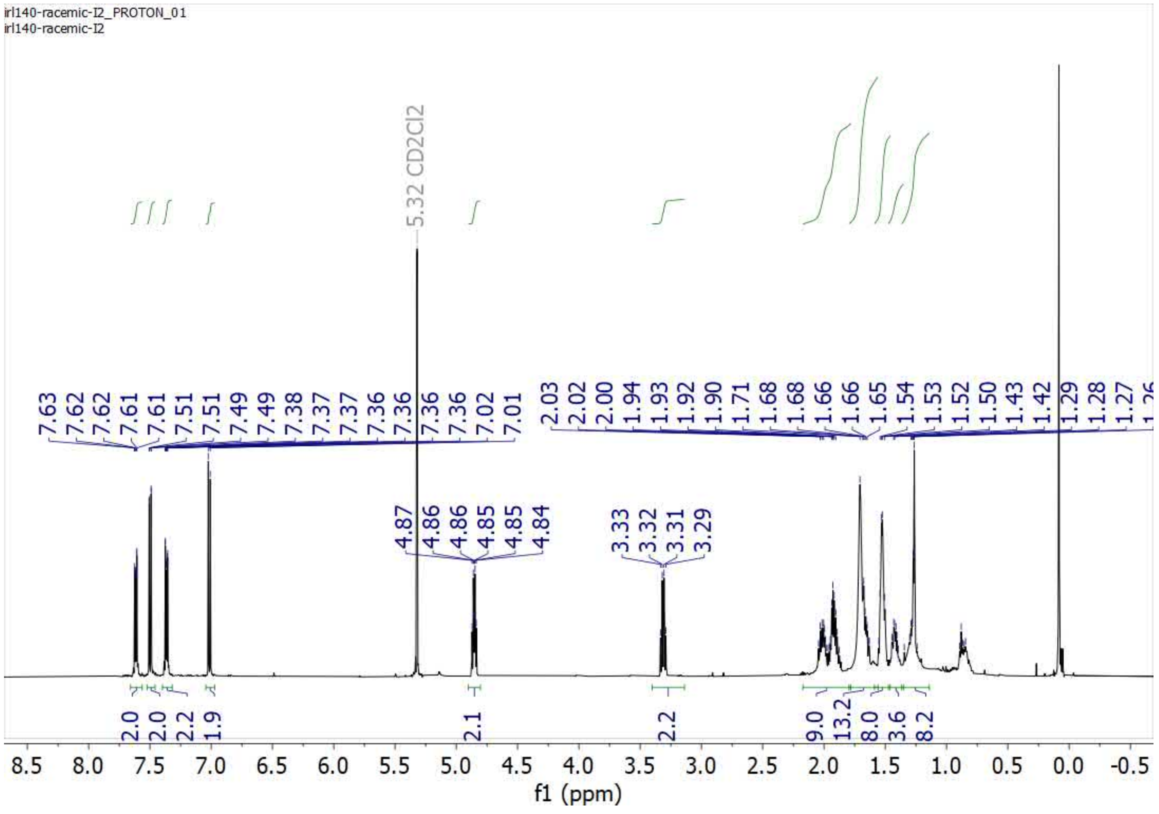

Figure S54. ^{1}H NMR spectrum of of [(1,2-bis(((5a*S*,9a*S*)-5a,6,7,8,9,9a-hexahydrobenzofuro[3,2-*b*]pyridin-2-yl)ethynyl)benzene)iodine(I)]tetrafluoroborate (**5-I**) in CD_2Cl_2 at 25°C, and 400 MHz.

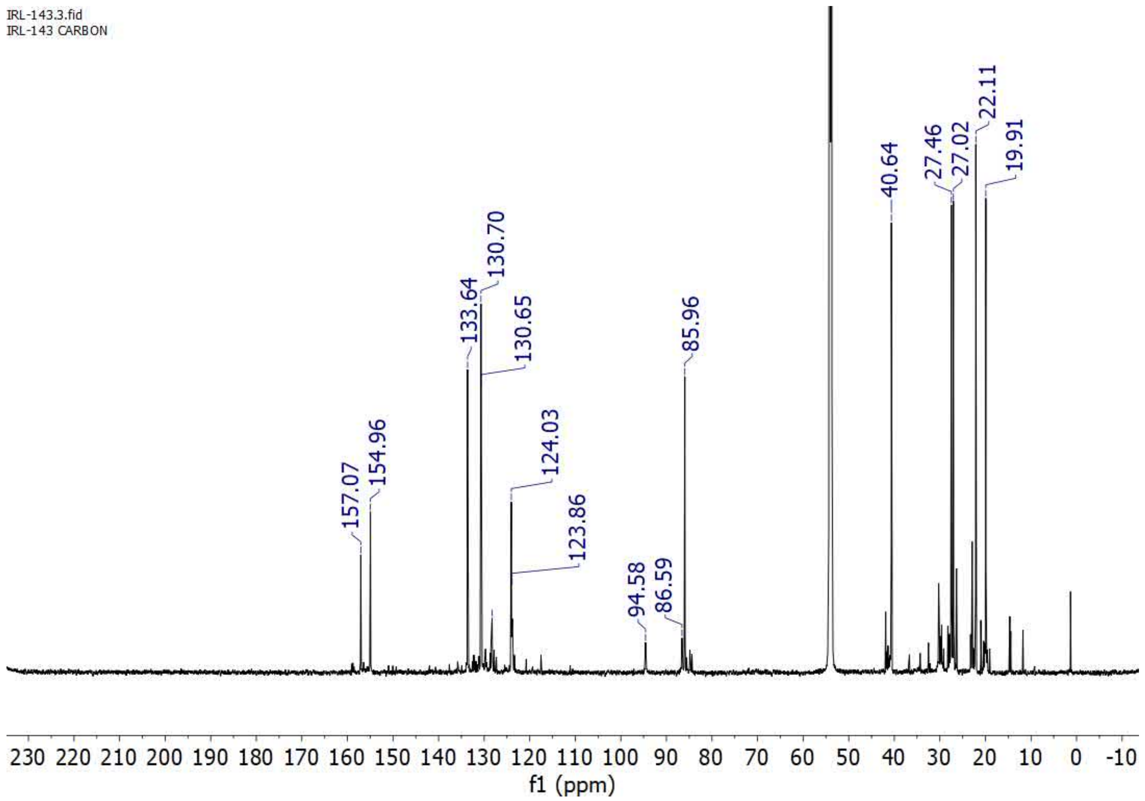

Figure S55. ^{13}C NMR spectrum of [(1,2-bis(((5a*S*,9a*S*)-5a,6,7,8,9,9a-hexahydrobenzofuro[3,2-*b*]pyridin-2-yl)ethynyl)benzene)silver(I)]tetrafluoroborate (**5-I**) in CD_2Cl_2 at 25℃, and 100.58 MHz.

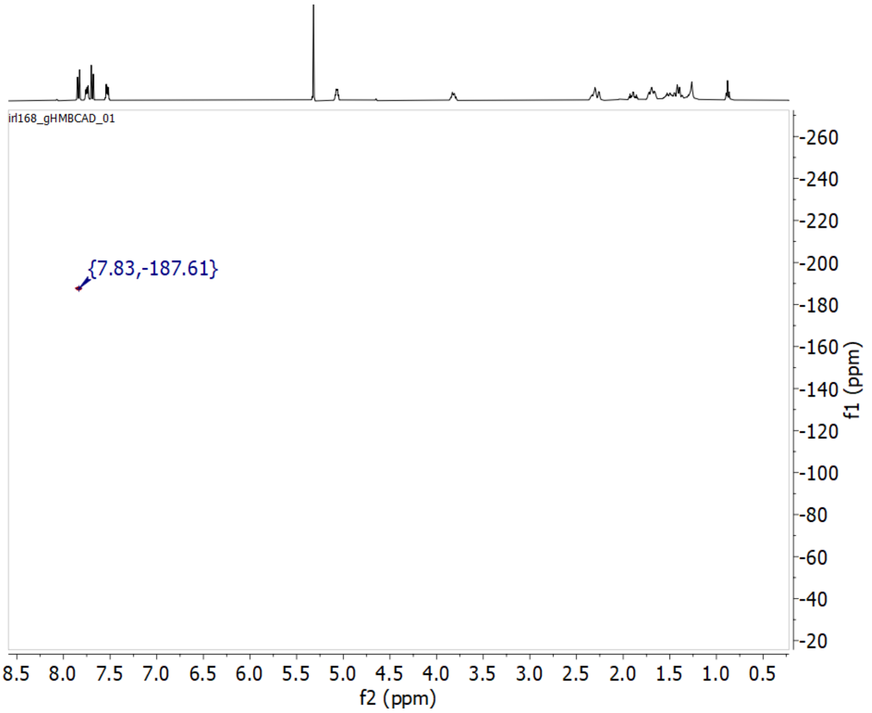

Figure S56. ^{1}H,^{15}N HMBC spectrum of [(1,2-bis(((5a*S*,9a*S*)-5a,6,7,8,9,9a-hexahydrobenzofuro[3,2-*b*]pyridin-2-yl)ethynyl)benzene)silver(I)]tetrafluoroborate (**5-I**) in CD_2Cl_2 at 25℃ (499.9/50.66 MHz).

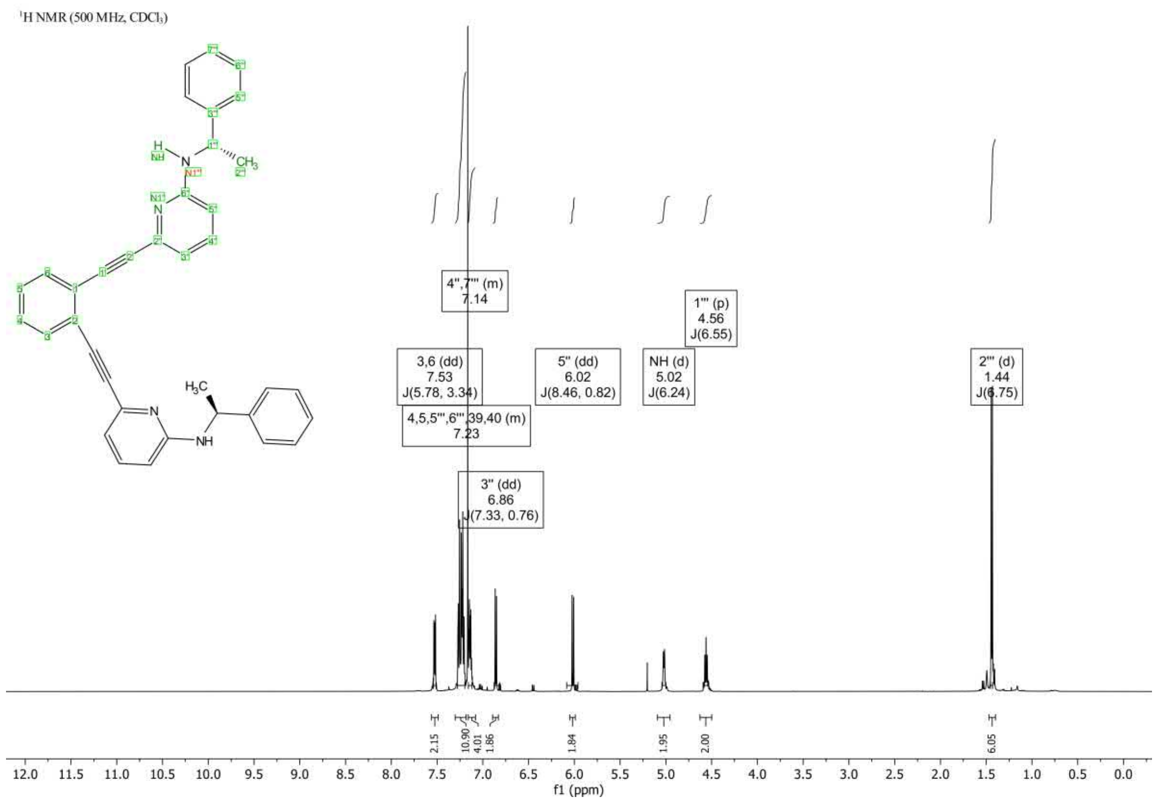

Figure S57. ^{1}H NMR spectrum of 1,2-Bis((6-(N-((1R)-1-phenylethyl)amino)pyridin-2-yl)ethinyl]benzene (*R*,*R*-**6**) in $CDCl_3$ at 25°C and 500 MHz.

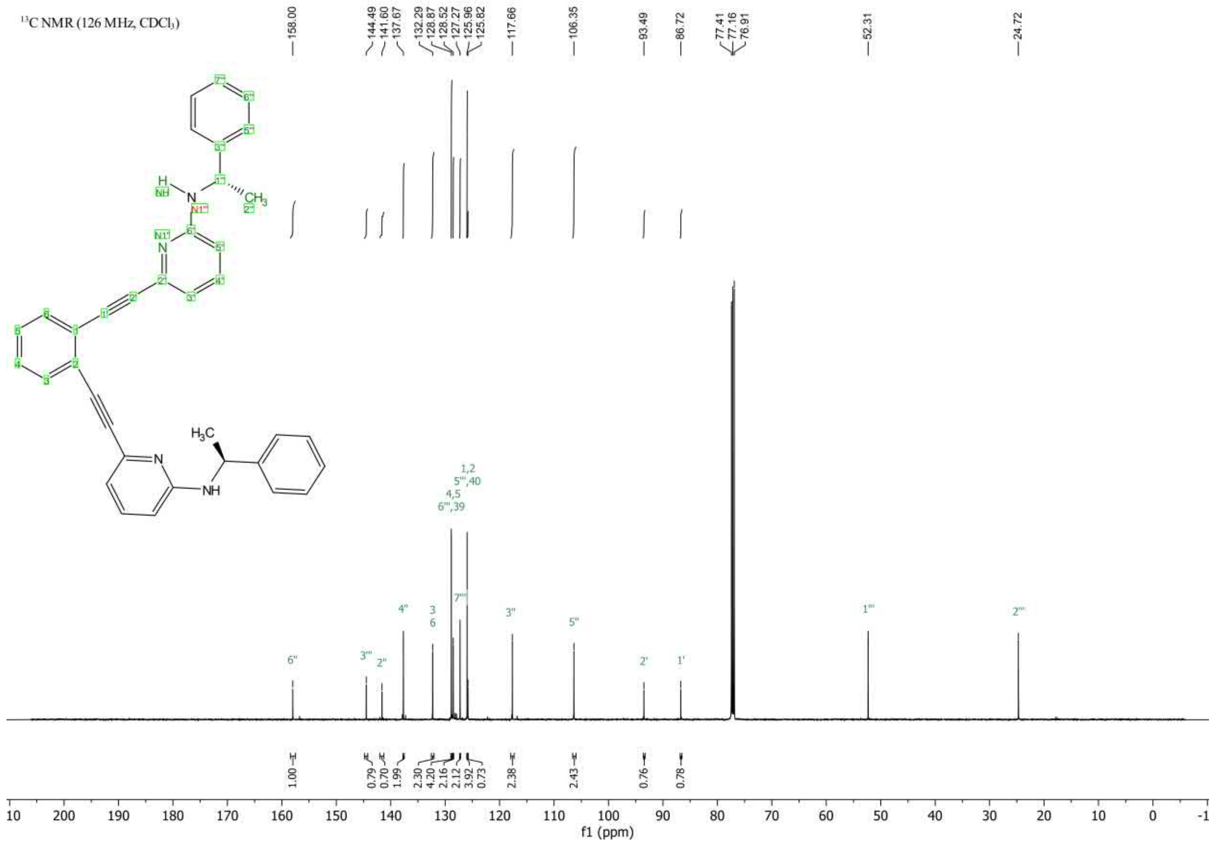

Figure S58. ^{13}C NMR spectrum of 1,2-bis((6-(N-((1R)-1-phenylethyl)amino)pyridin-2-yl)ethinyl]benzene (*R*,*R*-**6**) in $CDCl_3$ at 25℃, and 126 MHz.

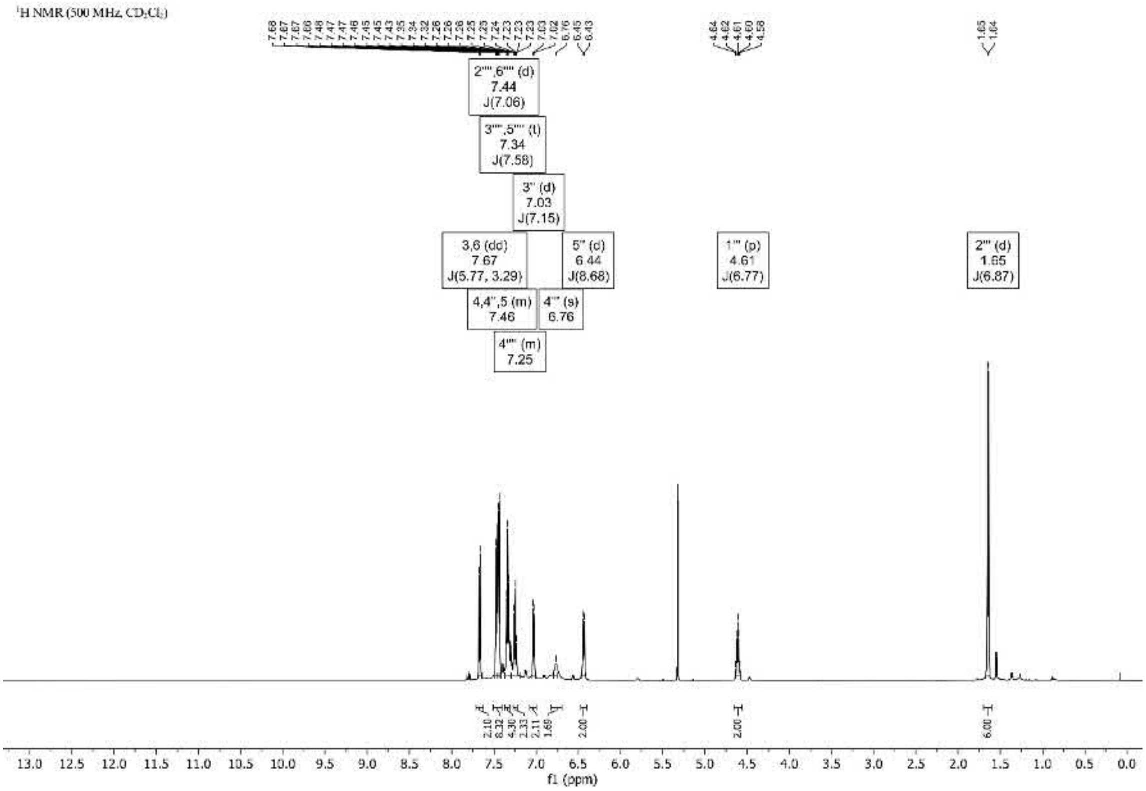

Figure S59. ^{1}H NMR spectrum of [(1,2-bis((6-(N-((1R)-1-phenylethyl)amino)pyridin-2-yl)ethinyl]benzene)silver(I)] tetrafluoroborate (*R*,*R*-**6**-Ag) in CD_2Cl_2 at 25°C and 500 MHz.

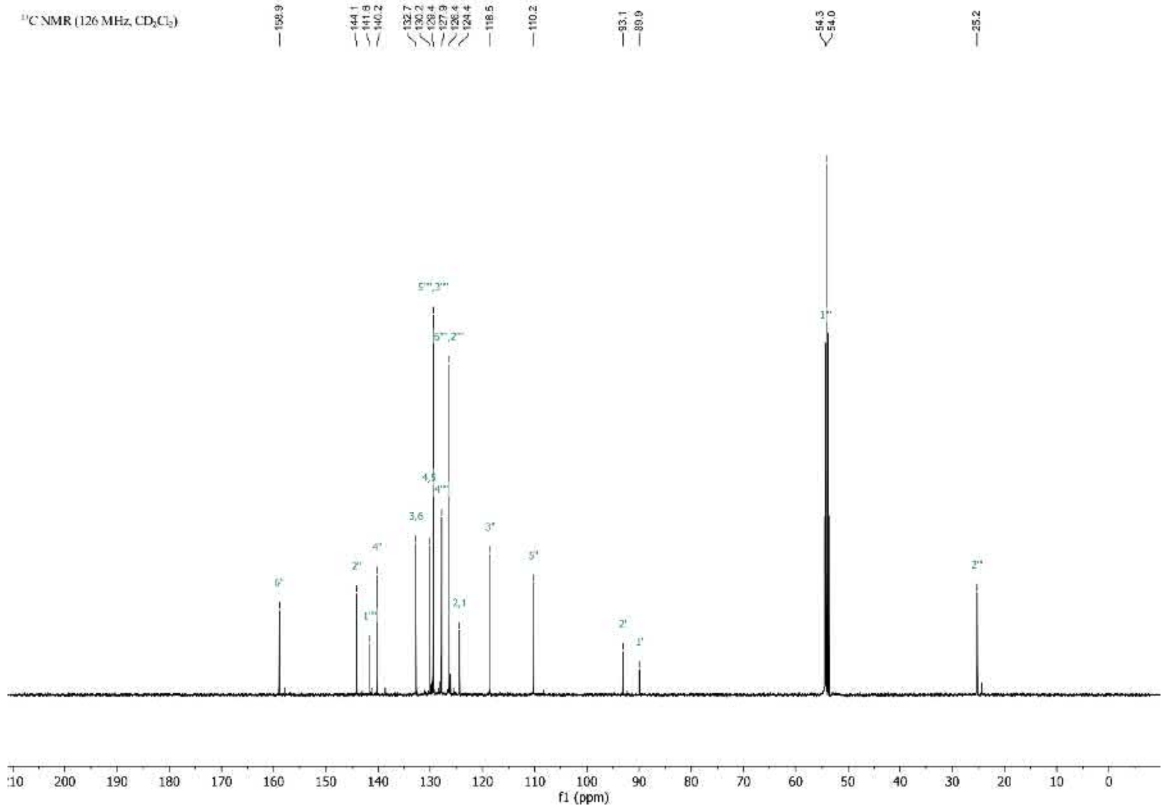

Figure S60. ^{13}C NMR spectrum of [(1,2-bis((6-(N-((1R)-1-phenylethyl)amino)pyridin-2-yl)ethinyl]benzene)silver(I)] tetrafluoroborate (*R*,*R*-**6**-Ag) in CD_2Cl_2 at 25°C, and 126 MHz.

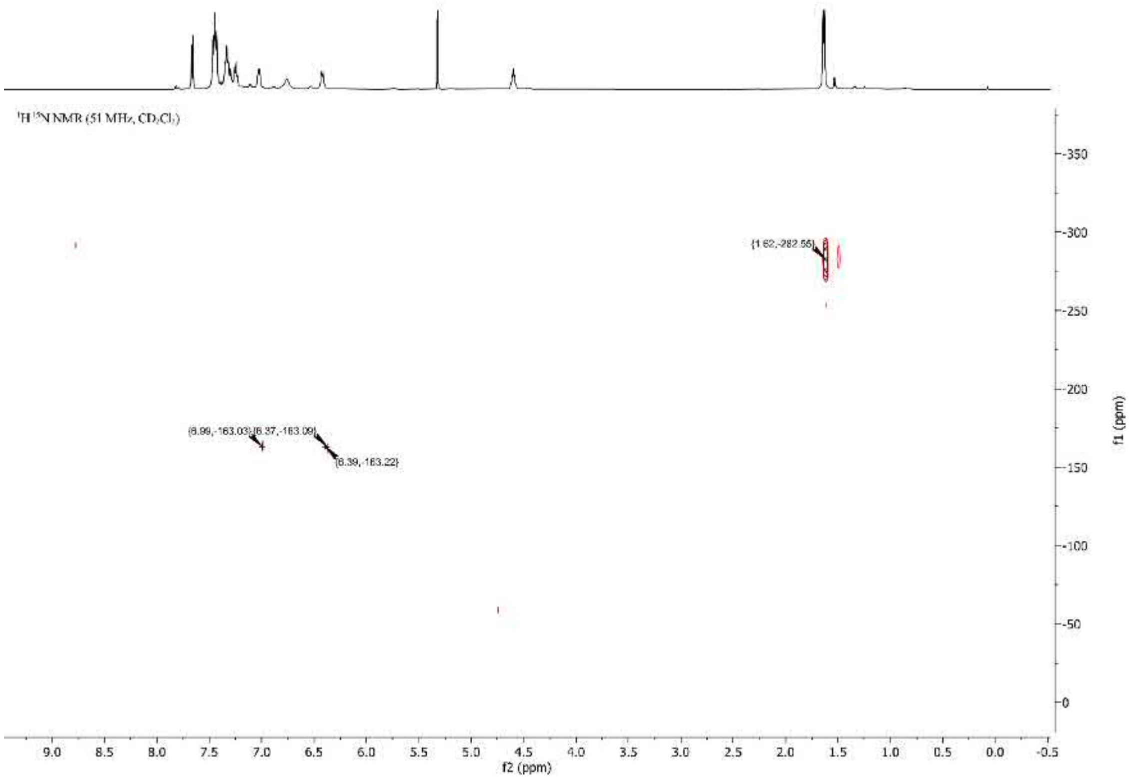

Figure S61. ^{1}H,^{15}N HMBC spectrum of [(1,2-bis((6-(N-((1R)-1-phenylethyl)amino)pyridin-2-yl)ethinyl]benzene)silver(I)] tetrafluoroborate (*R*,*R*-**6**-Ag) in CD_2Cl_2 at 25℃ (499.9/50.66 MHz).

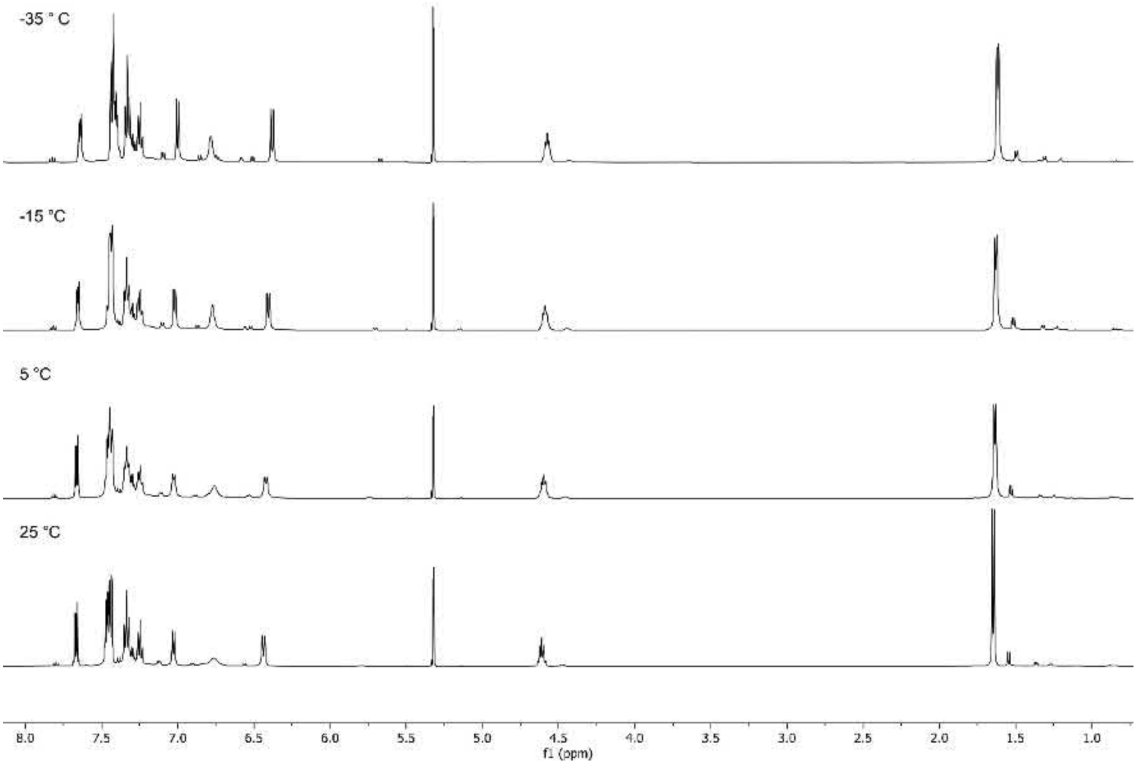

Figure S62. ^{1}H NMR of (*R*,*R*-**6**-Ag) at 25 °C, 5 °C, -15 °C, -35 °C. Upon cooling only one species is observed. The changes in the NMR spectrum are minor, with the sharpening of the 4’’’–H at 6.76 ppm being the most significant one.

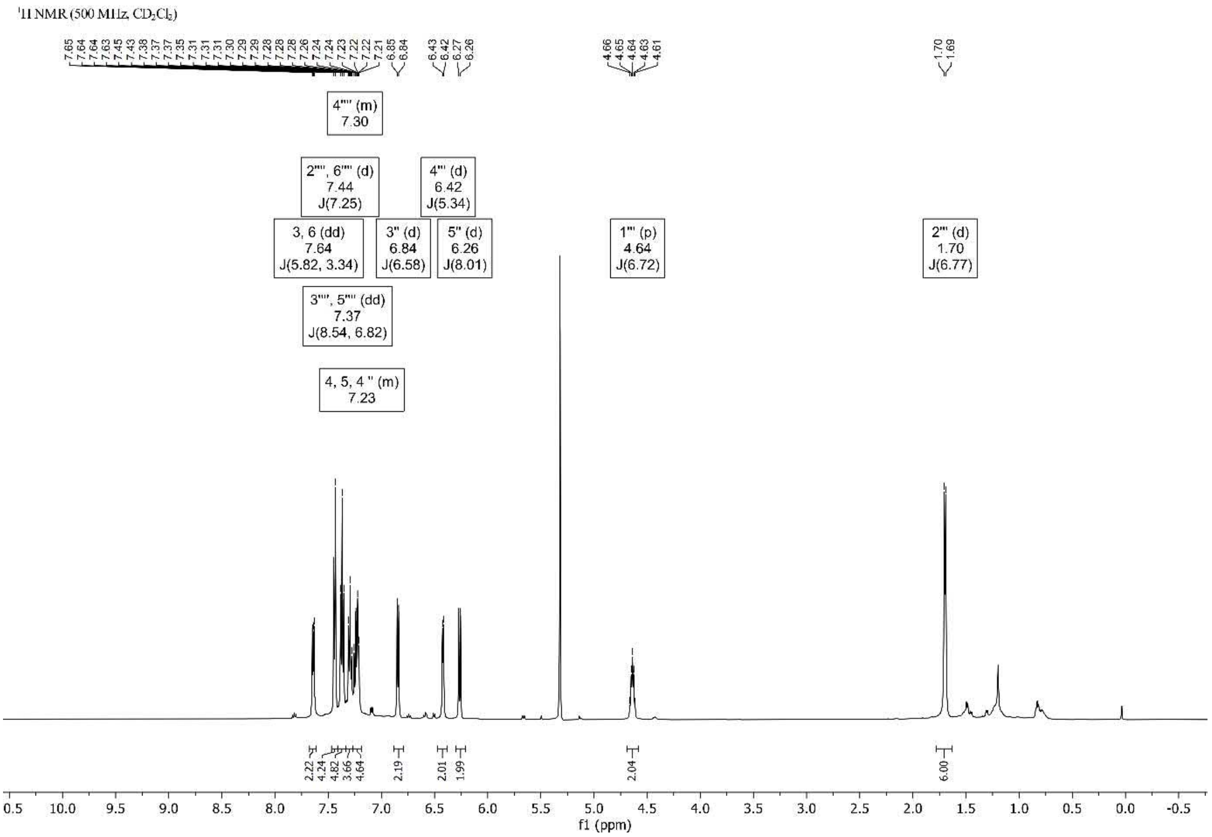

Figure S63. ^{1}H NMR spectrum of [(1,2-bis((6-(N-((1R)-1-phenylethyl)amino)pyridin-2-yl)ethinyl]benzene)iodine(I)] tetrafluoroborate (*R*,*R*-**I**-Ag) in CD_2Cl_2 at 25°C and 500 MHz.

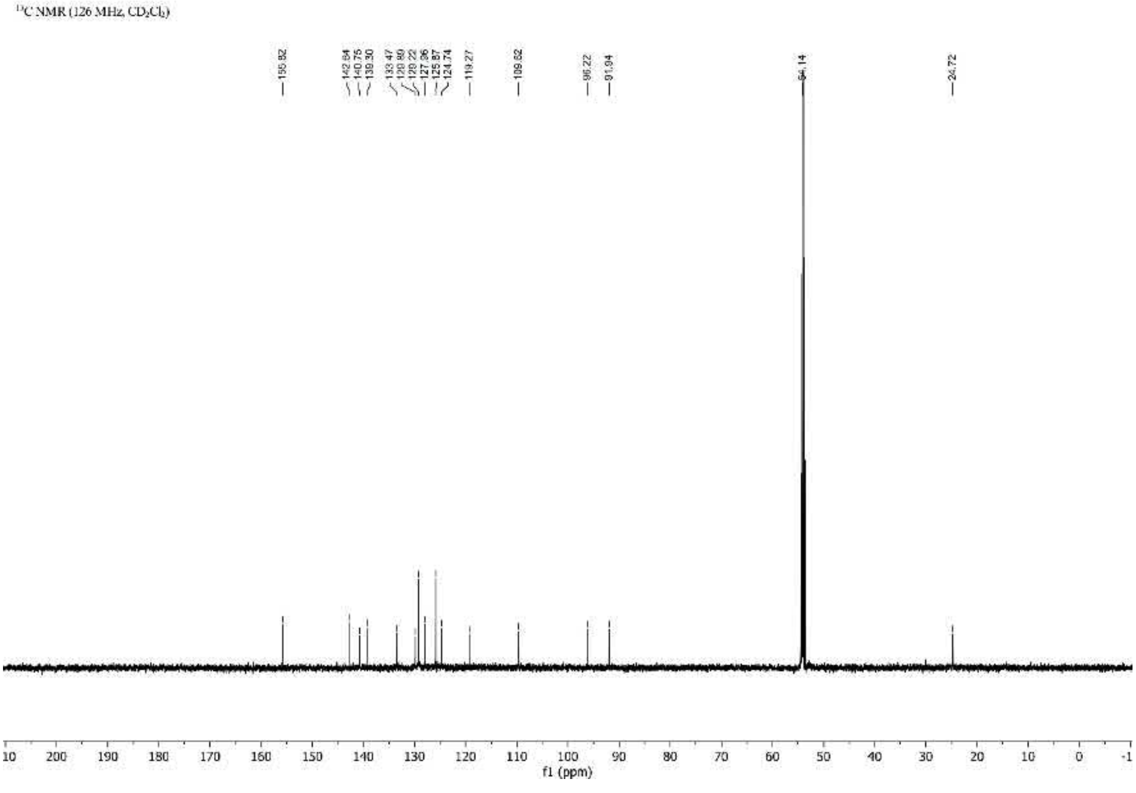

Figure S64. ^{13}C NMR spectrum of [(1,2-bis((6-(N-((1R)-1-phenylethyl)amino)pyridin-2-yl)ethinyl]benzene)iodine(I)] tetrafluoroborate (*R*,*R*-**6**-I) in CD_2Cl_2 at 25°C, and 126 MHz.

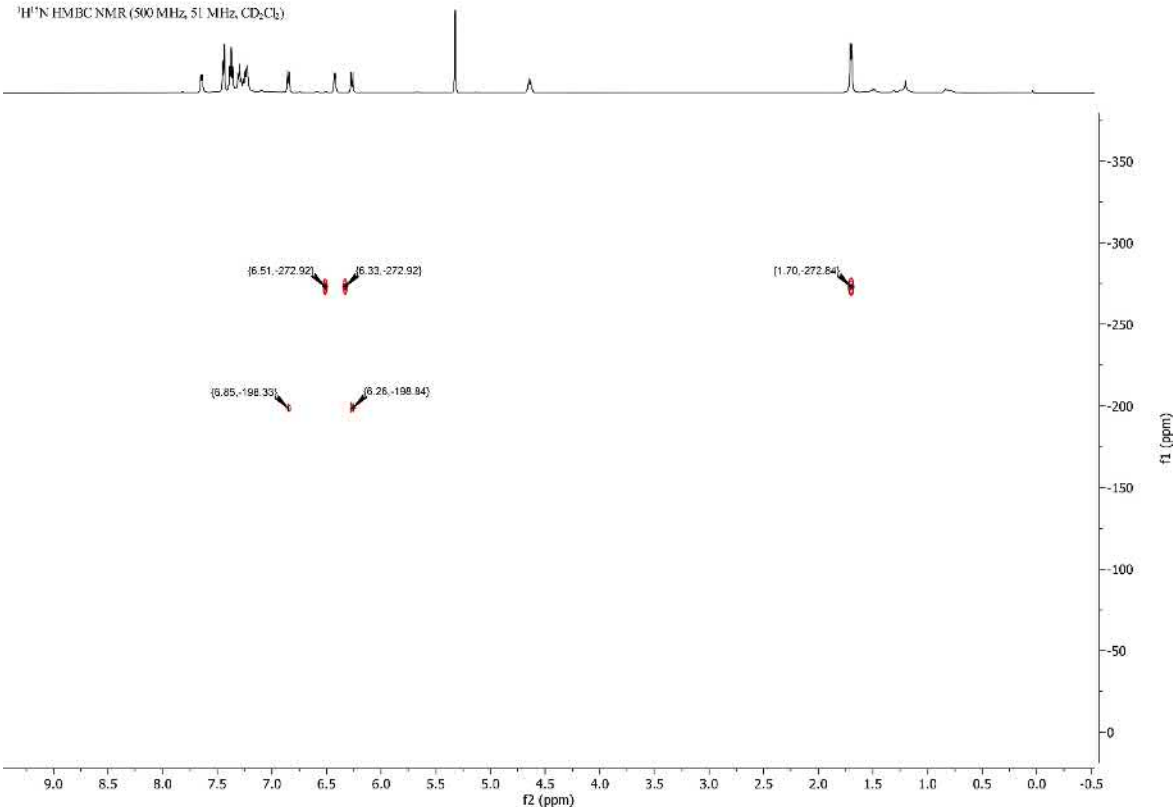

Figure S65. ^{1}H,^{15}N HMBC spectrum of [(1,2-bis((6-(N-((1R)-1-phenylethyl)amino)pyridin-2-yl)ethinyl]benzene)iodine(I)] tetrafluoroborate (*R*,*R*-**6**-I) in CD_2Cl_2 at 25°C (499.9/50.66 MHz).

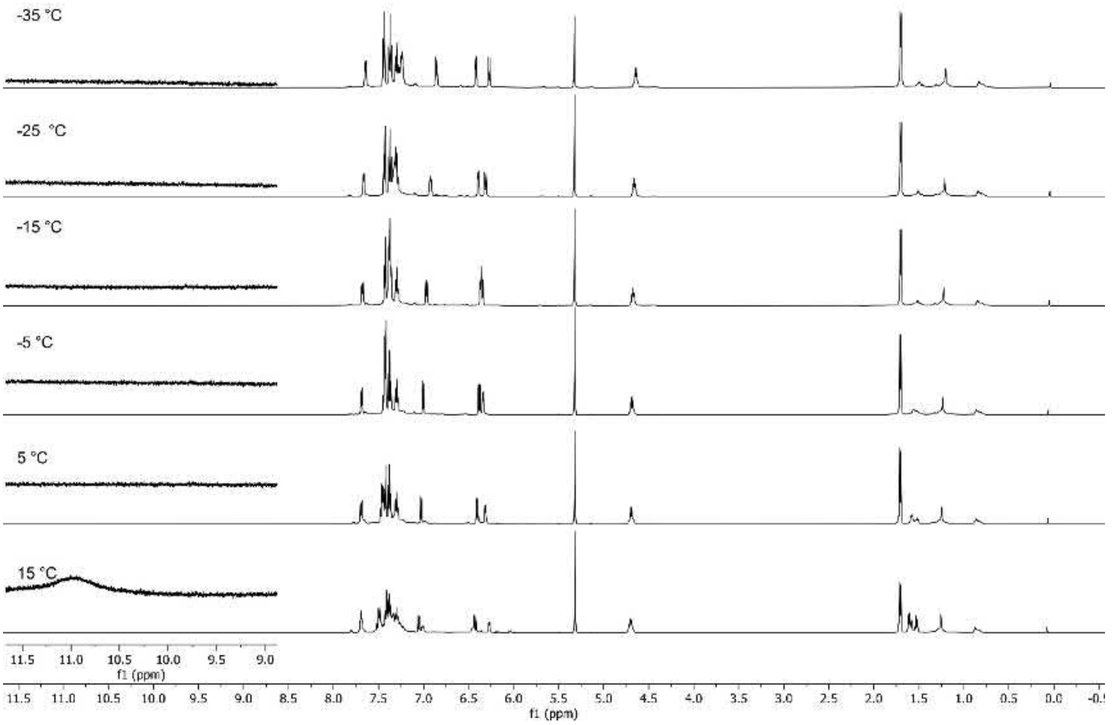

Figure S66. ^{1}H NMR of [(**6**)-I]BF_4 in CD_2Cl_2 at 15°C, 5°C, -5°C, -15°C, -25°C, -35 °C. Upon warmup, large shifts for the acidic NH-4'''' and pyridine ring protons 3''-H and 5''-H are observed. At 5 °C an increase in decomposition side product (1.53 ppm) is observed.

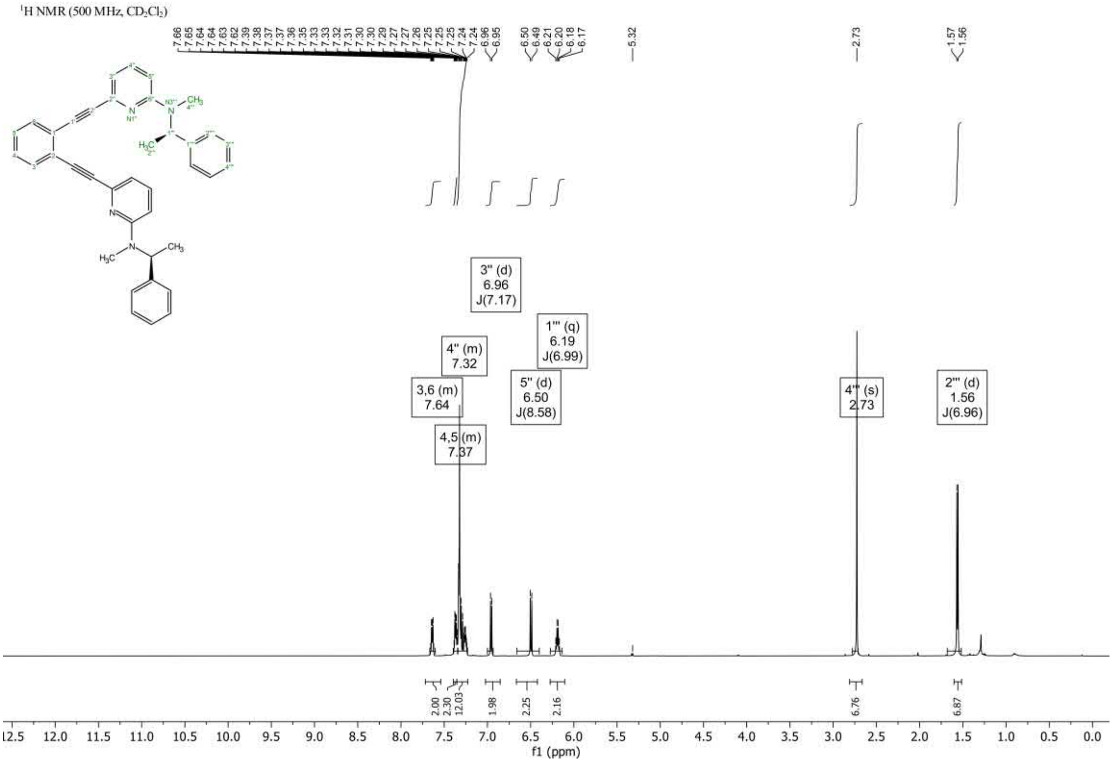

Figure S67. ^{1}H NMR spectrum of 1,2-bis((6-(*N*-methyl-*N*-((1R)-1-phenylethyl)amino)pyridin-2-yl)ethinyl]benzene (*R*,*R*-**7**) in CD_2Cl_2 at 25°C and 500 MHz.

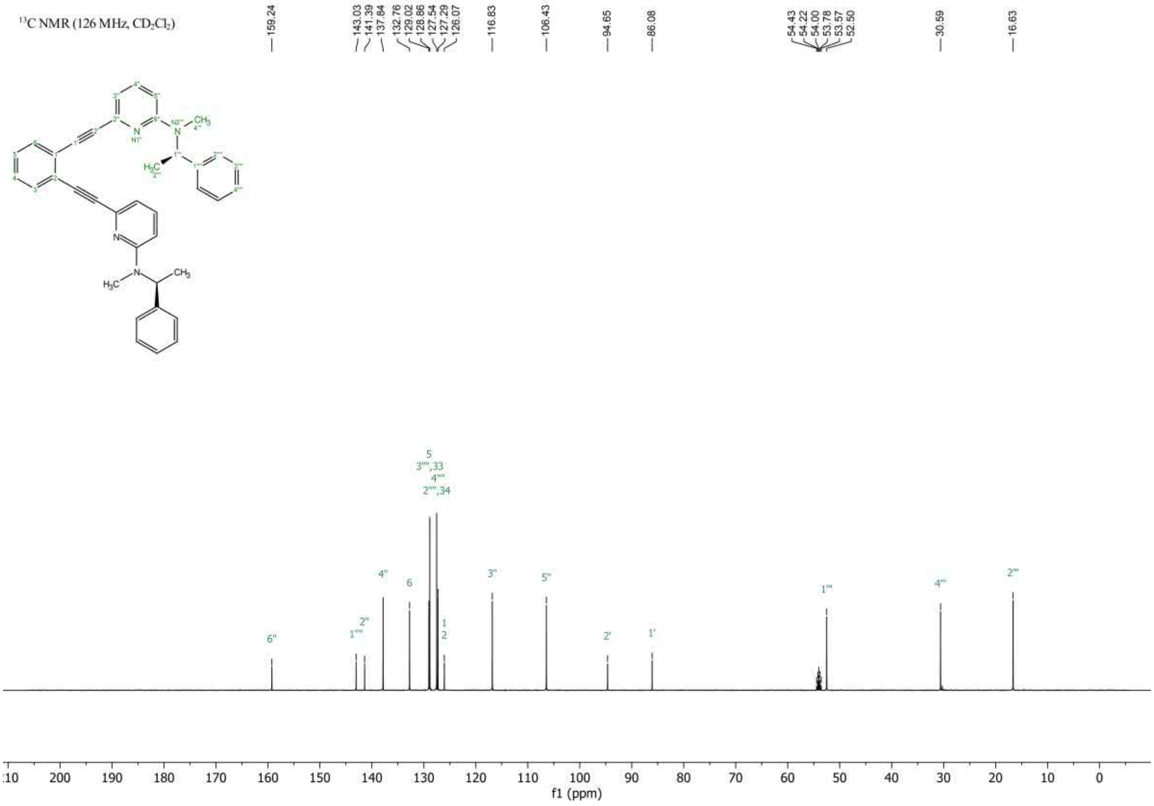

Figure S68. ^{13}C NMR spectrum of 1,2-bis((6-(*N*-methyl-*N*-((1R)-1-phenylethyl)amino)pyridin-2-yl)ethinyl]benzene (*R*,*R*-**7**) in CD_2Cl_2 at 25°C, and 126 MHz.

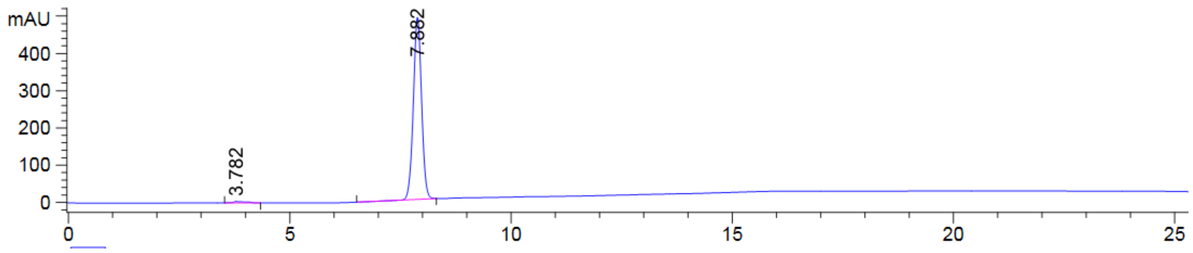

Figure S69. HPLC chromatogram of 1,2-bis((6-(*N*-methyl-*N*-((1*R*)-1-phenylethyl)amino)pyridin-2-yl)ethinyl]benzene (*R*,*R*-**7**), Analytical Lux® 5 µm i-Amylose-1, LC Column 250 x 4.6 mm, Hexane/iPrOH 90:10 to 20:80 [10min], 20:80 [10min] 0.75 ml/min. Single peak no shoulders R_t[enantiomer1] = 7.882 min.

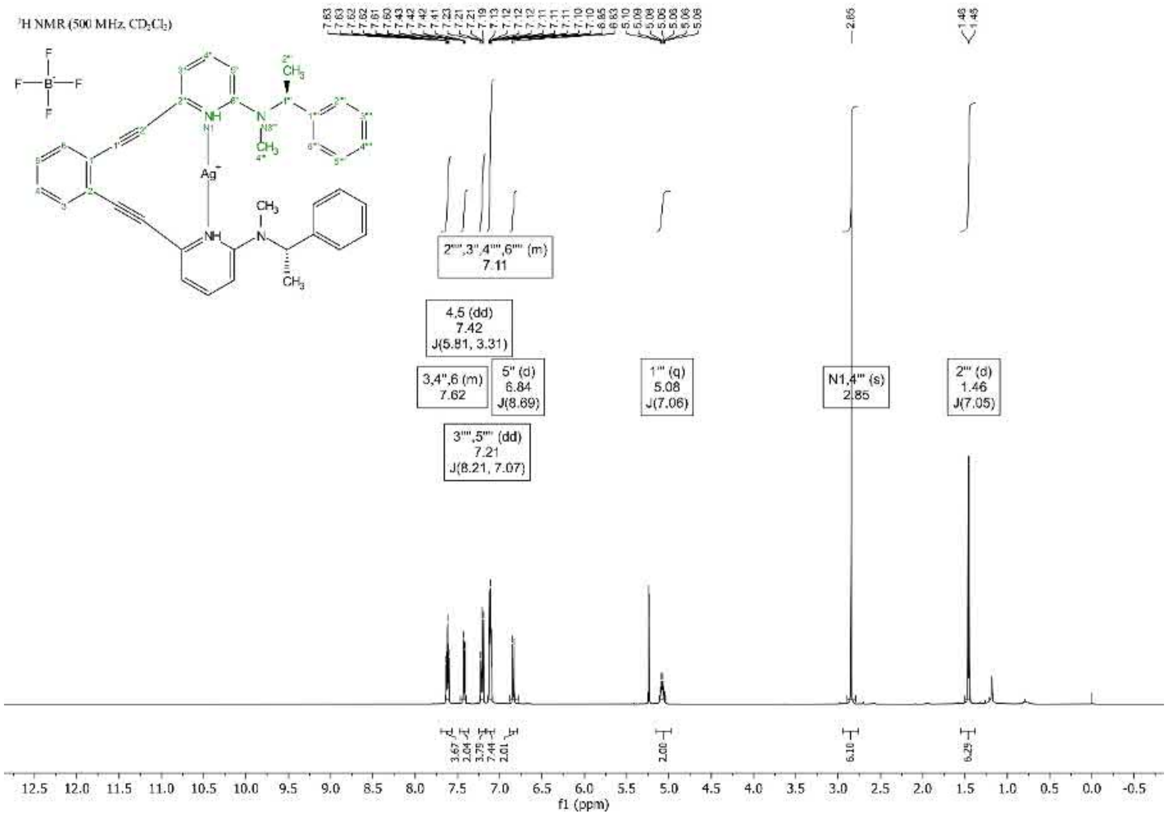

Figure S70. ^1H NMR spectrum of [(1,2-bis((6-(*N*-methyl-*N*-((1R)-1-phenylethyl)amino)pyridin-2-yl)ethinyl]benzene)silver(I)] tetrafluoroborate (*R*,*R*-**7**-Ag) in CD_2Cl_2 at 25°C and 500 MHz.

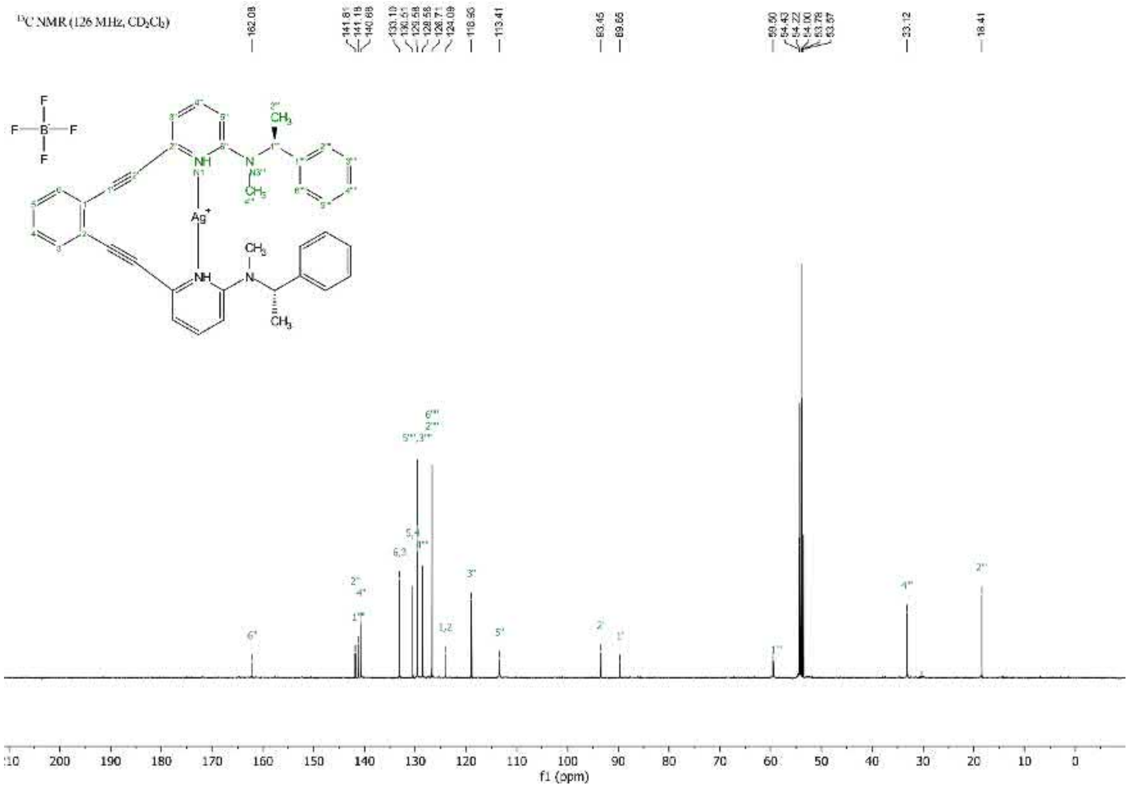

Figure S71. ^{13}C NMR spectrum of [(1,2-bis((6-(*N*-methyl-*N*-((1R)-1-phenylethyl)amino)pyridin-2-yl)ethinyl]benzene)silver(I)] tetrafluoroborate (*R*,*R*-**7**-Ag) in CD_2Cl_2 at 25°C, and 126 MHz.

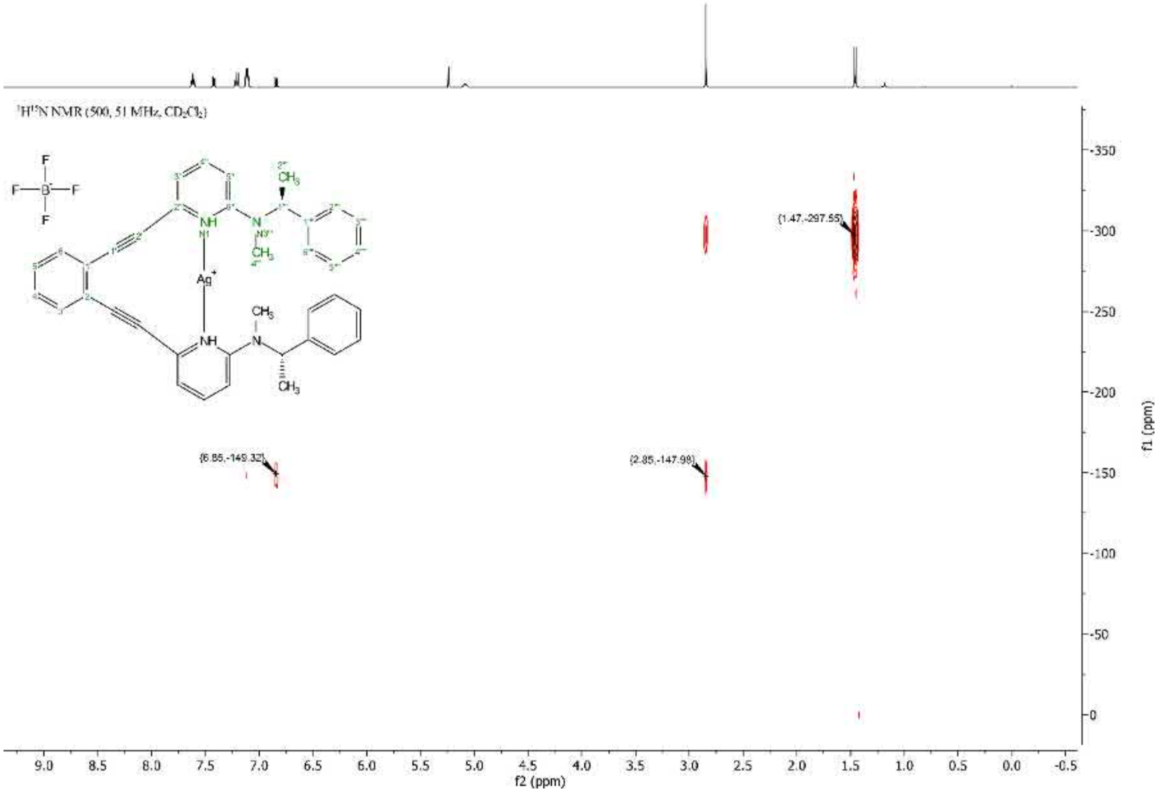

Figure S72. ^{1}H,^{15}N HMBC spectrum of [(1,2-bis((6-(*N*-methyl-*N*-((1R)-1-phenylethyl)amino)pyridin-2-yl)ethinyl]benzene)silver(I)] tetrafluoroborate (*R*,*R*-**7**-Ag) in CD_2Cl_2 at 25°C $(499.9/50.66\ \mathrm{MHz})$.

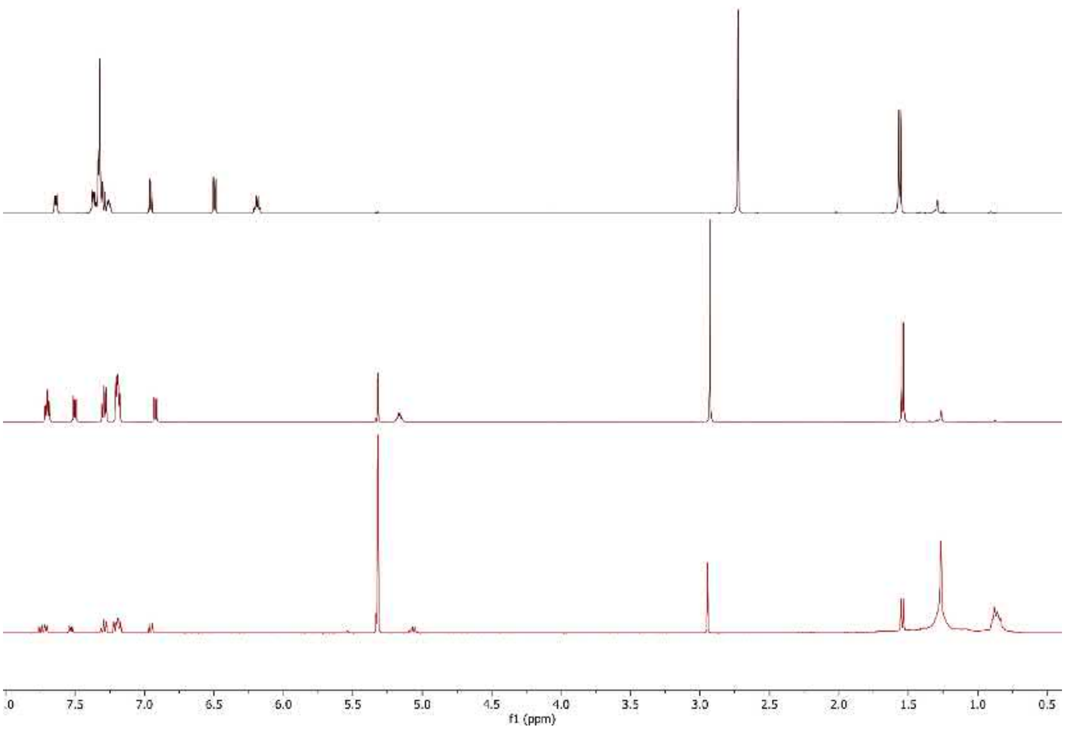

Figure S73. ^{1}H NMR spectra of *R*,*R*-**7** (top), *R*,*R*-**7**-Ag (0.01 mol L^{-1}) (middle), and *R*,*R*-**7**-Ag diluted 1:100 (~0.0001 mol L^{-1}) (bottom).

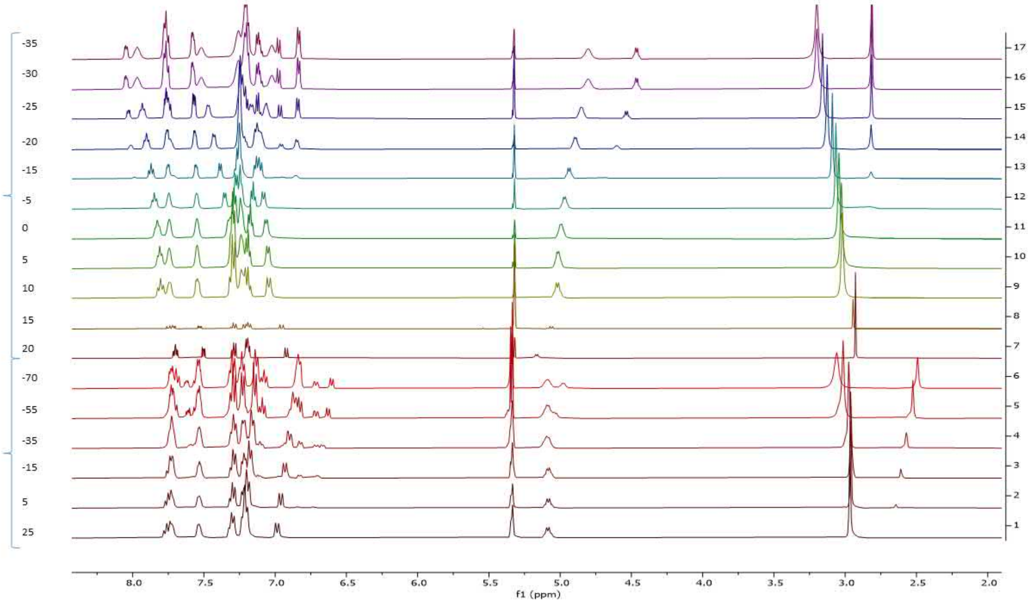

Figure S74. ^{1}H NMR spectra of *R*,*R*-**7**-Ag at different temperatures. Upon cooling, a new species and significant signal shifts are observed. Despite the slightly different chemical shifts of the new compound in both experiments, all features of the newly formed system are very similar, including peak shapes and peak order. The amount of new compound formed is significant and is unlikely to originate from balancing errors that leads to an excess of $AgBF_4$.

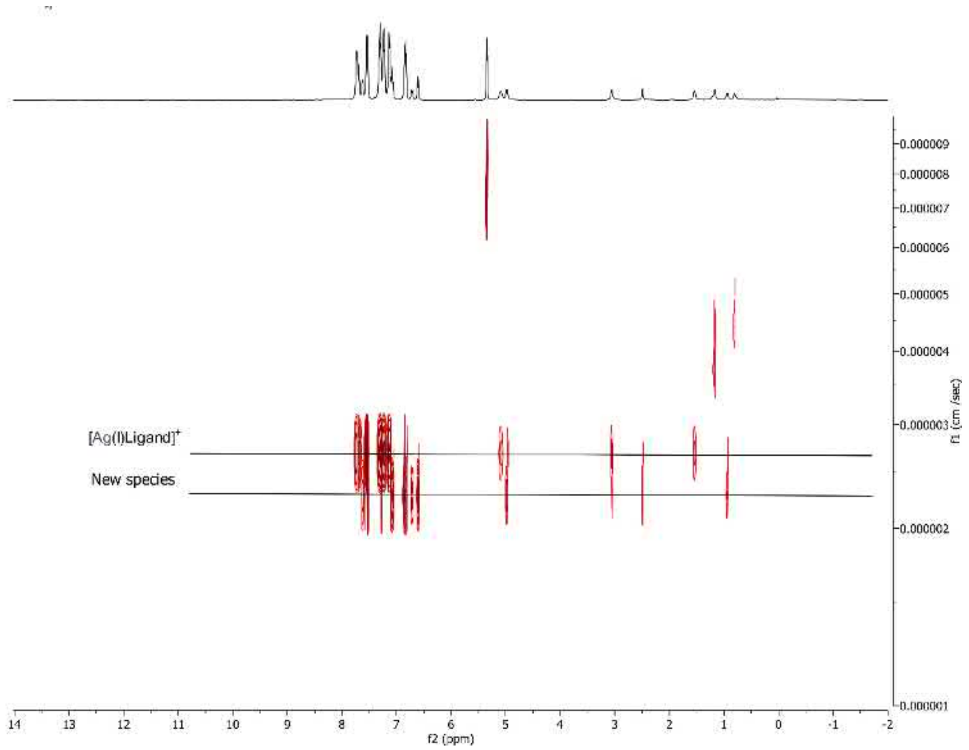

Figure S75. DOSY spectrum of the [1]of [(1,2-bis((6-(*N*-methyl-*N*-((1R)-1-phenylethyl)amino)pyridin-2-yl)ethinyl]benzene)silver(I)] tetrafluoroborate (*R*,*R*-**7**-Ag) measured at -70°C in CD_2Cl_2. Diffusion coefficient of *R*,*R*-**7**-Ag (2.68 10^{-10} m^2s^{-1}), and of the new species: 2.28 10^{-10} m^2s^{-1-} at -70℃ (499.9 MHz).

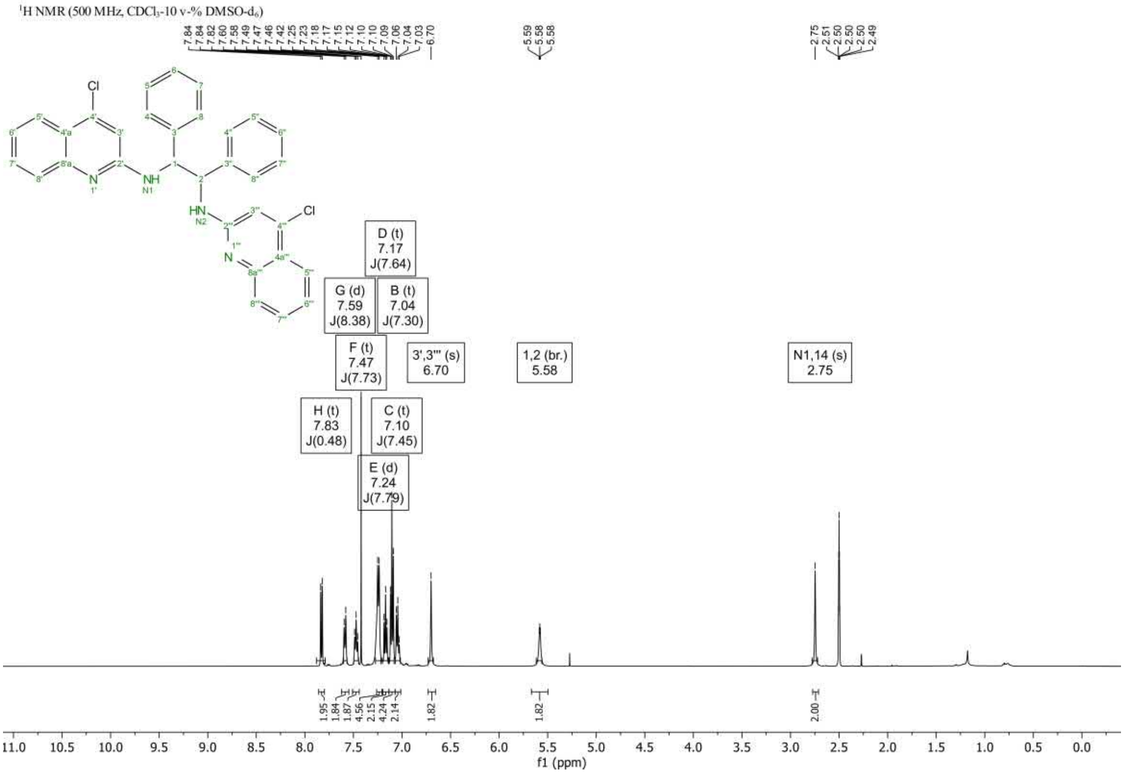

Figure S76. ^{1}H NMR spectrum of (1*R*,2*R*)-*N*[1],*N*[2]-bis(4-chloroquinolin-2-yl)-1,2-diphenylethane-1,2-diamine (*R*,*R*-**8**) in $CDCl_3$ 10 *v*/*v*-% DMSO at 25°C and 500 MHz.

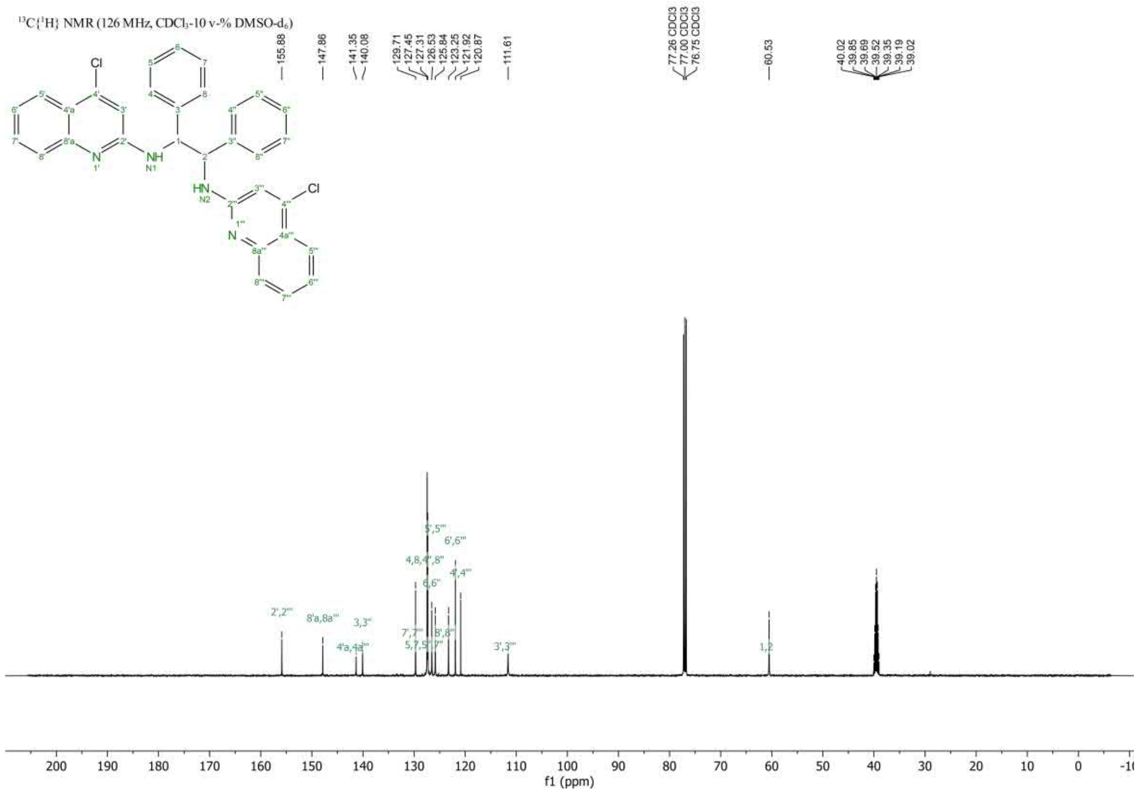

Figure S77. ^{13}C NMR spectrum of (1*R*,2*R*)-*N*[1],*N*[2]-bis(4-chloroquinolin-2-yl)-1,2-diphenylethane-1,2-diamine (*R*,*R*-**8**) in $CDCl_3$ 10 *v*/*v*-% DMSO at 25°C, and 126 MHz.

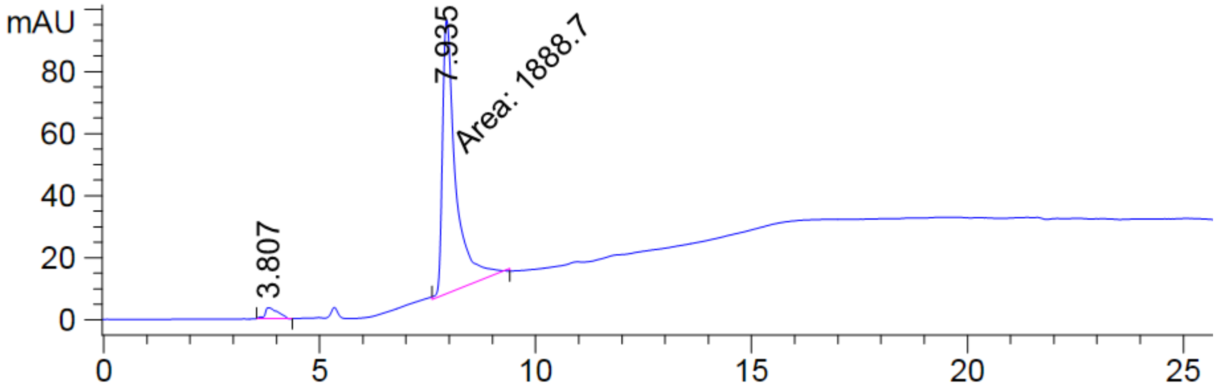

Figure S78. HPLC chromatogram of (1*R*,2*R*)-*N*[1],*N*[2]-bis(4-chloroquinolin-2-yl)-1,2-diphenylethane-1,2-diamine (*R*,*R*-**8**), Analytical Lux® 5 µm i-Amylose-1, LC Column 250 x 4.6 mm, hexane/*i*PrOH 90:10 to 20:80 [10min], 20:80 [10min] 0.75 ml/min. single peak R_t[enantiomer] = 7.935 min).

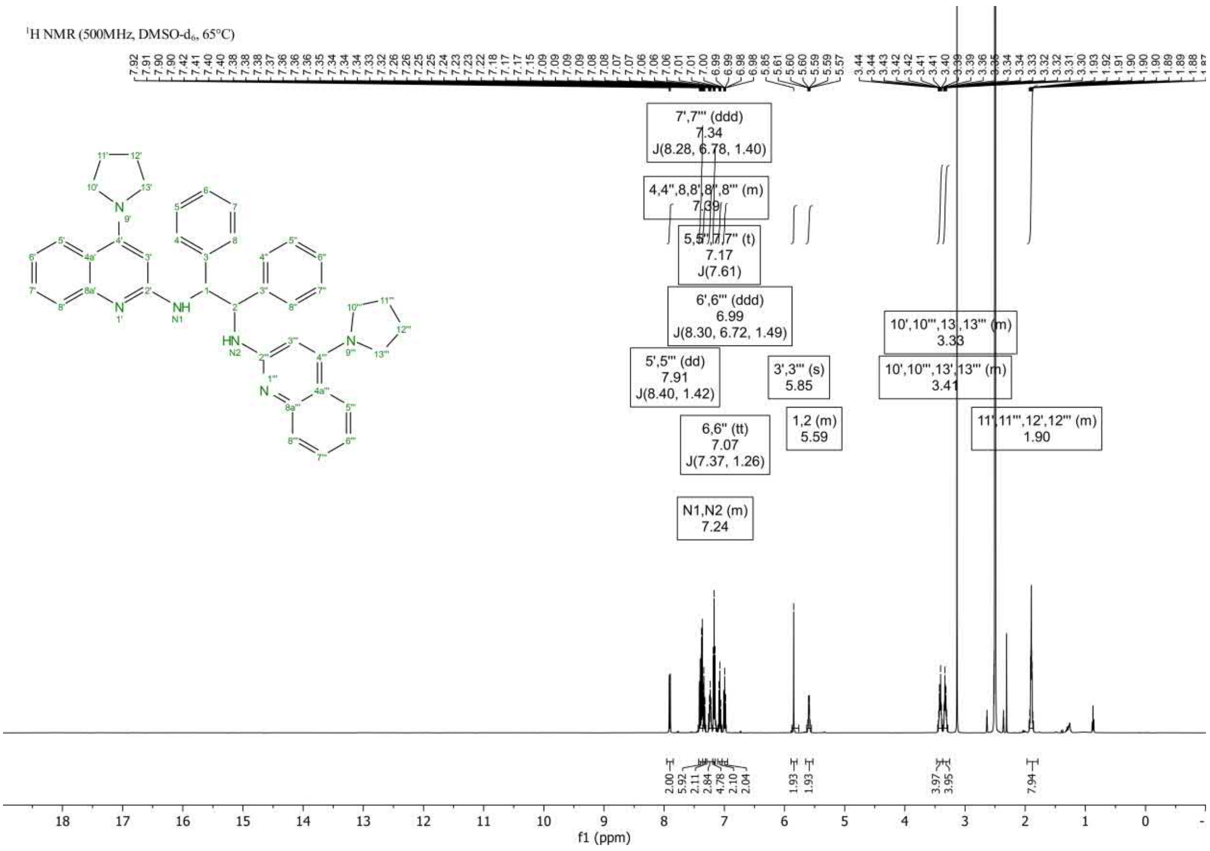

Figure S79. ^{1}H NMR spectrum of (1*R*,2*R*)-*N*^{1},*N*^{2}-bis(4-(pyrrolidin-1-yl)quinolin-2-yl)ethane-1,2-diphenyl-1,2-diamine (*R*,*R*-***9***) in DMSO-d6 at 65°C and 500 MHz.

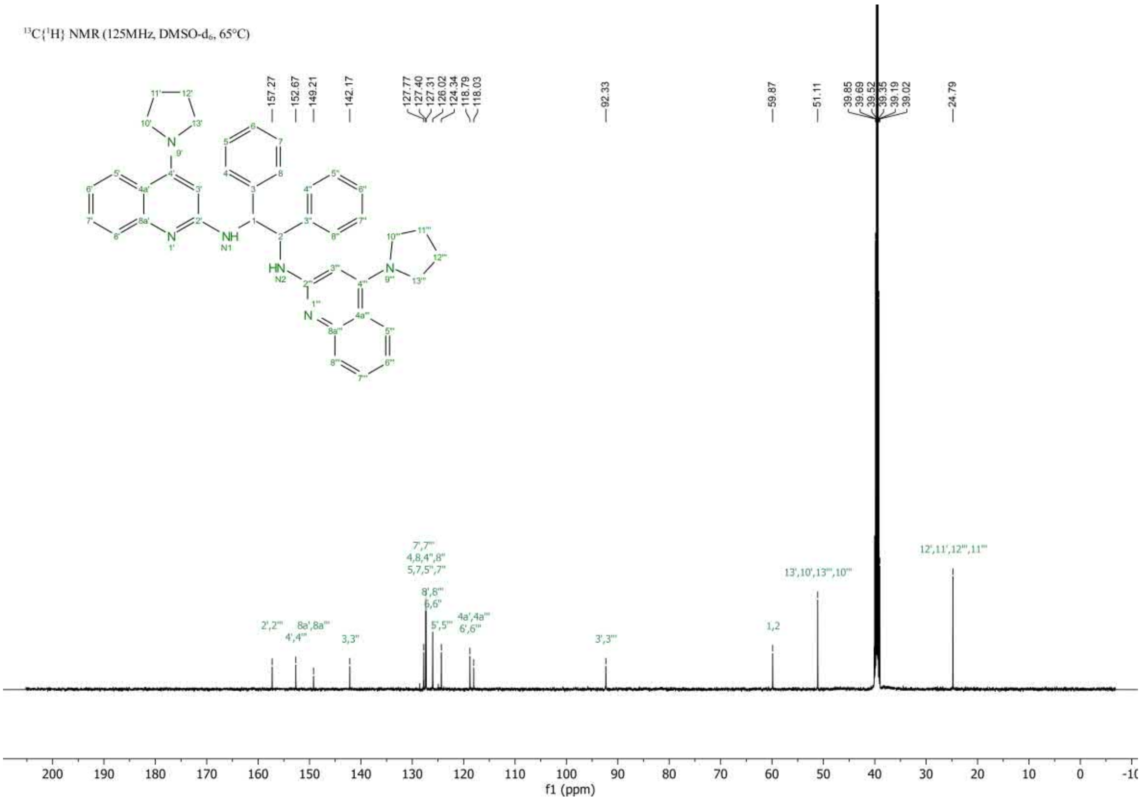

Figure S80. ^{13}C NMR spectrum of ((1*R*,2*R*)-*N*^{1},*N*^{2}-bis(4-(pyrrolidin-1-yl)quinolin-2-yl)ethane-1,2-diphenyl-1,2-diamine (*R*,*R*-***9***) in DMSO-d6 at 65°C, and 126 MHz.

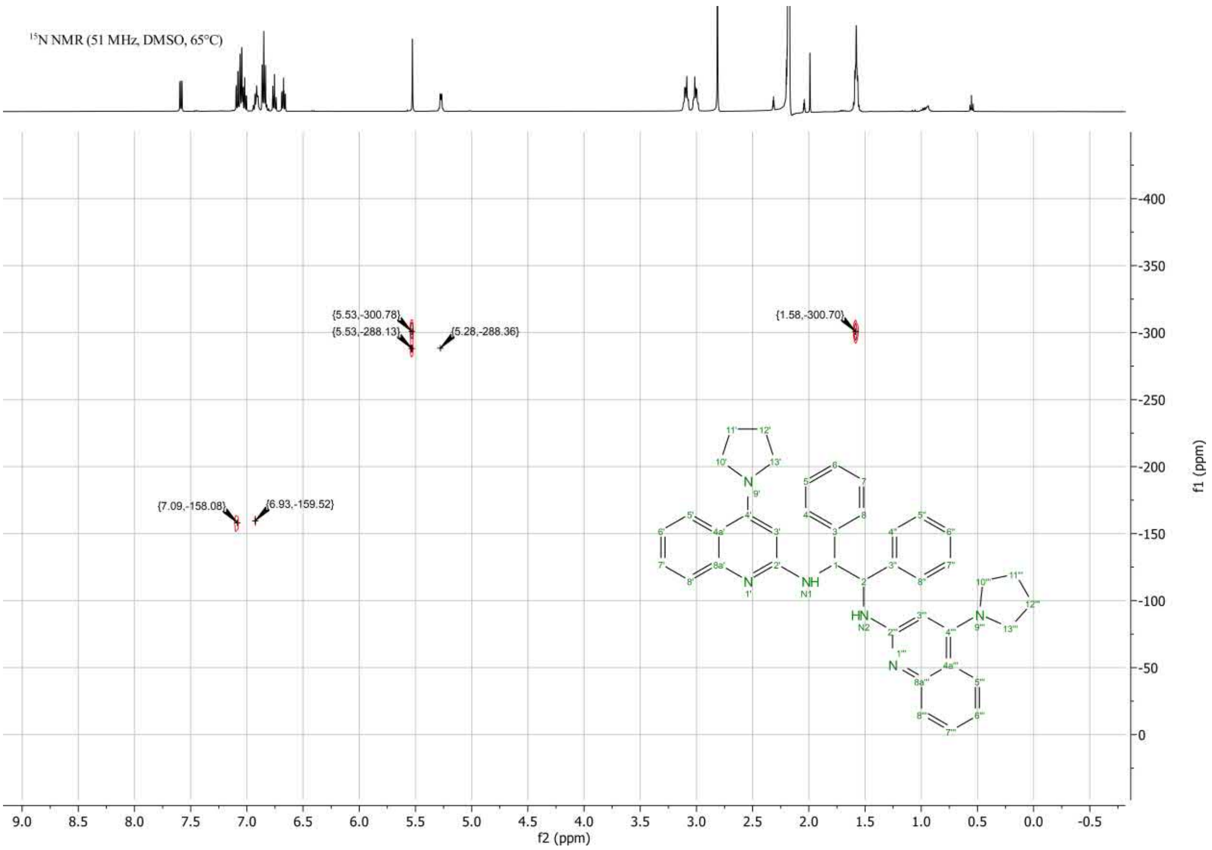

Figure S81. $^{1}H^{15}N$ NMR spectrum (1*R*,2*R*)-*N*[1],*N*[2]-bis(4-(pyrrolidin-1-yl)quinolin-2-yl)ethane-1,2-diphenyl-1,2-diamine (R,R-***9***) in DMSO-d6 at 65℃, and 126 MHz.

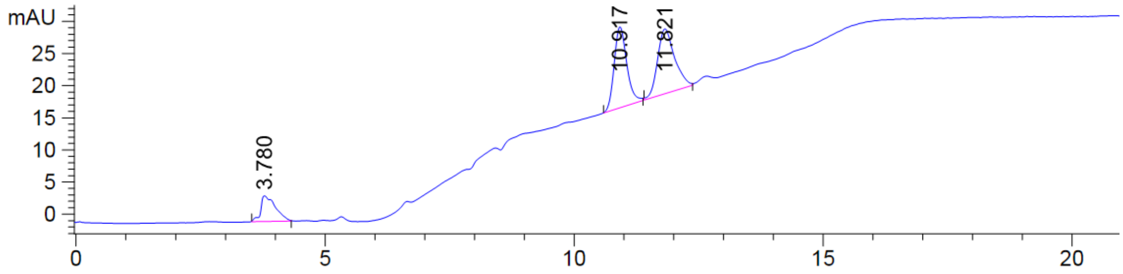

Figure S82. HPLC chromatogram of (1*R*,2*R*)-*N*[1],*N*[2]-bis(4-(pyrrolidin-1-yl)quinolin-2-yl)ethane-1,2-diphenyl-1,2-diamine (R,R-***9***), Analytical Lux® 5 µm i-Amylose-1, LC Column 250 x 4.6 mm, Hexane/iPrOH 90:10 to 20:80 [10min], 20:80 [10min] 0.75 ml/min. Semi stable conformers at r.t. R_t[conformer1-enantiomer1] = 10.917 min 48%, R_t[conformer2-enantiomer1] = 11.821 min 52%.

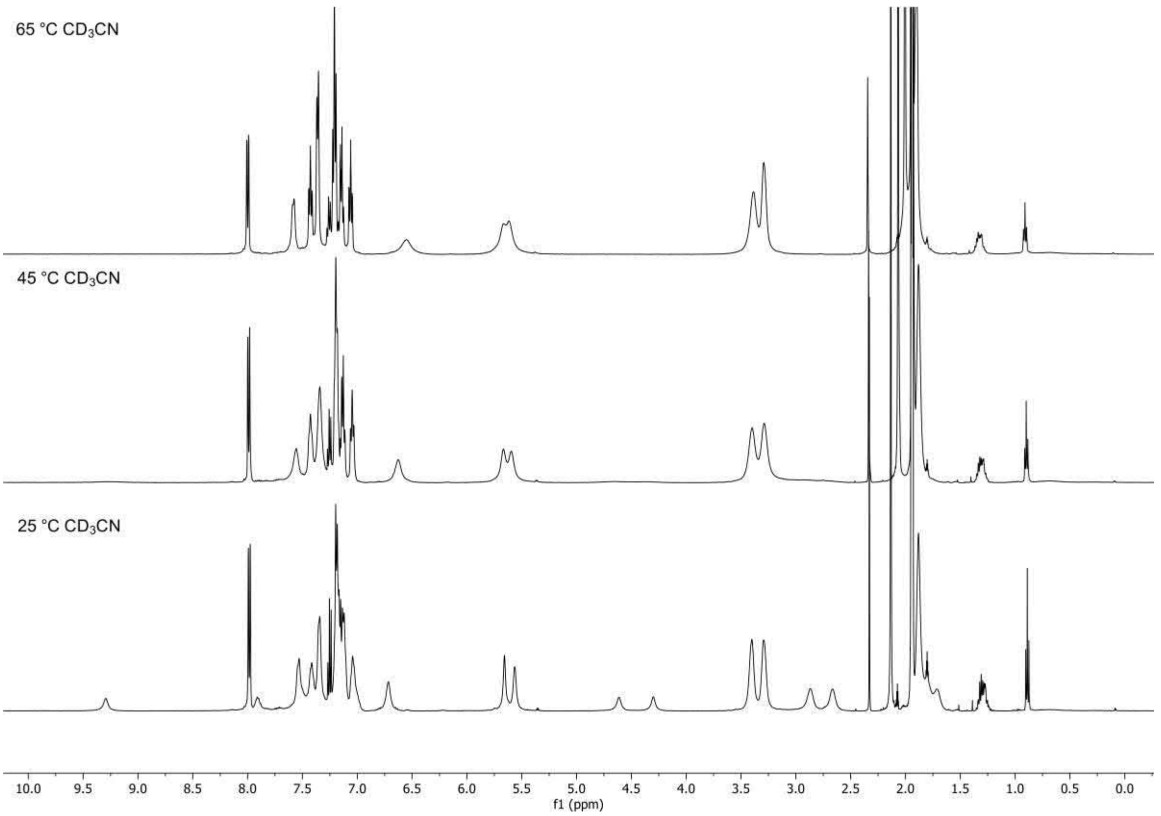

Figure S83. ^{1}H NMR spectra of crystalized (1*R*,2*R*)-*N*^{1},*N*^{2}-bis(4-(pyrrolidin-1-yl)quinolin-2-yl)ethane-1,2-diphenyl-1,2-diamine (R,R-***9***) in CD_3CN at 25°C, 45°C, 65 °C and 500 MHz. Revealing an set of isomers, that are in fast exchange at higher temperatures excluding a 1:1 mixture of enantiomers.

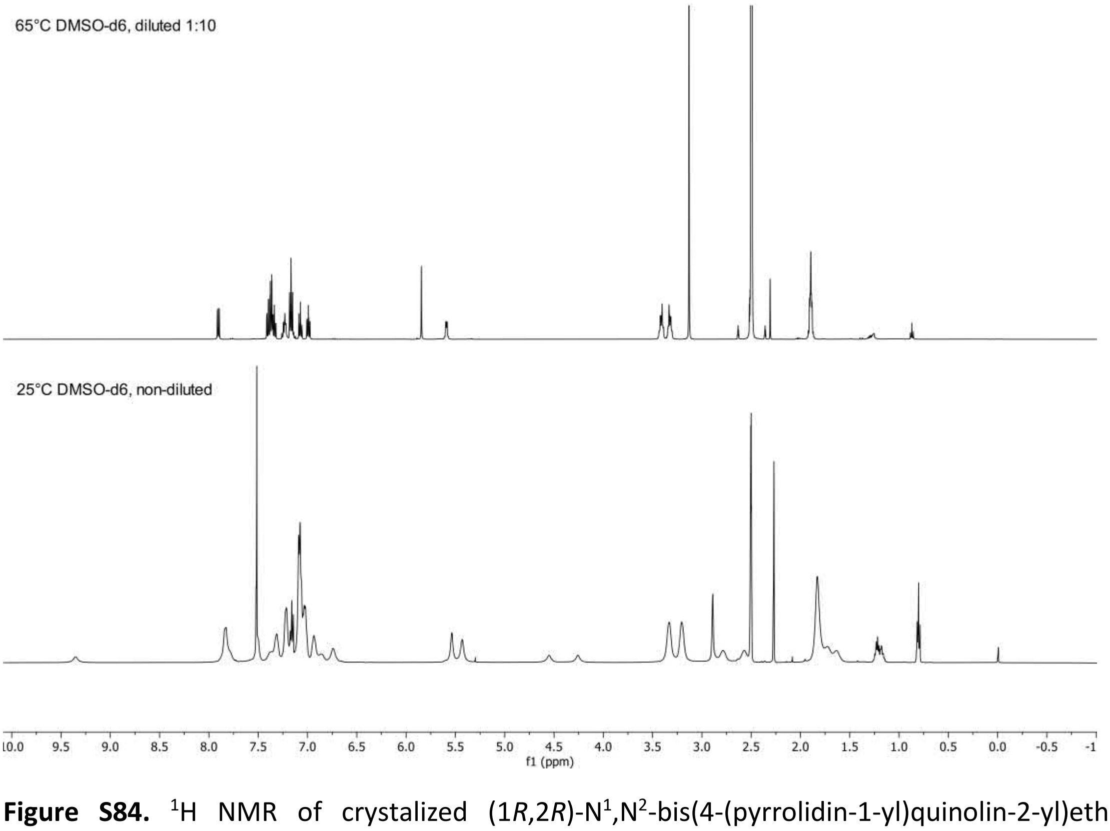

Figure S84. ^{1}H NMR of crystalized (1*R*,2*R*)-N^1,N^2-bis(4-(pyrrolidin-1-yl)quinolin-2-yl)ethane-1,2-diphenyl-1,2-diamine (R,R-***9***) in DMSO-d_6 at 25°C, and it's 1:10 dilution at 65 °C. The dilution has

been necessary to avoid signal broadening. Based on these data we speculated about a hindered rotation around the C1–C2-bond as the origin for the observed effects supported by intramolecular hydrogen bonding based on the increased basicity of the quinolin moieties. This would explain the strong shift of a hydrogen bonded NHAr proton at 9.4 ppm and the strongly effected C1–H and C2–H resonances at 4.5 and 4.25 ppm compared to ~5.5 ppm. The significant shift observed for the pyrrolidine protons at 2.8 ppm and 2.55 ppm can support the participation of the quinolin moiety in mesmeric stabilisation.

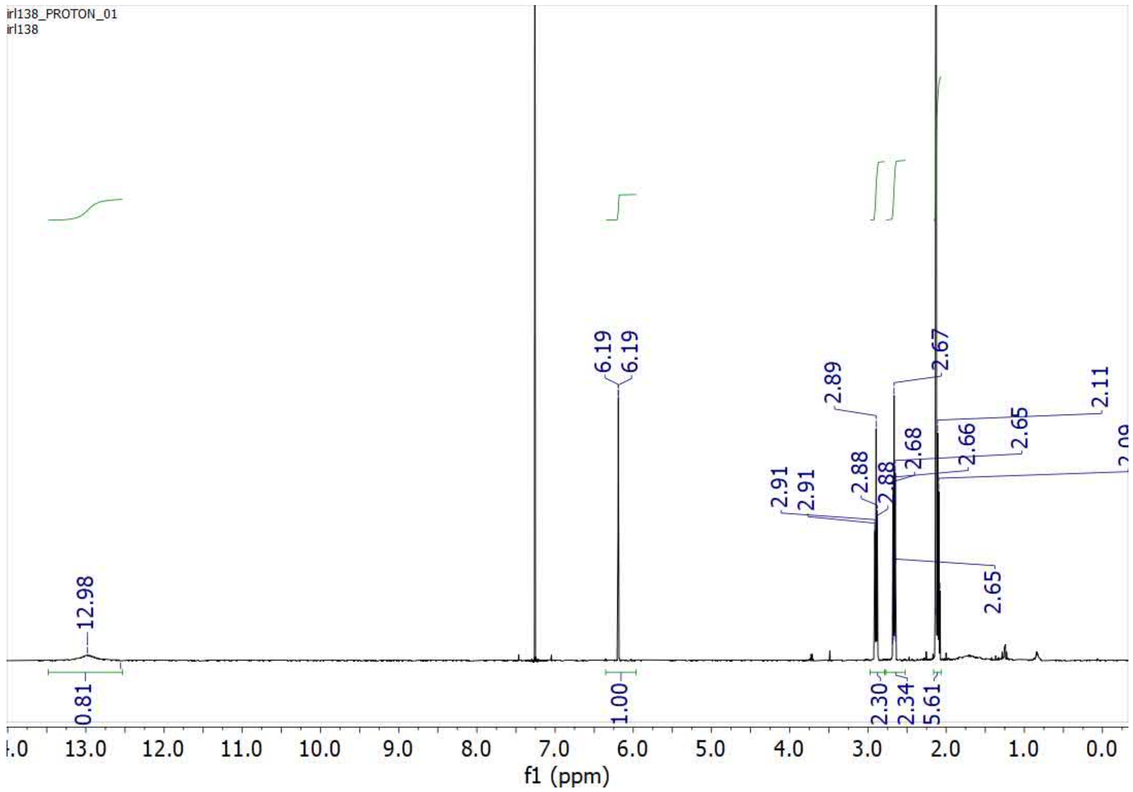

Figure S85. ^{1}H NMR spectrum of 4-methyl-6,7-dihydro-*5H*-cyclopenta[*b*]pyridin-2-ol (**10**) in $CDCl_3$ at 25℃ (499.9 MHz).

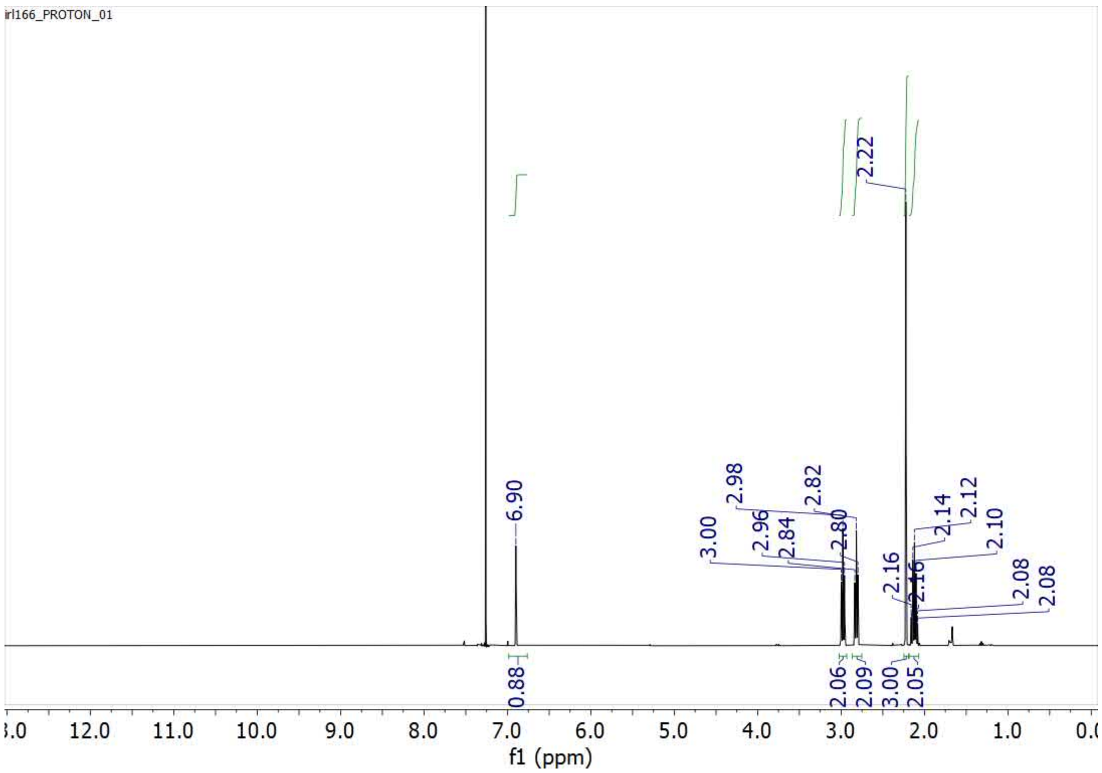

Figure S86. ^{1}H NMR spectrum of 2-chloro-4-methyl-6,7-dihydro-*5H*-cyclopenta[b]pyridine (**11**) in $CDCl_3$ at 25℃ (499.9 MHz).

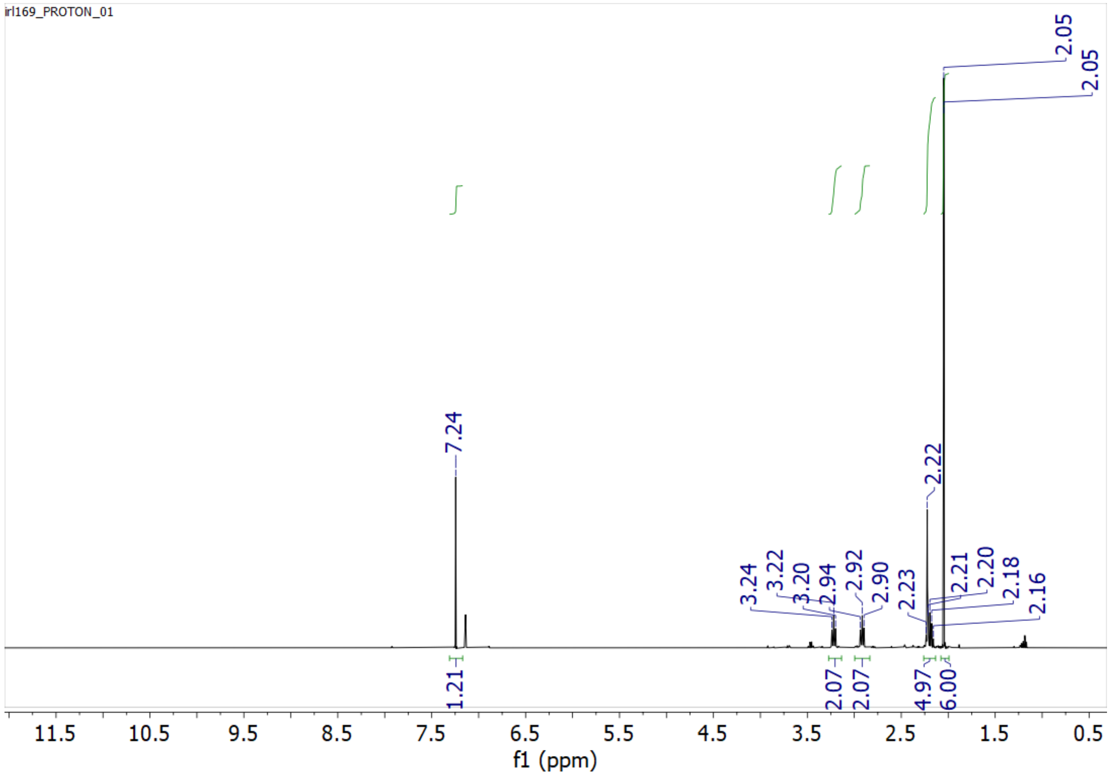

Figure S87. ^{1}H NMR spectrum of 2-chloro-4-methyl-6,7-dihydro-5H-cyclopenta[b]pyridin-7-yl acetate (**12**) in $CDCl_3$ at 25℃ (499.9 MHz).

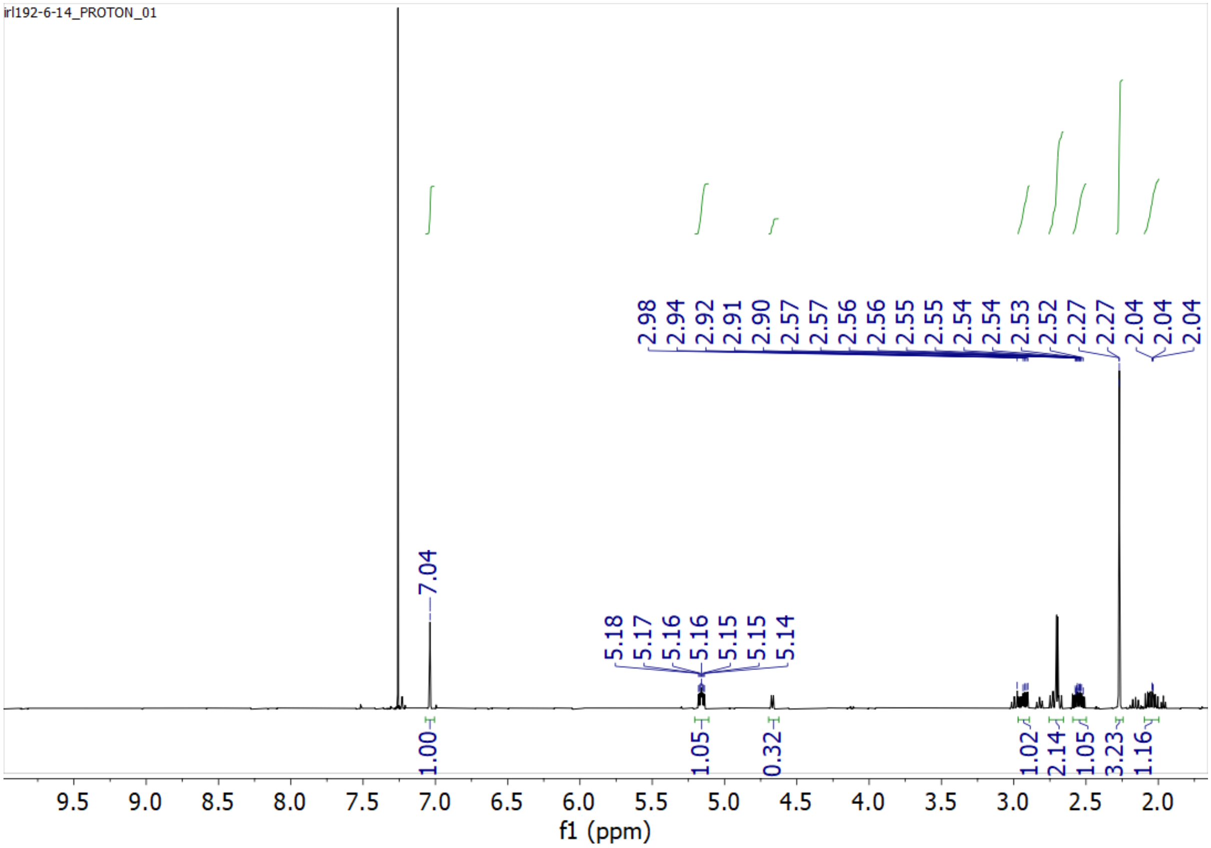

Figure S88. ^{1}H NMR spectrum of 2-chloro-4-methyl-6,7-dihydro-5H-cyclopenta[b]pyridin-7-ol (**13**) in $CDCl_3$ at 25°C (499.9 MHz).

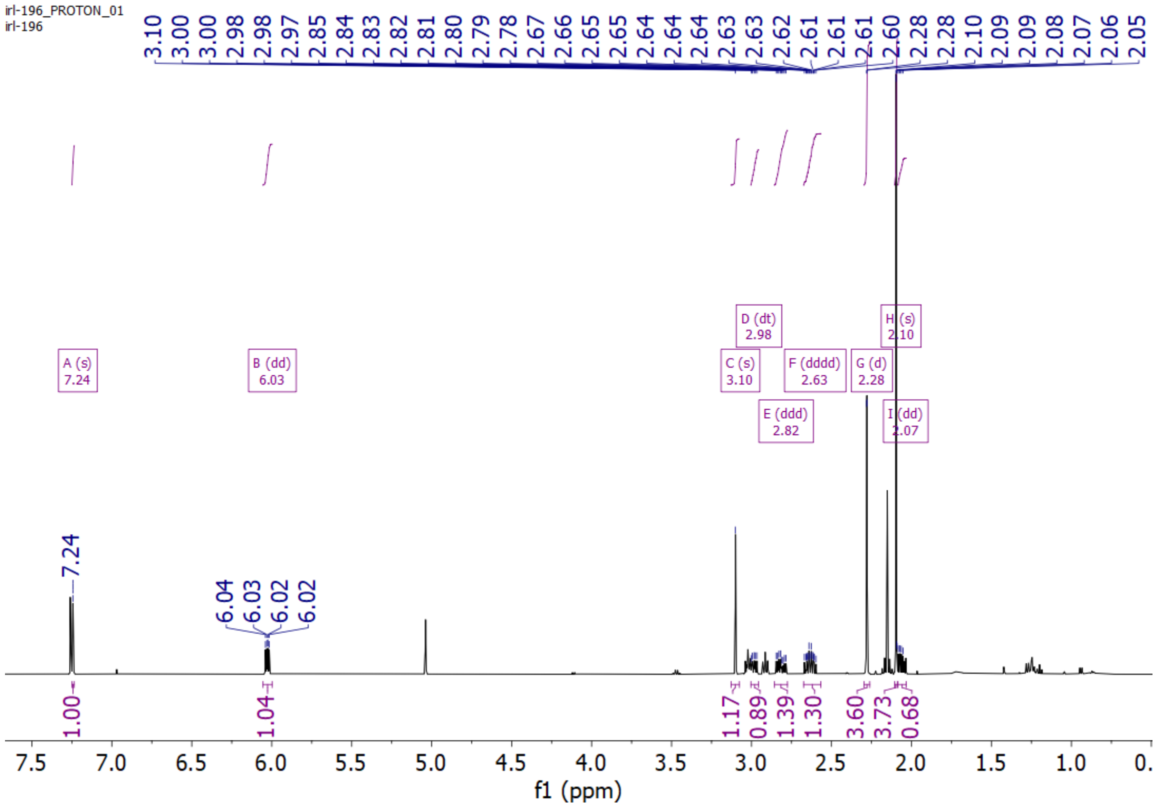

Figure S89. ^{1}H NMR spectrum of (*R*)-2-ethynyl-6,7-dihydro-5H-cyclopenta[b]pyridin-7-yl acetate (**14**) in $CDCl_3$ at 25°C (499.9 MHz).

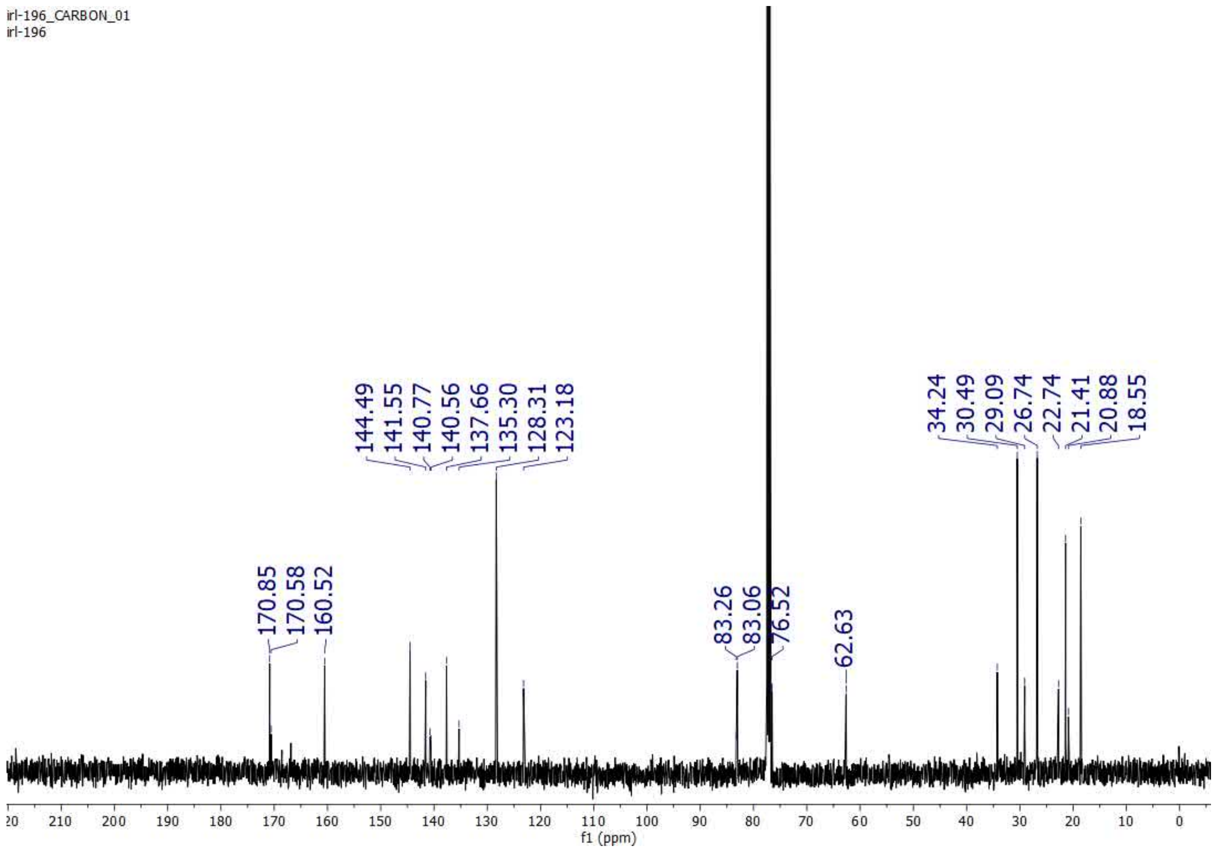

Figure S90. ^{13}C NMR spectrum of (*R*)-2-ethynyl-6,7-dihydro-5H-cyclopenta[b]pyridin-7-yl acetate (**14**) in $CDCl_3$ at 25°C (125.7 MHz).

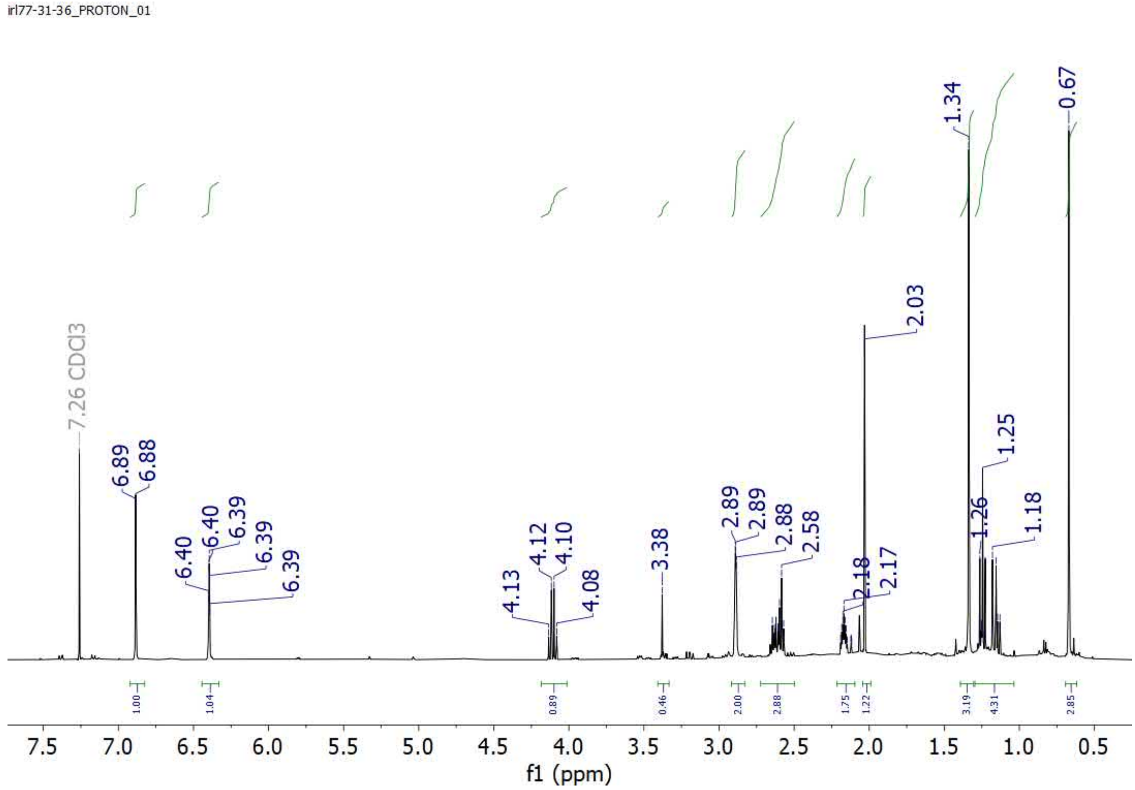

Figure S91. ^{1}H NMR spectrum of (6*R*,8*S*)-(-)-5,6,7,8-Tetrahydro-7,7-dimethyl-6,8-methanoisoquinolin-3-ol (**15**) in $CDCl_3$ at 25°C (399.9 MHz).

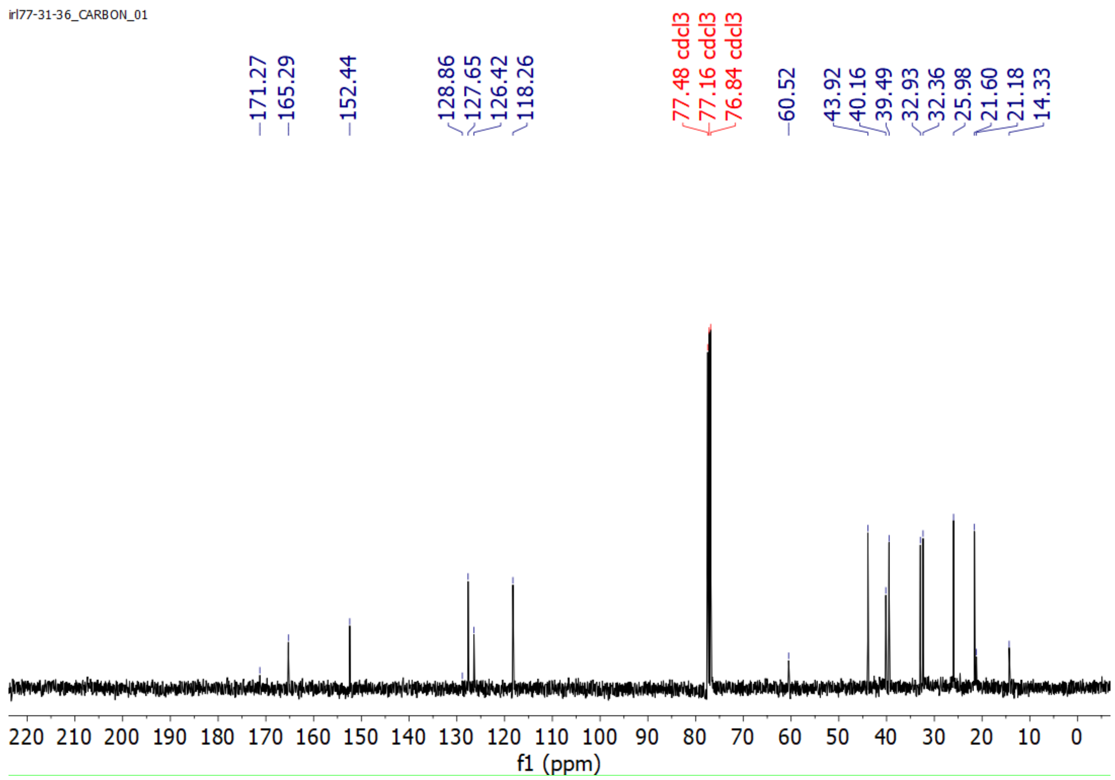

Figure S92. ^{13}C NMR spectrum of (6*R*,8*S*)-(-)-5,6,7,8-Tetrahydro-7,7-dimethyl-6,8-methanoisoquinolin-3-ol (**15**) in $CDCl_3$ at 25°C (100.6 MHz).

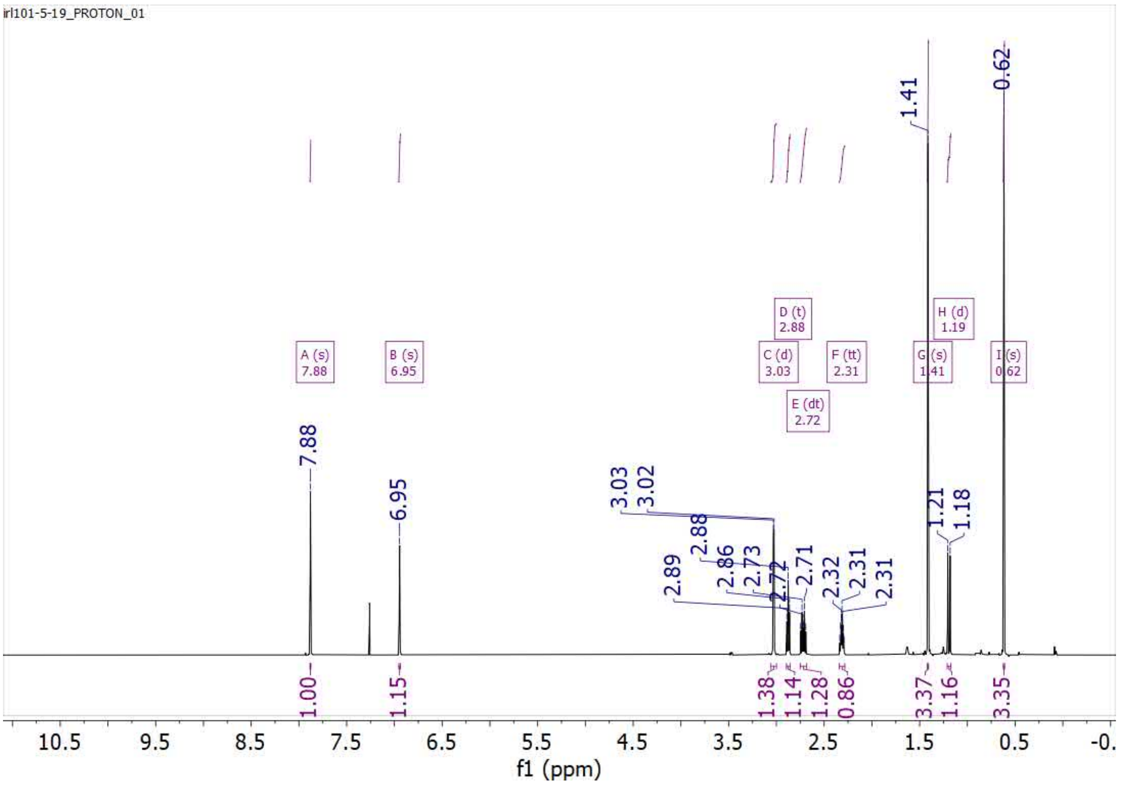

Figure S93. ^{1}H NMR spectrum (6*R*,8*S*)-(-)-5,6,7,8-tetrahydro-7,7-dimethyl-6,8-methano-isoquinolin-3-yl trifluoromethanesulfonate (**16**) in $CDCl_3$ at 25°C (399.9 MHz).

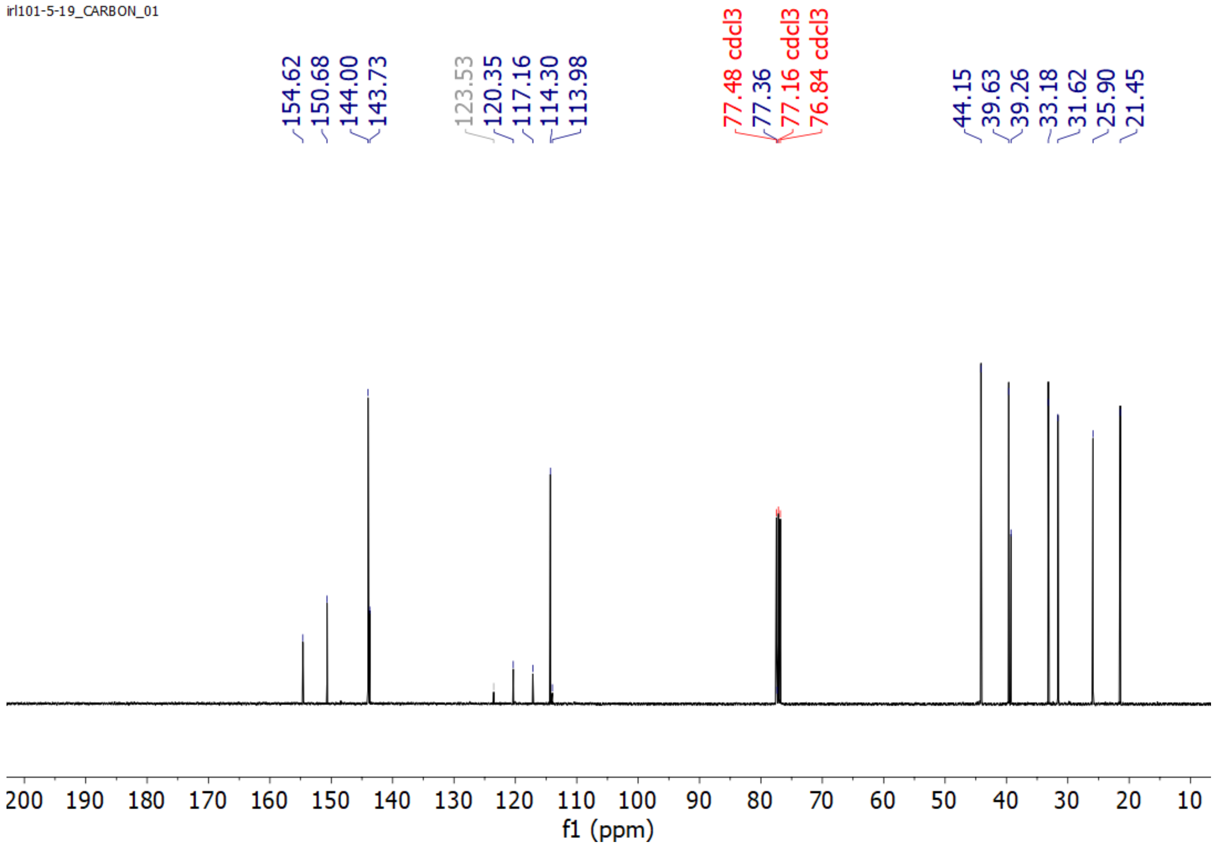

Figure S94. ^{13}C NMR spectrum of (6*R*,8*S*)-(-)-5,6,7,8-tetrahydro-7,7-dimethyl-6,8-methano-isoquinolin-3-yl trifluoromethanesulfonate (**16**) in $CDCl_3$ at 25°C (100.6 MHz).

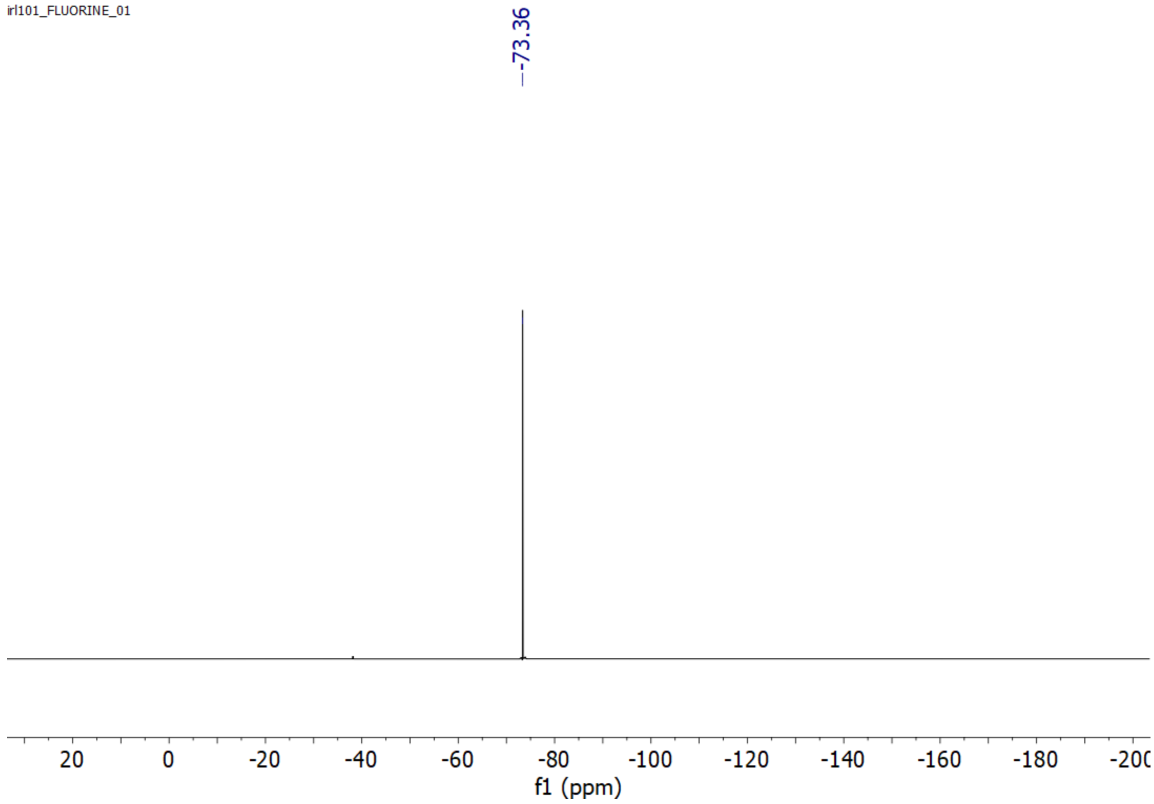

Figure S95. ^{19}F NMR spectrum (6*R*,8*S*)-(-)-5,6,7,8-tetrahydro-7,7-dimethyl-6,8-methano-isoquinolin-3-yl trifluoromethanesulfonate (**16**) in $CDCl_3$ at 25°C (376.3 MHz).

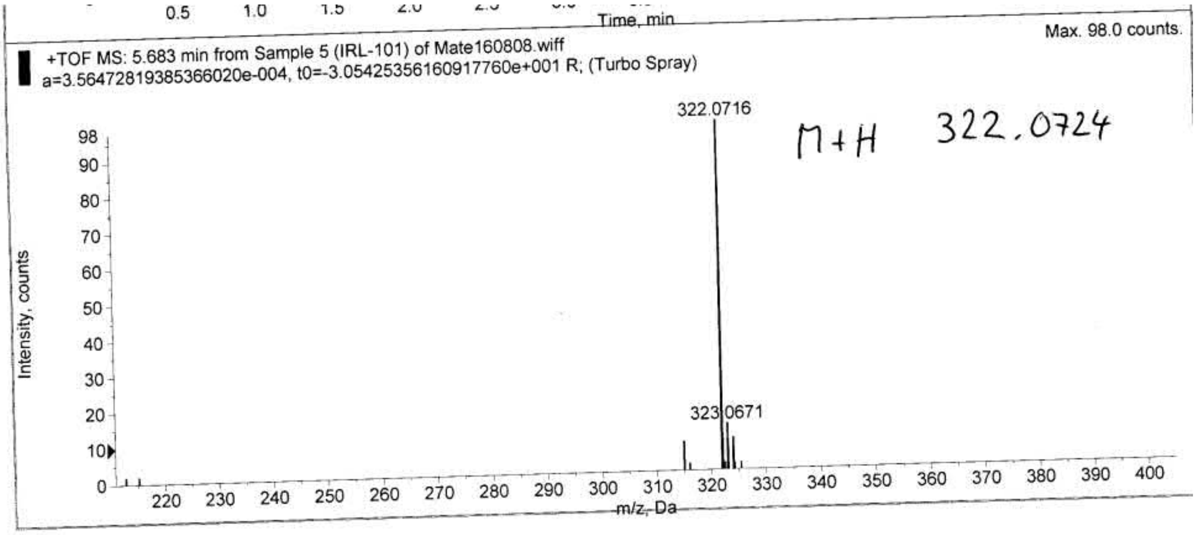

Figure S96. HRMS spectrum of (6*R*,8*S*)-(-)-5,6,7,8-tetrahydro-7,7-dimethyl-6,8-methano-isoquinolin-3-yl trifluoromethanesulfonate (**16**).

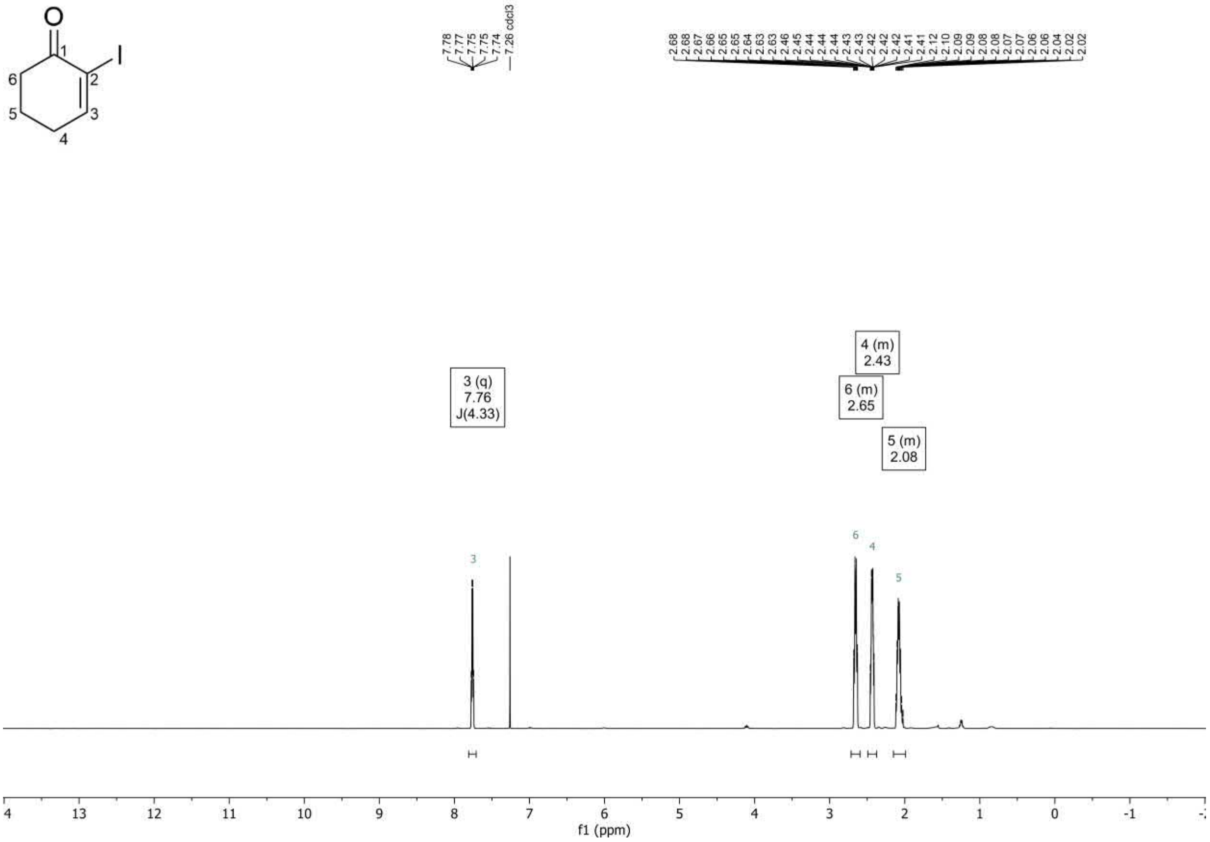

Figure S97. ^{1}H NMR spectrum of 2-Iodocyclohex-2-en-1-one (**17**) in $CDCl_3$ at 25°C.

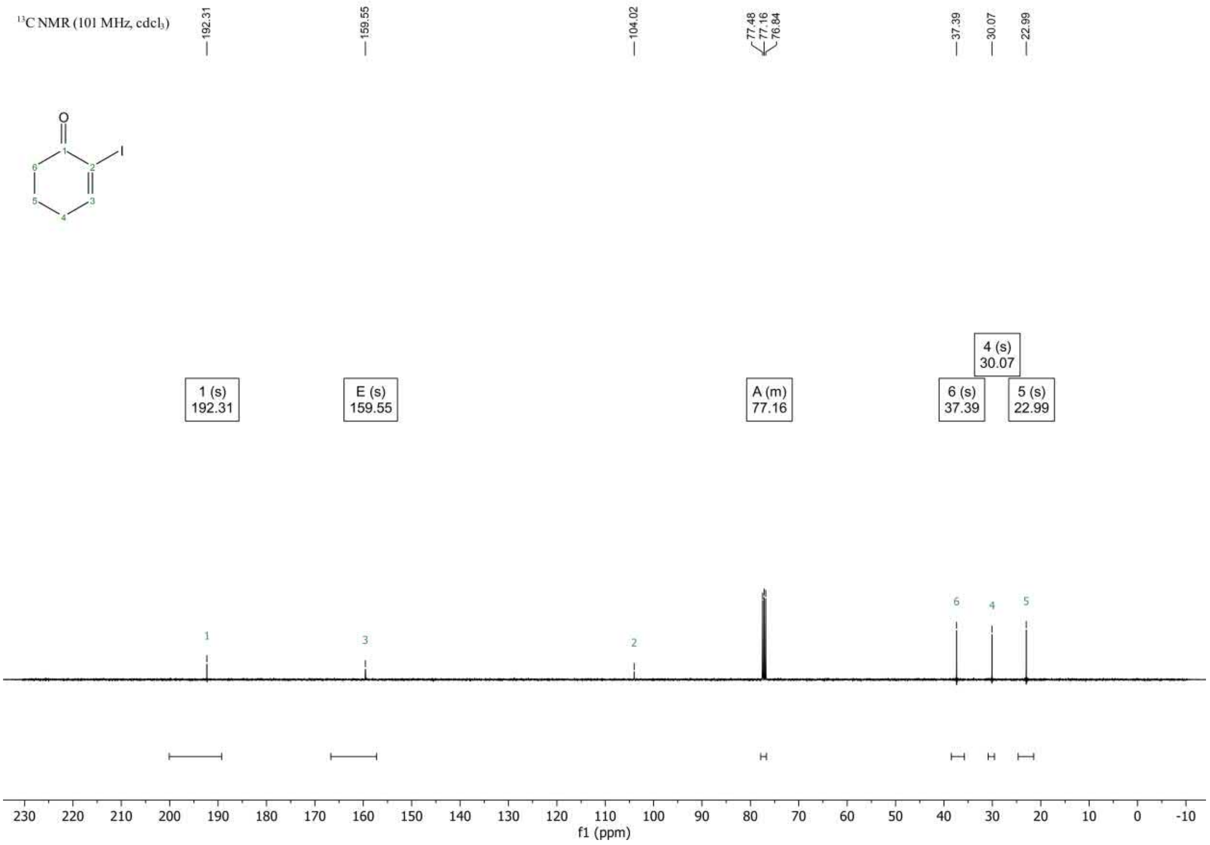

Figure S98. ^{13}C NMR spectrum of 2-Iodocyclohex-2-en-1-one (**17**) in $CDCl_3$ at 25°C.

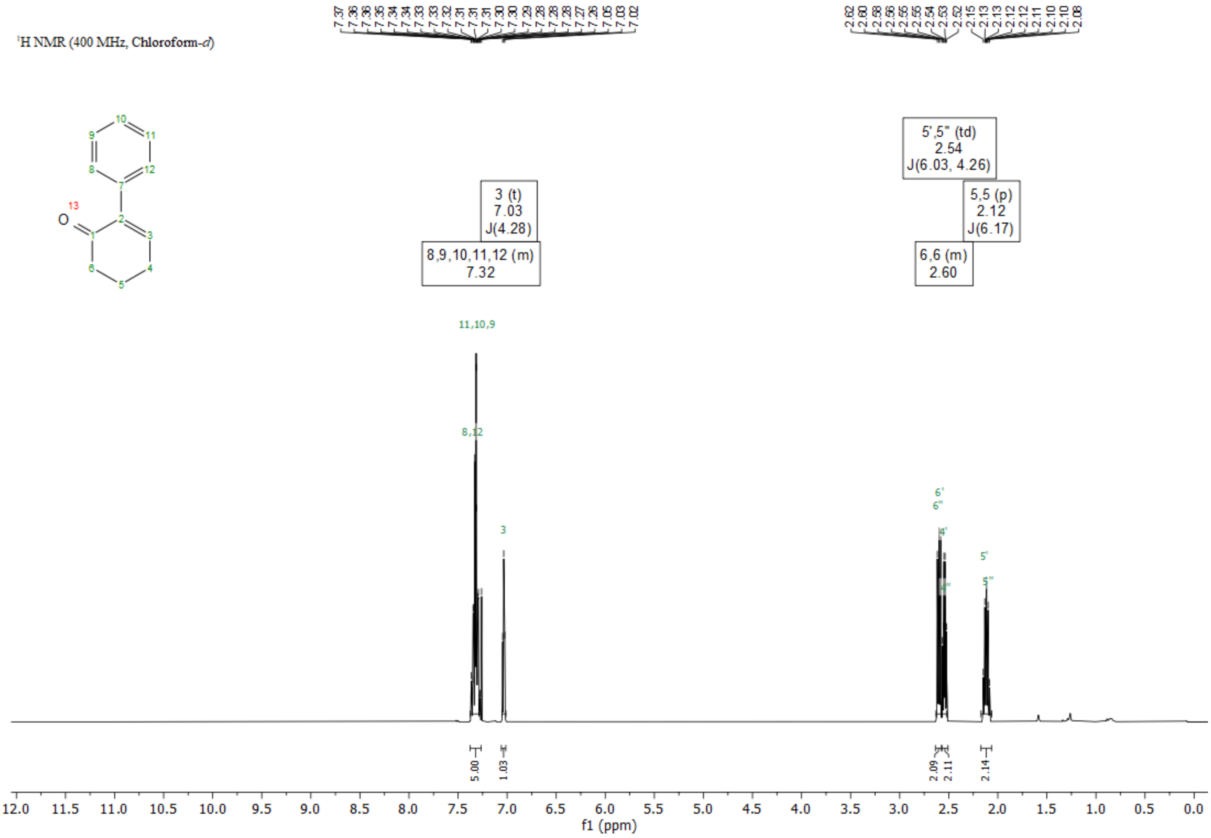

Figure S99. ^{1}H NMR spectrum of 2-iodocyclohex-2-en-1-one (**18**) in $CDCl_3$ at 25°C.

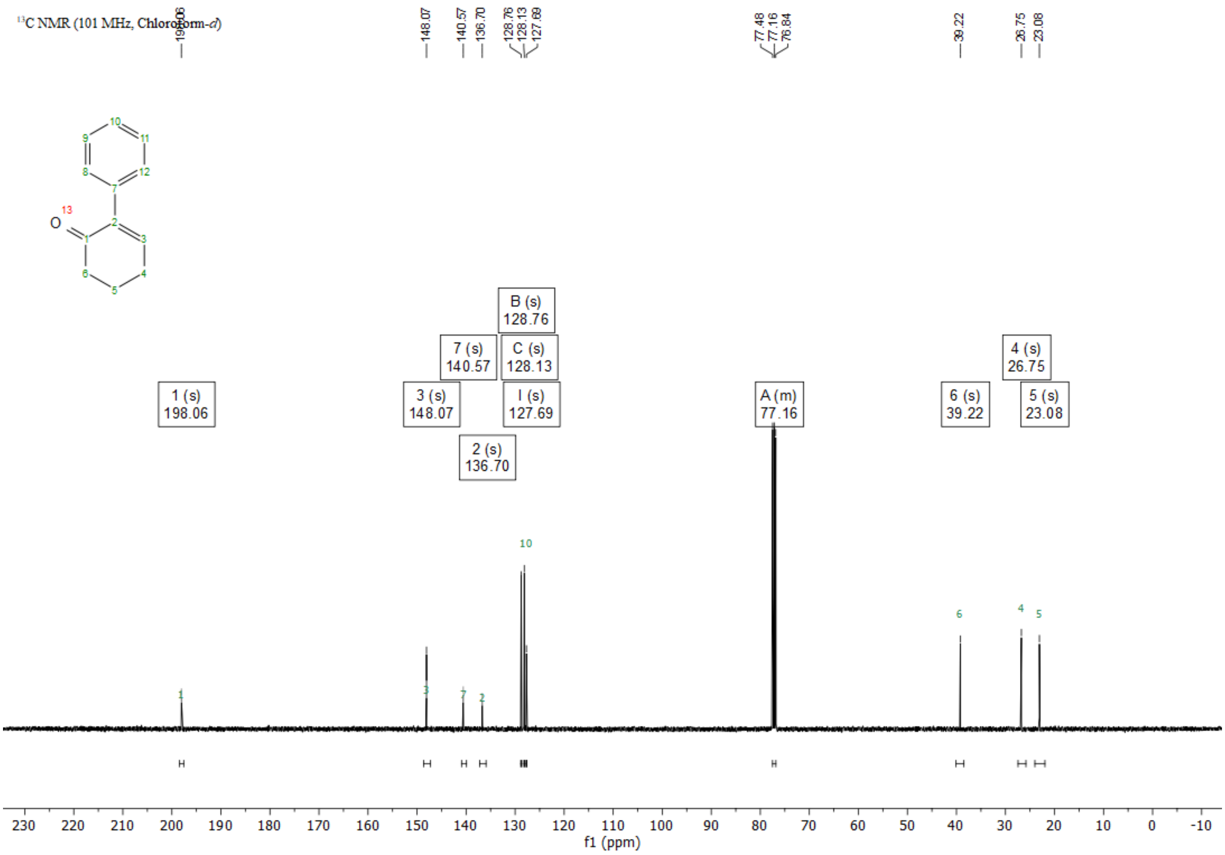

Figure S100. ^{13}C NMR spectrum of 2-iodocyclohex-2-en-1-one (**18**) in $CDCl_3$ at 25°C.

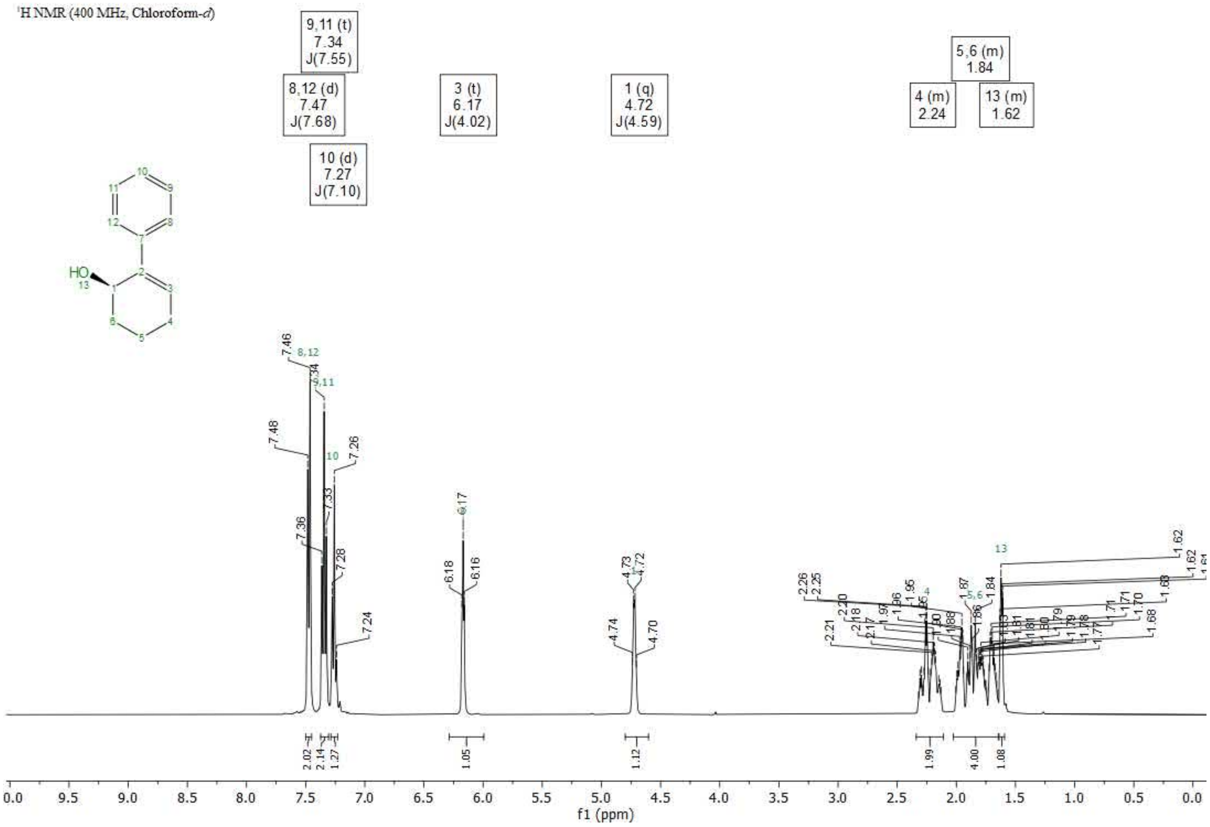

Figure S101. ^{1}H NMR spectrum of 2-phenylcyclohex-2-ene-1-ol (**19**) in $CDCl_3$ at 25°C, and 400 MHz.

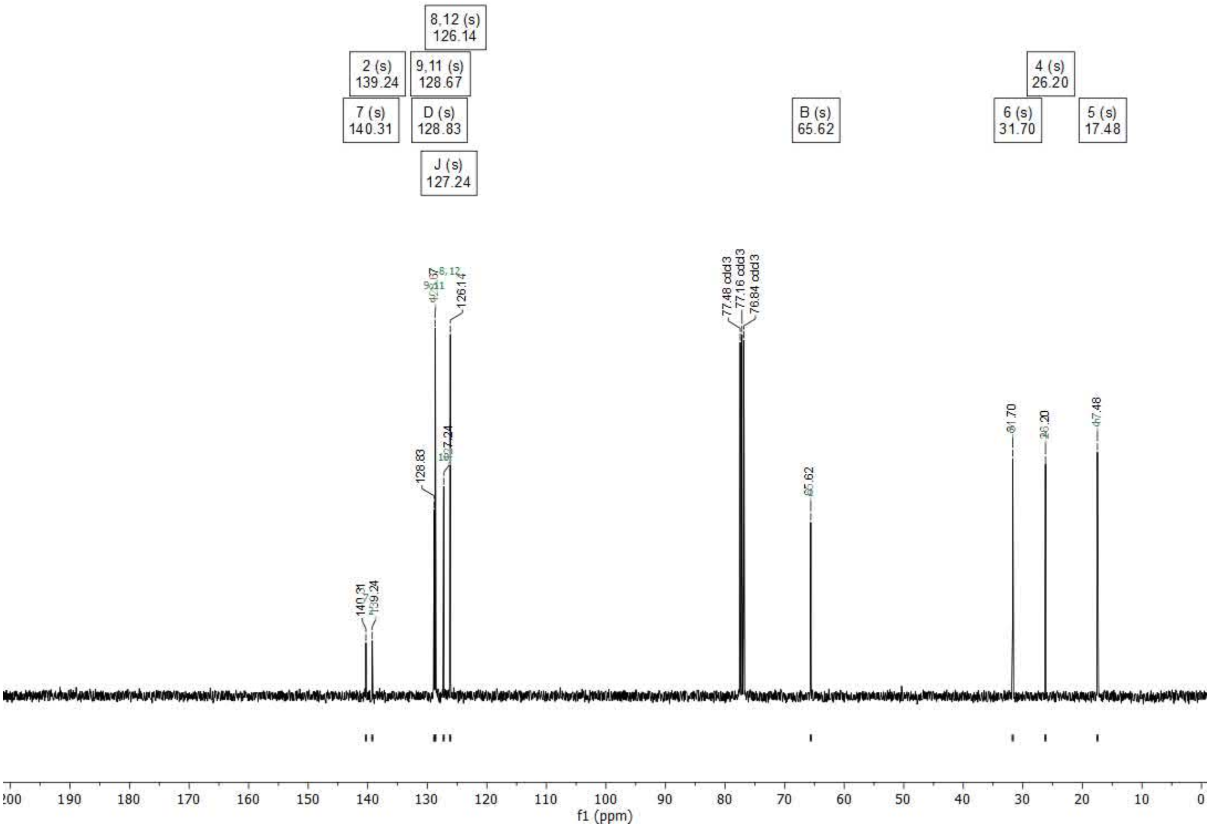

Figure S102. ^{13}C NMR spectrum of 2-phenylcyclohex-2-ene-1-ol (**19**) in $CDCl_3$ at 25°C, and 101 MHz.

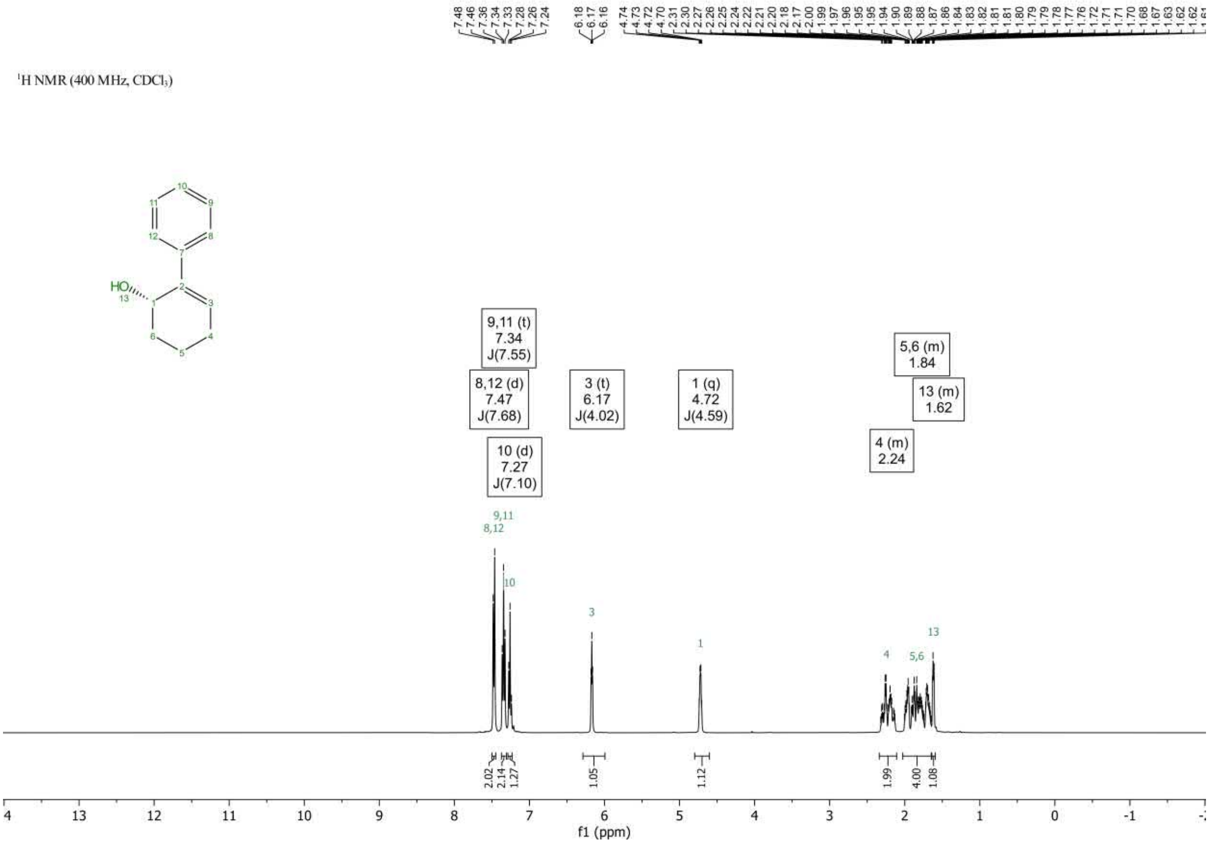

Figure S103. ^{1}H NMR spectrum of (S)-2-phenylcyclohex-2-ene-1-ol (**19**) in $CDCl_3$ at 25°C and 400 MHz.

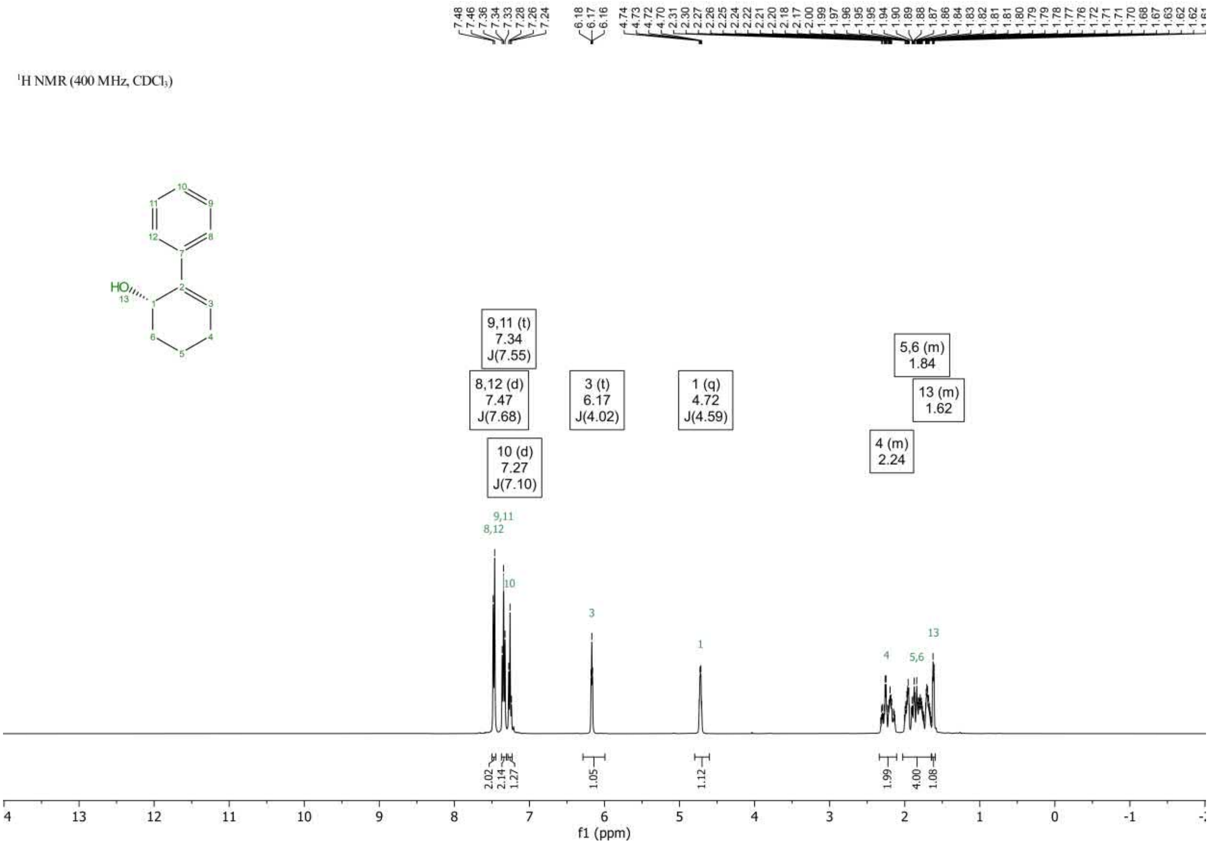

Figure S104. ^{13}C NMR spectrum of (S)-2-phenylcyclohex-2-ene-1-ol (**19**) in $CDCl_3$ at 25°C, and 101 MHz.

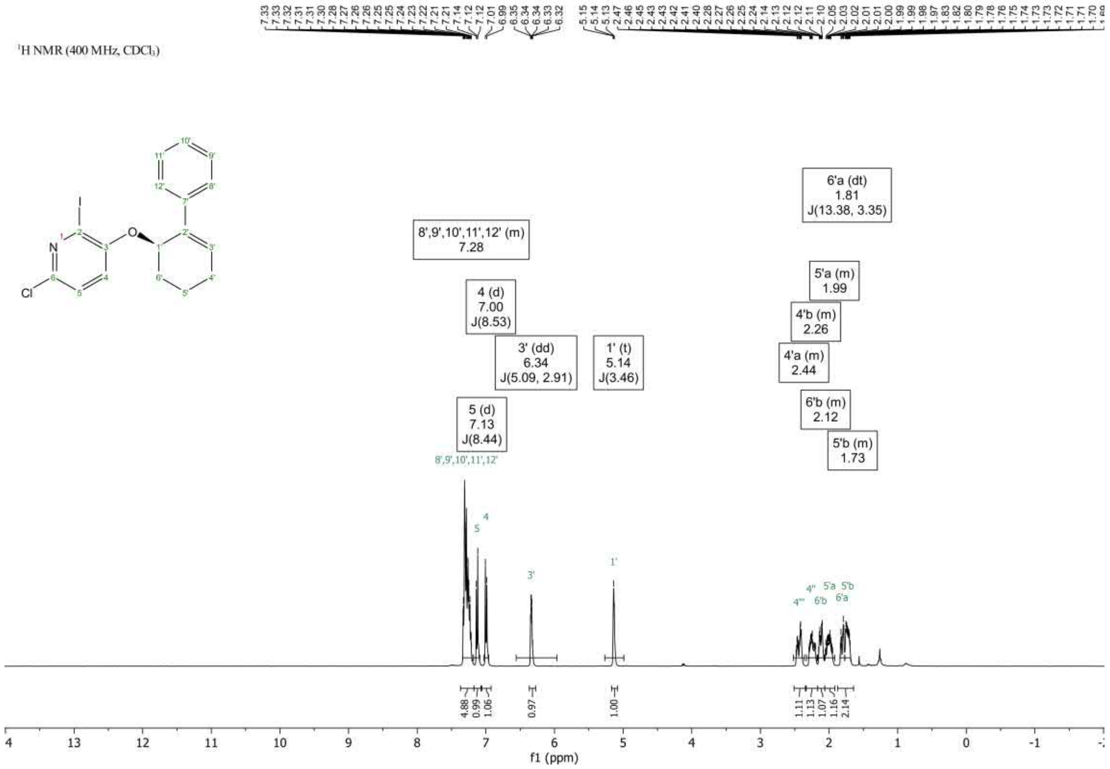

Figure S105. ^{1}H NMR spectrum of (R)-6-chloro-2-iodo-3-((2-phenylcyclohex-2-ene-1-oxy)pyridine (**20**) in $CDCl_3$ at 25°C and 400 MHz.

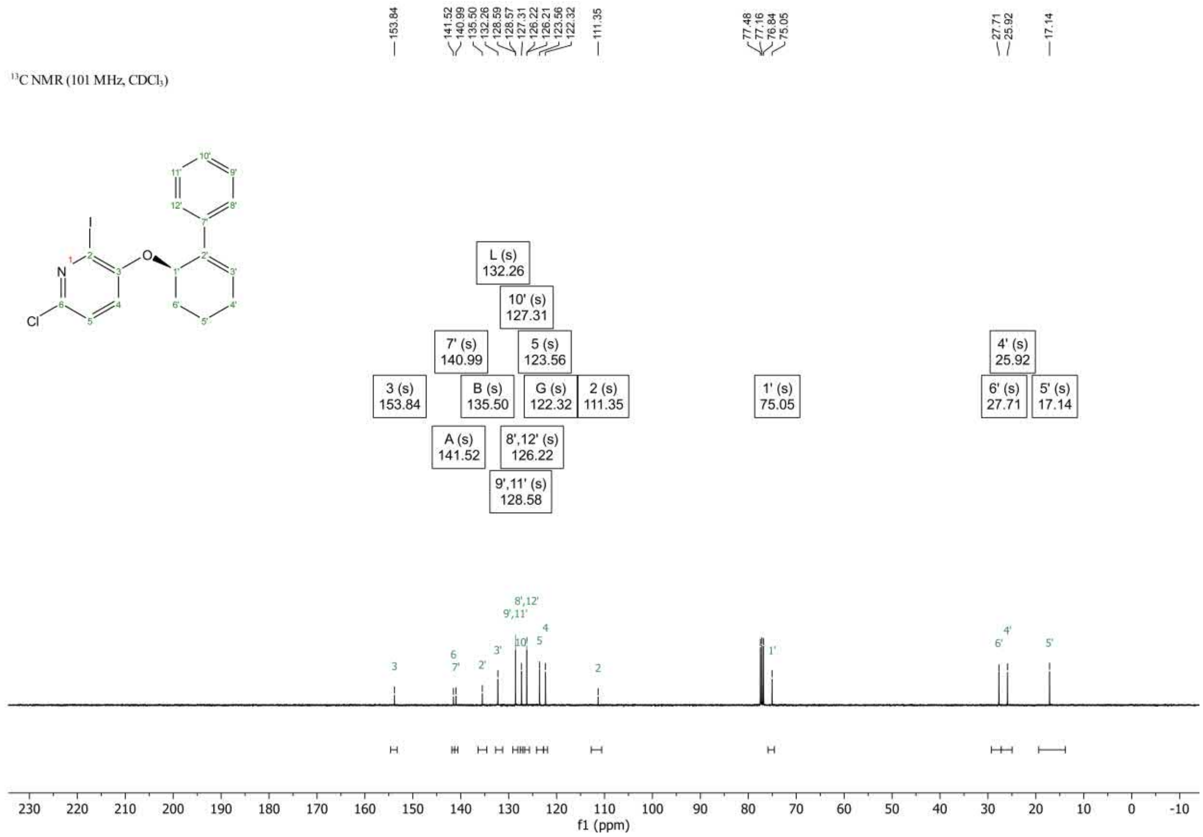

Figure S106. ^{13}C NMR spectrum of (*R*)-6-chloro-2-iodo-3-((2-phenylcyclohex-2-ene-1-oxy)pyridine (**20**) in $CDCl_3$ at 25°C, and 101 MHz.

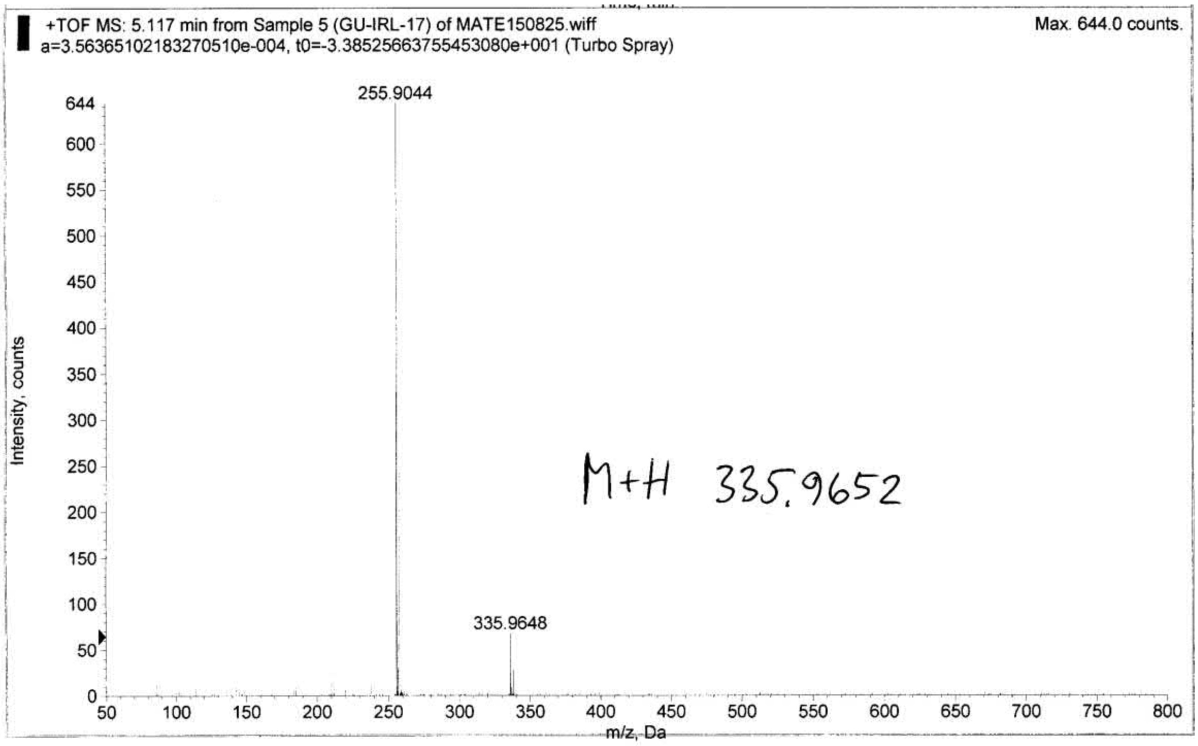

Figure S107. HRMS spectrum of (R)-6-chloro-2-iodo-3-((2-phenylcyclohex-2-ene-1-oxy)pyridine (**20**) in $CDCl_3$ at 25°C, and 101 MHz.

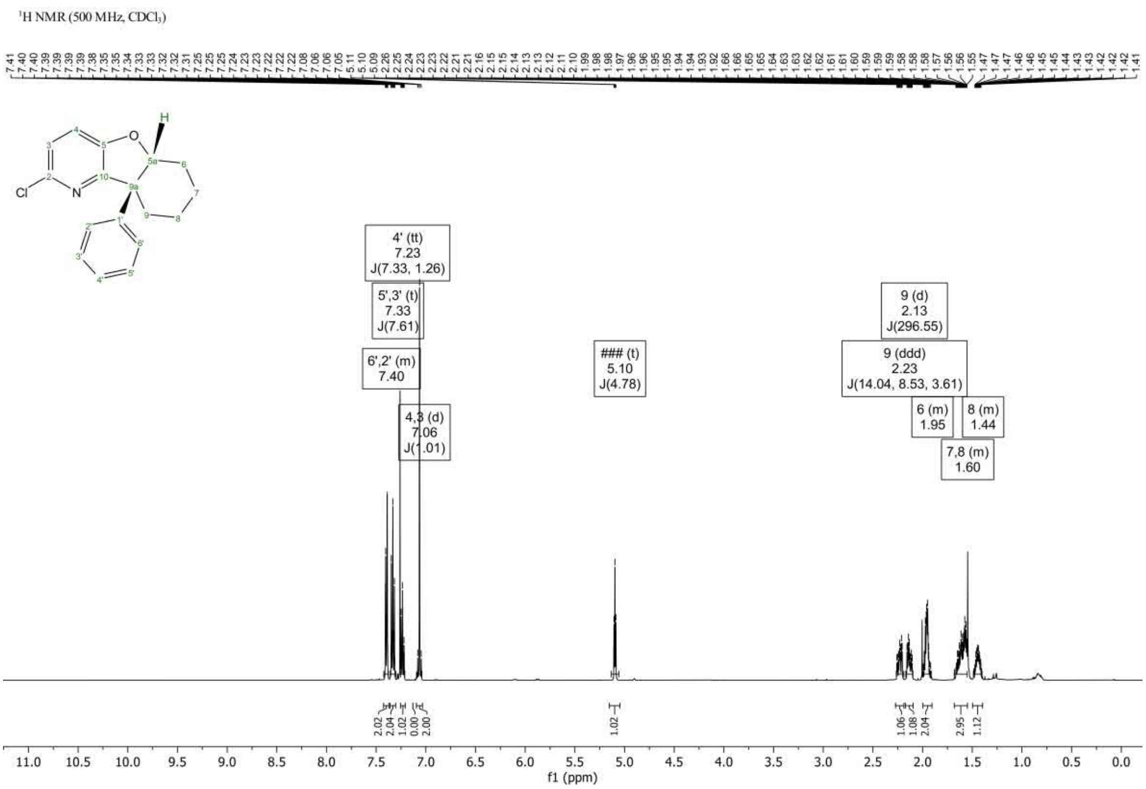

Figure S108. ^{1}H NMR spectrum of (5a*R*,9a*S*)-2-chloro-9a-phenyl-5a,6,7,8,9,9a-hexahydrobenzo-furo[3,2-b]pyridine (**22**) in $CDCl_3$ at 25℃ and 500 MHz.

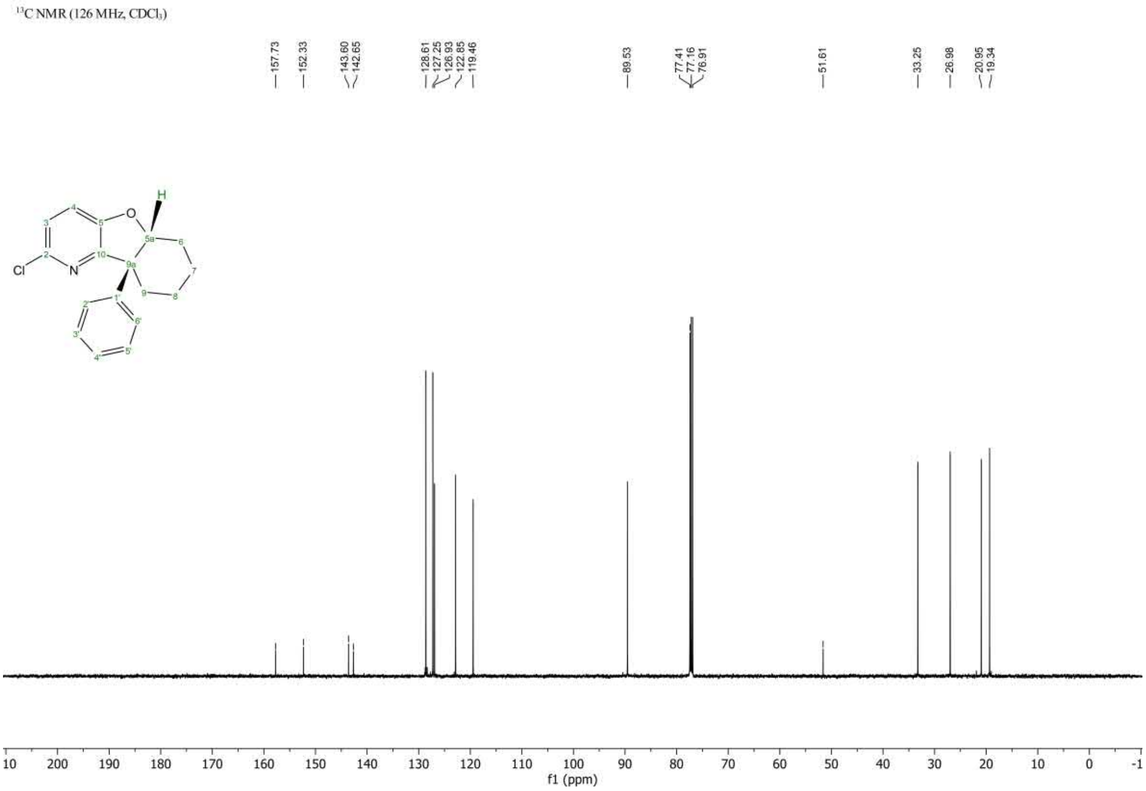

Figure S109. ^{13}C NMR spectrum of (5a*R*,9a*S*)-2-chloro-9a-phenyl-5a,6,7,8,9,9a-hexahydrobenzo-furo[3,2-b]pyridine (**22**) in $CDCl_3$ at 25℃, and 126 MHz.

(5a*R*,9a*S*)-2-Chloro-9a-phenyl-5a,6,7,8,9,9a-hexahydrobenzofuro[3,2-b]pyridine
LuxAmylose H_2O 050%, CH_3CN 050% 1 ml/min

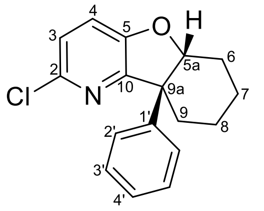

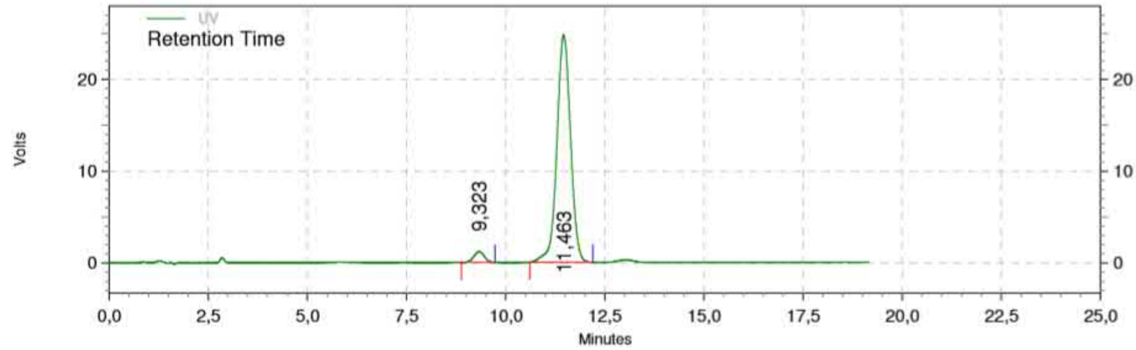

UV Results

Retention Time	Area	Area %	Height	Height %
9,323	87438	3,56	4650	4,48
11,463	2368042	96,44	99144	95,52

Figure S110. HPLC chromatohram of (5a*R*,9a*S*)-2-chloro-9a-phenyl-5a,6,7,8,9,9a-hexahydrobenzo-furo[3,2-b]pyridine (**22**).

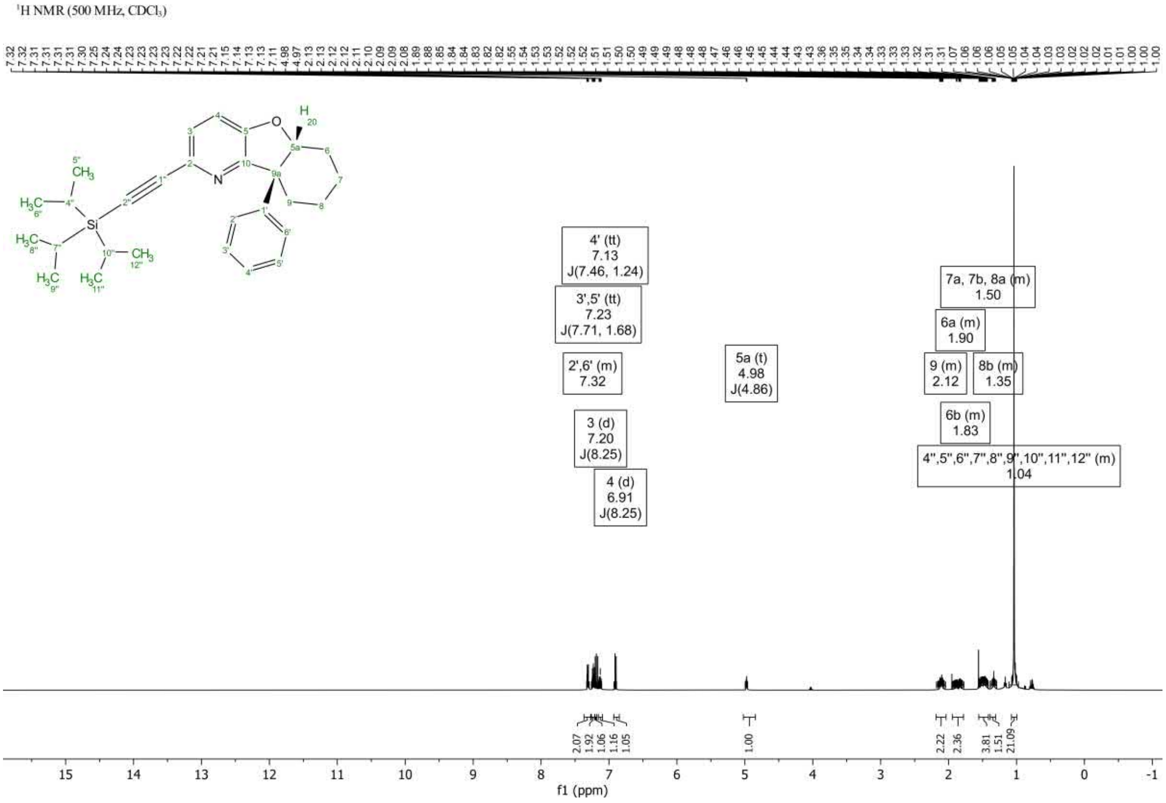

Figure S111. ^{1}H NMR spectrum of (5a*R*,9a*S*)-2-(2-tri(isopropyl)silylethyn-1-yl)-9a-phenyl-5a,6,7,8,9,9a-hexahydrobenzofuro[3,2-b]pyridine (5a*R*,9a*S*-**23**) in $CDCl_3$ at 25°C, 500 MHz.

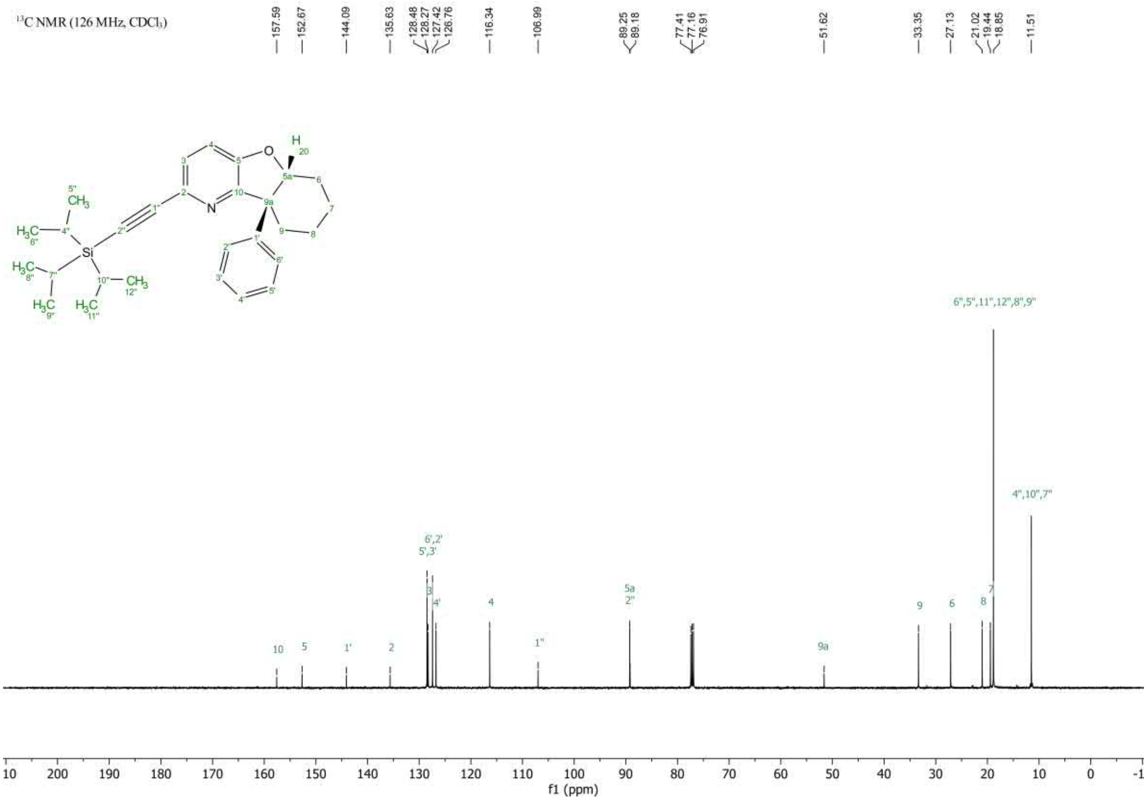

Figure S112. ^{13}C NMR spectrum of (5a*R*,9a*S*)-2-(2-tri(isopropyl)silylethyn-1-yl)-9a-phenyl-5a,6,7,8,9,9a-hexahydrobenzofuro[3,2-b]pyridine (5a*R*,9a*S*-**23**) in $CDCl_3$ at 25°C, and 126 MHz.

HPLC:
(5a*R*,9a*S*)-2-(2-tri(isopropyl)silylethyn-1-yl)-9a-phenyl-5a,6,7,8,9,9a-hexahydrobenzofuro[3,2-b]pyridine
LuxAmylose H_2O 030%, CH_3CN 070% 1 ml/min

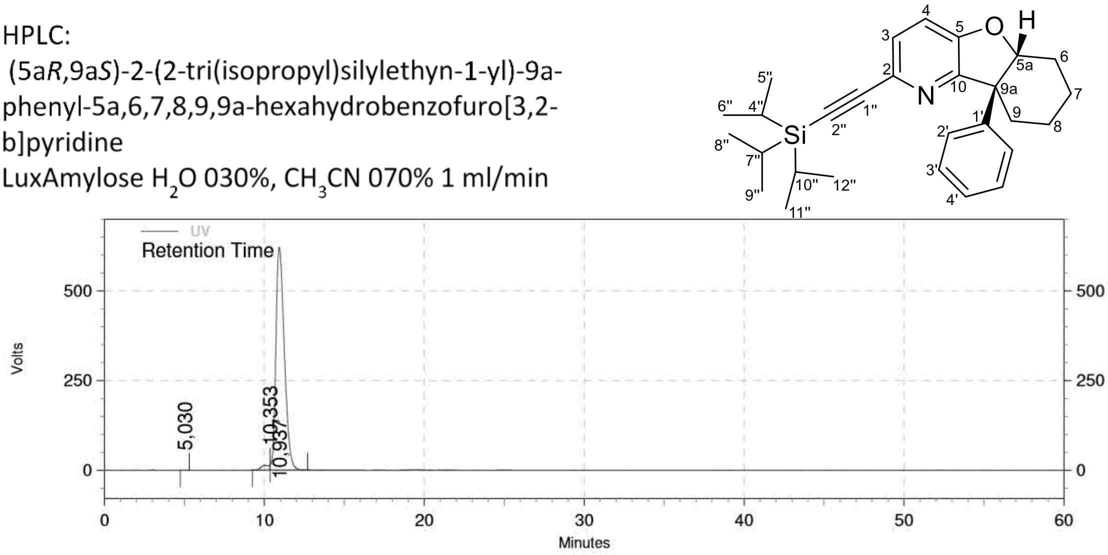

UV Results

Retention Time	Area	Area %	Height	Height %
5,030	5002	0,01	427	0,02
10,353	1885028	2,02	55352	2,18
10,937	91406072	97,97	2486443	97,81

Figure S113. HPLC chromatogram of (5a*R*,9a*S*)-2-(2-tri(isopropyl)silylethyn-1-yl)-9a-phenyl-5a,6,7,8,9,9a-hexahydrobenzofuro[3,2-b]pyridine (5aR,9aS-**23**), LuxAmylose H_2O 30%, CH_3CN 70% 1ml/min.

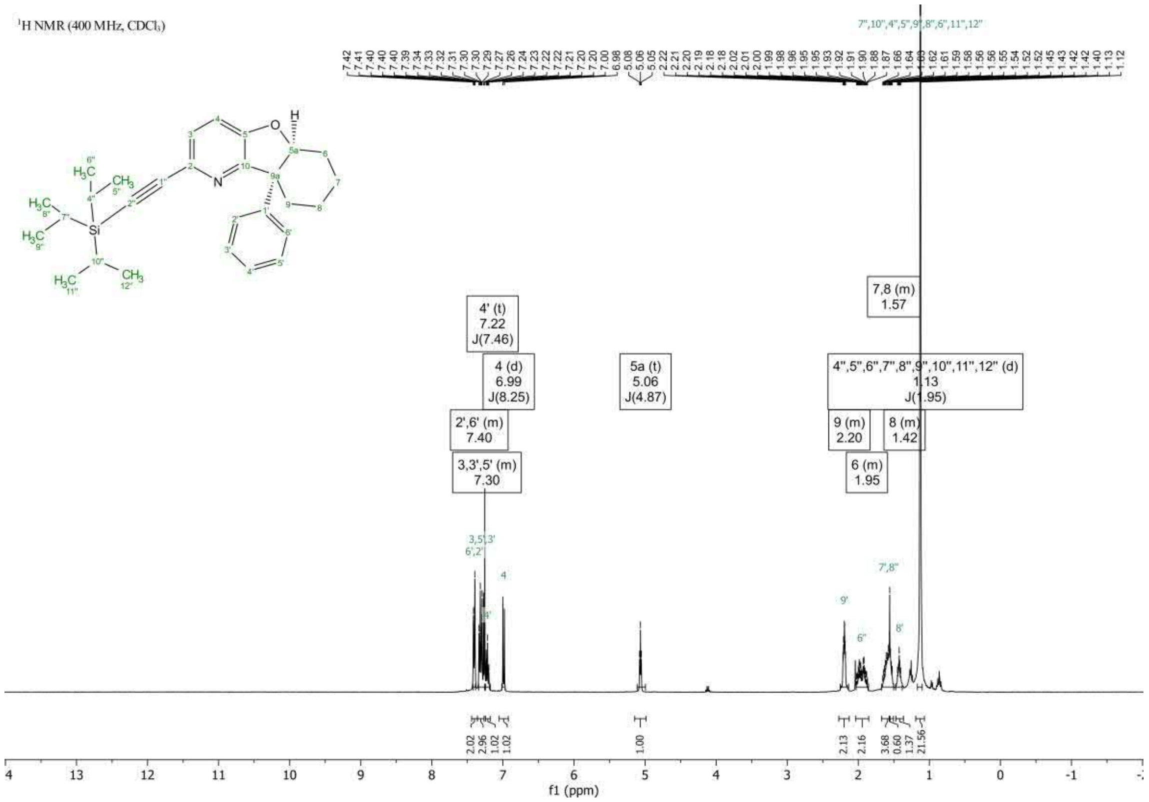

Figure S114. ^{1}H NMR spectrum of (5a*S*,9a*R*)-2-(2-tri(isopropyl)silylethyn-1-yl)-9a-phenyl-5a,6,7,8,9,9a-hexahydrobenzofuro[3,2-b]pyridine (**23**) in $CDCl_3$ at 25°C, 500 MHz.

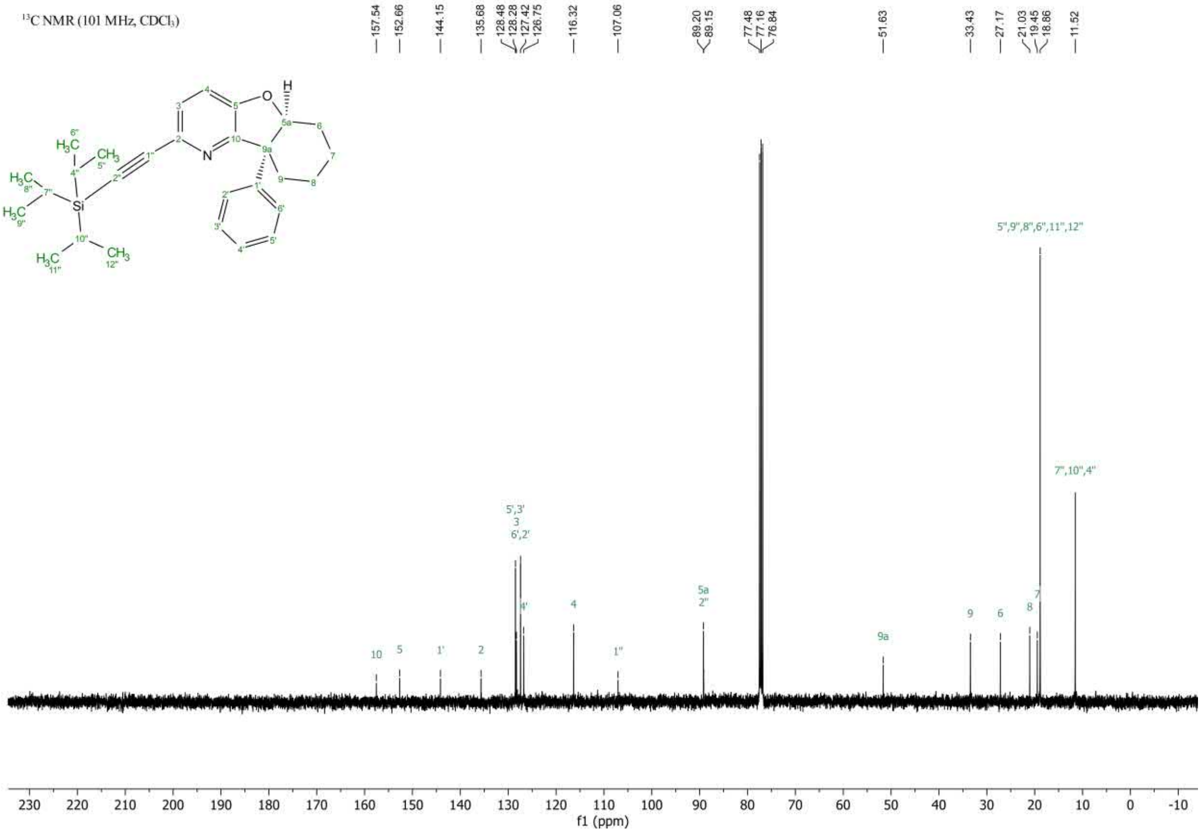

Figure S115. ^{13}C NMR spectrum of (5a*S*,9a*R*)-2-(2-tri(isopropyl)silylethyn-1-yl)-9a-phenyl-5a,6,7,8,9,9a-hexahydrobenzofuro[3,2-b]pyridine (5a*S*,9a*R*-**23**) in $CDCl_3$ at 25°C, and 126 MHz.

HPLC:
(5a*S*,9a*R*)-2-(2-tri(isopropyl)silylethyn-1-yl)-9a-phenyl-5a,6,7,8,9,9a-hexahydrobenzofuro[3,2-b]pyridine
LuxAmylose H_2O 030%, CH_3CN 070% 1 ml/min

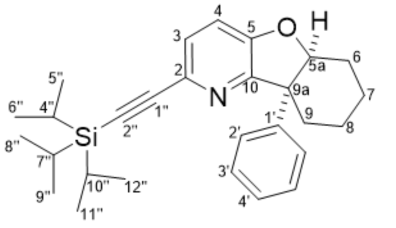

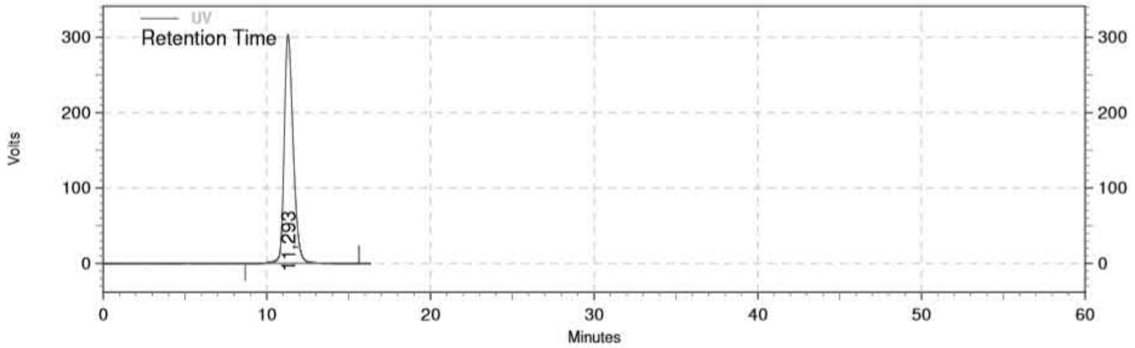

Figure S116. HPLC chromatogram of (5a*S*,9a*R*)-2-(2-tri(isopropyl)silylethyn-1-yl)-9a-phenyl-5a,6,7,8,9,9a-hexahydrobenzofuro[3,2-*b*]pyridine (5a*S*,9a*R*-**23**), LuxAmylose H_2O 30%, CH_3CN 70% 1ml/min.

HPLC:
(5a*R*,9a*S*)-2-(2-tri(isopropyl)silylethyn-1-yl)-9a-phenyl-5a,6,7,8,9,9a-hexahydrobenzofuro[3,2-b]pyridine
LuxAmylose H_2O 030%, CH_3CN 070% 1 ml/min

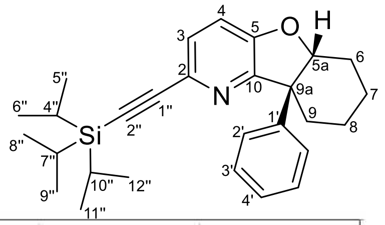

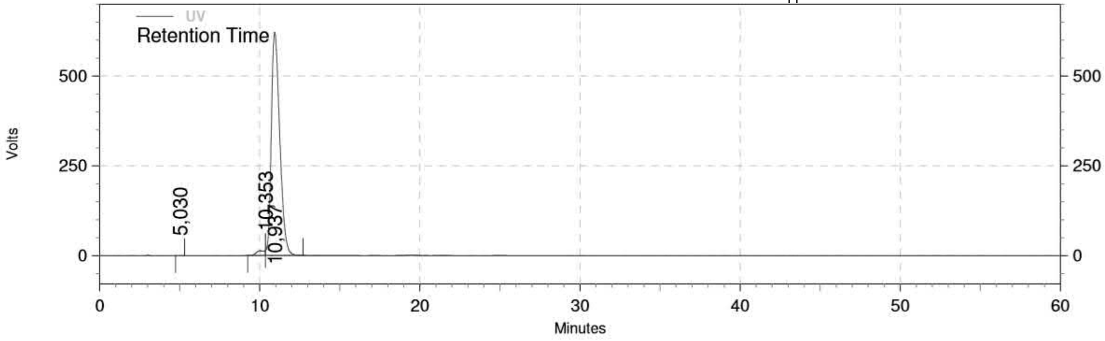

UV Results

Retention Time	Area	Area %	Height	Height %
5,030	5002	0,01	427	0,02
10,353	1885028	2,02	55352	2,18
10,937	91406072	97,97	2486443	97,81

Figure S117. HPLC chromatogram of (5aR,9a*S*)-2-(2-tri(isopropyl)silylethyn-1-yl)-9a-phenyl-5a,6,7,8,9,9a-hexahydrobenzofuro[3,2-*b*]pyridine (5a*S*,9a*R*-**23**), LuxAmylose H_2O 30%, CH_3CN 70% 1ml/min.

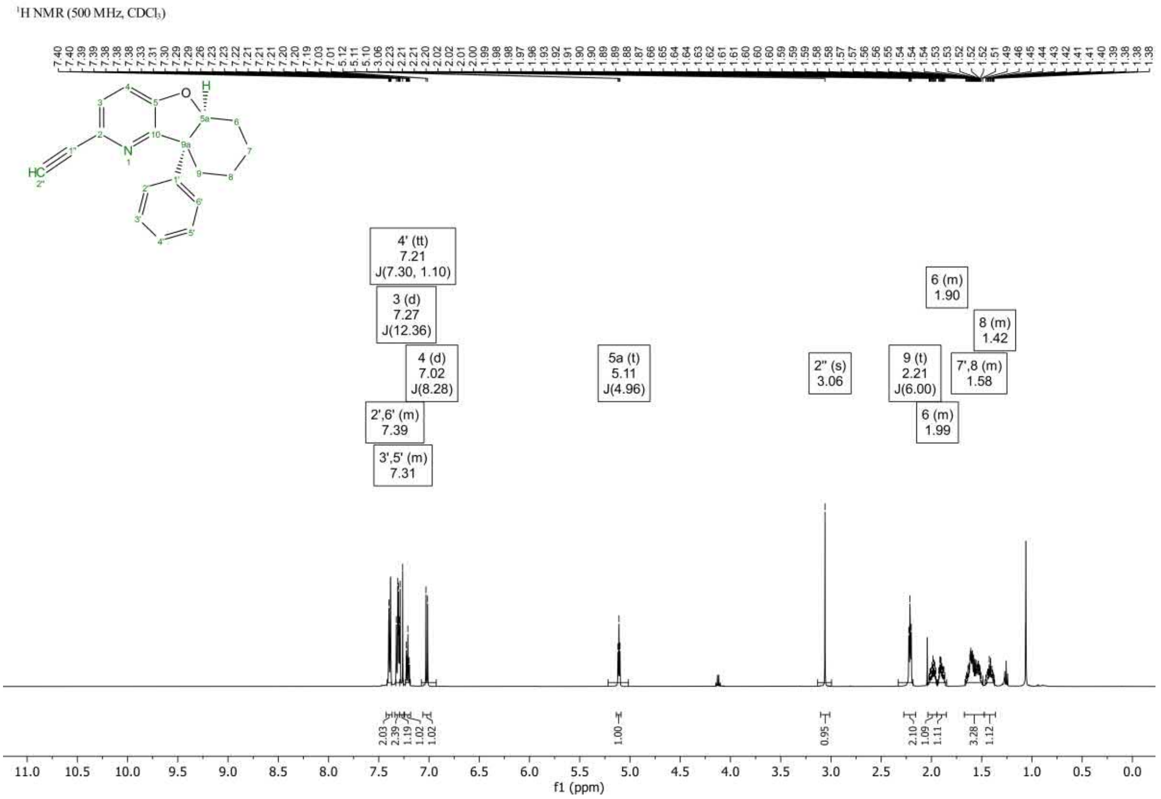

Figure S118. ^{1}H NMR spectrum of (5a*S*,9a*R*)-2-ethynyl-9a-phenyl-5a,6,7,8,9,9a-hexahydrobenzo-furo[3,2-b]pyridine ((5a*S*,9a*R*)-**24**) in $CDCl_3$ at 25°C and 500 MHz.

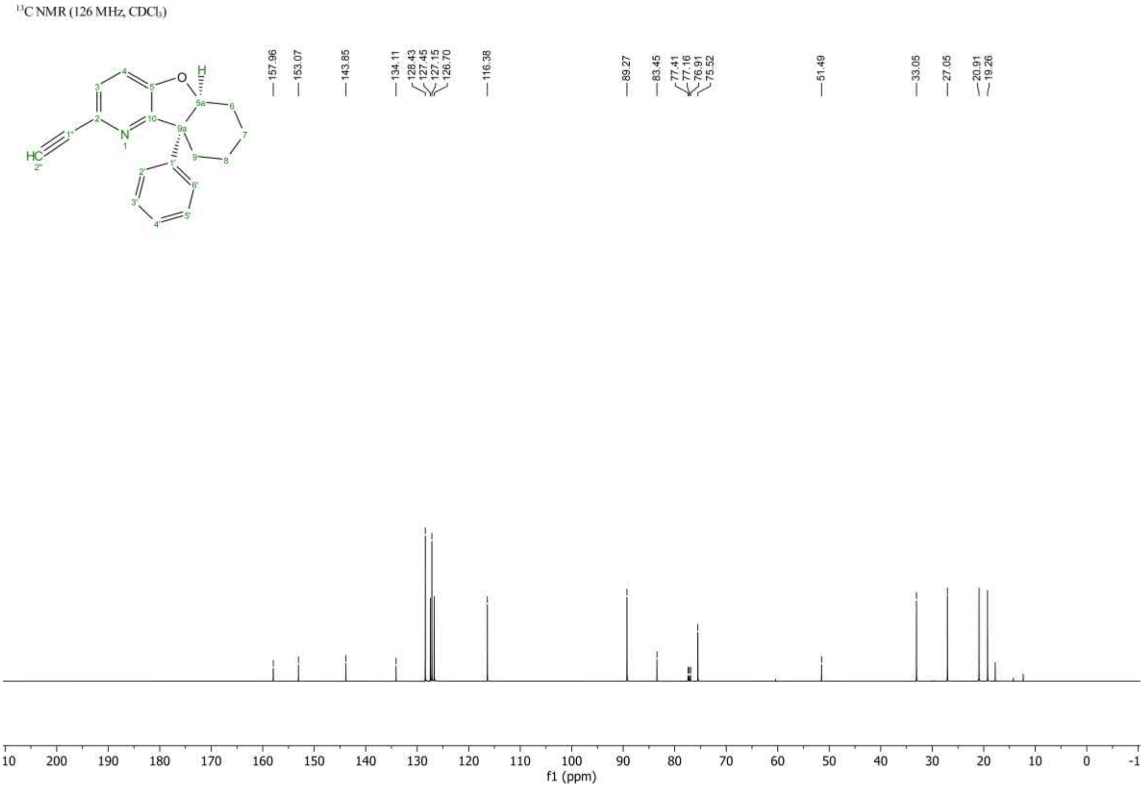

Figure S119. ^{13}C NMR spectrum of (5a*S*,9a*R*)-2-ethynyl-9a-phenyl-5a,6,7,8,9,9a-hexahydrobenzo-furo[3,2-b]pyridine ((5a*S*,9a*R*)-**24**) in $CDCl_3$ at 25°C, and 126 MHz.

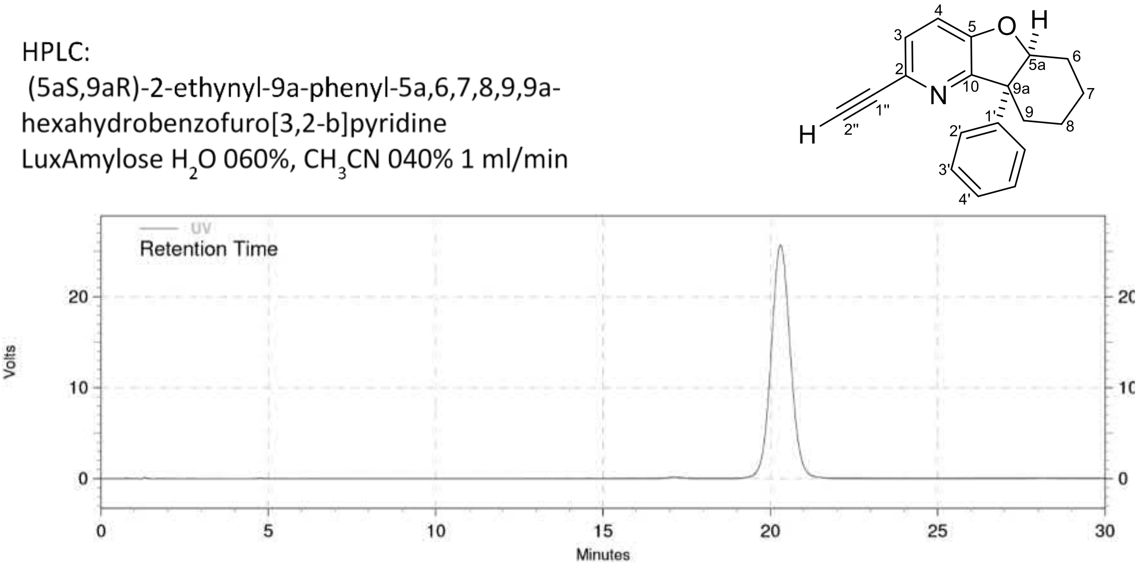

Figure S120. HPLC chromatogram of (5a*S*,9a*R*)-2-ethynyl-9a-phenyl-5a,6,7,8,9,9a-hexahydrobenzofuro[3,2-*b*]pyridine ((5a*S*,9a*R*)-**24**), LuxAmylose H_2O 60%, CH_3CN 40% 1ml/min.

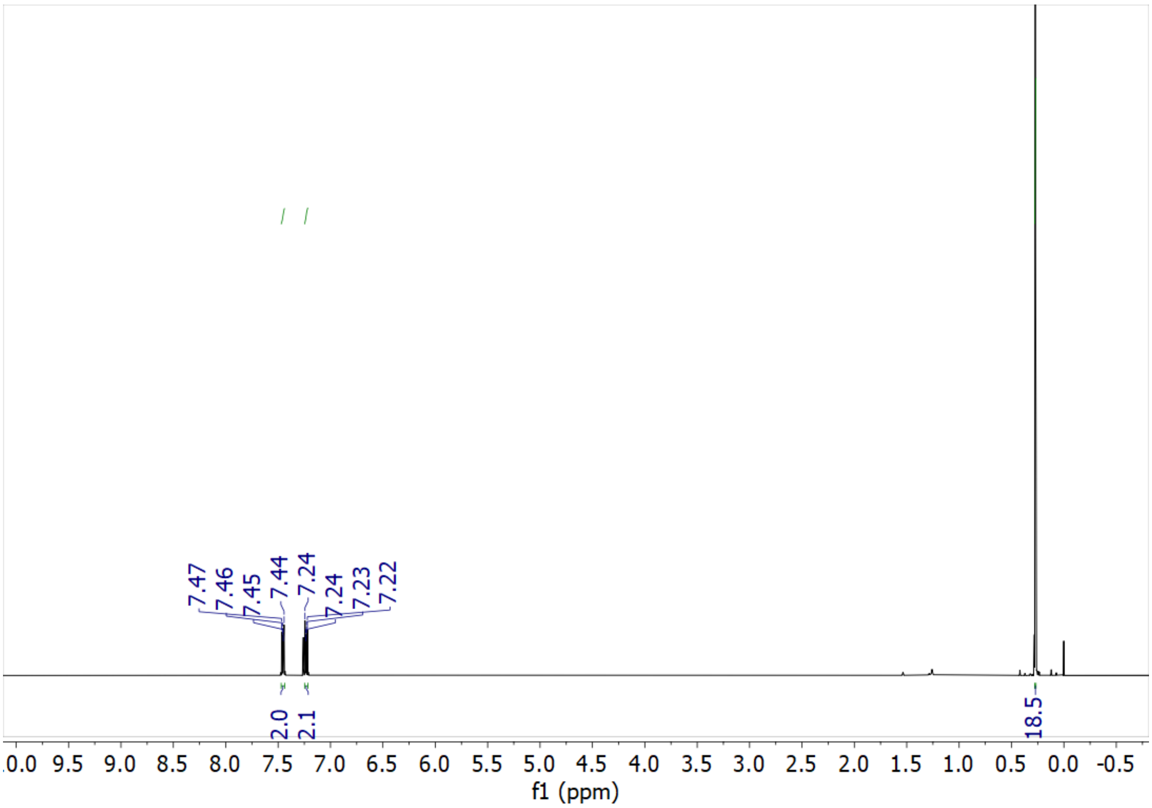

Figure S121. ^{1}H NMR spectrum of 1,2-bis((trimethylsilyl)ethynyl)benzene in $CDCl_3$ at 25°C and 399.9 MHz.

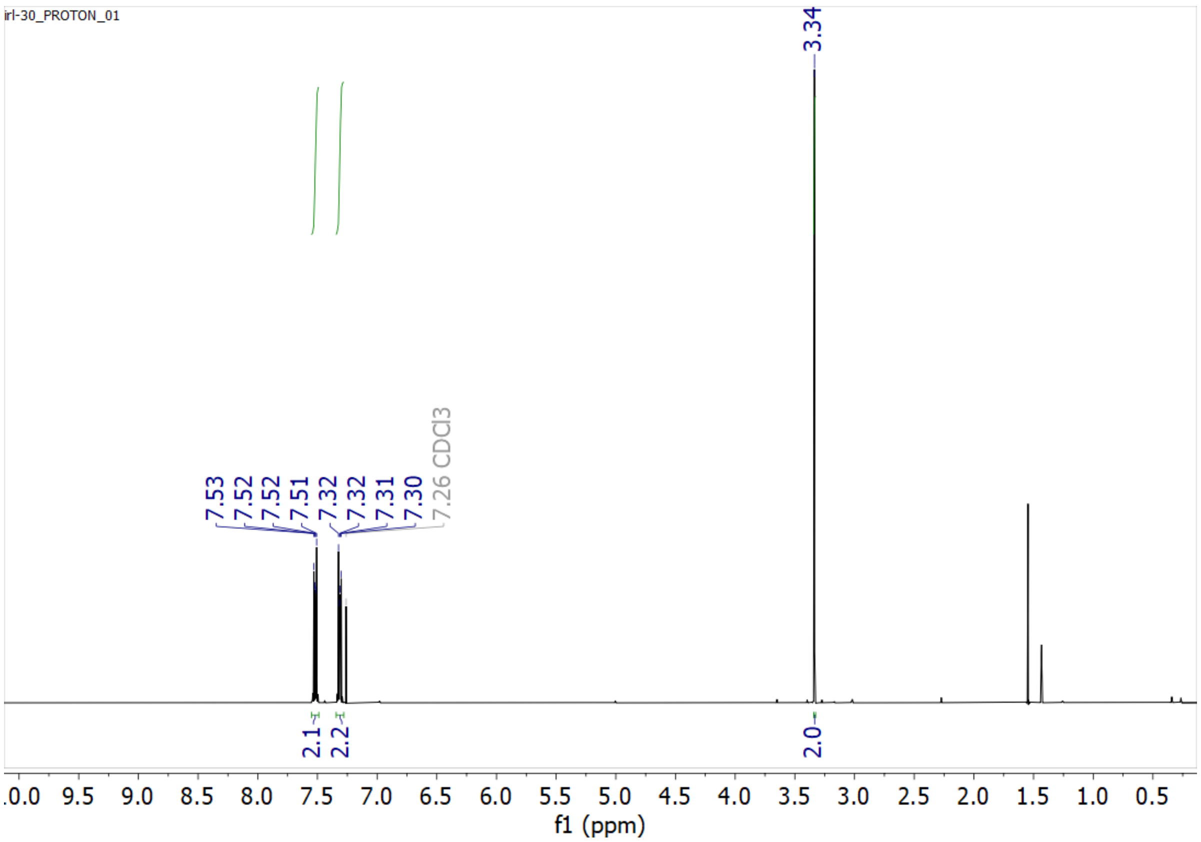

Figure S122. ^{1}H NMR spectrum of 1,2-diethynylbenzene in $CDCl_3$ at 25℃ and 399.9 MHz.

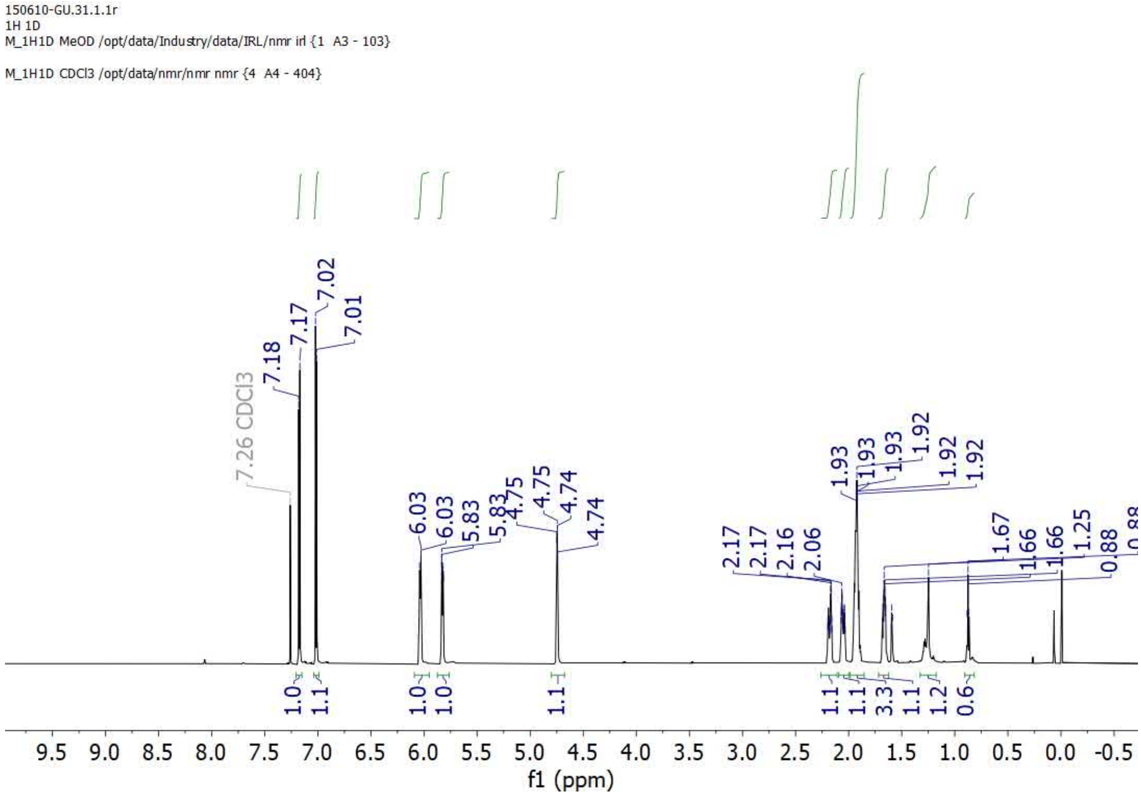

Figure S123. ^{1}H NMR spectrum of 6-chloro-3-(cyclohex-2-en-1-yloxy)-2-iodopyridine (**25**) in $CDCl_3$ at 25℃ and 800 MHz.

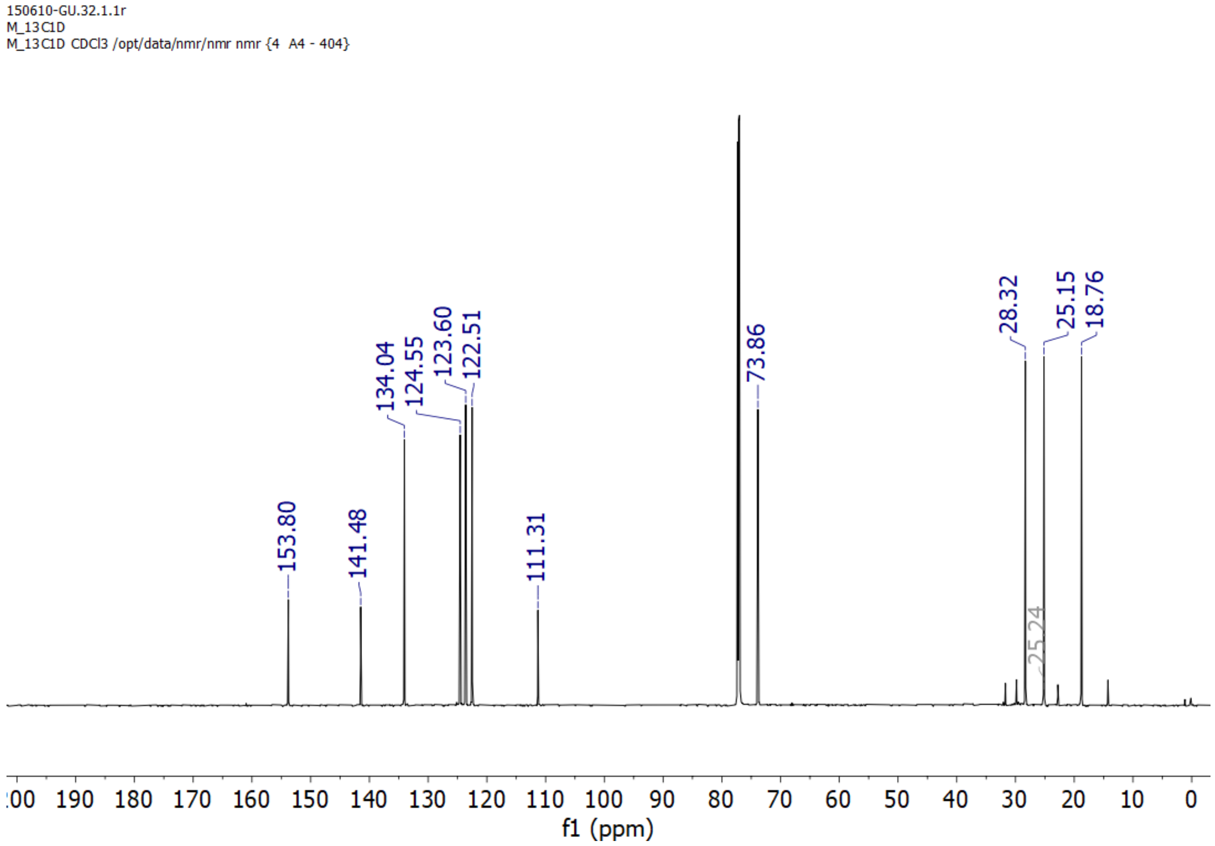

Figure S124. ^{13}C NMR spectrum of 6-chloro-3-(cyclohex-2-en-1-yloxy)-2-iodopyridine (**25**) in $CDCl_3$ at 25℃, and 200 MHz.

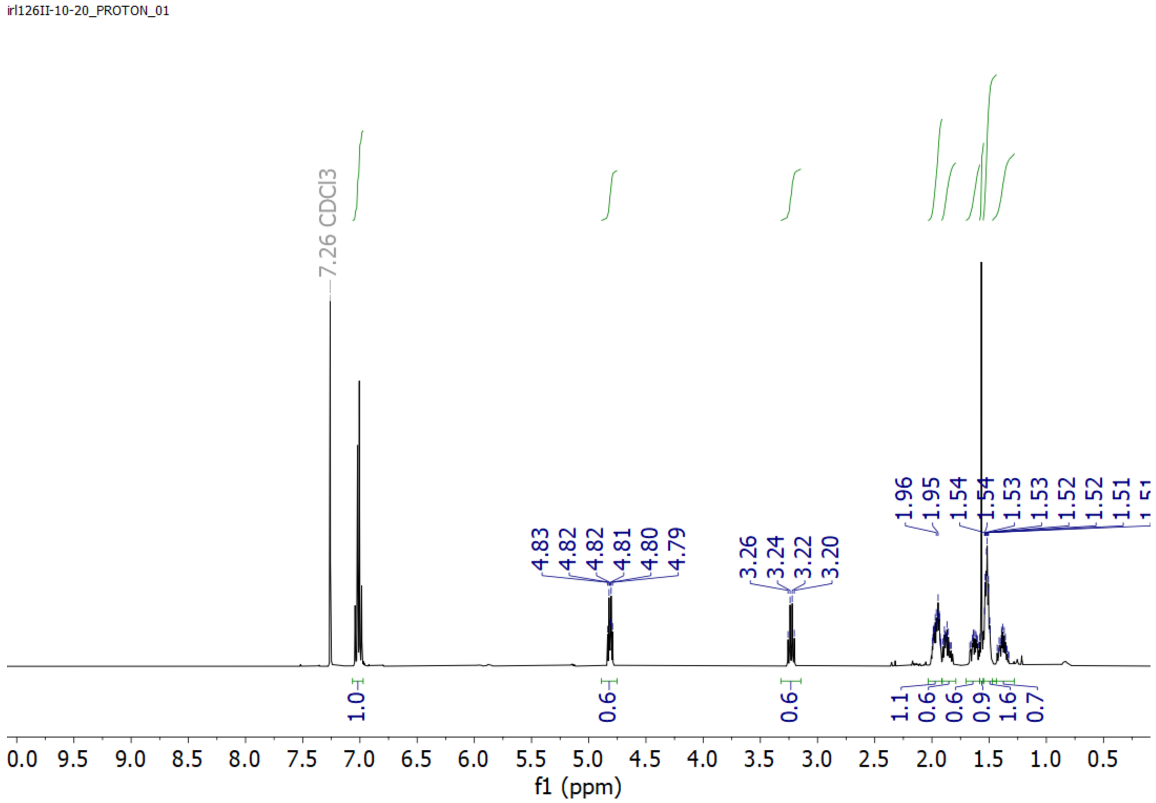

Figure S125. ^{1}H NMR spectrum of 2-chloro-5a,6,7,8,9,9a-hexahydrobenzofuro[3,2-*b*]pyridine (**26**) in $CDCl_3$ at 25℃ and 800 MHz.

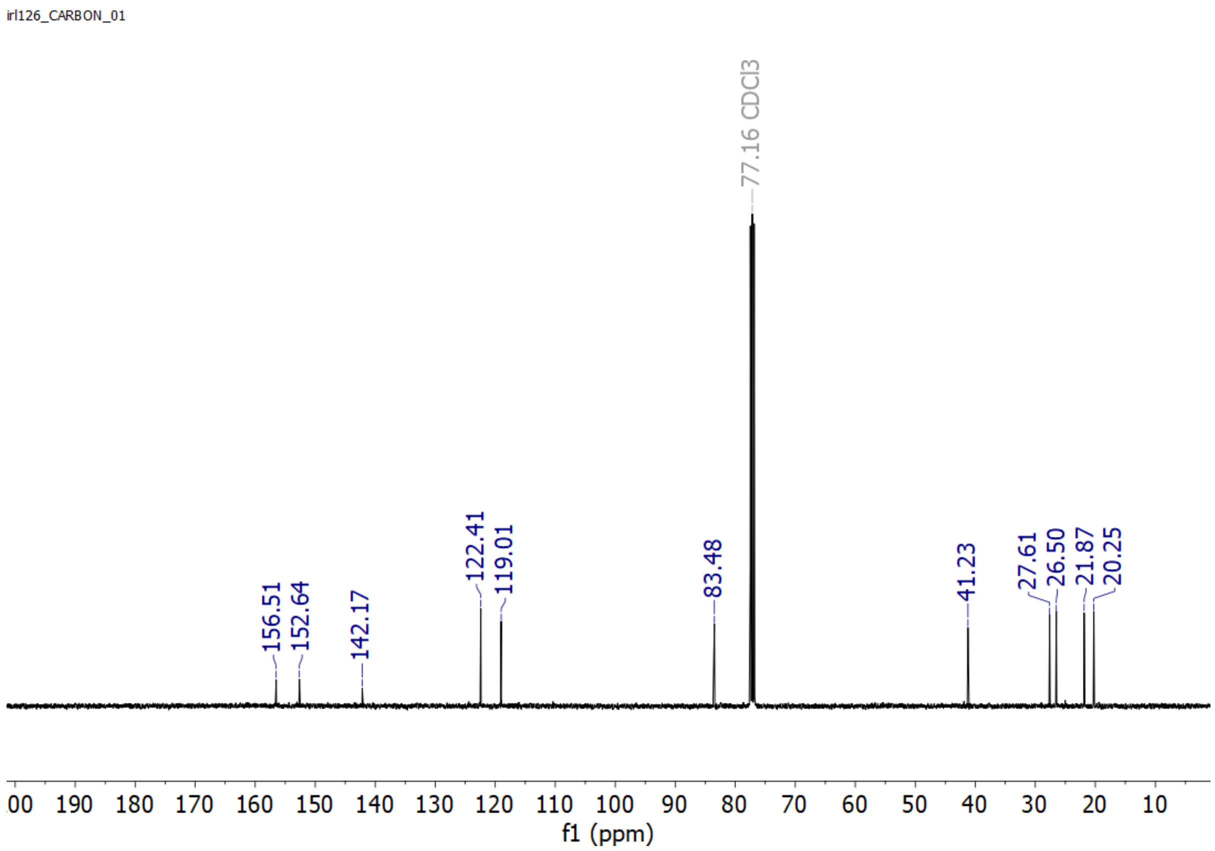

Figure S126. ^{13}C NMR spectrum of 2-chloro-5a,6,7,8,9,9a-hexahydrobenzofuro[3,2-*b*]pyridine (**26**) in $CDCl_3$ at 25°C, and 200 MHz.

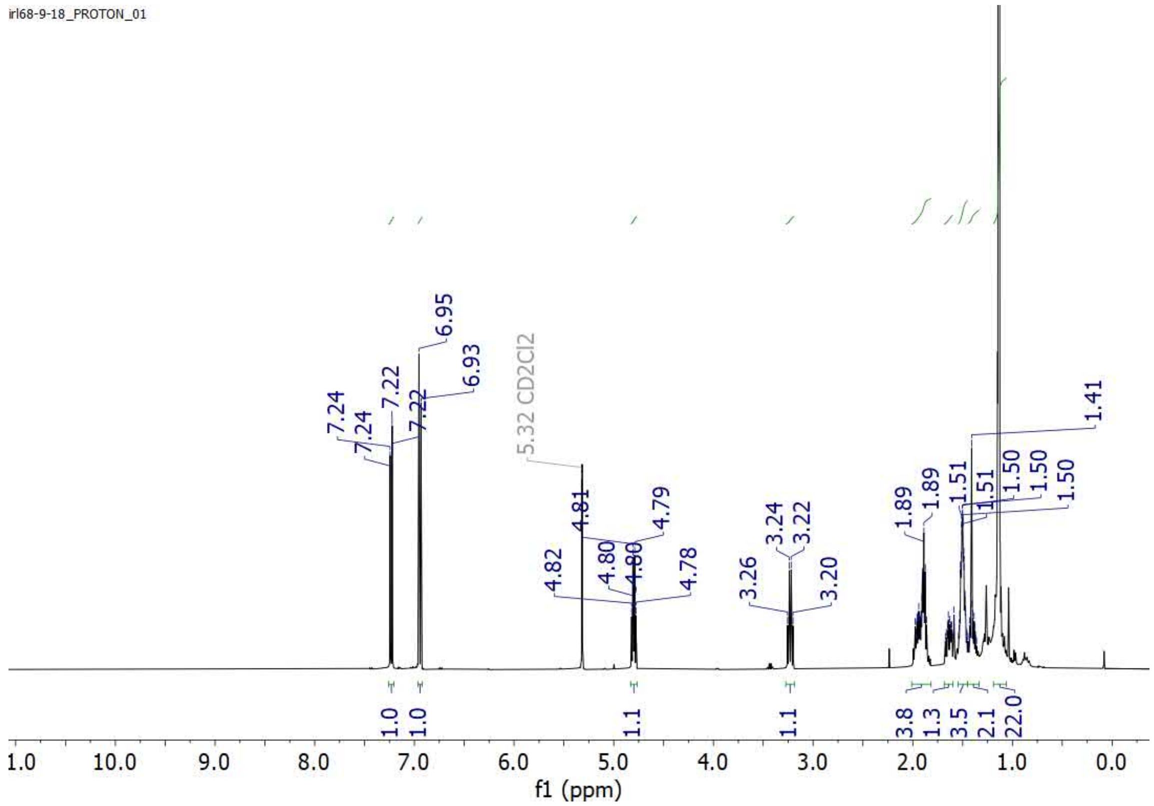

Figure S127. ^{1}H NMR spectrum of 2-((triisopropylsilyl)ethynyl)-5a,6,7,8,9,9a-hexahydrobenzo-furo[3,2-b]pyridine (**27**) in CD_2Cl_2 at 25°C and 399.9 MHz.

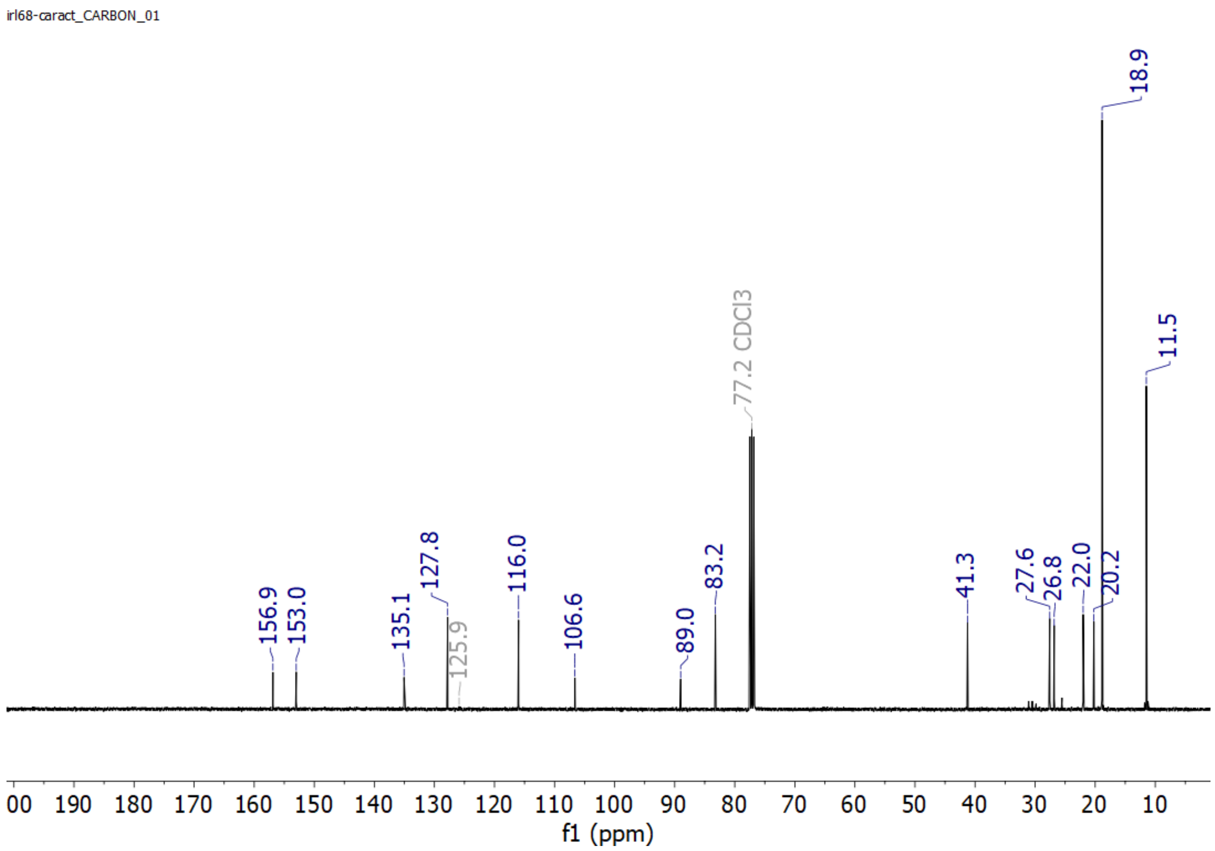

Figure S128. ^{13}C NMR spectrum of 2-((triisopropylsilyl)ethynyl)-5a,6,7,8,9,9a-hexahydrobenzofuro[3,2-b]pyridine (**27**) in $CDCl_3$ at 25°C, and 100.6 MHz.

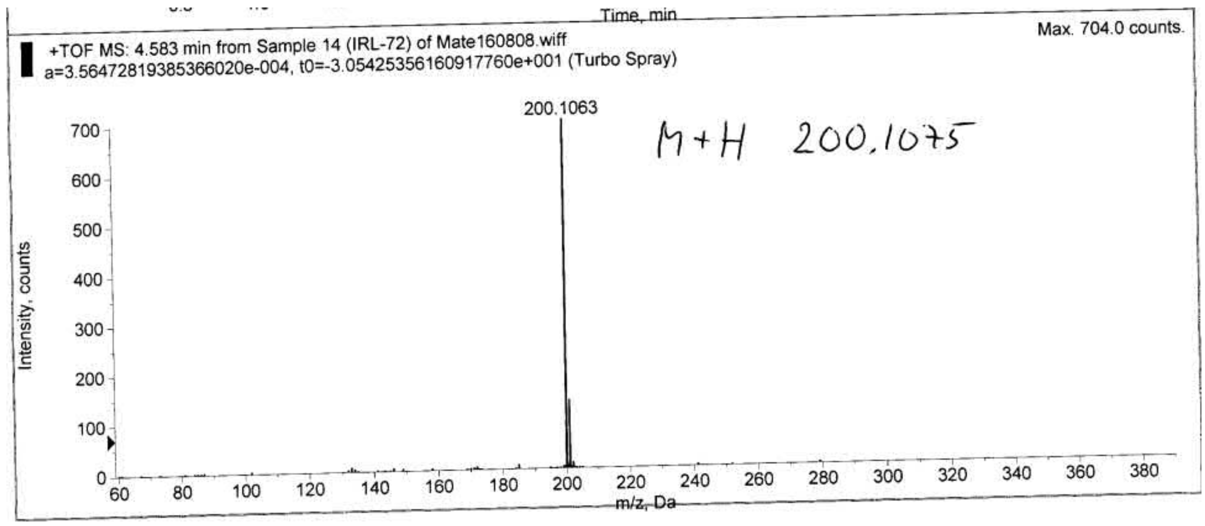

Figure S129. HRMS spectrum of 2-((triisopropylsilyl)ethynyl)-5a,6,7,8,9,9a-hexahydrobenzofuro[3,2-*s*]pyridine (**27**).

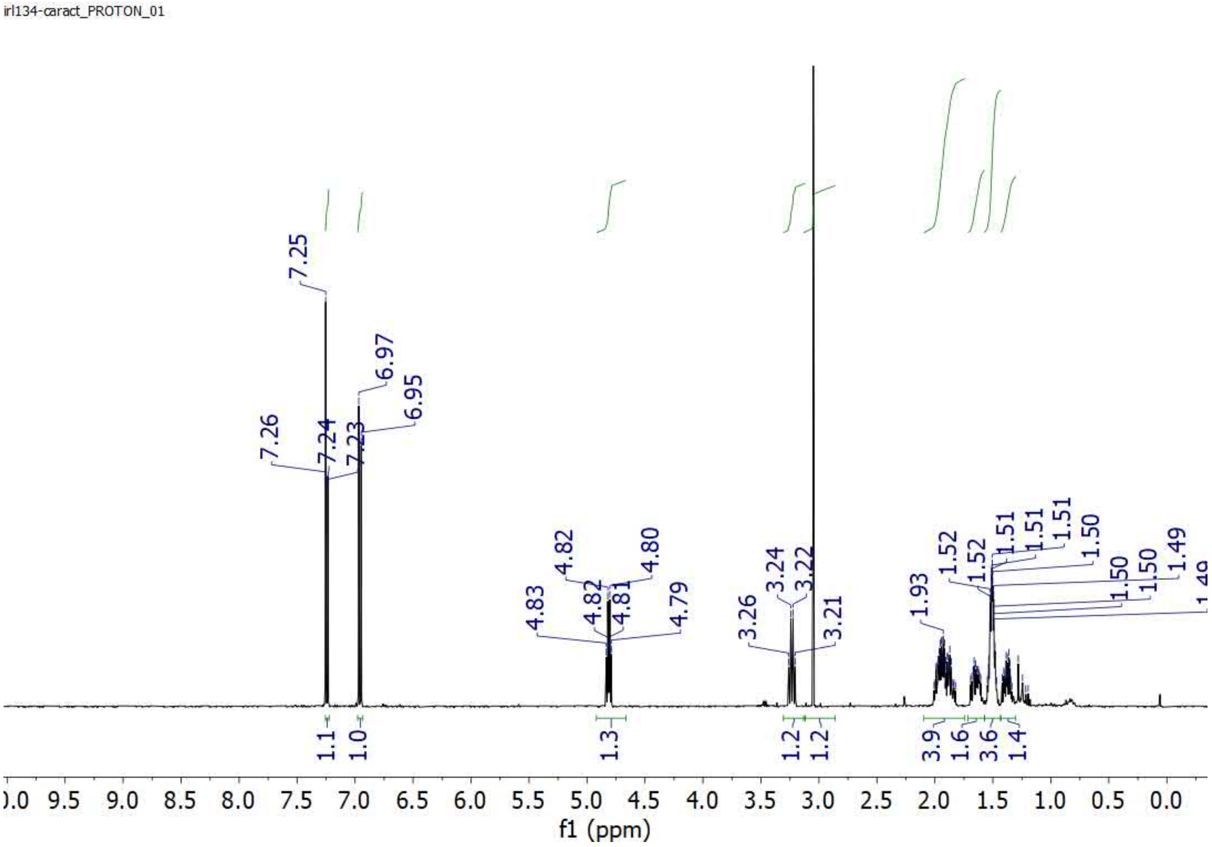

Figure S130. ^{1}H NMR spectrum of (**28**) in $CDCl_3$ at 25°C and 399.9 MHz.

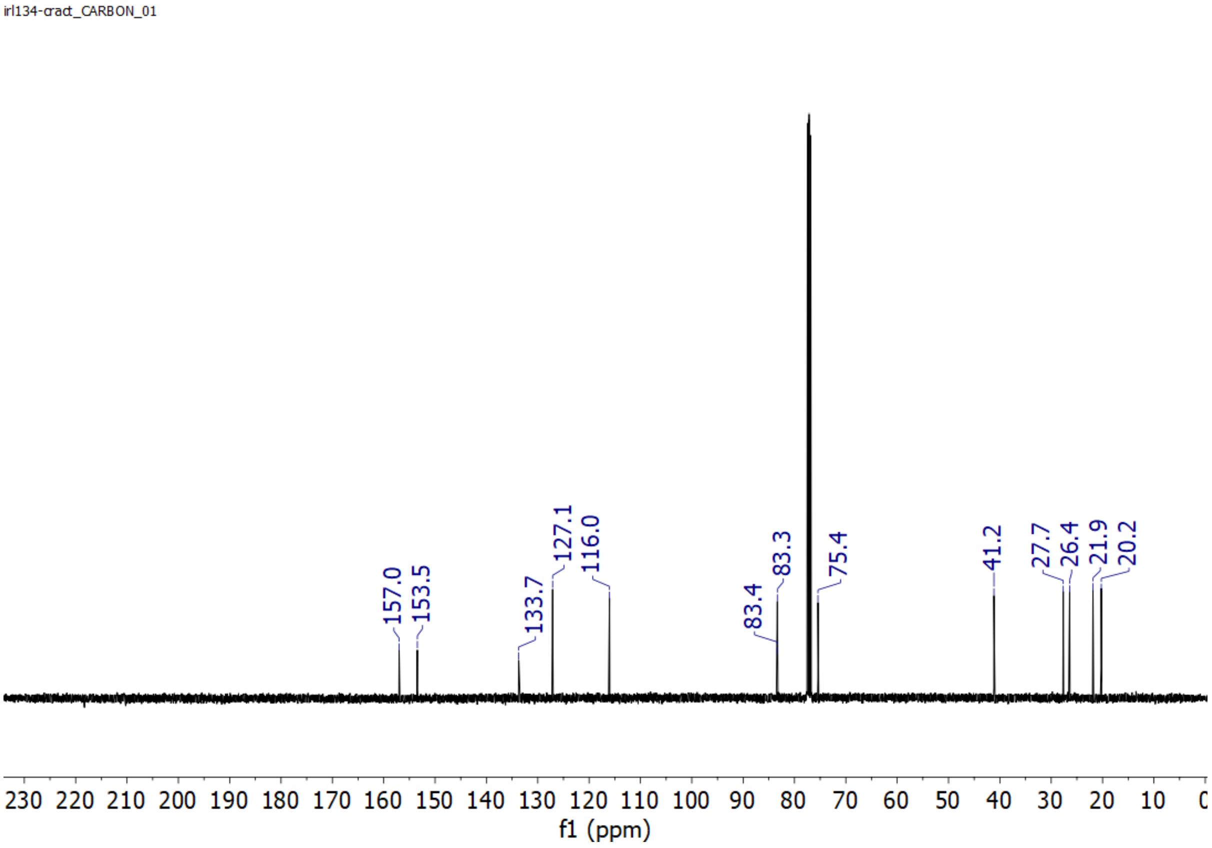

Figure S131. ^{13}C NMR spectrum of (**28**) in $CDCl_3$ at 25°C, and 100.6 MHz.

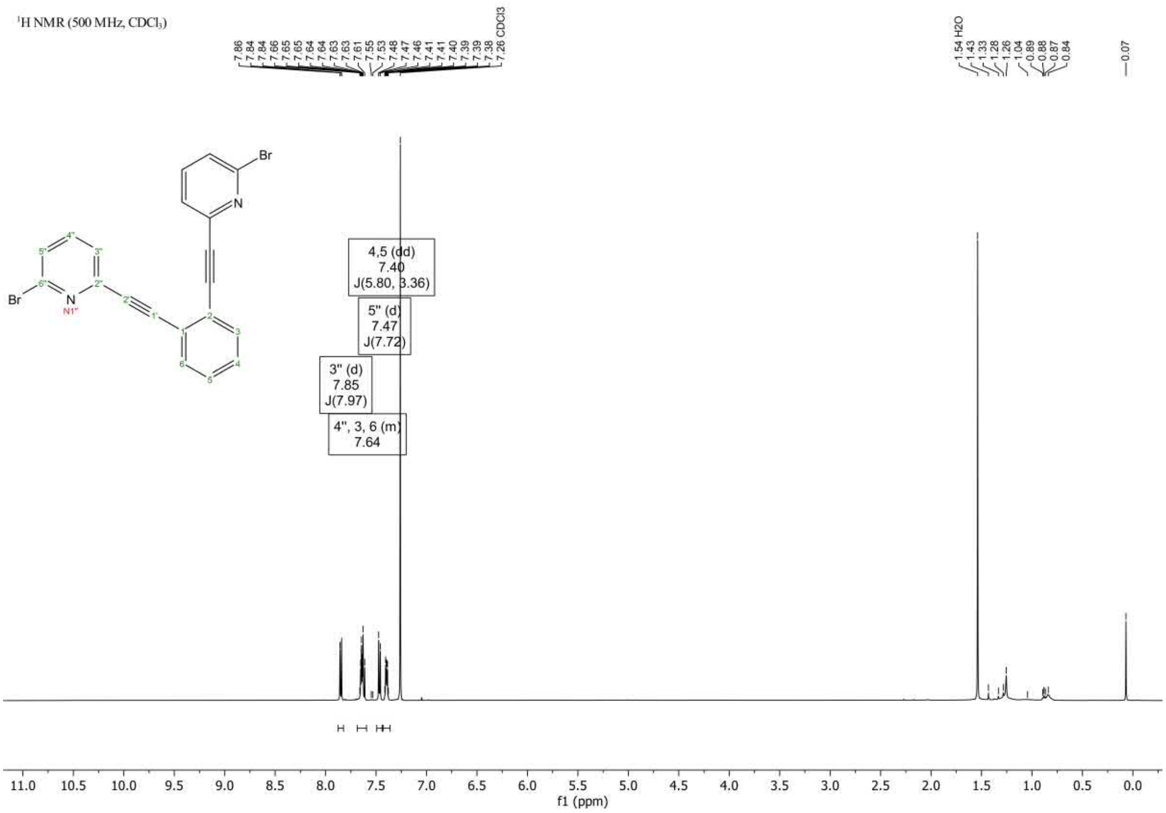

Figure S132. ^{1}H NMR spectrum of 1,2-bis((6-brompyridin-2-yl)ethynyl]benzene (**29**) in $CDCl_3$ at 25℃ and 500 MHz.

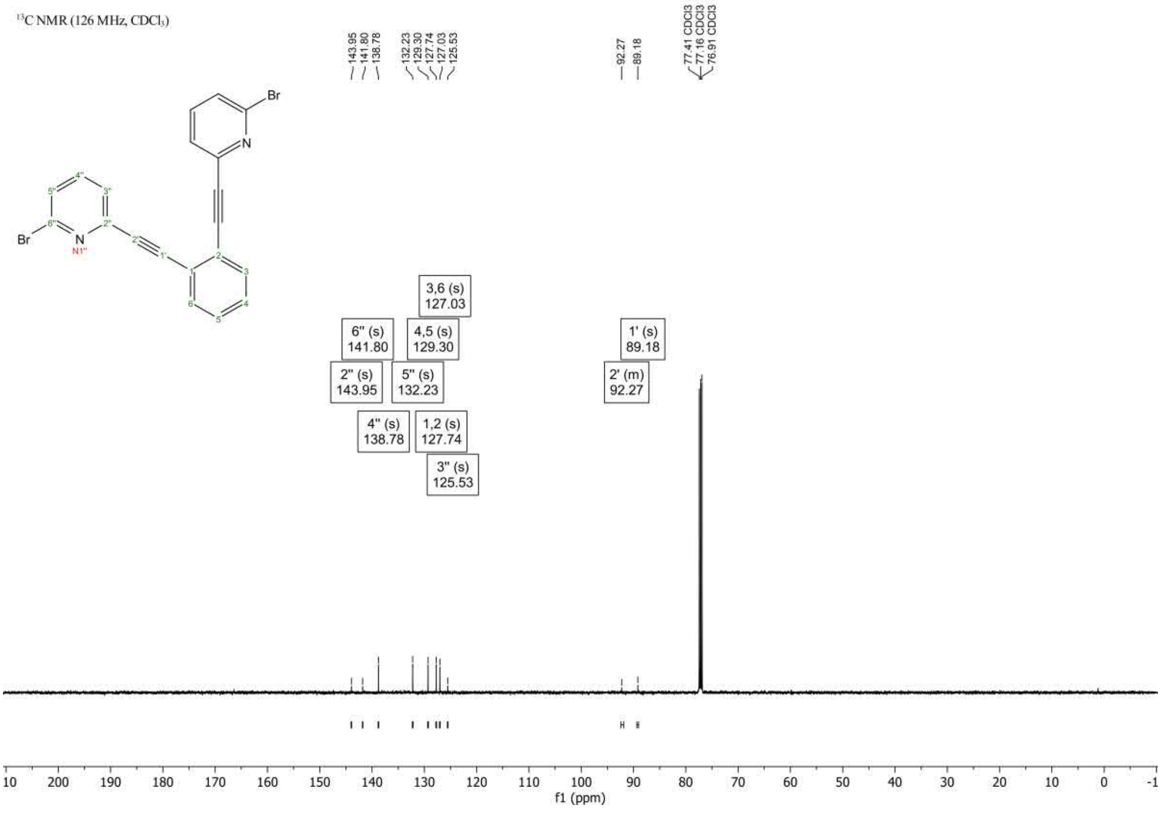

Figure S133. ^{13}C NMR spectrum of 1,2-bis((6-brompyridin-2-yl)ethynyl]benzene (**29**) in $CDCl_3$ at 25℃, and 126 MHz.

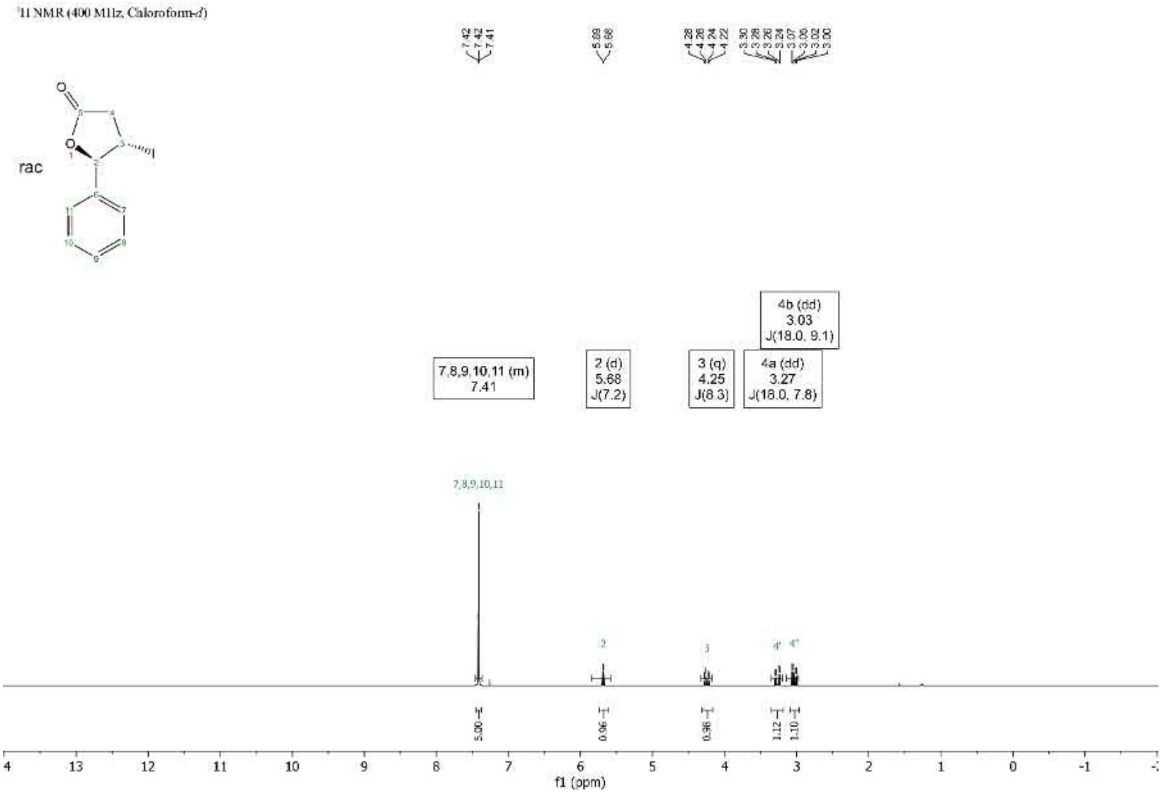

Figure S134. ^{1}H NMR spectrum of *rac*-4-Iodo-5-phenyldihydrofuran-2(3H)-one (**30**) in $CDCl_3$ at 25℃ and 500 MHz.

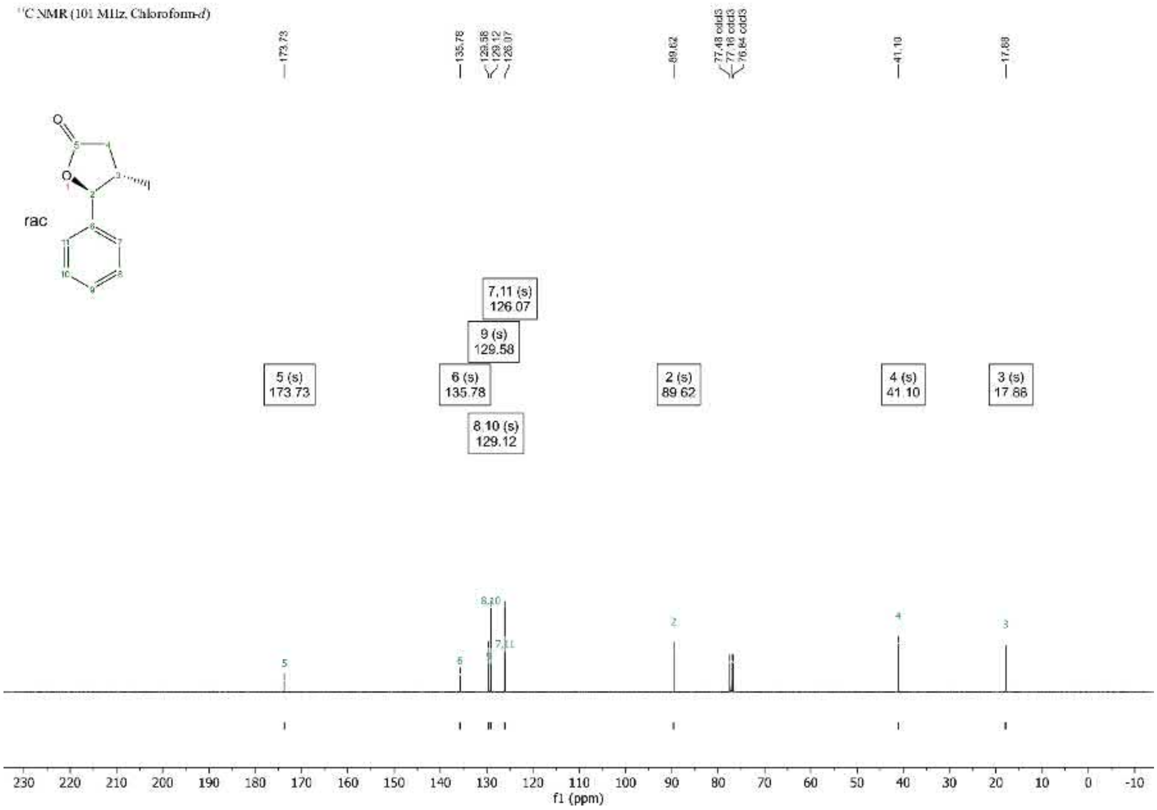

Figure S135. ^{13}C NMR spectrum of *rac*-4-Iodo-5-phenyldihydrofuran-2(3H)-one (**30**) in $CDCl_3$ at 25℃, and 126 MHz.

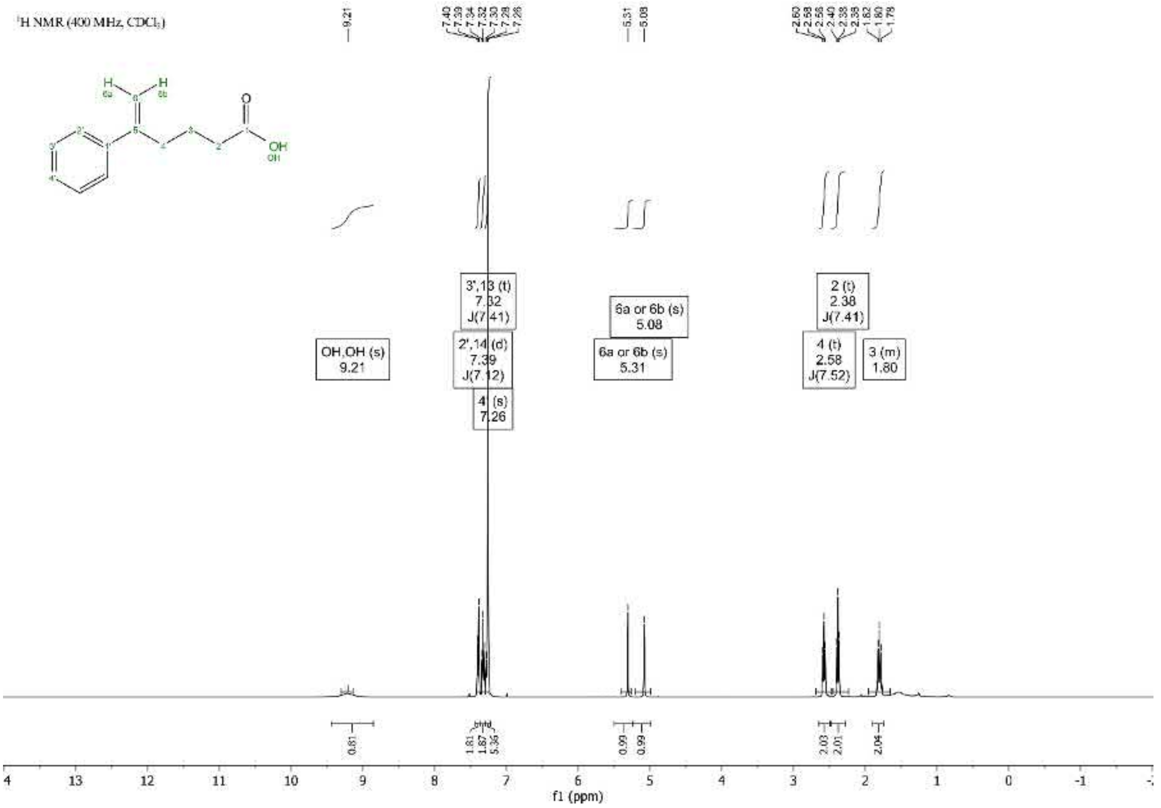

Figure S136. ^{1}H NMR spectrum of 5-phenyl-hex-5-enoic acid (**31**) in $CDCl_3$ at 25°C and 400 MHz.

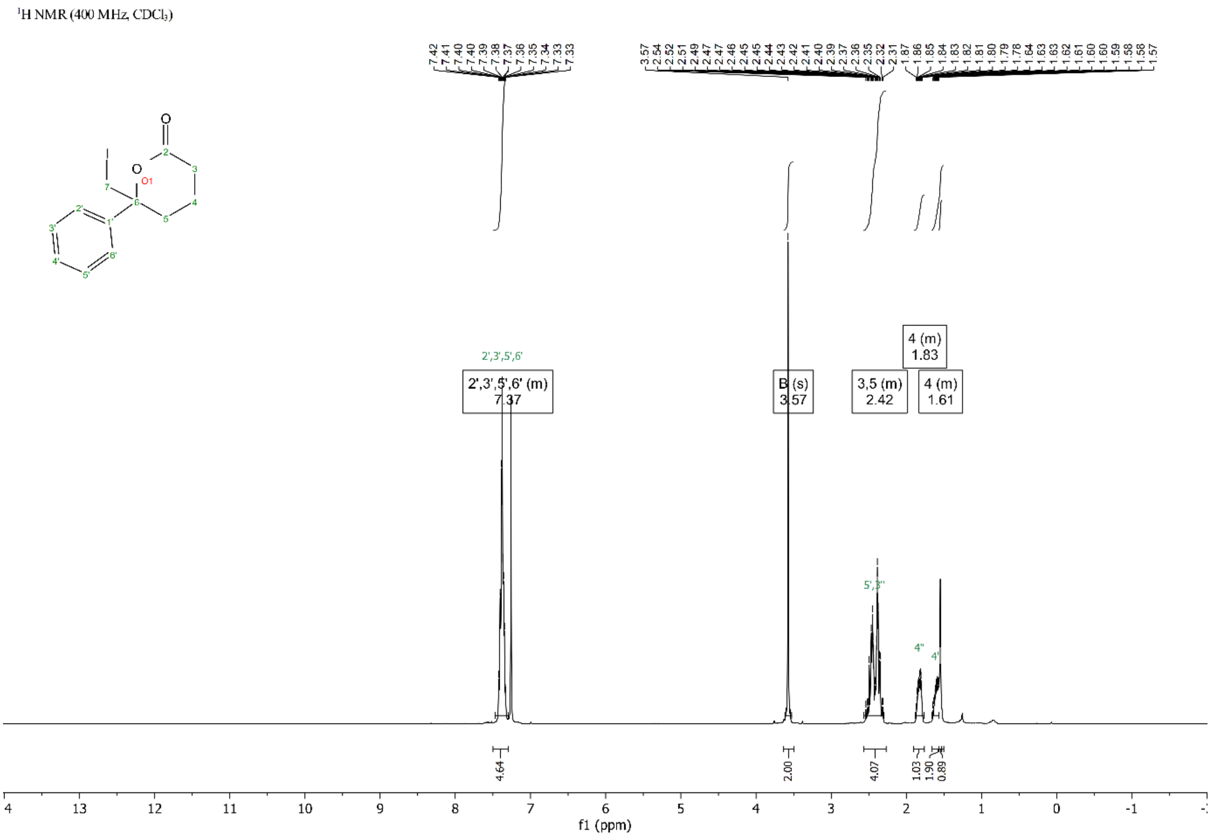

Figure S137. ^{1}H NMR spectrum of 6-(iodomethyl)-6-phenyltetrahydro-2H-pyran-2-one (**32**) in $CDCl_3$ at 25°C and 400 MHz.

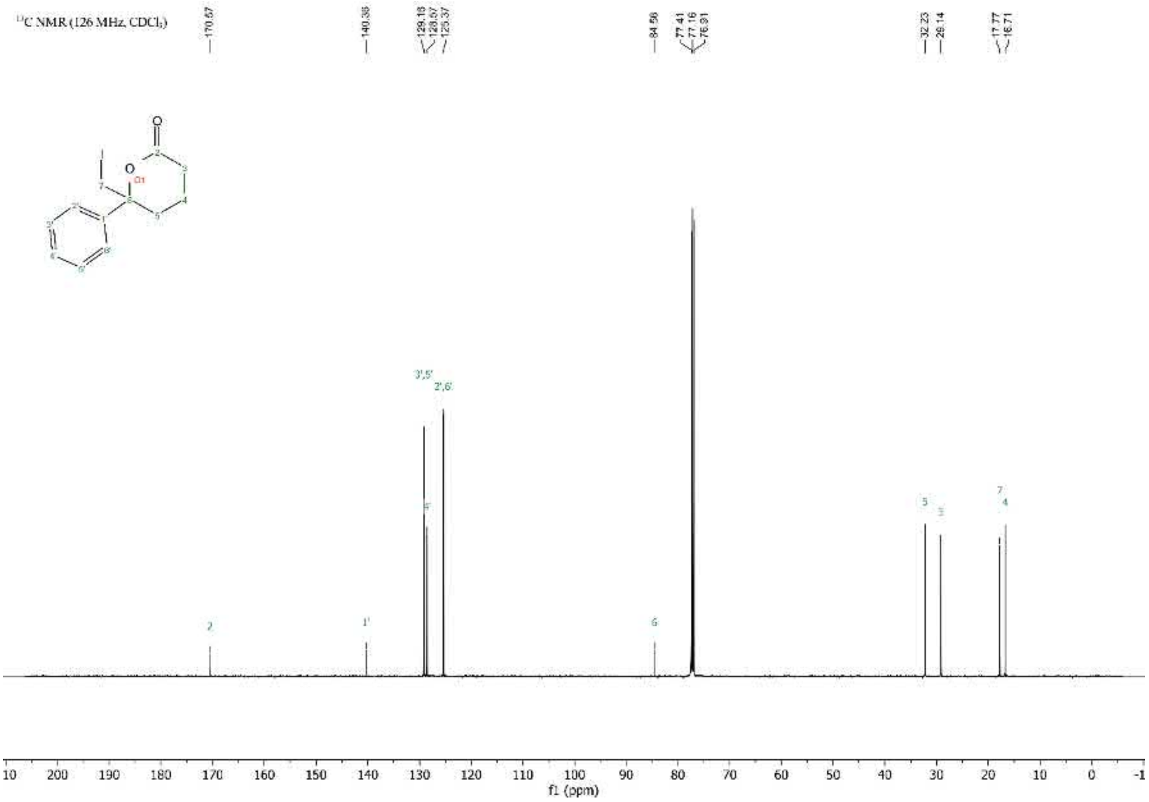

Figure S138. ^{13}C NMR spectrum of 6-(iodomethyl)-6-phenyltetrahydro-2H-pyran-2-one (**32**) in $CDCl_3$ at 25°C, and 126 MHz.

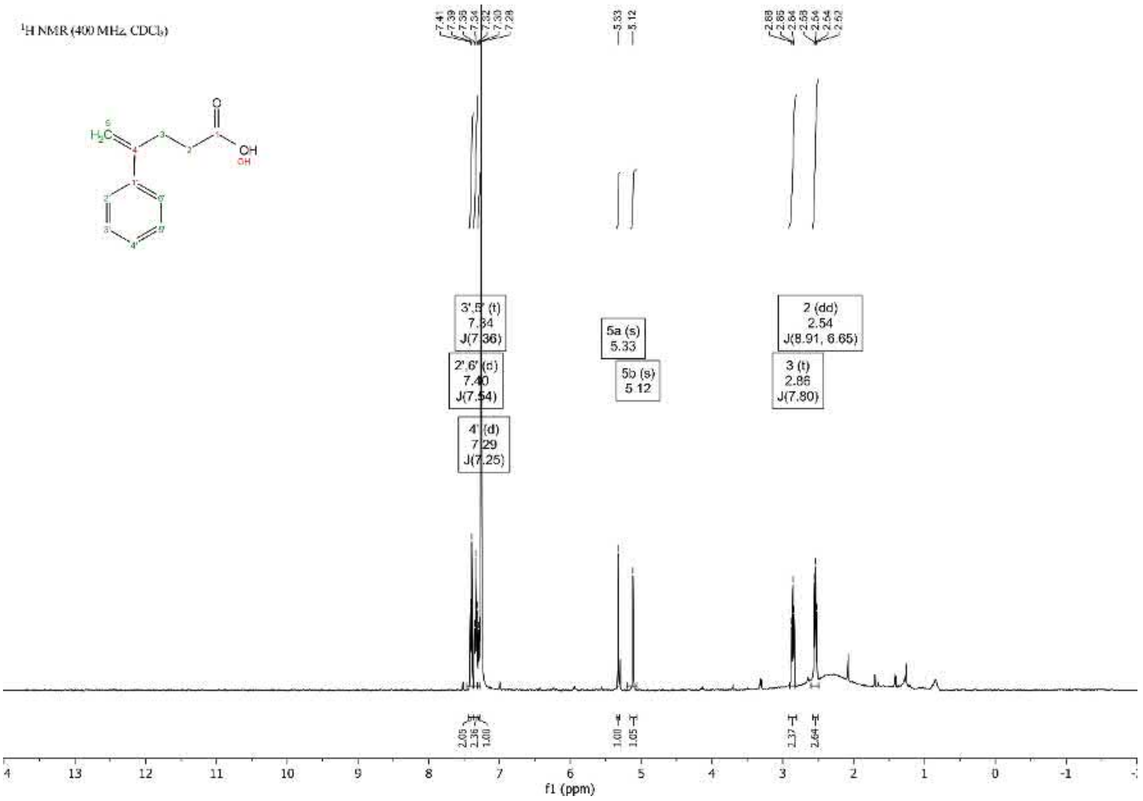

Figure S139. ^1H NMR spectrum of 4-phenyl-penten-5-enoic acid (**33**) in $CDCl_3$ at 25°C and 400 MHz.

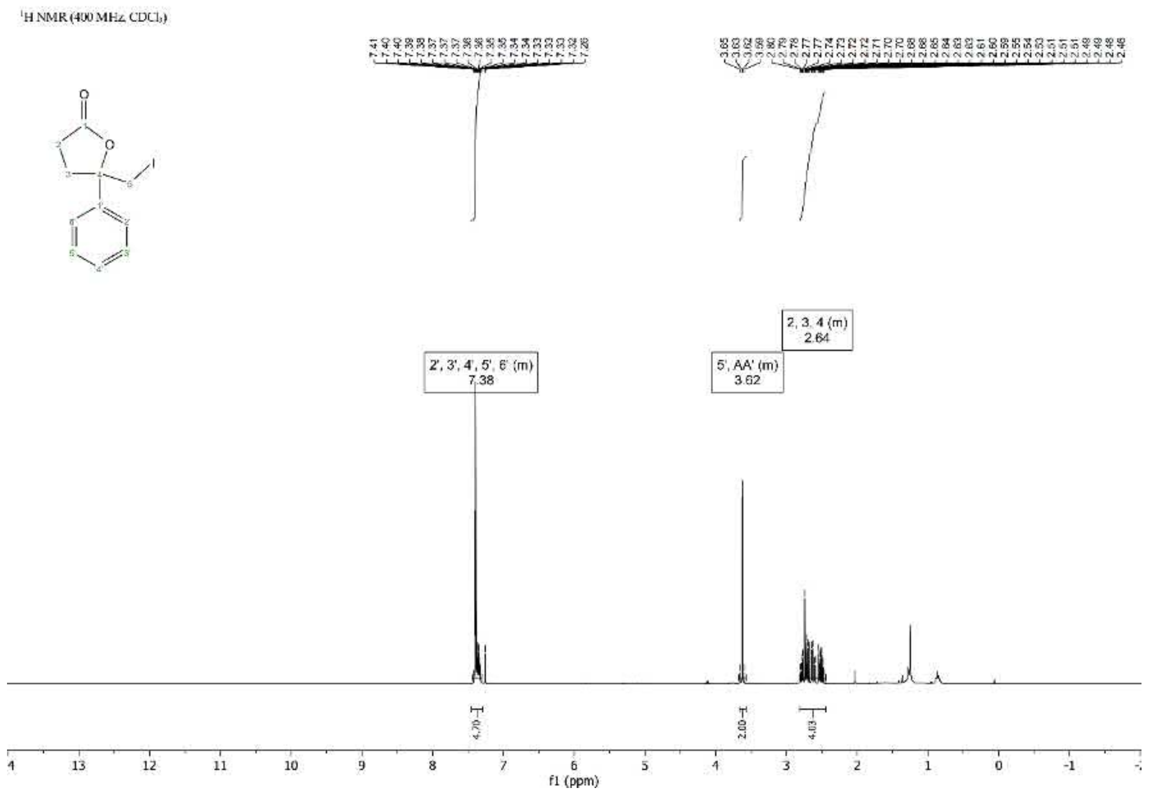

Figure S140. ^{1}H NMR spectrum of 5-(iodomethyl)-5-phenyldihydrofuran-2(3H)-one (**34**) in $CDCl_3$ at 25℃ and 400 MHz.

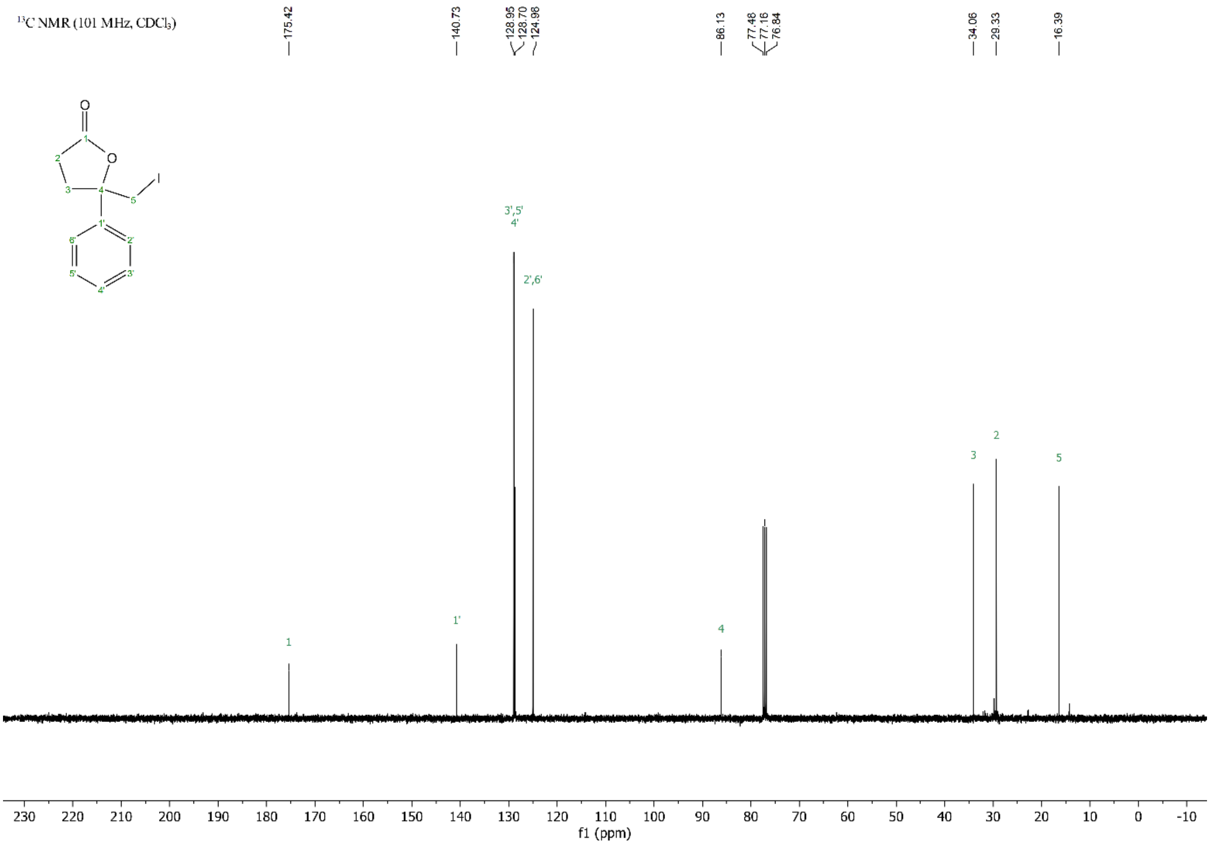

Figure S141. ^{13}C NMR spectrum of 5-(iodomethyl)-5-phenyldihydrofuran-2(3H)-one (**34**) in $CDCl_3$ at 25℃, and 126 MHz.

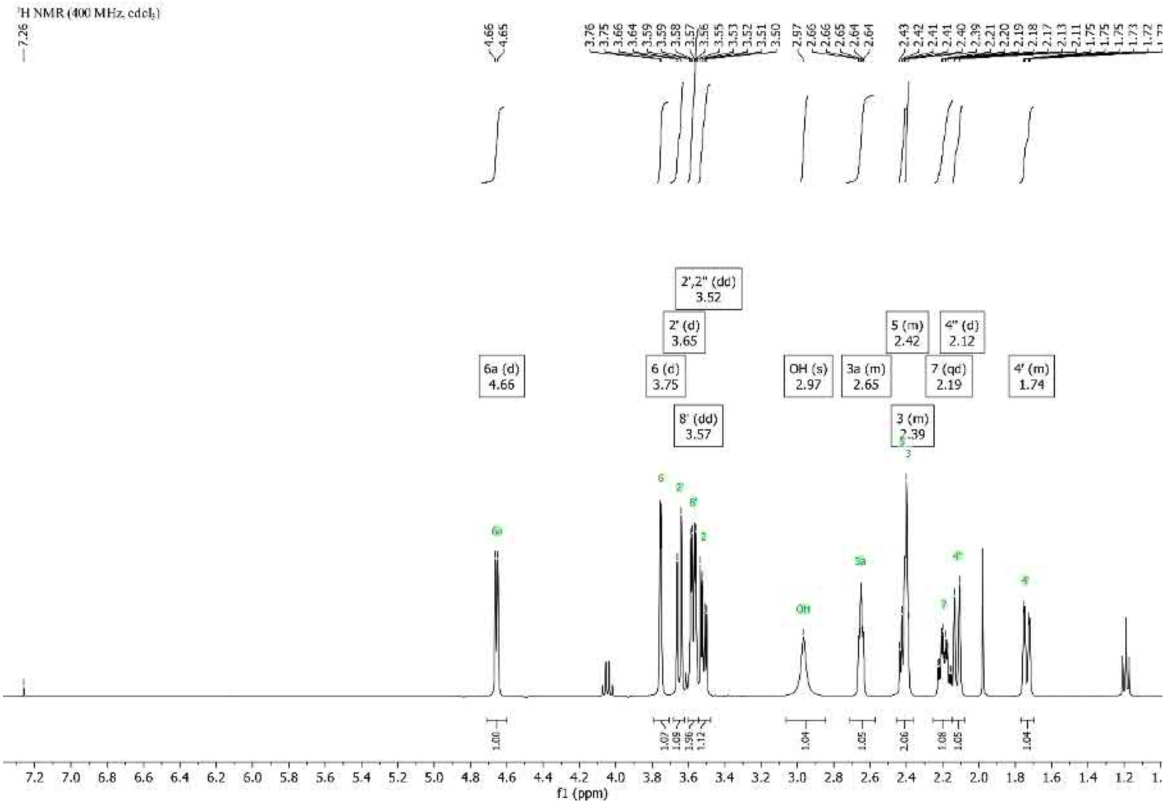

Figure S142. ^{1}H NMR spectrum of rac-(6-iodohexahydro-2H-3,5-methanocyclopenta[b]furan-7-yl)methanol (**35**) in $CDCl_3$ at 25°C and 400 MHz.

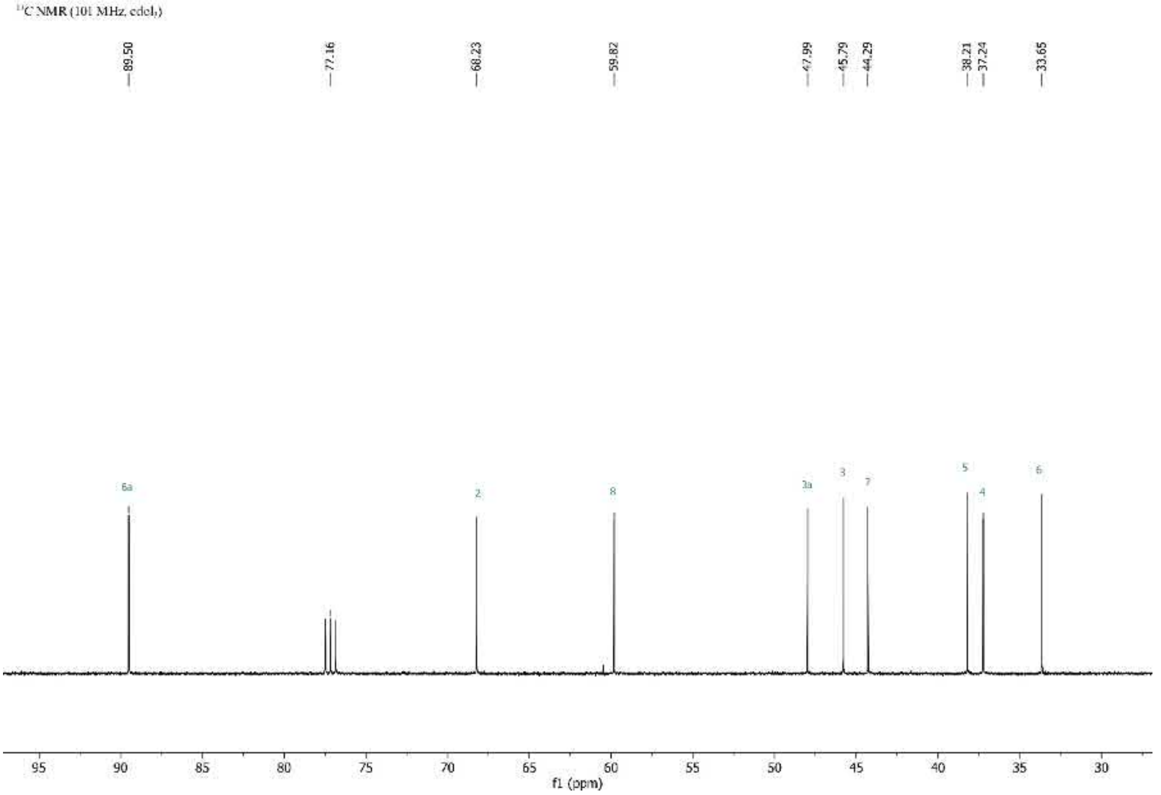

Figure S143. ^{13}C NMR spectrum of rac-(6-iodohexahydro-2H-3,5-methanocyclopenta[b]furan-7-yl)methanol (**35**) in $CDCl_3$ at 25°C, and 126 MHz.

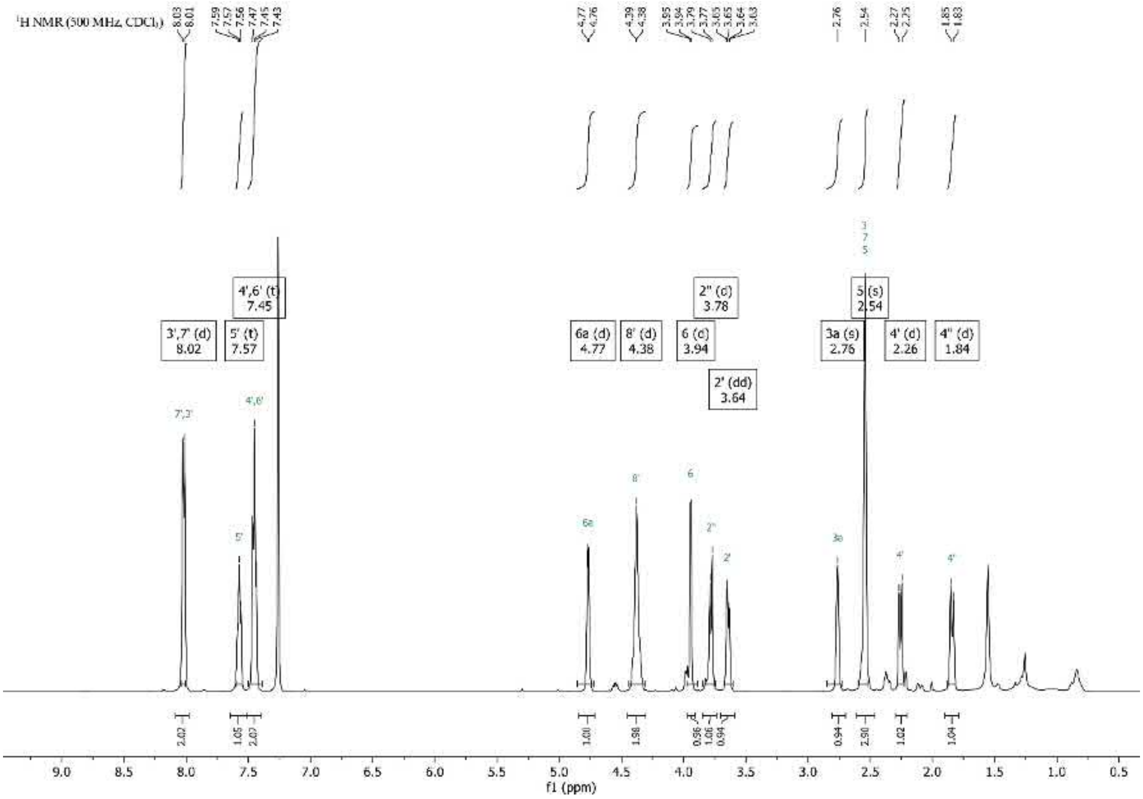

Figure S144. ^{1}H NMR spectrum *rac*-6-iodohexahydro-2H-3,5-methanocyclopenta[*b*]furan-7-yl)methyl benzoate (**36**) in $CDCl_3$ at 25℃ and 400 MHz.

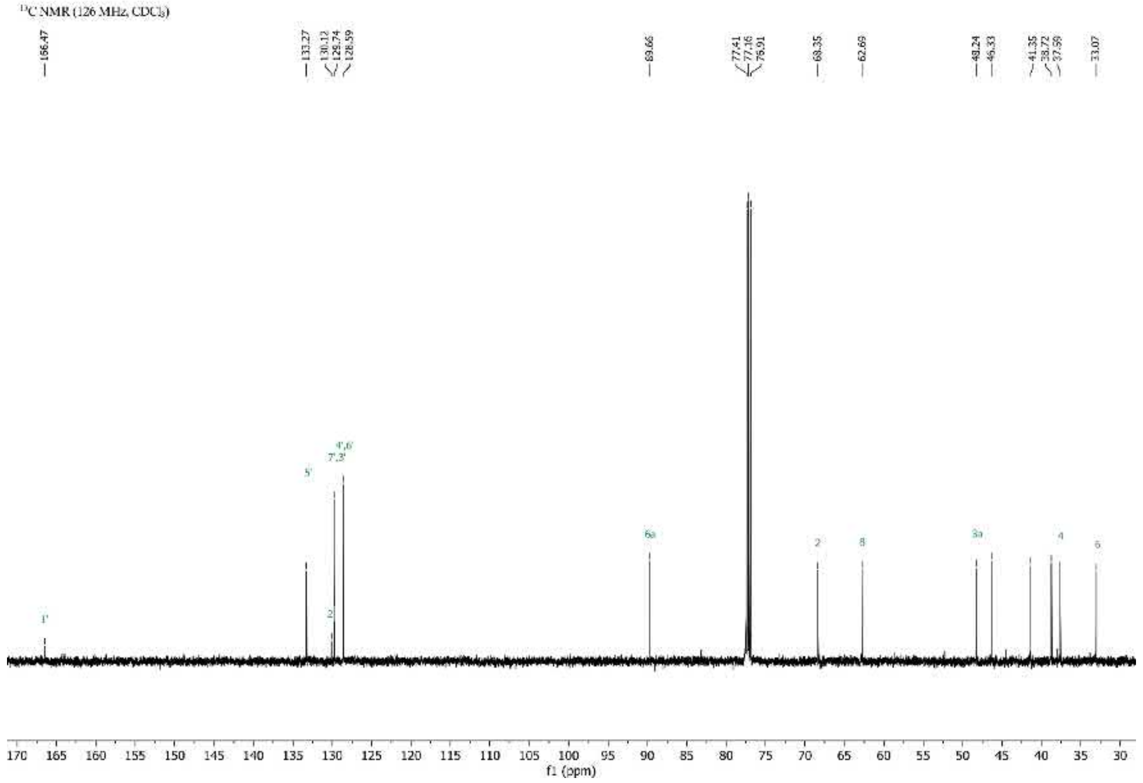

Figure S145. ^{13}C NMR spectrum of *rac*-6-iodohexahydro-2H-3,5-methanocyclopenta[*b*]furan-7-yl)methyl benzoate (**36**) in $CDCl_3$ at 25℃, and 126 MHz.

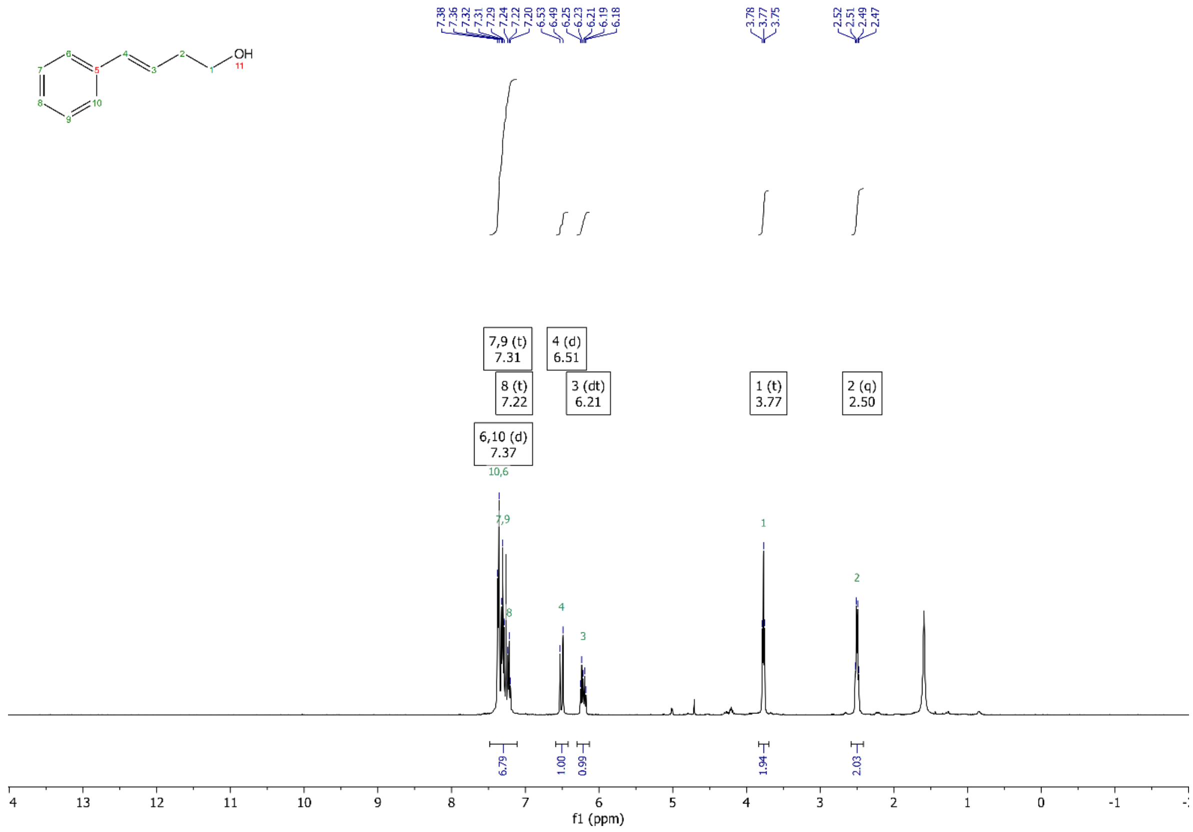

Figure S146. ^{1}H NMR spectrum 4-phenyl-but-3-enol (**37**) in $CDCl_3$ at 25°C and 400 MHz.

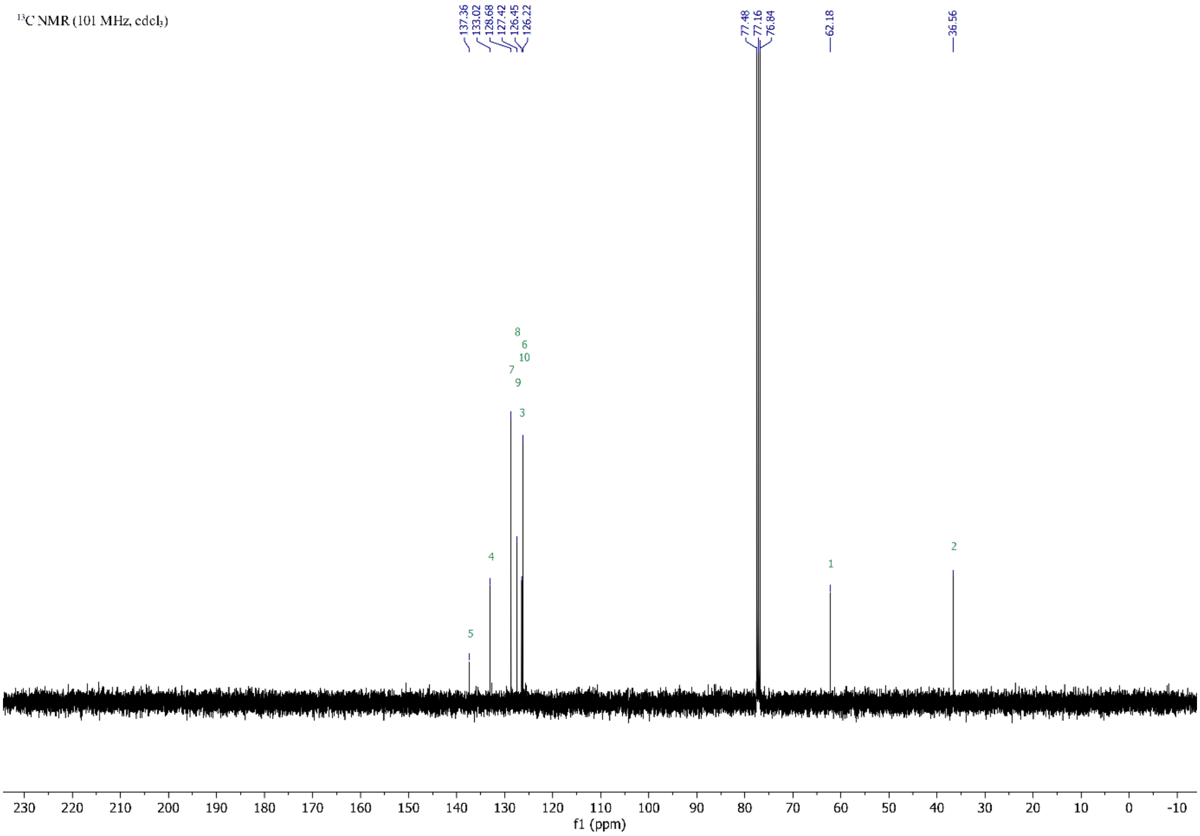

Figure S147. ^{13}C NMR spectrum of 4-phenyl-but-3-enol (**37**) in $CDCl_3$ at 25°C, and 101 MHz.

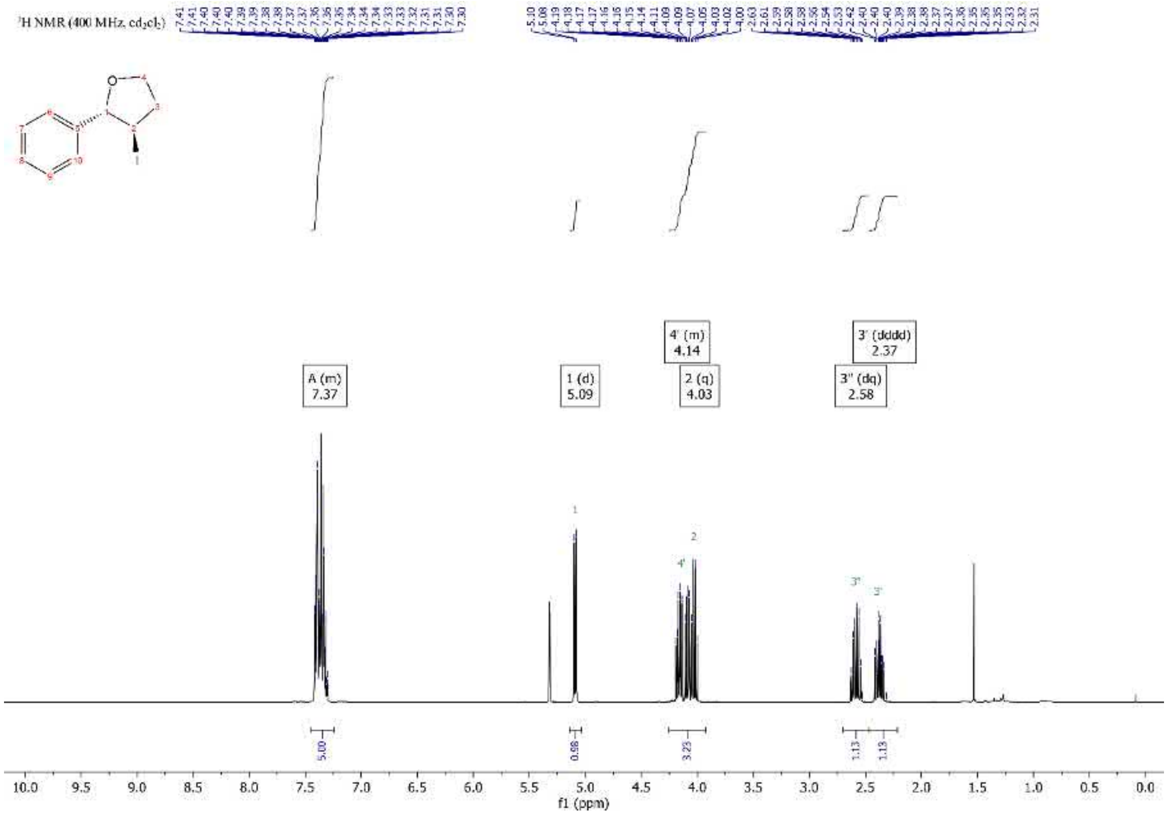

Figure S148. ^{1}H NMR spectrum rel-(1S,2R)-2-Iodo-1-phenyl-tetrahydrofuran (**38**) in $CDCl_3$ at 25℃ and 400 MHz.

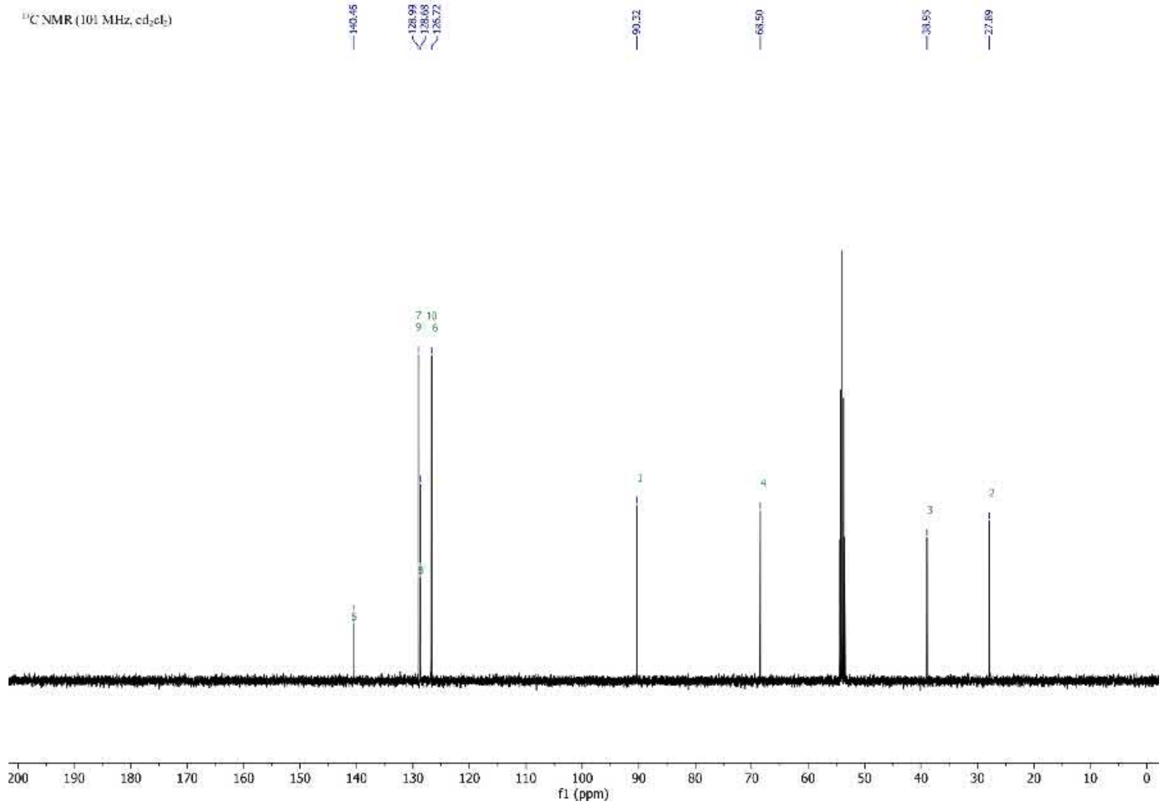

Figure S149. ^{13}C NMR spectrum of rel-(1S,2R)-2-Iodo-1-phenyl-tetrahydrofuran (**38**) in $CDCl_3$ at 25℃, and 101 MHz.

4. COMPUTATIONS

4.1. Optimized structures

The most stable form of complex [**4**-I]$^+$ is characterized by a strong, symmetric three-center, four-electron [N-I-N]$^+$ bond. Three, energetically close-lying conformers could be identified computationally for this form, which differ only in the conformation of the cyclohexyl groups of the chiral fused ring units (Figure S150). Among these conformers, the *chair-chair* combination is found to be the most favored structure (**A**$_1$). This structure is referred to as conformer **A** in the main text.

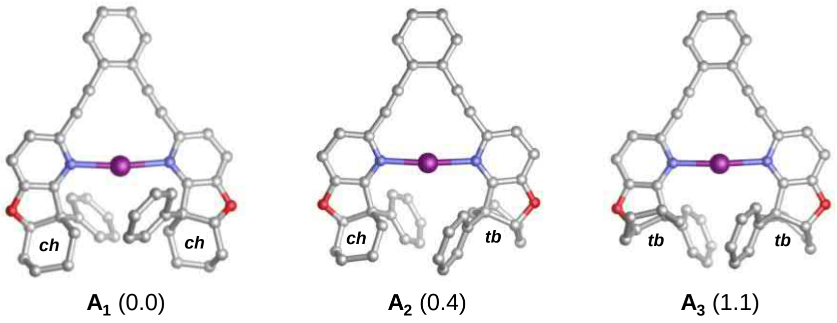

Figure S150. Optimized structures of the symmetric chelating forms of complex [**4**-I]$^+$. Labels *ch* and *tb* refer to the chair and twist-boat conformations of the fused cyclohexyl units. Relative stabilities (in terms of solution-phase Gibbs free energies) are given in kcal/mol. All H atoms are omitted for clarity.

The most favored singly coordinated (open) forms of complex [**4**-I]$^+$ identified computationally are depicted in Figure S151.

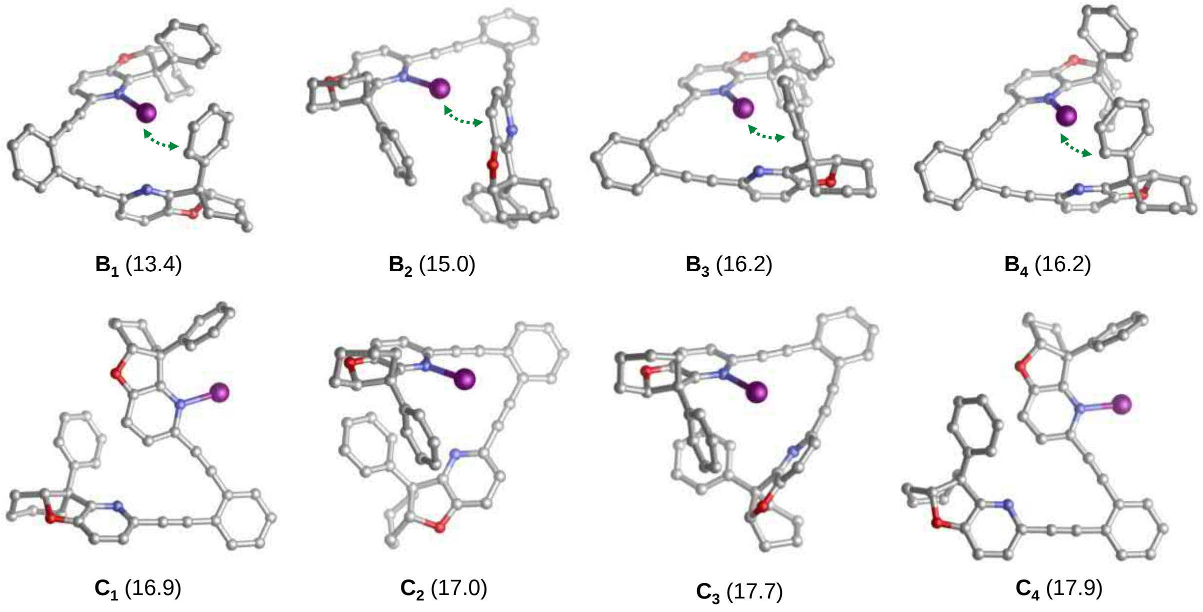

Figure S151. Optimized structures of the singly coordinated forms of complex [**4**-I]$^+$. Relative stabilities (in terms of solution-phase Gibbs free energies) are given in kcal/mol (with respect to conformer **A**$_1$). Close I$^+$–π contacts in B-type structures are highlighted with green dotted arrows.

These conformers are far less stable than the symmetric (closed) forms, and they can be classified into two groups according to the steric environment of the singly coordinated iodonium ion. In the **B**$_i$ series, the I$^+$ ion is in close contact with one of the aromatic rings of the ligand, either with the phenyl substituent, or with the pyridine unit, whereas these stabilizing interactions are absent in the **C**$_i$ conformers. These later forms can thus

be regarded as the reactive forms of complex [**4**-I]$^+$, because the iodonium center is readily available for substrate binding and electrophilic attack on the olefinic bond. Only a selection from the $\mathbf{B}_i$ and $\mathbf{C}_i$ conformers is presented in the paper (in Figure 1).

4.2. Total energy data

Table S18. Total energy data (in a.u.) computed for M06-2X/Def2-SVP optimized structures.[a]

conformer	$E_{0,sol}$	$G_{0,sol}$	$E_{0,sol}'$	G
closed forms				
A_1	-2253.5061	-2252.8743	-2255.6635	-2255.0317
A_2	-2253.5072	-2252.8749	-2255.6633	-2255.0310
A_3	-2253.5068	-2252.8741	-2255.6626	-2255.0300
open forms				
B_1	-2253.4804	-2252.8497	-2255.6411	-2255.0104
B_2	-2253.4773	-2252.8459	-2255.6392	-2255.0078
B_3	-2253.4750	-2252.8442	-2255.6368	-2255.0059
B_4	-2253.4788	-2252.8468	-2255.6379	-2255.0059
C_1	-2253.4743	-2252.8441	-2255.6350	-2255.0048
C_2	-2253.4764	-2252.8443	-2255.6367	-2255.0046
C_3	-2253.4771	-2252.8453	-2255.6353	-2255.0035
C_4	-2253.4736	-2252.8429	-2255.6339	-2255.0032

[a] $G = E_{0,sol}' + (G_{0,sol} - E_{0,sol}) + \Delta G_{conc}$, where $E_{0,sol}'$ and $E_{0,sol}$ are solution phase electronic energies obtained at the M06-2X/Def2TZVPP and M06-2X/Def2SVP levels, respectively, and $G_{0,sol}$ is solution phase Gibbs free energy computed at M06-2X/Def2SVP level. ΔG_{conc} = 0.003019 a.u. corresponding to c = 1 mol/dm^3 concentration.

4.3. Noncovalent interaction analysis

To identify the steric hindrance, we carried out the noncovalent interaction (NCI) analysis.[4,5] The NCI plots were calculated on the optimized geometries obtained with the ωB97X-D functional[6] in combination with the aug-cc-pVTZ basis.[7,8] The polarizable continuum model (PCM) of Tomasi and co-workers[9] was used to model the dichloromethane solvation effects. The plots were made with a standard gradient cutoff of s = 0.5 a.u. and a color scale $-0.04 < \rho < 0.04$ a.u..[4] The red, green, and blue colors represent repulsion, weak attraction, and strong attraction, respectively. All DFT calculations were carried out using the Gaussian 16 Rev. C.01 program package (g16_c01.enw).

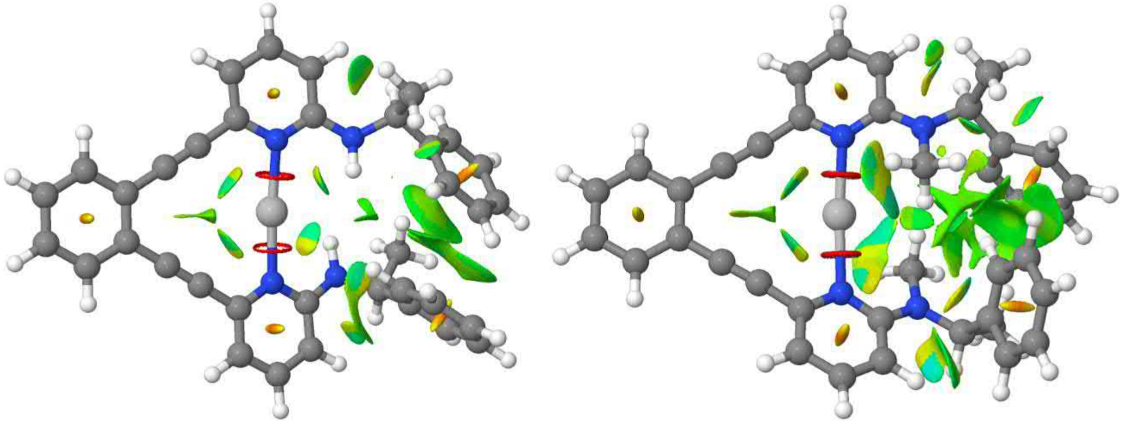

Figure S152. NCI plots of **6-Ag** and **7-Ag**. The red, yellow, green, and blue iso-surfaces represent strong repulsion, weak repulsion, weak attraction, and strong attraction, respectively, calculated at the ωB97X-D/aug-cc-pVTZ level of theory.

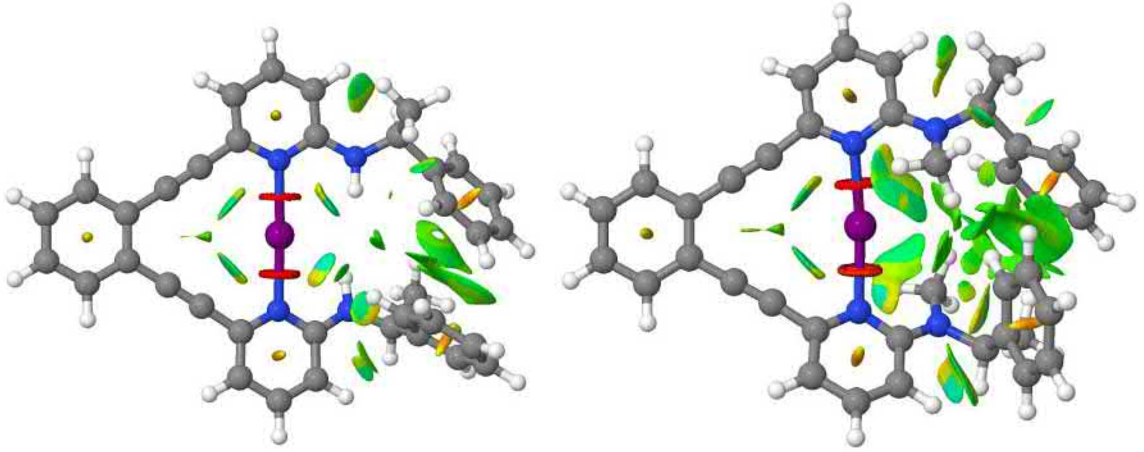

Figure S153. NCI plots of **6-I** and **7-I**. The red, yellow, green, and blue iso-surfaces represent strong repulsion, weak repulsion, weak attraction, and strong attraction, respectively, calculated at the ωB97X-D/aug-cc-pVTZ level of theory.

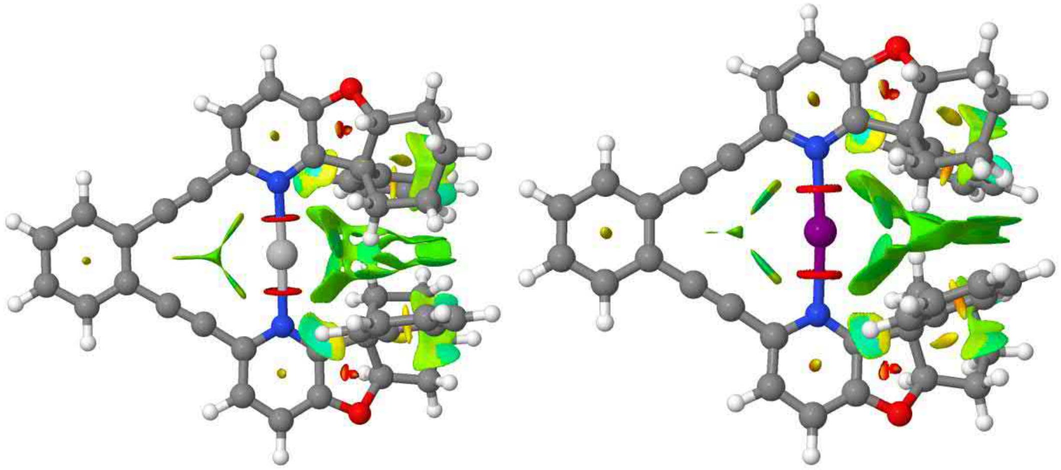

Figure S154. NCI plots of **4-Ag** and **4-I**. The red, yellow, green, and blue iso-surfaces represent strong repulsion, weak repulsion, weak attraction, and strong attraction, respectively, calculated at the ωB97X-D/aug-cc-pVTZ level of theory.

4.4. Cartesian coordinates

Cartesian coordinates of the optimized geometries are given below in standard XYZ format (units are in Å). The first line shows the number of atoms, the second line is the notation used for the conformers.

85

A$_1$

C	2.666598	-4.560769	9.030515
C	2.309835	-5.476176	8.045697
C	2.239663	-5.080043	6.701663
C	2.543482	-3.740210	6.353195
C	2.895847	-2.828822	7.360018
C	2.955272	-3.237468	8.688462
H	2.716966	-4.880658	10.072077
H	3.126600	-1.798911	7.085680
H	3.231623	-2.520002	9.462127
C	2.522051	-3.325296	4.983149
C	2.548604	-2.984949	3.816675
C	2.976673	-3.040268	0.224142
C	3.334475	-1.712753	-0.062595
C	3.356911	-0.754999	0.938091
C	3.064830	-1.197341	2.226404
C	2.724722	-2.532701	2.469068
N	2.634106	-3.427564	1.443825
H	3.621306	0.282041	0.730565
H	3.112912	-0.513403	3.073123
C	1.831491	-6.015274	5.697639
C	1.438446	-6.795196	4.852430
C	-0.164083	-8.172567	1.913808
C	-0.529128	-9.459791	2.341246
C	-0.168196	-9.917123	3.598120
C	0.514321	-9.017236	4.413076
C	0.847558	-7.739099	3.951889
N	0.545531	-7.355345	2.678468
H	-0.434385	-10.917379	3.939961
H	0.785344	-9.286583	5.433330
H	2.076499	-6.509161	8.306139
I	1.573184	-5.425993	1.928077
O	-1.222966	-10.127718	1.416714
O	3.624958	-1.529952	-1.352918
C	3.005371	-3.827393	-1.075296
C	3.883804	-2.840065	-1.915942
H	4.943406	-3.058958	-1.707858
C	-0.659921	-7.990948	0.488605
C	-1.717122	-9.147331	0.471179
H	-2.657705	-8.760034	0.894589
C	-1.359101	-6.654749	0.264138
C	-2.187299	-6.138103	1.273389
C	-1.229046	-5.929329	-0.923246
C	-2.850649	-4.925847	1.107128
H	-2.308909	-6.686739	2.210876
C	-1.901626	-4.716744	-1.096062
H	-0.590192	-6.295013	-1.727622
C	-2.709291	-4.207970	-0.082180
H	-3.479715	-4.539431	1.910763
H	-1.786045	-4.167021	-2.032229
H	-3.228851	-3.257867	-0.216546
C	3.719486	-5.167419	-0.940234
C	3.269000	-6.324429	-1.580889
C	4.882738	-5.247621	-0.158230
C	3.954376	-7.533428	-1.436719
H	2.368728	-6.299083	-2.195358
C	5.562579	-6.452832	-0.006266
H	5.258739	-4.355515	0.349700
C	5.098401	-7.604091	-0.645707

H	3.584576	-8.425560	-1.945830
H	6.458099	-6.493082	0.616055
H	5.629023	-8.550339	-0.528408
C	1.550950	-3.904608	-1.610588
H	1.061489	-4.778217	-1.157595
H	1.002664	-3.022416	-1.240598
C	3.638106	-2.822286	-3.407008
H	4.068273	-3.749013	-3.819973
H	4.208659	-1.984660	-3.834430
C	2.157183	-2.740664	-3.755552
H	2.030541	-2.734811	-4.847194
H	1.737811	-1.795181	-3.374031
C	1.418362	-3.920975	-3.134170
H	0.348480	-3.887275	-3.389002
H	1.817715	-4.855391	-3.558877
C	0.532210	-8.283418	-0.461740
H	1.096845	-7.350770	-0.603722
H	1.216304	-8.978224	0.052889
C	-1.975784	-9.801575	-0.865985
H	-2.618390	-10.677918	-0.695910
H	-2.556696	-9.088152	-1.472651
C	0.158808	-8.920031	-1.801801
H	1.086941	-9.165717	-2.339341
H	-0.399702	-8.215357	-2.437279
C	-0.688596	-10.170472	-1.592774
H	-0.925782	-10.643241	-2.556089
H	-0.125795	-10.908377	-0.997635

85
A_2

C	2.564634	-4.384431	9.210056
C	2.019450	-5.241200	8.259086
C	2.136195	-4.946606	6.892678
C	2.814431	-3.770836	6.487571
C	3.358611	-2.918137	7.459357
C	3.233171	-3.224542	8.810636
H	2.467632	-4.622596	10.270101
H	3.880412	-2.015013	7.140953
H	3.659868	-2.554015	9.557723
C	2.944287	-3.460022	5.096760
C	3.053037	-3.203681	3.913891
C	3.012283	-3.114422	0.279569
C	3.681406	-1.911747	0.011714
C	4.168022	-1.120185	1.042911
C	3.948857	-1.581246	2.333821
C	3.257472	-2.779012	2.561960
N	2.787826	-3.524253	1.524773
H	4.690927	-0.184870	0.842932
H	4.305388	-1.017860	3.195411
C	1.576880	-5.825167	5.911367
C	1.105581	-6.565091	5.070544
C	-0.138483	-8.160768	2.051387
C	-0.638542	-9.374537	2.551387
C	-0.571376	-9.673906	3.902954
C	0.008184	-8.712820	4.722805
C	0.514720	-7.522978	4.186198
N	0.451081	-7.275576	2.847631
H	-0.958496	-10.613639	4.296688
H	0.083815	-8.869188	5.798248
H	1.495723	-6.148102	8.563350
I	1.576132	-5.424340	2.081121
O	-1.133002	-10.164194	1.599542
O	3.759583	-1.625608	-1.284085
C	2.544151	-3.716719	-1.033054
C	3.263827	-2.748888	-2.051864
H	4.144552	-3.251197	-2.476681
C	-0.268438	-8.184997	0.535103

C	-1.309824	-9.355728	0.413426
H	-2.319397	-8.920890	0.485878
C	-0.906632	-6.946491	-0.081742
C	-0.762606	-6.675741	-1.447014
C	-1.758449	-6.127666	0.674197
C	-1.471386	-5.632932	-2.046710
H	-0.100581	-7.287281	-2.059242
C	-2.443385	-5.067809	0.081991
H	-1.897898	-6.318410	1.741174
C	-2.311649	-4.822763	-1.286008
H	-1.355781	-5.450825	-3.117101
H	-3.093700	-4.438929	0.692504
H	-2.861457	-4.004404	-1.754159
C	3.034301	-5.139478	-1.293368
C	2.558016	-5.846242	-2.407700
C	4.053488	-5.719614	-0.527298
C	3.087257	-7.091915	-2.745245
H	1.780759	-5.415737	-3.041625
C	4.570316	-6.974288	-0.852974
H	4.463880	-5.192297	0.336166
C	4.093437	-7.663727	-1.966595
H	2.710870	-7.615935	-3.625864
H	5.359844	-7.406664	-0.235902
H	4.507039	-8.638831	-2.229550
C	1.010491	-3.552088	-1.151896
H	0.666700	-4.091973	-2.042664
H	0.515070	-4.033840	-0.294770
C	2.349346	-2.237160	-3.142709
H	2.010704	-3.118940	-3.708275
H	2.930132	-1.618006	-3.840953
C	1.156902	-1.456149	-2.575736
H	0.368919	-1.418640	-3.341452
H	1.458250	-0.416491	-2.386148
C	0.609083	-2.084388	-1.276990
H	0.973624	-1.528361	-0.397425
H	-0.486839	-2.007618	-1.247842
C	1.136443	-8.561293	-0.038601
H	1.674250	-7.630852	-0.282548
H	1.706706	-9.048507	0.769186
C	-1.195276	-10.234304	-0.809758
H	-1.901255	-11.069608	-0.692845
H	-1.530677	-9.645484	-1.678796
C	1.150662	-9.518554	-1.235891
H	0.853033	-9.004315	-2.162626
H	2.188863	-9.847444	-1.388964
C	0.233324	-10.716654	-1.022746
H	0.278749	-11.393122	-1.887684
H	0.561331	-11.293022	-0.141849

85

A_3

C	2.451403	-4.900018	9.172635
C	1.806505	-5.617584	8.170405
C	1.975855	-5.262649	6.823739
C	2.808319	-4.165022	6.491730
C	3.451097	-3.452492	7.515110
C	3.273265	-3.818981	8.845271
H	2.312438	-5.185781	10.216101
H	4.089911	-2.608562	7.252581
H	3.778568	-3.257590	9.632208
C	2.989109	-3.778412	5.125222
C	3.139104	-3.445507	3.966051
C	3.089375	-3.182522	0.341027
C	3.783431	-1.984011	0.123923
C	4.300070	-1.255394	1.187111
C	4.082773	-1.770320	2.458456
C	3.362567	-2.958887	2.637761

N	2.869303	-3.642496	1.569531
H	4.843884	-0.324603	1.025229
H	4.460683	-1.255517	3.341088
C	1.311088	-6.006040	5.796668
C	0.749397	-6.641111	4.925981
C	-0.355033	-8.069049	1.772976
C	-1.274939	-9.038709	2.193118
C	-1.560533	-9.215024	3.541013
C	-0.869910	-8.406333	4.433980
C	0.047385	-7.450532	3.976118
N	0.276930	-7.288736	2.644623
H	-2.281448	-9.961027	3.875411
H	-1.028999	-8.499247	5.507801
H	1.163954	-6.462989	8.418743
I	1.598947	-5.510530	2.028031
O	-1.807588	-9.729971	1.191381
O	3.855244	-1.639094	-1.157949
C	2.602029	-3.717468	-0.995587
C	3.343216	-2.722455	-1.971861
H	4.217872	-3.223415	-2.410823
C	-0.268605	-8.098898	0.256195
C	-1.113356	-9.402003	-0.039114
H	-1.888484	-9.202637	-0.788645
C	-0.937819	-6.879558	-0.384978
C	-1.679785	-5.953418	0.357973
C	-0.868888	-6.713319	-1.776589
C	-2.321178	-4.882295	-0.268441
H	-1.772990	-6.056534	1.441093
C	-1.518900	-5.651738	-2.403598
H	-0.315072	-7.428941	-2.387303
C	-2.244087	-4.726655	-1.650930
H	-2.890111	-4.171316	0.333308
H	-1.455836	-5.547013	-3.488514
H	-2.750637	-3.893216	-2.140818
C	3.063309	-5.137308	-1.318033
C	2.561776	-5.790597	-2.452653
C	4.075444	-5.769162	-0.582736
C	3.054851	-7.037542	-2.837723
H	1.787867	-5.320999	-3.061880
C	4.555698	-7.024688	-0.956259
H	4.506766	-5.282770	0.294366
C	4.049294	-7.663844	-2.087738
H	2.653626	-7.521713	-3.730252
H	5.337683	-7.499679	-0.361101
H	4.431596	-8.641891	-2.385303
C	1.070130	-3.516431	-1.106863
H	0.709698	-4.021827	-2.012671
H	0.566396	-4.010713	-0.262923
C	2.445127	-2.154828	-3.048025
H	2.097450	-3.009448	-3.648919
H	3.040955	-1.519411	-3.718437
C	1.258730	-1.378276	-2.462398
H	0.472158	-1.314484	-3.227920
H	1.567431	-0.346362	-2.244416
C	0.703557	-2.036097	-1.182413
H	1.087784	-1.518436	-0.287793
H	-0.389859	-1.932655	-1.143310
C	1.173069	-8.314934	-0.250009
H	1.118102	-8.494504	-1.331902
H	1.765371	-7.397486	-0.133787
C	-0.264636	-10.598927	-0.440632
H	-0.905790	-11.491659	-0.407066
H	0.049105	-10.469992	-1.487160
C	1.859362	-9.502997	0.426111
H	2.783068	-9.719934	-0.129716
H	2.172398	-9.221720	1.444213
C	0.949417	-10.752204	0.473784

H 0.595565 -10.929762 1.501859
H 1.513477 -11.651314 0.190970

85
B$_1$
C 7.468741 -0.428004 -1.221153
C 6.348474 0.377212 -1.039097
C 5.233545 -0.115712 -0.346700
C 5.248799 -1.437147 0.166746
C 6.387201 -2.230833 -0.022794
C 7.487910 -1.727940 -0.711853
H 8.332253 -0.038609 -1.761890
H 6.396701 -3.246709 0.373344
H 8.367871 -2.356855 -0.853948
C 4.090193 -1.931239 0.853051
C 3.055474 -2.274658 1.387664
C -0.399130 -3.125000 1.445963
C -0.715134 -3.283725 2.805591
C 0.270990 -3.124791 3.765529
C 1.546680 -2.795828 3.293354
C 1.768268 -2.647339 1.918764
N 0.795652 -2.826391 1.000401
H 0.063661 -3.245965 4.829113
H 2.372230 -2.646895 3.989511
C 4.088887 0.717788 -0.124104
C 3.116477 1.411094 0.101634
C -0.119783 3.082443 0.454787
C 0.222691 4.184230 1.252838
C 1.526606 4.386273 1.678004
C 2.469429 3.452958 1.264471
C 2.109978 2.345251 0.490749
N 0.800772 2.171490 0.124205
H 1.795815 5.244640 2.293777
H 3.517731 3.559217 1.540345
H 6.325944 1.395244 -1.429352
I 0.226124 0.360636 -0.844522
O -0.822734 4.959454 1.530315
O -2.004472 -3.601929 2.995924
C -1.635288 -3.502327 0.644569
C -2.705518 -3.310446 1.757144
H -2.975055 -2.241274 1.806787
C -1.620410 3.127711 0.200431
C -1.859638 4.633930 0.573696
H -1.651824 5.244287 -0.318536
C -2.004233 2.927553 -1.262531
C -3.118926 2.179616 -1.648336
C -1.235789 3.550999 -2.258593
C -3.467586 2.071586 -2.996830
H -3.726009 1.663863 -0.904105
C -1.570492 3.425846 -3.603750
H -0.357036 4.140621 -1.981707
C -2.696080 2.689345 -3.978043
H -4.347736 1.490493 -3.278743
H -0.952747 3.909447 -4.362191
H -2.967551 2.597024 -5.030972
C -1.848281 -2.655517 -0.597656
C -3.046503 -1.993100 -0.874414
C -0.804053 -2.557945 -1.539992
C -3.210559 -1.261559 -2.057266
H -3.881205 -2.040490 -0.174553
C -0.963366 -1.816875 -2.715975
H 0.137069 -3.075164 -1.351942
C -2.175811 -1.166866 -2.979846
H -4.162016 -0.762766 -2.249458
H -0.146766 -1.772713 -3.440358
H -2.302402 -0.591334 -3.898145
C -1.492304 -5.007049 0.274284

H	-0.825623	-5.089099	-0.596516
H	-0.990696	-5.524004	1.108995
C	-3.958466	-4.162595	1.678339
H	-4.627885	-3.715402	0.927525
H	-4.476067	-4.087529	2.646441
C	-3.683271	-5.614925	1.299938
H	-4.637016	-6.145488	1.167410
H	-3.144474	-6.118091	2.119647
C	-2.837353	-5.681320	0.030924
H	-2.672295	-6.725752	-0.271343
H	-3.367434	-5.183962	-0.799942
C	-2.300154	2.164413	1.216357
H	-2.337258	1.154707	0.781655
H	-1.651838	2.098482	2.105088
C	-3.216553	4.982224	1.138004
H	-3.178246	6.025060	1.485506
H	-3.935705	4.946674	0.303937
C	-3.686119	2.603228	1.693939
H	-4.020550	1.895634	2.466448
H	-4.420035	2.552867	0.874858
C	-3.654006	4.025676	2.239417
H	-4.643860	4.318481	2.616102
H	-2.951609	4.087215	3.086933

85

B_2

C	3.784703	-5.908415	8.704123
C	2.663534	-6.167488	7.923305
C	2.758817	-6.170149	6.522602
C	4.008159	-5.905689	5.905395
C	5.125850	-5.644619	6.709935
C	5.014593	-5.646745	8.097378
H	3.698128	-5.909588	9.791312
H	6.083739	-5.439432	6.230915
H	5.894386	-5.441589	8.708781
C	4.131026	-5.893879	4.475707
C	4.227948	-5.870245	3.265192
C	4.133568	-4.663636	-0.097778
C	3.921399	-5.815769	-0.882330
C	3.887048	-7.060492	-0.278784
C	4.043603	-7.071756	1.117594
C	4.205027	-5.862614	1.823288
N	4.269113	-4.664213	1.206187
H	3.750664	-7.979773	-0.849303
H	4.069750	-8.017411	1.662555
C	1.587534	-6.436079	5.743014
C	0.581238	-6.664213	5.099247
C	-1.798035	-7.312793	2.403317
C	-2.994263	-7.504135	3.109722
C	-3.027533	-7.414155	4.493729
C	-1.828121	-7.124547	5.135545
C	-0.639859	-6.947798	4.419635
N	-0.659480	-7.062735	3.051390
H	-3.954002	-7.562651	5.048816
H	-1.785770	-7.028650	6.219735
H	1.698756	-6.370650	8.389355
I	1.193866	-6.927888	2.028172
O	-4.017122	-7.793071	2.307998
O	3.789644	-5.532135	-2.182901
C	4.267178	-3.485212	-1.044591
C	3.546041	-4.100544	-2.282188
H	2.459692	-3.969008	-2.154781
C	-2.067812	-7.599442	0.934294
C	-3.623534	-7.428540	0.960125
H	-3.860648	-6.358906	0.854309
C	-1.447229	-6.588294	-0.023743
C	-0.685296	-6.970400	-1.132597

C	-1.625683	-5.219555	0.230354
C	-0.118547	-6.007635	-1.969470
H	-0.509806	-8.023640	-1.351690
C	-1.062732	-4.257956	-0.604778
H	-2.204839	-4.895727	1.099973
C	-0.308082	-4.650401	-1.711419
H	0.476012	-6.323817	-2.828627
H	-1.214187	-3.198609	-0.390590
H	0.126734	-3.897842	-2.372095
C	3.557718	-2.237141	-0.539664
C	4.170628	-0.981149	-0.496328
C	2.234012	-2.346036	-0.083145
C	3.476834	0.137666	-0.027543
H	5.202867	-0.858517	-0.823536
C	1.542214	-1.233722	0.388459
H	1.740436	-3.322072	-0.085373
C	2.161579	0.017829	0.413367
H	3.976081	1.107925	-0.005331
H	0.517376	-1.344987	0.747652
H	1.622384	0.891910	0.782441
C	5.783061	-3.311404	-1.321517
H	6.228934	-2.742628	-0.492603
H	6.248583	-4.310060	-1.291668
C	3.975677	-3.590717	-3.639051
H	3.557233	-2.576898	-3.749427
H	3.503107	-4.218858	-4.409074
C	5.491025	-3.539828	-3.800127
H	5.745985	-3.126122	-4.786188
H	5.901156	-4.562527	-3.760854
C	6.106221	-2.699586	-2.684723
H	7.198778	-2.643368	-2.799462
H	5.722364	-1.669370	-2.756729
C	-1.673000	-9.083109	0.690978
H	-0.590924	-9.143049	0.504159
H	-1.858080	-9.643154	1.621771
C	-4.403531	-8.230132	-0.055751
H	-5.472131	-8.130793	0.184707
H	-4.247940	-7.746088	-1.033366
C	-2.472118	-9.764258	-0.420449
H	-2.141401	-10.810312	-0.494007
H	-2.269417	-9.292328	-1.394723
C	-3.966476	-9.687830	-0.127666
H	-4.541759	-10.208747	-0.905621
H	-4.183945	-10.190501	0.829054

85

B_3

C	3.943017	-6.162634	8.679653
C	2.783852	-6.249581	7.915287
C	2.867888	-6.391000	6.522425
C	4.137671	-6.446470	5.893454
C	5.292749	-6.360023	6.681112
C	5.194441	-6.219052	8.063043
H	3.869695	-6.050564	9.762073
H	6.268160	-6.402793	6.195714
H	6.102881	-6.152311	8.663292
C	4.222896	-6.576428	4.466594
C	4.247765	-6.654948	3.254928
C	4.087604	-5.391695	-0.070410
C	3.995756	-6.527751	-0.893493
C	4.036244	-7.789526	-0.322840
C	4.157321	-7.833614	1.072389
C	4.212215	-6.647317	1.813324
N	4.177263	-5.424216	1.237257
H	3.975240	-8.697735	-0.923127
H	4.191257	-8.790780	1.592910
C	1.678460	-6.506112	5.734561

C	0.688566	-6.630831	5.039965
C	-1.661188	-6.922114	2.251800
C	-2.654510	-7.739607	2.811515
C	-2.600168	-8.131847	4.141059
C	-1.494271	-7.717970	4.872360
C	-0.482489	-6.946122	4.292910
N	-0.603401	-6.553361	2.982859
H	-3.381731	-8.752158	4.580268
H	-1.376039	-8.005103	5.916367
H	1.801752	-6.208558	8.387417
I	0.934921	-5.383876	2.135784
O	-3.584576	-8.094230	1.929965
O	3.881522	-6.204916	-2.191769
C	4.176702	-4.179510	-0.978025
C	3.517782	-4.796283	-2.245383
H	2.421078	-4.763262	-2.138891
C	-1.941429	-6.801783	0.759507
C	-3.450979	-7.221343	0.782314
H	-4.056215	-6.330137	1.006677
C	-1.832139	-5.397346	0.175668
C	-2.461019	-4.333987	0.840347
C	-1.162188	-5.134077	-1.023313
C	-2.438449	-3.045699	0.312035
H	-2.977364	-4.513999	1.787014
C	-1.144937	-3.843825	-1.557559
H	-0.642969	-5.930584	-1.556558
C	-1.786959	-2.797899	-0.897476
H	-2.932570	-2.233087	0.847060
H	-0.621366	-3.658624	-2.497393
H	-1.769851	-1.790524	-1.316725
C	3.432086	-2.982653	-0.407117
C	2.060086	-3.094167	-0.125544
C	4.074214	-1.787690	-0.067744
C	1.362352	-2.061557	0.500708
H	1.517664	-3.999928	-0.412746
C	3.373936	-0.744254	0.544187
H	5.136519	-1.654013	-0.270143
C	2.020287	-0.878015	0.839864
H	0.300298	-2.185550	0.720462
H	3.901635	0.176633	0.798671
H	1.478773	-0.067539	1.329999
C	5.684228	-3.939321	-1.238314
H	6.126543	-3.450064	-0.359538
H	6.174190	-4.923443	-1.315236
C	3.914276	-4.189891	-3.573650
H	3.397744	-3.218429	-3.643324
H	3.512044	-4.824442	-4.377626
C	5.417969	-3.979386	-3.720502
H	5.626953	-3.464089	-4.668945
H	5.925481	-4.957060	-3.764253
C	5.959811	-3.182904	-2.536095
H	7.042865	-3.022022	-2.640913
H	5.485463	-2.187681	-2.513345
C	-1.062317	-7.883547	0.065725
H	-0.060307	-7.468231	-0.118979
H	-0.925418	-8.718187	0.771918
C	-3.975182	-7.903821	-0.459580
H	-4.990293	-8.266090	-0.240902
H	-4.070475	-7.130039	-1.238241
C	-1.667455	-8.464273	-1.213806
H	-1.730197	-7.699387	-2.003042
H	-0.995979	-9.252725	-1.583414
C	-3.061588	-9.020491	-0.948319
H	-3.479258	-9.470073	-1.859799
H	-3.007635	-9.817058	-0.188067

B_4

chir-op-conf53-optall.out

C	3.682681	-4.662050	8.782761
C	3.983860	-4.359003	7.458265
C	2.988806	-4.442763	6.474683
C	1.672873	-4.836900	6.831238
C	1.389691	-5.135375	8.169759
C	2.388455	-5.047993	9.136638
H	4.461899	-4.595426	9.543100
H	0.377965	-5.437151	8.442618
H	2.154128	-5.283296	10.175769
C	0.669115	-4.927919	5.809013
C	-0.099442	-5.000360	4.871265
C	-1.325362	-6.394859	1.834907
C	-2.202172	-5.428002	1.316967
C	-2.454497	-4.269540	2.035037
C	-1.772039	-4.131692	3.248836
C	-0.900652	-5.140149	3.679978
N	-0.703863	-6.277251	2.982266
H	-3.134560	-3.501070	1.666293
H	-1.901561	-3.236853	3.857791
C	3.273813	-4.107194	5.111450
C	3.446689	-3.815355	3.944377
C	3.204026	-3.526251	0.308448
C	3.700488	-2.235107	0.097460
C	4.178592	-1.463251	1.149682
C	4.103711	-2.017953	2.418622
C	3.592935	-3.305352	2.620476
N	3.184390	-4.043931	1.543070
H	4.580999	-0.464498	0.979618
H	4.431914	-1.458765	3.293835
H	4.991145	-4.054309	7.172059
I	2.539882	-6.043898	1.839832
O	3.696196	-1.874681	-1.178348
O	-2.707556	-5.781664	0.125400
C	-1.334939	-7.584881	0.885179
C	-1.883943	-6.865070	-0.382588
H	-1.038226	-6.377829	-0.899860
C	2.857554	-4.149432	-1.039591
C	2.991931	-2.870952	-1.961697
H	3.624389	-3.088592	-2.830930
C	3.923776	-5.190819	-1.399709
C	5.275273	-4.928700	-1.128952
C	3.588845	-6.391556	-2.032654
C	6.264405	-5.845009	-1.478715
H	5.572821	-3.999304	-0.636806
C	4.581156	-7.307966	-2.386652
H	2.548504	-6.628992	-2.254258
C	5.920078	-7.040979	-2.109668
H	7.309165	-5.622119	-1.256159
H	4.301413	-8.238825	-2.883645
H	6.693213	-7.760119	-2.384652
C	0.032281	-8.223301	0.692651
C	0.773383	-8.612928	1.828496
C	0.595085	-8.451834	-0.565317
C	2.046384	-9.178113	1.698605
H	0.349207	-8.465321	2.821707
C	1.859275	-9.041227	-0.695718
H	0.057192	-8.175436	-1.472960
C	2.595358	-9.394561	0.428460
H	2.598989	-9.471170	2.593980
H	2.267670	-9.215336	-1.692545
H	3.586282	-9.839216	0.324592
C	-2.363489	-8.612299	1.437440
H	-1.878651	-9.195566	2.234102
H	-3.191819	-8.057752	1.908437
C	-2.694065	-7.691841	-1.363221
H	-1.991909	-8.271222	-1.982073

H	-3.211582	-6.995617	-2.040034
C	-3.670237	-8.650609	-0.688014
H	-4.152328	-9.278672	-1.450736
H	-4.469099	-8.079096	-0.187372
C	-2.942596	-9.505759	0.346566
H	-3.629358	-10.235507	0.799494
H	-2.136595	-10.080210	-0.142391
C	1.411289	-4.668241	-1.163748
H	1.245310	-4.875335	-2.231224
H	1.287061	-5.629442	-0.650763
C	1.655967	-2.279982	-2.390237
H	1.852145	-1.274825	-2.790142
H	1.254322	-2.879319	-3.219753
C	0.385972	-3.647283	-0.669252
H	-0.615009	-3.993059	-0.969038
H	0.384513	-3.627832	0.433913
C	0.666796	-2.232701	-1.226932
H	-0.266379	-1.754072	-1.552395
H	1.078973	-1.584607	-0.436987

85

C_1

C	5.212534	-6.749286	7.765524
C	5.122651	-6.309262	6.449065
C	3.884267	-6.311540	5.791795
C	2.721128	-6.766097	6.467631
C	2.833839	-7.202073	7.794628
C	4.069782	-7.192371	8.435795
H	6.177904	-6.745463	8.272899
H	1.941713	-7.550529	8.315971
H	4.142199	-7.535775	9.468706
C	1.457843	-6.773298	5.788470
C	0.408272	-6.769737	5.175279
C	-1.919616	-7.185024	2.491992
C	-3.148469	-6.761342	3.032240
C	-3.217911	-6.331647	4.346669
C	-2.017497	-6.344860	5.062585
C	-0.833464	-6.766789	4.443563
N	-0.789824	-7.191154	3.159717
H	-4.155106	-6.002849	4.796380
H	-1.990497	-6.020958	6.103012
C	3.750844	-5.844398	4.449005
C	3.549605	-5.443918	3.319125
C	3.440888	-3.887009	0.006436
C	2.108313	-4.119194	-0.360855
C	1.244094	-4.815069	0.471695
C	1.757377	-5.258504	1.683776
C	3.085028	-5.014991	2.046479
N	3.907137	-4.343726	1.174480
H	0.210794	-5.014146	0.182476
H	1.132118	-5.822499	2.378907
H	6.007117	-5.957428	5.916734
I	5.919940	-4.114582	1.702897
O	1.823124	-3.637580	-1.569534
O	-4.154944	-6.851571	2.151555
C	-2.220033	-7.754101	1.110065
C	-3.556422	-7.010620	0.837850
H	-3.333843	-5.989807	0.484284
C	4.144381	-3.237447	-1.176088
C	2.873938	-2.700282	-1.914286
H	2.585739	-1.738318	-1.462956
C	5.067783	-2.083538	-0.803122
C	6.381295	-1.992341	-1.274854
C	4.599081	-1.094039	0.074743
C	7.207300	-0.942188	-0.873114
H	6.787180	-2.750923	-1.943847
C	5.425456	-0.049206	0.481375

H	3.579959	-1.148174	0.467495
C	6.735611	0.029494	0.008341
H	8.230200	-0.889648	-1.249494
H	5.044859	0.702311	1.174896
H	7.386670	0.845289	0.326644
C	-1.137634	-7.525102	0.067139
C	0.133343	-8.086305	0.274856
C	-1.354269	-6.801932	-1.113655
C	1.147485	-7.937670	-0.669164
H	0.331628	-8.641936	1.192638
C	-0.337899	-6.652659	-2.061513
H	-2.322813	-6.345005	-1.317734
C	0.915096	-7.222365	-1.845611
H	2.125961	-8.385100	-0.483238
H	-0.534205	-6.088415	-2.975038
H	1.704931	-7.114914	-2.591497
C	-2.495091	-9.273436	1.309182
H	-1.533328	-9.795592	1.408890
H	-3.028788	-9.409936	2.264255
C	-4.545660	-7.680794	-0.095558
H	-4.187024	-7.532351	-1.125949
H	-5.504058	-7.146446	-0.012956
C	-4.711607	-9.174665	0.167202
H	-5.361617	-9.612676	-0.603809
H	-5.212132	-9.328069	1.137388
C	-3.349838	-9.863611	0.193191
H	-3.465915	-10.945202	0.355484
H	-2.844523	-9.734752	-0.779675
C	4.805243	-4.391419	-1.981130
H	5.778355	-4.635084	-1.529948
H	4.176961	-5.289474	-1.865941
C	2.983625	-2.557255	-3.414299
H	1.984570	-2.313268	-3.804268
H	3.626834	-1.683792	-3.608083
C	4.934889	-4.109960	-3.478876
H	5.380374	-4.992762	-3.959744
H	5.616662	-3.264738	-3.661326
C	3.572565	-3.791870	-4.085330
H	3.660748	-3.619168	-5.166922
H	2.893509	-4.649281	-3.947597

85

C_2

C	4.487742	-5.365647	8.518111
C	4.784629	-5.225685	7.166357
C	3.781105	-5.401124	6.203038
C	2.461621	-5.733977	6.604765
C	2.182866	-5.863562	7.973179
C	3.187132	-5.678966	8.919132
H	5.273007	-5.226530	9.262092
H	1.169306	-6.118658	8.284313
H	2.953402	-5.785263	9.979442
C	1.438119	-5.965442	5.627448
C	0.586233	-6.208191	4.794914
C	-0.916065	-7.469138	1.808111
C	-2.299207	-7.356252	2.039108
C	-2.759929	-6.792476	3.217328
C	-1.781314	-6.384872	4.128761
C	-0.422974	-6.554641	3.826607
N	0.004617	-7.093204	2.663130
H	-3.824625	-6.682781	3.425316
H	-2.065538	-5.942022	5.083537
C	4.060602	-5.211386	4.812923
C	4.213317	-4.999866	3.626553
C	3.442897	-3.646859	0.331668
C	4.443448	-4.223123	-0.466585
C	5.422823	-5.033751	0.086033

C	5.337842	-5.282267	1.452647
C	4.326512	-4.723834	2.235607
N	3.407990	-3.884284	1.644881
H	6.210049	-5.471502	-0.528467
H	6.055355	-5.935842	1.947395
H	5.794413	-4.972163	6.841870
I	1.987583	-2.944717	2.874335
O	4.334501	-3.872815	-1.745599
O	-3.032155	-7.855794	1.033359
C	-0.740820	-8.235397	0.502843
C	-2.149086	-7.997172	-0.110679
H	-2.159392	-7.018346	-0.617581
C	2.634864	-2.699630	-0.542557
C	3.006857	-3.314578	-1.930265
H	2.340847	-4.170070	-2.122097
C	1.133627	-2.788086	-0.307434
C	0.329664	-1.655679	-0.147239
C	0.547409	-4.056683	-0.185769
C	-1.027964	-1.787891	0.146134
H	0.757434	-0.656016	-0.220158
C	-0.805030	-4.187235	0.118270
H	1.159373	-4.958447	-0.294513
C	-1.598305	-3.052305	0.289255
H	-1.639530	-0.893214	0.273984
H	-1.234212	-5.183718	0.233424
H	-2.656843	-3.153937	0.534157
C	0.413831	-7.747025	-0.358413
C	1.713106	-7.782889	0.174813
C	0.250606	-7.277461	-1.667851
C	2.809649	-7.364951	-0.576072
H	1.862886	-8.135364	1.195650
C	1.350388	-6.858816	-2.422541
H	-0.738287	-7.229533	-2.125244
C	2.634060	-6.899145	-1.881546
H	3.809197	-7.414506	-0.139852
H	1.196243	-6.499998	-3.442004
H	3.492925	-6.572268	-2.471963
C	-0.566261	-9.732180	0.892045
H	0.473000	-9.891576	1.213061
H	-1.202954	-9.940468	1.767941
C	-2.687108	-9.059967	-1.049639
H	-2.189569	-8.924668	-2.022650
H	-3.757324	-8.859819	-1.209120
C	-2.445875	-10.485100	-0.562621
H	-2.776580	-11.196697	-1.332751
H	-3.050637	-10.679238	0.338554
C	-0.970113	-10.683939	-0.227778
H	-0.779093	-11.720274	0.087084
H	-0.353796	-10.498936	-1.124735
C	3.259810	-1.286339	-0.365746
H	2.823054	-0.809910	0.524273
H	4.334786	-1.410185	-0.157734
C	2.996462	-2.367587	-3.106974
H	3.415388	-2.900576	-3.973090
H	1.940704	-2.154825	-3.339762
C	3.133826	-0.388235	-1.597836
H	3.646502	0.561032	-1.385477
H	2.079821	-0.145164	-1.803674
C	3.736479	-1.066491	-2.823183
H	3.677389	-0.406476	-3.699725
H	4.803981	-1.276895	-2.645814

85

C_3

C	0.148228	7.567620	-0.878259
C	0.865821	6.551690	-0.252256
C	0.493951	5.212533	-0.424405

C	-0.627308	4.903538	-1.237580
C	-1.335702	5.938006	-1.867106
C	-0.948059	7.261947	-1.687193
H	-1.505613	8.059869	-2.179130
H	-2.194721	5.690703	-2.491775
C	-1.052638	3.545266	-1.402625
C	-1.436650	2.396363	-1.512414
C	-1.971722	1.090305	-1.710178
C	-2.640222	0.773824	-2.896616
C	-3.183265	-0.487850	-3.113339
C	-3.048507	-1.412893	-2.088982
C	-2.391444	-1.082862	-0.891947
N	-1.862726	0.132597	-0.727750
I	-0.737957	0.612398	0.982685
N	2.792410	1.113003	0.010256
C	3.173394	-0.106064	0.316744
C	3.206527	-0.610494	1.625574
C	2.762164	0.176043	2.680259
C	2.330328	1.466488	2.353196
C	2.364434	1.899614	1.017761
C	1.806703	3.166434	0.617837
C	1.232848	4.142913	0.179743
H	1.972381	2.144485	3.129345
H	2.763976	-0.187372	3.708255
O	3.707005	-1.845610	1.695021
C	4.208183	-2.231904	0.381772
C	5.727217	-2.278089	0.431579
C	6.327602	-0.875692	0.482085
H	5.965635	-0.375696	1.394877
H	7.419188	-0.942588	0.586795
C	5.953411	-0.044623	-0.764830
C	4.782840	-0.646123	-1.550538
H	5.123865	-1.482097	-2.180380
H	4.350054	0.105474	-2.227085
C	3.655596	-1.179204	-0.640812
C	2.520932	-1.711855	-1.527077
C	2.523762	-3.037420	-1.981565
C	1.528958	-3.507297	-2.841546
C	0.518168	-2.652735	-3.281828
C	0.522020	-1.322424	-2.860719
C	1.510105	-0.857197	-1.991617
H	1.506381	0.190065	-1.684887
H	-0.245154	-0.633378	-3.220416
H	-0.257912	-3.016792	-3.958272
H	1.553069	-4.546415	-3.174572
H	3.316164	-3.723694	-1.676506
H	6.814685	0.036925	-1.443472
H	5.697659	0.981903	-0.463619
H	6.023693	-2.871761	1.308486
H	6.082991	-2.818222	-0.459807
H	3.811701	-3.236824	0.200348
C	-2.656717	-2.217561	0.091925
C	-1.498978	-2.601813	1.002399
C	-1.613423	-2.599816	2.397365
C	-0.506413	-2.867027	3.202991
C	0.731196	-3.150333	2.627007
C	0.846578	-3.184211	1.237404
C	-0.255962	-2.911764	0.432267
H	-0.128269	-2.904435	-0.653373
H	1.804173	-3.414209	0.771681
H	1.604511	-3.344780	3.251993
H	-0.615429	-2.846267	4.288565
H	-2.559898	-2.357922	2.879726
C	-3.963019	-1.810944	0.832602
C	-4.766646	-3.004874	1.348980
C	-5.130221	-3.936282	0.197194
C	-3.864508	-4.453038	-0.477029

C	-2.953112	-3.342704	-0.946411
H	-1.999283	-3.758620	-1.306922
O	-3.544466	-2.646879	-2.074771
H	-4.093050	-5.092788	-1.341960
H	-3.284935	-5.065852	0.232014
H	-5.729025	-4.784005	0.558161
H	-5.746524	-3.392961	-0.537732
H	-5.672303	-2.627024	1.845098
H	-4.195345	-3.566942	2.104015
H	-3.714332	-1.101025	1.634933
H	-4.604719	-1.262173	0.125175
H	-3.701502	-0.738832	-4.039128
H	-2.712631	1.550995	-3.656670
H	1.728243	6.785266	0.372806
H	0.449858	8.606384	-0.736818

85

C_4

C	7.017485	0.070600	-0.312053
C	5.692915	0.460119	-0.477259
C	4.653572	-0.395960	-0.083633
C	4.952991	-1.661642	0.483978
C	6.295567	-2.034297	0.642476
C	7.316214	-1.174634	0.247339
H	7.821522	0.739984	-0.620087
H	6.527157	-3.006095	1.079651
H	8.355810	-1.478856	0.377003
C	3.891331	-2.533380	0.887181
C	2.964617	-3.245559	1.221310
C	-0.374225	-4.428956	1.726767
C	-0.209395	-5.657398	2.382159
C	1.065520	-6.117870	2.682896
C	2.123579	-5.298537	2.291957
C	1.871325	-4.088618	1.625379
N	0.622903	-3.664416	1.337485
H	1.227484	-7.069323	3.189958
H	3.154073	-5.590272	2.495151
C	3.289420	-0.005313	-0.243515
C	2.121940	0.309519	-0.366598
C	-1.046528	2.042318	-0.942020
C	-1.973710	1.064158	-0.558721
C	-1.562141	-0.187815	-0.127315
C	-0.192478	-0.419172	-0.066242
C	0.726329	0.562181	-0.451713
N	0.263713	1.774356	-0.908315
H	-2.286407	-0.950556	0.162836
H	0.196484	-1.375114	0.296764
H	5.446764	1.429748	-0.912091
I	1.661297	3.165934	-1.611744
O	-3.233067	1.477999	-0.686000
O	-1.363217	-6.285764	2.622328
C	-1.848921	-4.215454	1.446032
C	-2.465533	-5.414443	2.253011
H	-2.900843	-5.044680	3.193127
C	-1.821843	3.234323	-1.484999
C	-3.197748	2.921965	-0.806770
H	-3.177202	3.313570	0.222195
C	-1.298226	4.586662	-1.015639
C	-1.056219	5.645493	-1.896627
C	-1.012796	4.766350	0.346137
C	-0.535353	6.851824	-1.427422
H	-1.249399	5.538042	-2.963852
C	-0.486413	5.967927	0.814109
H	-1.184223	3.949115	1.052004
C	-0.244606	7.016571	-0.073892
H	-0.350753	7.666012	-2.130145
H	-0.259895	6.082361	1.875345

H	0.169739	7.958788	0.288302
C	-2.399297	-2.885528	1.950702
C	-3.723841	-2.526644	1.651311
C	-1.641787	-2.010228	2.739913
C	-4.263017	-1.321489	2.099798
H	-4.350155	-3.194181	1.056565
C	-2.179936	-0.801449	3.187655
H	-0.613803	-2.258913	3.003212
C	-3.488192	-0.446845	2.863294
H	-5.293739	-1.065177	1.848446
H	-1.564877	-0.133382	3.793540
H	-3.905837	0.500201	3.209526
C	-2.083082	-4.398935	-0.074643
H	-3.105157	-4.086543	-0.333837
H	-1.392573	-3.728061	-0.609603
C	-3.481145	-6.211181	1.462653
H	-4.308579	-5.523038	1.230033
H	-3.900225	-7.003651	2.099122
C	-2.886915	-6.800009	0.176242
H	-3.711163	-7.028927	-0.514701
H	-2.397136	-7.757024	0.404690
C	-1.874027	-5.851493	-0.498909
H	-0.844545	-6.153386	-0.246375
H	-1.951063	-5.929297	-1.592266
C	-1.903881	3.049359	-3.026544
H	-0.987583	3.447896	-3.486332
H	-1.915622	1.968852	-3.242504
C	-4.427638	3.420584	-1.528507
H	-5.309239	3.003563	-1.019994
H	-4.462721	4.512998	-1.388965
C	-3.156375	3.657174	-3.660618
H	-3.137586	3.440090	-4.738350
H	-3.160128	4.752991	-3.552963
C	-4.415611	3.088918	-3.015528
H	-5.315348	3.499667	-3.494273
H	-4.443912	1.995587	-3.153630

NCI analyses

Optimized geometries obtained at the ωB97X-D/aug-cc-pVTZ level of theory

4-I

I	-0.87000200	-0.00003400	0.00000600
N	-0.95019000	2.27781100	0.09227100
N	-0.95031600	-2.27773500	-0.09233600
C	0.13082000	3.03504800	0.18501700
C	-2.14770800	2.86351500	-0.17619900
C	0.13067100	-3.03502000	-0.18509500
C	-2.14787900	-2.86339000	0.17605400
C	1.58828900	2.66560800	0.41865400
C	0.08112400	4.41060100	-0.06178800
C	-2.23013500	4.22512200	-0.43573500
C	-3.32395200	2.06281100	-0.17143700
C	1.58817700	-2.66564400	-0.41859700
C	0.08087900	-4.41058500	0.06161400
C	-2.23038600	-4.22501600	0.43547300
C	-3.32406600	-2.06261300	0.17137500
C	1.92129500	1.73215400	1.60410000
C	2.00530500	4.07735200	0.90321500
C	-1.10099900	5.02629700	-0.40121600
O	1.27945800	5.00621400	0.07062700
H	-3.19693300	4.64812500	-0.66018100
C	-4.34731200	1.43334000	-0.13609500
C	1.92132300	-1.73213200	-1.60396400
C	2.00514400	-4.07738700	-0.90323000

C	-1.10129700	-5.02624600	0.40092700
O	1.27918400	-5.00626000	-0.07075900
H	-3.19721500	-4.64798000	0.65985400
C	-4.34739700	-1.43309500	0.13601900
C	3.33459600	1.95286600	2.19096800
H	1.82603100	0.68621700	1.31751100
H	1.17836900	1.91658200	2.38217400
C	3.47555000	4.28850900	1.07875400
H	1.54745100	4.18882800	1.89377700
H	-1.15047100	6.08406200	-0.61134100
C	-5.56626300	0.70033500	-0.07427800
C	3.33463800	-1.95291200	-2.19078200
H	1.82613900	-0.68621000	-1.31729800
H	1.17841800	-1.91643600	-2.38208600
C	3.47538900	-4.28863000	-1.07869000
H	1.54735100	-4.18876400	-1.89382900
H	-1.15083000	-6.08402300	0.61097600
C	-5.56630600	-0.70002800	0.07414700
C	3.84012900	3.40333700	2.28456700
H	3.33943600	1.51998800	3.19096000
H	4.04953000	1.35501100	1.63222400
H	3.68328600	5.33240100	1.30992700
H	4.03410800	4.02579600	0.18178100
C	-6.78216900	1.37986100	-0.14968900
C	3.84010000	-3.40341000	-2.28442800
H	3.33955000	-1.51997800	-3.19075000
H	4.04958100	-1.35513000	-1.63197100
H	3.68307800	-5.33252200	-1.30989800
H	4.03390500	-4.02599400	-0.18166900
C	-6.78225100	-1.37949400	0.14946300
H	4.91965800	3.39322400	2.42795800
H	3.41354900	3.87309400	3.17345200
C	-7.97816200	0.69019900	-0.07586100
H	-6.77644700	2.45417700	-0.26417700
H	4.91964000	-3.39334200	-2.42773400
H	3.41356700	-3.87309600	-3.17337300
C	-7.97820400	-0.68976900	0.07556200
H	-6.77658900	-2.45381100	0.26394000
H	-8.91226300	1.23008900	-0.13514800
H	-8.91233600	-1.22961200	0.13478000
C	2.20113100	-2.24162300	0.92936900
C	3.51315600	-1.77448900	1.02788600
C	1.46483300	-2.30566600	2.11297300
C	4.06043000	-1.39955400	2.24326000
H	4.13768500	-1.70235300	0.15607000
C	2.00485500	-1.91869800	3.33028800
H	0.44336100	-2.65492600	2.11409200
C	3.30916900	-1.46486400	3.40451200
H	5.08192600	-1.04640700	2.27539200
H	1.39544200	-1.97767700	4.22146100
H	3.73401900	-1.16320100	4.35170000
C	2.20134500	2.24148200	-0.92923400
C	3.51352000	1.77475100	-1.02767900
C	1.46501900	2.30506700	-2.11284800
C	4.06089600	1.39974600	-2.24298500
H	4.13809000	1.70299900	-0.15586000
C	2.00514600	1.91802300	-3.33009300
H	0.44344100	2.65401300	-2.11403100
C	3.30959900	1.46458200	-3.40424200
H	5.08250600	1.04692200	-2.27505900
H	1.39570500	1.97664000	-4.22127000
H	3.73453700	1.16287400	-4.35137600

4-Ag

Ag	0.88995900	-0.00002300	-0.00031500
N	0.94182200	-2.16888500	0.17175400
N	0.94219800	2.16884700	-0.17229400

C	-0.14077500	-2.92566000	0.29756600
C	2.12899800	-2.81086500	-0.01857000
C	-0.14033700	2.92573900	-0.29798400
C	2.12949400	2.81070300	0.01762600
C	-1.60001700	-2.52268900	0.42882000
C	-0.10731800	-4.32854400	0.22567300
C	2.21184600	-4.19948200	-0.14302800
C	3.31995000	-2.02578600	-0.07479100
C	-1.59967300	2.52292600	-0.42865800
C	-0.10667400	4.32862600	-0.22636000
C	2.21258300	4.19934700	0.14168000
C	3.32032100	2.02544100	0.07396700
C	-1.94612400	-1.39572100	1.41803800
C	-2.03937500	-3.83365300	1.12830800
C	1.07454600	-4.99650800	-0.02711900
O	-1.31925400	-4.88896800	0.44269700
H	3.18588700	-4.65048900	-0.31544200
C	4.37478000	-1.42628100	-0.05337200
C	-1.94626000	1.39582200	-1.41757000
C	-2.03910900	3.83382000	-1.12820600
C	1.07536500	4.99649800	0.02588400
O	-1.31861600	4.88916800	-0.44305100
H	3.18673800	4.65025200	0.31372200
C	4.37503100	1.42571400	0.05291400
C	-3.41805500	-1.45767900	1.87228000
H	-1.74653200	-0.40919800	0.98724600
H	-1.28699600	-1.49919300	2.29140500
C	-3.51268500	-4.01950600	1.34434800
H	-1.57749100	-3.79033700	2.13067100
H	1.12210600	-6.07994700	-0.10901200
C	5.60791100	-0.70669900	-0.03355700
C	-3.41829100	1.45794500	-1.87150800
H	-1.74679200	0.40935800	-0.98659400
H	-1.28731500	1.49895800	-2.29111100
C	-3.51247100	4.01982800	-1.34376300
H	-1.57759300	3.79027800	-2.13073000
H	1.12308500	6.07995100	0.10748900
C	5.60803400	0.70590400	0.03360100
C	-3.92665000	-2.85760400	2.27899900
H	-3.52728800	-0.78795500	2.73232200
H	-4.05445200	-1.02529600	1.09269400
H	-3.69478100	-4.97587300	1.84950600
H	-4.06896200	-4.03674000	0.40265800
C	6.82841800	-1.39576000	-0.06746800
C	-3.92686200	2.85789300	-2.27818300
H	-3.52776400	0.78818800	-2.73149200
H	-4.05456700	1.02565400	-1.09177400
H	-3.69462000	4.97617700	-1.84894100
H	-4.06842900	4.03721500	-0.40188600
C	6.82865400	1.39474100	0.06800000
H	-5.01916100	-2.82948800	2.37348400
H	-3.53324700	-3.08955500	3.27972800
C	8.03071600	-0.69809600	-0.03409300
H	6.82260400	-2.48319900	-0.11837800
H	-5.01940500	2.82988100	-2.37232000
H	-3.53375500	3.08972700	-3.27905400
C	8.03083500	0.69685200	0.03510400
H	6.82301800	2.48218100	0.11890500
H	8.97256400	-1.24424100	-0.06086200
H	8.97277600	1.24281800	0.06225600
C	-2.16290600	2.30652100	0.99530600
C	-3.53585000	2.16134200	1.25338200
C	-1.30122800	2.22111200	2.09976000
C	-4.01958100	1.96421900	2.54443000
H	-4.26356300	2.20500100	0.45368300
C	-1.77990000	2.00799200	3.39208300
H	-0.22632400	2.32002700	1.97923100

C	-3.14577200	1.88513000	3.62574700
H	-5.09328700	1.86414800	2.69935200
H	-1.07298700	1.94771100	4.21884800
H	-3.52462900	1.72677600	4.63464200
C	-2.16379500	-2.30610800	-0.99489400
C	-3.53683400	-2.16086500	-1.25240700
C	-1.30256500	-2.22072200	-2.09970000
C	-4.02109300	-1.96377900	-2.54326600
H	-4.26422300	-2.20445000	-0.45240200
C	-1.78175800	-2.00760500	-3.39182800
H	-0.22760800	-2.31963200	-1.97960600
C	-3.14772700	-1.88475100	-3.62494400
H	-5.09486000	-1.86368600	-2.69774500
H	-1.07517500	-1.94733300	-4.21887500
H	-3.52699500	-1.72646000	-4.63369400

6-I

I	-0.88174000	0.12497300	-0.30078500
N	-0.63941200	-2.13328000	-0.54665500
N	-1.18452000	2.35668000	-0.03781300
C	-5.34679300	-1.21334200	0.22022100
C	-6.45278000	-2.05180000	0.36078300
C	-7.72471800	-1.52404400	0.48455000
C	-7.91212800	-0.14882100	0.46933900
C	-6.82707700	0.69723900	0.33340600
C	-5.53649100	0.18235500	0.20932200
C	-4.05103400	-1.78777900	0.08048200
C	-2.96896500	-2.29449200	-0.05084500
C	-1.70367200	-2.92305000	-0.24076700
C	-4.43791700	1.08063300	0.08818800
C	-3.52315800	1.85609000	0.00492700
C	-2.46755700	2.81304000	-0.04217000
C	0.56421400	-2.68067900	-0.81126400
C	0.75309500	-4.06816400	-0.66040100
C	-0.31413900	-4.85267700	-0.32027600
C	-1.57440500	-4.28739800	-0.12601000
C	-0.14770700	3.22183100	-0.00300700
C	-0.39988100	4.60906100	-0.05523100
C	-1.68971600	5.05863000	-0.09217900
C	-2.75308400	4.15734700	-0.06614400
H	-6.30125600	-3.12146900	0.36959300
H	-8.57143700	-2.18667100	0.59204800
H	-6.96684100	1.76851700	0.32404000
H	-8.90549900	0.26545400	0.56487900
H	1.72885200	-4.49733100	-0.81927700
H	-2.43555900	-4.88771700	0.11847400
H	0.42264000	5.30447900	-0.06515800
H	-0.18089200	-5.91922300	-0.20964700
H	-1.88490400	6.12063500	-0.13193600
H	-3.77936900	4.48642400	-0.06629200
N	1.54722700	-1.86053700	-1.22455300
H	1.32193600	-0.88366700	-1.30989500
N	1.09930000	2.72297600	0.06589700
H	1.19691900	1.72555400	0.15937900
C	2.95590900	-2.19194500	-1.31753100
H	3.05644000	-3.13129100	-1.86500700
C	2.31463900	3.51577700	0.26245400
H	2.30444900	4.32291800	-0.47084600
C	3.62717100	-1.09661400	-2.14219600
H	4.69232900	-1.29293900	-2.23516900
H	3.18946100	-1.05227500	-3.13846100
H	3.51181800	-0.12709300	-1.65435600
C	2.38465200	4.11676500	1.66743500
H	3.28812000	4.71662500	1.76483100
H	1.52585100	4.75431500	1.87115500
H	2.40869900	3.33120100	2.42142000
C	3.61709700	-2.35472900	0.04269100

C	3.01944100	-1.91634000	1.21593000
C	4.88427100	-2.92553500	0.10863500
C	3.67758200	-2.03533900	2.43225300
H	2.03599400	-1.46705300	1.19059700
C	5.54688300	-3.04022400	1.31917400
H	5.36001600	-3.28011600	-0.79758600
C	4.94449800	-2.59297600	2.48789300
H	3.19798500	-1.68646300	3.33656100
H	6.53307800	-3.48252600	1.35238000
H	5.45933600	-2.68349400	3.43425000
C	3.52608400	2.65493700	-0.03288400
C	3.85858700	1.57816300	0.78564400
C	4.35499300	2.96164600	-1.10510000
C	5.00761000	0.84014800	0.54950000
H	3.22755400	1.31166400	1.62463700
C	5.50629900	2.22451700	-1.34326300
H	4.10578800	3.79194100	-1.75335300
C	5.83892500	1.16766900	-0.51065600
H	5.24830500	0.00297000	1.19021800
H	6.14367800	2.48030900	-2.17846000
H	6.73618100	0.59248900	-0.69293900

6-Ag

Ag	-0.98830800	0.11364000	-0.31216200
N	-0.71518800	-1.99838400	-0.60861900
N	-1.10447700	2.24804700	-0.07306600
C	-5.36298300	-1.12197600	0.28674900
C	-6.48421900	-1.92780400	0.48217400
C	-7.73773800	-1.36239900	0.62683900
C	-7.89028000	0.01662600	0.57727500
C	-6.78882500	0.83035900	0.38622200
C	-5.51696800	0.27679900	0.24098800
C	-4.08115600	-1.71616300	0.12153900
C	-3.00268700	-2.22067600	-0.04112900
C	-1.73688800	-2.82884000	-0.28403400
C	-4.39177600	1.12965100	0.06443100
C	-3.44733900	1.86283000	-0.05969400
C	-2.35351600	2.77411600	-0.14506500
C	0.48029300	-2.52504800	-0.92446100
C	0.70937700	-3.91017900	-0.82149500
C	-0.32445600	-4.72968700	-0.45570000
C	-1.58407700	-4.19491600	-0.19689100
C	-0.04395100	3.07715600	-0.06324100
C	-0.22431200	4.46763000	-0.20610300
C	-1.49048200	4.97562400	-0.31574300
C	-2.59019400	4.12527600	-0.26764500
H	-6.36025100	-3.00047200	0.51701100
H	-8.59803700	-1.99863800	0.77748000
H	-6.90139600	1.90425300	0.34975100
H	-8.86983300	0.45890700	0.68892300
H	1.68526500	-4.31502200	-1.03469200
H	-2.42311600	-4.81585900	0.07233400
H	0.62590800	5.12836300	-0.22183600
H	-0.16326200	-5.79546400	-0.37806700
H	-1.63294900	6.04131700	-0.42461200
H	-3.60155600	4.49419200	-0.32163500
N	1.44057700	-1.67448400	-1.34813300
H	1.18460700	-0.70380600	-1.42209900
N	1.18955000	2.52765200	0.06514300
H	1.21331000	1.56475000	0.36282800
C	2.85648500	-1.97818200	-1.41139900
H	2.99248800	-2.87010700	-2.02741900
C	2.39489200	3.31120000	0.34959600
H	2.42447100	4.12537400	-0.37366300
C	3.55251900	-0.81790400	-2.11445200
H	4.61939800	-1.01096700	-2.19202100
H	3.14584500	-0.68433200	-3.11626400

H	3.42320500	0.10523200	-1.54753100
C	2.37820900	3.90080600	1.76091400
H	3.27318700	4.49935000	1.92385500
H	1.50678600	4.53656100	1.91118200
H	2.35234600	3.10986800	2.50963500
C	3.46218000	-2.25134300	-0.04336400
C	2.84933200	-1.83496800	1.13023400
C	4.68390700	-2.91049900	0.03567800
C	3.44682600	-2.06655600	2.36077900
H	1.89900000	-1.31873900	1.08966700
C	5.28740100	-3.13859000	1.26161200
H	5.16953300	-3.24763700	-0.87211900
C	4.66872900	-2.71680200	2.43089600
H	2.95627400	-1.73513200	3.26589600
H	6.23872800	-3.65089700	1.30589300
H	5.13544800	-2.89779000	3.38922700
C	3.63160600	2.47213400	0.10691300
C	3.96660600	1.41781700	0.95061600
C	4.48180200	2.77916100	-0.94956300
C	5.13110100	0.69379600	0.74882400
H	3.32205500	1.15234700	1.77920400
C	5.65106800	2.06071400	-1.15033800
H	4.23262800	3.59489500	-1.61625600
C	5.98046300	1.01903600	-0.29694300
H	5.36771700	-0.13225200	1.40512400
H	6.30364400	2.31638400	-1.97382100
H	6.89009700	0.45526000	-0.45174600

7-I

I	0.79046400	0.12102300	0.13319200
N	0.64326700	-2.21627700	0.42810800
N	1.14156700	2.33623000	-0.23797000
C	5.40803000	-1.17933000	-0.08108100
C	6.53054400	-2.00559900	-0.10319700
C	7.80133000	-1.45973700	-0.09791700
C	7.96958200	-0.08169300	-0.07170900
C	6.86658200	0.75203300	-0.05283800
C	5.57872400	0.21736300	-0.05847300
C	4.10094100	-1.74323900	-0.08105500
C	2.99041900	-2.20207200	-0.07900700
C	1.72013000	-2.85037600	-0.11831600
C	4.44671700	1.07919900	-0.04828700
C	3.48039400	1.79376700	-0.04428200
C	2.42488300	2.74849700	-0.01881900
C	-0.54051900	-2.86992300	0.52057900
C	-0.70980400	-4.08448300	-0.18992400
C	0.36475600	-4.67559900	-0.78850900
C	1.62464900	-4.08529300	-0.71115200
C	0.13976200	3.24577400	-0.30890100
C	0.41362000	4.59449600	-0.00035100
C	1.69102300	4.98986000	0.28051200
C	2.72851600	4.06387700	0.24286900
H	6.39474000	-3.07725800	-0.12184300
H	8.66362000	-2.11080300	-0.11368900
H	6.99094200	1.82516000	-0.03534100
H	8.96289600	0.34357300	-0.06710000
H	-1.66868900	-4.56701300	-0.23442700
H	2.50283600	-4.55248800	-1.12618700
H	-0.37476900	5.32630000	-0.05027700
H	0.23816300	-5.61783600	-1.30230600
H	1.89839800	6.02931800	0.49014100
H	3.75369100	4.34777800	0.41644800
N	-1.52336100	-2.36295900	1.30161200
N	-1.10217000	2.84341500	-0.67945200
C	-2.89231700	-2.89701800	1.15479800
H	-2.83490000	-3.98475900	1.20960900
C	-2.28397300	3.61518300	-0.26330200

H	-1.96168200	4.22875300	0.57483800
C	-3.83093200	-2.47375400	2.27879000
H	-4.81055400	-2.89745200	2.06622500
H	-3.50569500	-2.85309700	3.24546000
H	-3.93910300	-1.39312300	2.32957800
C	-2.79780500	4.55076300	-1.35140900
H	-3.62179800	5.14857400	-0.96544100
H	-2.00443600	5.22406600	-1.67261000
H	-3.15576700	4.01937100	-2.23128100
C	-3.48313000	-2.51610700	-0.19017900
C	-3.40072100	-1.21028800	-0.65814800
C	-4.15564100	-3.46349100	-0.95112100
C	-3.97154700	-0.86204100	-1.87129400
H	-2.89895700	-0.45598500	-0.06390100
C	-4.73060500	-3.11761500	-2.16523000
H	-4.22978600	-4.48384700	-0.59400900
C	-4.63599100	-1.81488500	-2.63010700
H	-3.90708000	0.15938800	-2.22040300
H	-5.24642800	-3.86733300	-2.74936200
H	-5.07921900	-1.54213000	-3.57790100
C	-3.32667800	2.67237700	0.31804500
C	-4.53423600	2.39564600	-0.30539100
C	-3.06446900	2.09224800	1.55774500
C	-5.46743700	1.56279400	0.29980800
H	-4.76619800	2.83181200	-1.26681800
C	-3.99653300	1.27346600	2.16860500
H	-2.12399300	2.30004200	2.05412600
C	-5.20535400	1.00573100	1.53855600
H	-6.40134400	1.35254000	-0.20267600
H	-3.78360600	0.84922200	3.14070500
H	-5.93465000	0.36281000	2.01169800
C	-1.22424600	-1.41549200	2.36438600
H	-0.15437200	-1.39767900	2.54194900
H	-1.55955700	-0.40592200	2.12610800
H	-1.70521800	-1.73369700	3.28428200
C	-1.30651700	1.87016900	-1.74205900
H	-0.36224100	1.63670600	-2.22396600
H	-1.75286200	0.94866400	-1.37578000
H	-1.96501200	2.29800900	-2.49690000

7-Ag

Ag	1.01533900	0.07341300	0.10011800
N	0.70462600	-2.11299000	0.37546800
N	1.16094700	2.25675100	-0.25552500
C	5.43040500	-1.14244400	-0.09128300
C	6.55871000	-1.96021400	-0.13192400
C	7.82516100	-1.40876300	-0.06379800
C	7.98279200	-0.03356100	0.04593500
C	6.87392400	0.79172000	0.08387100
C	5.59025300	0.25163000	0.01411800
C	4.12728900	-1.70973900	-0.14397800
C	3.01929700	-2.17441300	-0.16683800
C	1.73869900	-2.80417500	-0.17532900
C	4.45216900	1.10369200	0.03275100
C	3.48604900	1.81854700	0.02495700
C	2.40478200	2.74735600	-0.00829600
C	-0.49239300	-2.72868500	0.49747500
C	-0.70332600	-3.98110900	-0.13349300
C	0.34164400	-4.62975000	-0.72678600
C	1.61139700	-4.06023300	-0.71856000
C	0.13482200	3.12502100	-0.36796900
C	0.34779800	4.50844900	-0.15899900
C	1.59910900	4.97079900	0.13454500
C	2.66660600	4.08141200	0.20329700
H	6.43087500	-3.02984100	-0.21417700
H	8.69203300	-2.05313800	-0.09512900
H	6.99057100	1.86277300	0.16475700

H	8.97260200	0.39652600	0.10028100
H	-1.67405900	-4.43957200	-0.14554900
H	2.46868600	-4.56012100	-1.13949400
H	-0.45924100	5.21036600	-0.27924400
H	0.17910500	-5.59303300	-1.18909400
H	1.76086900	6.02950500	0.27916300
H	3.67388300	4.40970000	0.40239400
N	-1.46296900	-2.14428900	1.24983400
N	-1.09552300	2.65351000	-0.70442400
C	-2.82020200	-2.72593500	1.24725600
H	-2.70031800	-3.80266300	1.35980800
C	-2.30501200	3.45746100	-0.48858200
H	-2.03876400	4.22938100	0.22871900
C	-3.67787700	-2.29042600	2.42887400
H	-4.60200600	-2.86453200	2.39171600
H	-3.18836800	-2.49883900	3.37938400
H	-3.94479200	-1.23690000	2.37845700
C	-2.76586500	4.14622800	-1.76665600
H	-3.62448200	4.78548400	-1.56741600
H	-1.96103900	4.76501400	-2.16061500
H	-3.04719800	3.43285900	-2.54081400
C	-3.54474000	-2.46259000	-0.06079700
C	-3.67110200	-1.16851600	-0.55251100
C	-4.12112400	-3.50829600	-0.77024800
C	-4.33710000	-0.92910000	-1.74287900
H	-3.26552200	-0.33460400	0.00616300
C	-4.79544000	-3.27236400	-1.95976400
H	-4.04361800	-4.52088000	-0.39230200
C	-4.89792100	-1.98118900	-2.45332900
H	-4.42996600	0.08416900	-2.10933700
H	-5.23501000	-4.09832000	-2.50193000
H	-5.41728600	-1.79382500	-3.38302500
C	-3.36516400	2.62828700	0.22122100
C	-4.61530300	2.36881300	-0.32058300
C	-3.08133500	2.15868900	1.50255200
C	-5.56646100	1.65932400	0.40276000
H	-4.86588300	2.72127600	-1.31124900
C	-4.02851000	1.46022800	2.22849900
H	-2.10908600	2.35728100	1.93710300
C	-5.27896700	1.20744600	1.67815000
H	-6.53342200	1.46030100	-0.03794400
H	-3.79560300	1.11750800	3.22764900
H	-6.02029300	0.65814300	2.24168800
C	-1.16875600	-0.94675500	2.00920000
H	-0.17861200	-1.01874100	2.45357600
H	-1.22052600	-0.03658500	1.40144300
H	-1.88518000	-0.83368000	2.81247500
C	-1.26068400	1.41569100	-1.43907700
H	-1.37767500	0.54550200	-0.79046900
H	-2.16292500	1.48032000	-2.04284000
H	-0.41997700	1.25265700	-2.10990700

5. References

1. M. Curini, F. Epifano, F. Maltese, M. C. Marcotullio, A. Tubaro, G. Altinier, S. P. Gonzales and J. C. Rodriguez, *Bioorg. Med. Chem. Lett.*, 2004, **14**, 2241-2243.
2. L. Zhou, J. Chen, C. K. Tan and Y. Y. Yeung, *J. Am. Chem. Soc.*, 2011, **133**, 9164-9167.
3. M. C. Dobish and J. N. Johnston, *J. Am. Chem. Soc.*, 2012, **134**, 6068-6071.
4. J. Contreras-Garcia, E. R. Johnson, S. Keinan, R. Chaudret, J. P. Piquemal, D. N. Beratan and W. Yang, *J Chem Theory Comput*, 2011, **7**, 625-632.
5. E. R. Johnson, S. Keinan, P. Mori-Sanchez, J. Contreras-Garcia, A. J. Cohen and W. Yang, *J. Am. Chem. Soc.*, 2010, **132**, 6498-6506.
6. J. D. Chai and M. Head-Gordon, *Phys. Chem. Chem. Phys.*, 2008, **10**, 6615-6620.
7. T. H. Dunning, *J. Chem. Phys.*, 1989, **90**, 1007-1023.
8. D. E. Woon and T. H. Dunning, *J. Chem. Phys*, 1995, **103**, 4572-4585.
9. J. Tomasi, B. Mennucci and R. Cammi, *Chem. Rev.*, 2005, **105**, 2999-3093.
